# Efficacy and tolerability of antidepressants in individuals suffering from physical conditions and depressive disorders: network meta-analysis

**DOI:** 10.1192/bjp.2025.18

**Published:** 2025-08

**Authors:** Beatrice De Luca, Andrea Canozzi, Carlotta Mosconi, Chiara Gastaldon, Davide Papola, Alessia Metelli, Federico Tedeschi, Francesco Amaddeo, Marianna Purgato, Marco Solmi, Corrado Barbui, Giovanni Vita, Giovanni Ostuzzi

**Affiliations:** 1 WHO Collaborating Centre for Research and Training in Mental Health and Service Evaluation, Department of Neurosciences, Biomedicine and Movement Sciences, Section of Psychiatry, University of Verona, Verona, Italy; 2 Department of Global Health and Social Medicine, Harvard Medical School, Boston, USA; 3 SCIENCES Lab, Department of Psychiatry, University of Ottawa, Ottawa, Canada; 4 Regional Centre for the Treatment of Eating Disorders and On Track: The Champlain First Episode Psychosis Program, Department of Mental Health, The Ottawa Hospital, Ottawa, Canada; 5 Ottawa Hospital Research Institute (OHRI) Clinical Epidemiology Program, University of Ottawa, Ottawa, Canada; 6 Department of Child and Adolescent Psychiatry, Charité Universitätsmedizin, Berlin, Germany

**Keywords:** Antidepressants, comorbidity, depressive disorders, evidence-based mental health, network meta-analyses

## Abstract

**Background:**

Antidepressants are effective for depression, but most evidence excludes individuals with comorbid physical conditions.

**Aims:**

To assess antidepressants’ efficacy and tolerability in individuals with depression and comorbid physical conditions.

**Methods:**

Systematic review and network meta-analysis of randomised controlled trials (RCTs). Co-primary outcomes were efficacy on depressive symptoms and tolerability (participants dropping out because of adverse events). Bias was assessed with the Cochrane Risk-of-Bias 2 tool and certainty of estimates with the Confidence in Network Meta-Analysis approach. A study protocol was registered in advance (https://osf.io/9cjhe/).

**Results:**

Of the 115 included RCTs, 104 contributed to efficacy (7714 participants) and 82 to tolerability (6083 participants). The mean age was 55.7 years and 51.9% of participants were female. Neurological and cardiocirculatory conditions were the most represented (26.1% and 18.3% of RCTs, respectively). The following antidepressants were more effective than placebo: imipramine, nortriptyline, amitriptyline, desipramine, sertraline, paroxetine, citalopram, fluoxetine, escitalopram, mianserin, mirtazapine and agomelatine, with standardised mean differences ranging from −1.01 (imipramine) to −0.34 (escitalopram). Sertraline and paroxetine were effective for the largest number of ICD-11 disease subgroups (four out of seven). In terms of tolerability, sertraline, imipramine and nortriptyline were less tolerated than placebo, with relative risks ranging from 1.47 (sertraline) to 3.41 (nortriptyline). For both outcomes, certainty of evidence was ‘low’ or ‘very low’ for most comparisons.

**Conclusion:**

Antidepressants are effective in individuals with comorbid physical conditions, although tolerability is a relevant concern. Selective serotonin reuptake inhibitors (SSRIs) have the best benefit–risk profile, making them suitable as first-line treatments, while tricyclics are highly effective but less tolerated than SSRIs and placebo.

Major depressive disorder (MDD) has a point prevalence of approximately 5% in the general population.^1,2^ It is one of the most common comorbidities in individuals with physical conditions, with an estimated prevalence of up to 20%.^3–5^ In these populations, MDD is associated with worse quality of life, daily functioning, overall disease burden, medication adherence, disease progression and suicide risk.^4,6–9^

Antidepressants are prescribed in about 15–30% of individuals with physical conditions.^10–12^ Although extensive meta-analyses on antidepressants in the general population are available,^12^ most of the included trials have explanatory designs and typically exclude individuals with physical conditions, which raises concerns about the generalisability of these findings.

## Rationale of the antidepressant treatment

Depression is a multifactorial disorder encompassing biological, psychological and social components. Biologically, various physical conditions can significantly influence inflammation, immunity, endocrine and metabolic pathways, stress responses and neural activity, increasing vulnerability to depression.^13–15^ Moreover, each physical condition can activate different sets of etiological pathways. Similarly, various physical conditions may be associated with different psychological experiences based on their intrinsic features, such as the degree of social and work impairment or reduced life expectancy. For this reason, the efficacy and tolerability of antidepressants might be different in these individuals compared to the general population.

Treating depression and anxiety and enhancing quality of life in individuals with physical conditions is crucial, but antidepressants can pose risks of serious side-effects that may worsen the underlying illness.^16^ A large umbrella review^17^ examining 176 systematic reviews across 43 physical diseases concluded that antidepressants are effective and safe in people with physical conditions and comorbid depression. However, the qualitative approach employed in this analysis prevents a clear estimation of the clinical effect of antidepressants and a comparison between available agents.

On these grounds, we performed a systematic review and network meta-analysis (NMA) to assess the comparative efficacy and tolerability of antidepressants in adults suffering from depression and comorbid physical conditions.

## Method

This study was conducted and reported according to the Preferred Reporting Items for Systematic Reviews and Meta-Analyses (PRISMA) guidelines for NMA^18^ (see Supplement A, pp. 2–4). The study protocol was registered in advance in the online repository Open Science Forum (https://osf.io/9cjhe/). Changes to the original protocol are reported in Supplement S (p. 179).

### Search strategy and selection criteria

We performed a systematic review and NMA including randomised controlled trials (RCTs) comparing antidepressants with placebo and between each other. Only studies with at least 4 weeks of follow-up were included, as this is a reasonable timeframe within which we expect to observe a clinical response to antidepressants. We searched for RCTs that included adults (≥18 years of age) with a primary diagnosis of one or more physical conditions and a diagnosis of acute depressive episode (including single episode of depression, recurrent depressive disorder, mixed depressive and anxiety disorder or adjustment disorder with clinically relevant depressive symptoms) as assessed by clinical examination with or without the support of validated rating scales (e.g. Hamilton Depression Rating Scale (HDRS), Montgomery–Åsberg Depression Rating Scale (MADRS), Beck Depression Inventory (BDI)) or manualised diagnostic criteria (e.g. the *Diagnostic and Statistical Manual of Mental Disorders* (DSM)^19^ and the International Classification of Diseases (ICD)^20^). If the presence of clinically relevant depression was not clearly described, we assessed its presence according to baseline mean scores on validated rating scales measuring depression, considering the following validated cut-offs indicating at least ‘moderate’ depression: HDRS^21^ ≥ 14; MADRS^22^ ≥ 20; BDI version I (BDI-I)^23^ ≥ 10 and version II (BDI-II)^24^ ≥ 14; Major Depression Inventory (MDI)^25^ ≥ 26; Center for Epidemiologic Studies Depression Scale (CES-D)^26^ ≥ 16; Brief Zung Self-Rating Depression Scale (BZSRD)^27^ ≥ 44; Hospital Anxiety and Depression Scale (HADS)^28^ ≥ 11; Patient Health Questionnaire (PHQ-9)^29^ ≥ 10; Symptom Checklist-90-Revised (SCL-90-R)^30^ depression subscale ≥ 20. In the case of studies with mixed populations (i.e. depression and anxiety), we included only those with at least 80% of participants suffering from clinically relevant depression, as defined above.

We excluded conditions for which a clear pathogenesis could not be identified (e.g. physically unexplained symptoms, functional symptoms), or conditions for which a relevant psychiatric or psychological component may be particularly relevant (e.g. somatic symptoms disorders, fibromyalgia, irritable bowel syndrome). We also excluded minor depression, dysthymia, prolonged grief disorder or complicated bereavement. No exclusion criteria were applied in terms of setting (e.g. in- and out-patient settings; physical, surgical, psychiatric setting) or type of antidepressant, as even those that are uncommonly prescribed or no longer marketed (e.g. nomifensine) can indirectly contribute to estimate the differential effect between other antidepressants.

We searched electronic databases (MEDLINE, Embase, PsycINFO, CENTRAL, CINAHL), databases of regulatory agencies (e.g. Food and Drug Administration, European Medicines Agency) and trial registers (e.g. clinicaltrials.gov, the World Health Organization’s International Clinical Trials Registry Platform) from inception until 30 April 2024, without language restrictions (see Supplement B for the full research syntax). Two review authors (B.D.L., A.C.) independently assessed titles and abstracts of all retrieved records, and then the full texts of all potentially eligible records. Disagreements were resolved by discussion and consensus with a third review author (G.O.). Data extraction was performed between 1 June 2024 and 15 July 2024 by two review authors (B.D.L., A.C.) independently, in agreement with the recommendations of the Cochrane Handbook for Systematic Reviews of interventions.^31^ Two review authors (G.V., A.C.) independently assessed the risk of bias of included studies using the Cochrane Risk-of-Bias tool, version 2 (RoB2).^32^ Disagreements were resolved by discussion and consensus with a third review author (G.O., C.B.).

### Outcomes

The primary outcomes were (a) depressive symptoms measured as the mean change score (or, alternatively, the end-point score)^33^ on validated rating scales (HDRS, BDI, MADRS or any other) by the end of the trial, and (b) tolerability, defined as the number of individuals withdrawing from treatment because of adverse events by the end of the trial, as a proportion of the total number of randomised participants.

Secondary outcomes included the following dichotomous outcomes: response; remission; acceptability; death caused by worsening of the physical condition; death from any cause; serious adverse events (SAEs); and the following continuous outcomes: anxiety symptoms; quality of life; social functioning.

### Data analysis

Analyses were conducted with Stata (version 18.5 for macOS), and with the R programming language (R version 4.4.0 for macOS; R Foundation for Statistical Computing, Vienna, Austria; https://cran.r-project.org/) within the R Studio integrated development environment. For each outcome and comparison, we performed a standard pairwise, random-effects meta-analysis and a NMA with a random-effects model in a frequentist framework, using the R packages *netmeta* and the Stata package *mvmeta*.^34,35^ For dichotomous outcomes, risk ratios were log-transformed and pooled using a conventional normal-normal random-effects model with 95% confidence intervals, applying a strict intention-to-treat (ITT) approach (all randomly assigned participants as the denominator). For continuous outcomes, we pooled mean differences if all trials used the same rating scale; otherwise, we employed standardised mean differences (SMDs), including all participants that contributed to the analysis in the original trial. We calculated SMDs as the ratio between the mean difference between groups and the standard deviation of outcome among participants, according to the Cochrane Handbook.^31^ If we could not retrieve missing data from trial authors, we imputed them with validated statistical methods, including imputing response and remission according to mean rating scale scores, standard deviations and validated cut-offs.^31,36,37^

For the NMA, common heterogeneity across all comparisons was assumed in each network, and global heterogeneity was assessed using τ^2^ (low, τ ² ≤ 0.010; moderate, 0.010 < τ ² ≤ 0.242; and high, τ ² > 0.242), estimated by the DerSimonian–Laird method through the R *netmeta* function^38^ and *I*² (low, 0–40%; moderate, 30–60%; substantial, 50–90%; and considerable, 75–100%).^31^

To assess the assumption of transitivity, we compared the distribution of the following variables across treatment strategies: sample size; year of publication; follow-up duration; blinding (double blind versus open label); setting (in-patient, out-patient or mixed settings); industry sponsorship; mean age; percentage of female participants; mean psychopathology score at baseline; mean duration of depression; depression as the primary study outcome; mean number of co-occurring physical conditions; mean severity of the physical condition at baseline (according to a classification derived from the Severity of Illness Instrument).^39^

For the primary outcomes, we visually inspected boxplots on the distribution of continuous variables across treatments and assessed whether imbalances were large enough to threaten the transitivity assumption according to both the Kruskal–Wallis test and meta-regression analyses.

We evaluated the presence of inconsistency, defined as the statistical disagreement between direct and indirect evidence of a treatment comparison, by comparing direct and indirect evidence within each closed loop and comparing the goodness of fit for a NMA model that assumes consistency with a model that allows for inconsistency in a ‘design-by-treatment interaction model’ framework by using the R Studio *netmeta* package and *decomp.design* and *netsplit* commands.^34,39^ Where we found evidence of inconsistency, we investigated this further using both a node-splitting and a side-splitting approach between comparisons. For each outcome, we calculated the probability of each antidepressant of being at each possible rank and the treatment hierarchy by means of *p*-scores (mean normalised rank probabilities), which reflect the extent of certainty that a treatment is better than competing treatments, based on the point estimates and standard errors.^40^ If a comparison included ten studies or more, we assessed publication bias by visually inspecting the funnel plot, tested them for asymmetry with the Egger’s regression test and investigated possible reasons for funnel plot asymmetry.

We performed sensitivity analyses excluding RCTs with the following characteristics: placebo controlled; not double blind; with overall high risk of bias according to RoB2; with follow-up shorter than 3 months; efficacy on depressive symptoms was not the primary study aim; without a formal diagnosis of major depression (post hoc); with a small sample size (<50 participants; post hoc analysis); recruiting participants with illness of less than moderate severity. Furthermore, we performed meta-regression analyses on the same variables assessed for transitivity, to identify possible treatment effect moderators.

We also performed the following subgroup analyses for the primary outcomes, provided that at least three RCTs can be pooled together: analysing each subgroup of diseases (according to the ICD-11 classification) and each physical condition separately; grouping treatments according to their class, namely selective serotonin reuptake inhibitors (SSRIs), selective serotonin and noradrenaline reuptake inhibitors (SNRIs), tricyclic antidepressants (TCAs) and other antidepressants.

For the primary outcomes, certainty of the pooled evidence was assessed using the Confidence in Network Meta-Analysis (CINeMA) approach.^41^

## Results

**Fig. 1:**
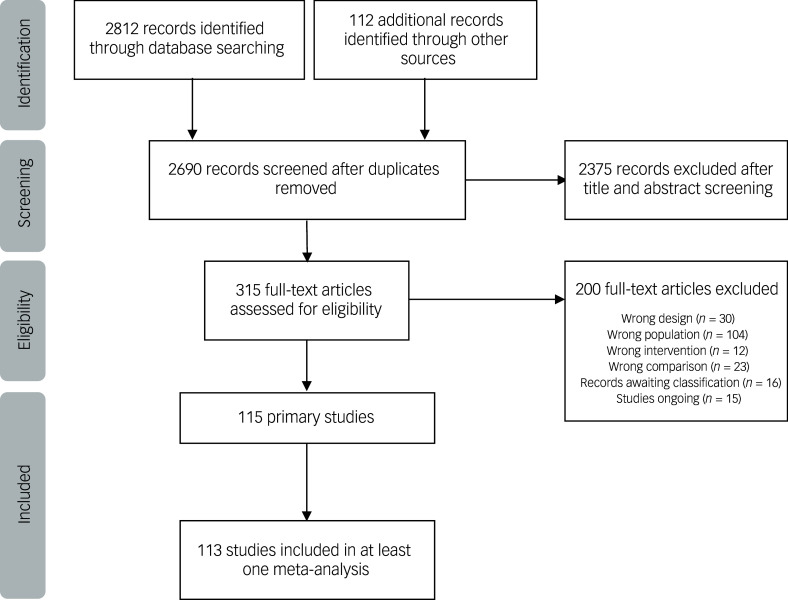
Process of study selection.

We identified 2924 records after the database and hand-search. After removing duplicates and examining titles and abstracts, we selected 315 records for full-text assessment. Of these, 115 primary studies were eligible for inclusion.^42–156^ Of these, 104 RCTs including 7714 participants contributed to the analysis of efficacy, and 82 RCTs including 6083 participants contributed to the analysis of tolerability (Fig. 1; Supplement C, pp. 8–21). The study by Rampello et al^156^ met the inclusion criteria, but was not included in the meta-analysis because of concerns regarding the quality of the data presented. Results showed an exceptionally large effect size (SMD 5.9) for reboxetine versus placebo in post-stroke depression, which we deemed clinically improbable and possibly caused by a reporting error of the variance. Unfortunately, we could not retrieve additional information from the study authors to clarify this issue. Detailed characteristics of included studies are reported in Supplement D (pp. 22–7). The mean sample size of included studies was 80.9 individuals (median: 51; interquartile range (IQR): 31–88) with 55 (47.4%) studies including ≤50 participants. For the primary outcome of efficacy, the mean age of included participants was 56.1 years (s.d. 11.45) and the mean proportion of female participants was 51.8%, while for tolerability the mean age was 55.6 years (s.d. 12.45) and the mean proportion of female participants was 52.2%. According to the RoB2, 33 studies (31.7%) for the primary outcome of efficacy and six studies (7.3%) for tolerability had an overall high risk of bias (Supplement E, pp. 28–9). Table 1 describes the characteristics of studies included in the two primary analyses. The transitivity assumption was not violated for any of the potential effect modifiers analysed (Supplement F, pp. 30–2).

**Table 1:** Characteristics of studies contributing to the co-primary outcomes

	Efficacy network	Tolerability network
Number of studies, *n*	104	82
Number of individuals, *n*	7714	6083
Number of treatments, *n*	24	20
Female participants, *n* (%)	4036 (51.8%)	2935 (52.2%)
Age, mean (s.d.)	56.09 (11.45)	55.66 (12.45)
Physical diagnosis		
Circulatory system diseases	16 (15.4%)	13 (16.3%)
Ear/mastoid process diseases	1 (1.0%)	1 (1.3%)
Endocrine/nutritional/metabolic diseases	12 (11.5%)	9 (11.3%)
Genito-urinary system diseases	4 (3.8%)	4 (5.0%)
Infectious diseases	9 (8.7%)	7 (8.8%)
Injury/poisoning diseases	1 (1.0%)	0
Mixed/multiple diseases	1 (1.0%)	0
Musculoskeletal/connective tissue diseases	7 (6.7%)	6 (7.5%)
Oncological diseases	8 (7.7%)	8 (10%)
Nervous system diseases	29 (27.9%)	21 (26.3%)
Neurocognitive diseases	9 (8.7%)	7 (8.8%)
Respiratory diseases	7 (6.7%)	4 (5.0%)
Severity of physical diagnosis, *n* (%)		
Moderate	51 (49%)	32 (40%)
Important	32 (30.8%)	30 (37.5%)
Very important	19 (18.3%)	12 (15.0%)
Unknown	2 (1.9%)	6 (7.5%)
Sample size, mean (s.d.)	81.01 (97.15)	74.14 (73.80)
Follow-up weeks, mean (s.d.)	11.92 (10.99)	11.27 (7.65)
Blinding, *n* (%)		
Double blind	98 (94.2%)	76 (95.0%)
Open label	6 (5.8%)	4 (5.0%)
Year of publication, *n* (%)		
1980–1989	5 (4.8%)	7 (8.8%)
1990–1999	22 (21.2%)	19 (23.8%)
2000–2009	42 (40.4 %)	30 (37.5%)
2010–2019	33 (31.7%)	23 (28.8%)
2020–2024	2 (1.9%)	1 (1.3%)
Setting, *n* (%)		
In-patients	13 (12.5%)	7 (8.8%)
Out-patients	65 (62.5%)	52 (65.0%)
Mixed	6 (5.8%)	4 (5.0%)
Not reported	20 (19.2%)	17 (21.3%)
Country income, *n* (%)		
High income	77 (74.0%)	53 (66.3%)
Upper-middle income	17 (16.3%)	12 (5.0%)
Lower-middle income	6 (5.8%)	3 (3.8%)
Mixed	4 (3.8%)	2 (2.5%)
Study design, *n* (%)		
Placebo controlled	82 (79.4%)	62 (80.0%)
Active comparator only	22 (20.6%)	18 (20.0%)
Antidepressant class (study arms), *n* (%)		
TCA	12 (11.5%)	11 (13.8%)
SSRI	56 (53.8%)	43 (53.8%)
SNRI	4 (3.8%)	1 (1.3%)
Other antidepressants	6 (5.8%)	3 (3.8%)
Mixed	26 (25.0%)	22 (27.5%)

TCA, tricyclic antidepressants; SSRI, selective serotonin reuptake inhibitors; SNRI, serotonin and noradrenaline reuptake inhibitors.

**Fig. 2:**
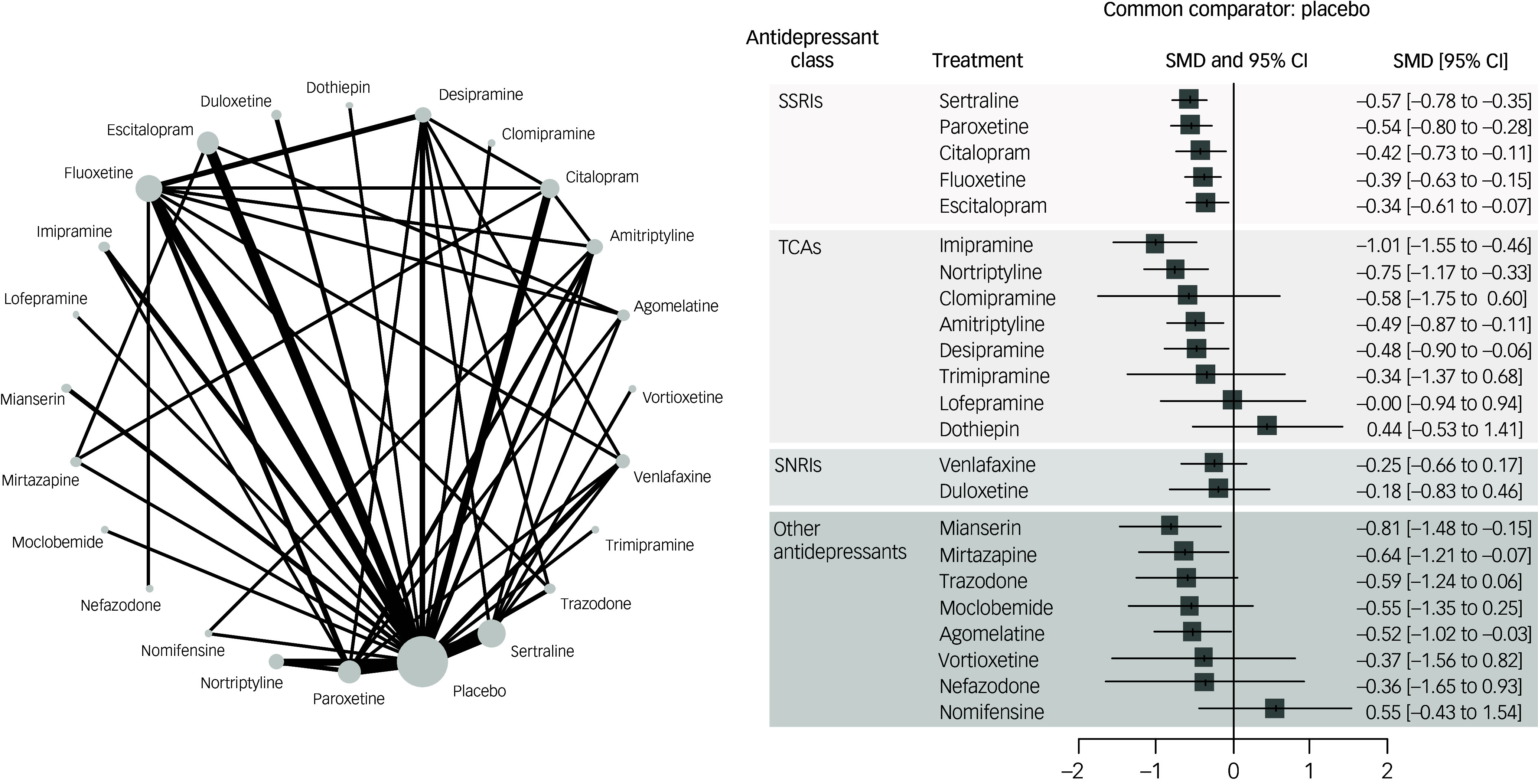
Network map and forest plot for the primary outcome efficacy. SMD, standardised mean difference; SSRI, selective serotonin reuptake inhibitor; TCA, tricyclic antidepressant; SNRI, serotonin and noradrenaline reuptake inhibitor.

In terms of efficacy (mean change at rating scales measuring depression), the following antidepressants outperformed placebo (Fig. 2; ordered by effect size): imipramine, nortriptyline, amitriptyline, desipramine among TCAs; sertraline, paroxetine, citalopram, fluoxetine, escitalopram among SSRIs; mianserin, mirtazapine, agomelatine among other antidepressants, with SMDs ranging from −1.01 (imipramine) to −0.34 (escitalopram). Head-to-head comparisons showed relatively few statistically significant differences between antidepressants (Supplement G, pp. 39–40). Notably, imipramine outperformed fluoxetine and venlafaxine. *P*-scores showed imipramine as the best-performing treatment, followed by mianserin and nortriptyline.

Overall, the NMA showed moderate heterogeneity (τ^2^ = 0.16; *I* ^2^ = 70.0%), and no overall incoherence emerged according to the global approach (design-by-treatment test, *P* = 0.30), while the local separate indirect from direct evidence (SIDE) approach showed significant inconsistency of two over 38 comparisons (agomelatine versus escitalopram; placebo versus fluoxetine).

Certainty of evidence according to the CINeMA approach was ‘low’ or ‘very low’ for most comparisons, with few exceptions (i.e. ‘moderate’ certainty for the comparisons desipramine versus placebo, dothiepin versus imipramine, imipramine versus nomifensine) (Supplement G, pp. 44–7).

Sensitivity analyses (Supplement G, pp. 48–52) removing studies not blinded, with follow-up durations shorter than 3 months, recruiting individuals without a formal diagnosis of depression, recruiting individuals with illness of less than moderate severity, for which the efficacy on depressive symptoms was not the primary outcome and those with a sample size <50 did not show relevant differences compared with the primary analysis in terms of heterogeneity, which instead was moderately reduced after removing placebo-controlled studies (τ^2^ = 0.02; *I* ^2^ = 18.6%). Effect estimates from sensitivity analyses did not change significantly compared to the primary analysis. Meta-regression analyses showed that increasing baseline severity of the physical condition was associated with a smaller treatment effect (*β* = −0.18; *P* = 0.04).

When performing subgroups on different clusters of diseases (Supplement G, pp. 52–9), sertraline and paroxetine outperformed placebo in four out of seven physical conditions; fluoxetine in three; nortriptyline and citalopram in two; agomelatine, imipramine, trazodone, desipramine, amitriptyline, mirtazapine, mianserin and moclobemide in one (Table 2). No consistency issues emerged in any of the subgroup analyses; heterogeneity was substantial for circulatory system diseases (*I* ^2^ = 85.5%, which remained high also after analysing RCTs on ischemic heart disease separately) (Table 2; Supplement G, pp. 52–9). After grouping antidepressants in classes, TCAs, other antidepressants and SSRIs outperformed placebo in terms of efficacy, and no differences emerged between classes. Heterogeneity was moderate, and no inconsistency issues emerged (Table 2, Supplement I, pp. 86–94).

**Table 2:** Subgroup analyses

Subgroup	Conditions included	Outcome	Studies, *N*	Participants, *N*	Treatments, *N*	Significant differences with placebo			Heterogeneity	Inconsistency
						Antidepressant	ESM	Effect size (95% CI)		
Antidepressant classes	All	Efficacy	99	7573	5	TCA	SMD	0.55 (−0.74 to −0.36)	τ^2^ = 0.14 *I* ^2^ = 68.9%	Global: *P* = 0.54 Local: 0/9
						Other antidepressants	SMD	−0.53 (−0.78 to −0.29)		
						SSRI	SMD	0.46 (−0.58 to −0.35)		
		Tolerability	79	6039	5	TCA	Risk ratio	1.84 (1.35 to 2.50)	τ^2^ = 0 *I* ^2^ = 0%	Global: *P* = 0.99 Local: 0/9
						SSRI	Risk ratio	1.37 (1.09 to 1.70)		
Circulatory system diseases	IHD, hypertension	Efficacy	16	2075	8	Nortriptyline	SMD	−1.56 (−2.90 to −0.23)	τ^2^ = 0.21 *I* ^2^ = 85.5%	Global: *P* = 0.12 Local: 0/3
						Paroxetine	SMD	–1.51 (–2.40 to −0.63)		
						Fluoxetine	SMD	−0.97 (−1.64 to −0.29)		
						Sertraline	SMD	−0.68 (−1.13 to −0.23)		
		Tolerability	11	1971	6	Sertraline	Risk ratio	1.67 (1.07 to 2.62)	τ^2^ = 0 *I* ^2^ = 0%	Not applicable
Endocrinological diseases	Diabetes, hypothyroidism, PCOS	Efficacy	12	643	8	Escitalopram	SMD	−2.49 (−3.44 to −1.53)	*τ* ^2^ = 0.04 *I* ^2^ = 34.4%	Global: *P* = 0.79 Local: 0/7
						Agomelatine	SMD	−0.99 (−1.50 to −0.48)		
						Paroxetine	SMD	−0.65 (−1.14 to −0.16)		
						Sertraline	SMD	−0.54 (−0.94 to −0.14)		
		Tolerability	9	558	5	None			*τ* ^2^ = 0 *I* ^2^ = 0%	Global: *P* = 0.99 Local: 0/8
Infectious diseases	HIV, AIDS	Efficacy	8	491	7	Imipramine	SMD	−1.15 (−1.57 to −0.73)	τ^2^ = 0.02 *I* ^2^ = 17.6%	Global: *P* = 0.31 Local: 0/2
						Paroxetine	SMD	−0.77 (−1.34 to −0.19)		
						Fluoxetine	SMD	−0.40 (−0.77 to −0.02)		
		Tolerability	7	443	6	Imipramine	Risk ratio	2.14 (1.08 to 4.22)	τ^2^ = 0 *I* ^2^ = 0%	Global: *P* = 0.77 Local: 0/7
Neurological diseases	Parkinson’s disease, multiple sclerosis, epilepsy, stroke, traumatic brain injury, amputation-related chronic pain	Efficacy	29	1300	15	Trazodone	SMD	−1.33 (−2.06 to −0.61)	τ^2^ = 0.03 *I* ^2^ = 20.4%	Global: *P* = 0.66 Local: 0/21
						Desipramine	SMD	−0.94 (−1.48 to −0.41)		
						Nortriptyline	SMD	−0.74 (−1.12 to −0.36)		
						Paroxetine	SMD	−0.54 (−0.90 to −0.18)		
						Amitriptyline	SMD	−0.50 (−0.84 to −0.17)		
						Citalopram	SMD	−0.47 (−0.75 to −0.19)		
						Fluoxetine	SMD	−0.41 (−0.76 to −0.05)		
		Tolerability	23	1150	14	Citalopram	Risk ratio	2.91 (1.07 to 7.88)	*τ* ^2^ = 0 *I* ^2^=0%	Global: *P* = 0.99 Local: 0/19
						Mirtazapine	Risk ratio	4.97 (1.19 to 20.84)		
Neurocognitive disorders	Alzheimer’s disease, other dementias	Efficacy	9	1126	8	Moclobemide	SMD	−0.55 (−0.70 to −0.40)	τ^2^ = 0 *I* ^2^=0%	Global: *P* = 0.99 Local: 0/19
						Sertraline	SMD	−0.51 (−0.96 to −0.06)		
		Tolerability	7	504	6	None			τ^2^ = 0 *I* ^2^ = 0%	Global: *P* = 0.59 Local: 0/5
Oncological diseases	Breast, colorectal, gynaecological, gastrointestinal, lung cancers	Efficacy	8	602	7	Mianserin	SMD	−0.79 (−1.15 to −0.43)	τ^2^ = 0 *I* ^2^ = 0%	Global: *P* = 0.41 Local: 0/4
		Tolerability	8	602	7	None			τ^2^ = 0.04 *I* ^2^ = 3.8%	Global: *P* = 0.23 Local: 0/5
Respiratory diseases	COPD, asthma	Efficacy	7	396	6	Sertraline	SMD	−1.70 (−2.80 to −0.59)	τ^2^ = 1.18 *I* ^2^ = 92.4%	Not applicable
		Tolerability	4	208	4	None			τ^2^ = 0 *I* ^2^ = 0%	Not applicable

ESM, effect size measure; TCA, tricyclic antidepressant; SMD, standardised mean difference; SSRI, selective serotonin reuptake inhibitor; IHD, ischemic heart disease; PCOS, polycystic ovarian syndrome; HIV, human immunodeficiency virus; AIDS, acquired immunodeficiency syndrome; COPD, chronic obstructive pulmonary disease.

**Fig. 3:**
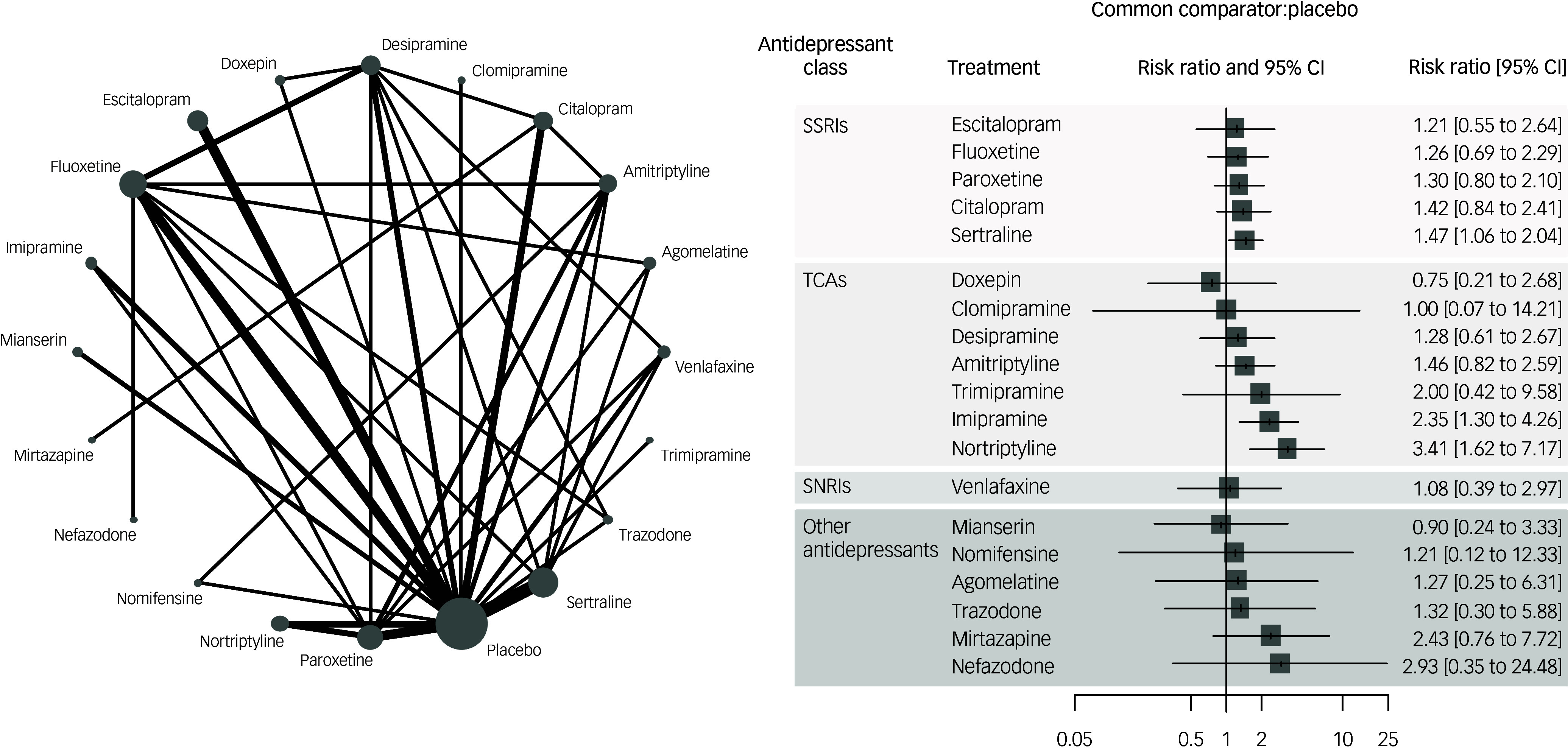
Network map and forest plot for the primary outcome tolerability. SSRI, selective serotonin reuptake inhibitor; TCA, tricyclic antidepressant; SNRI, serotonin and noradrenaline reuptake inhibitor.

In terms of tolerability (individuals discontinuing treatment because of adverse events), placebo outperformed sertraline, imipramine and nortriptyline (Fig. 3; ordered by effect size), with risk ratios ranging from 1.47 (sertraline) to 3.41 (nortriptyline). Head-to-head comparisons showed relatively few statistically significant differences between antidepressants, with amitriptyline, doxepin, paroxetine, sertraline and fluoxetine being more tolerable than nortriptyline (Supplement H, p. 65). *P*-scores showed doxepin as the best-performing treatment, followed by mianserin and venlafaxine.

Overall, the NMA on tolerability showed no heterogeneity (τ^2^ = 0; *I* ^2^ = 0%), and no overall incoherence emerged according to both the global approach (design-by-treatment test, *P* = 0.99) and the local SIDE approach, which did not show inconsistency for any of the 34 comparisons.

Certainty of evidence according to the CINeMA approach was ‘low’ or ‘very low’ for most comparisons, with few exceptions. Notably, certainty was ‘high’ for the comparisons amitriptyline versus paroxetine, sertraline versus placebo, citalopram and paroxetine; and ‘moderate’ for nortriptyline versus placebo, doxepin, venlafaxine, sertraline, paroxetine, citalopram, amitriptyline; amitriptyline versus citalopram, sertraline, placebo; paroxetine and citalopram versus placebo (Supplement H, pp. 70–4).

Sensitivity analyses did not affect heterogeneity or global and local consistency, and effect estimates did not change significantly compared to the primary analysis (Supplement H, pp. 75–9). Meta-regression analyses did not show any possible moderating effect of the analysed variables.

When performing subgroups on different clusters of diseases (Supplement H, pp. 80–5), sertraline was less tolerable than placebo for circulatory system diseases; imipramine was less tolerable than placebo for infectious diseases; citalopram and mirtazapine were less tolerable than placebo for nervous system diseases (Table 2) (Supplement J, pp. 95–101).

**Fig. 4:**
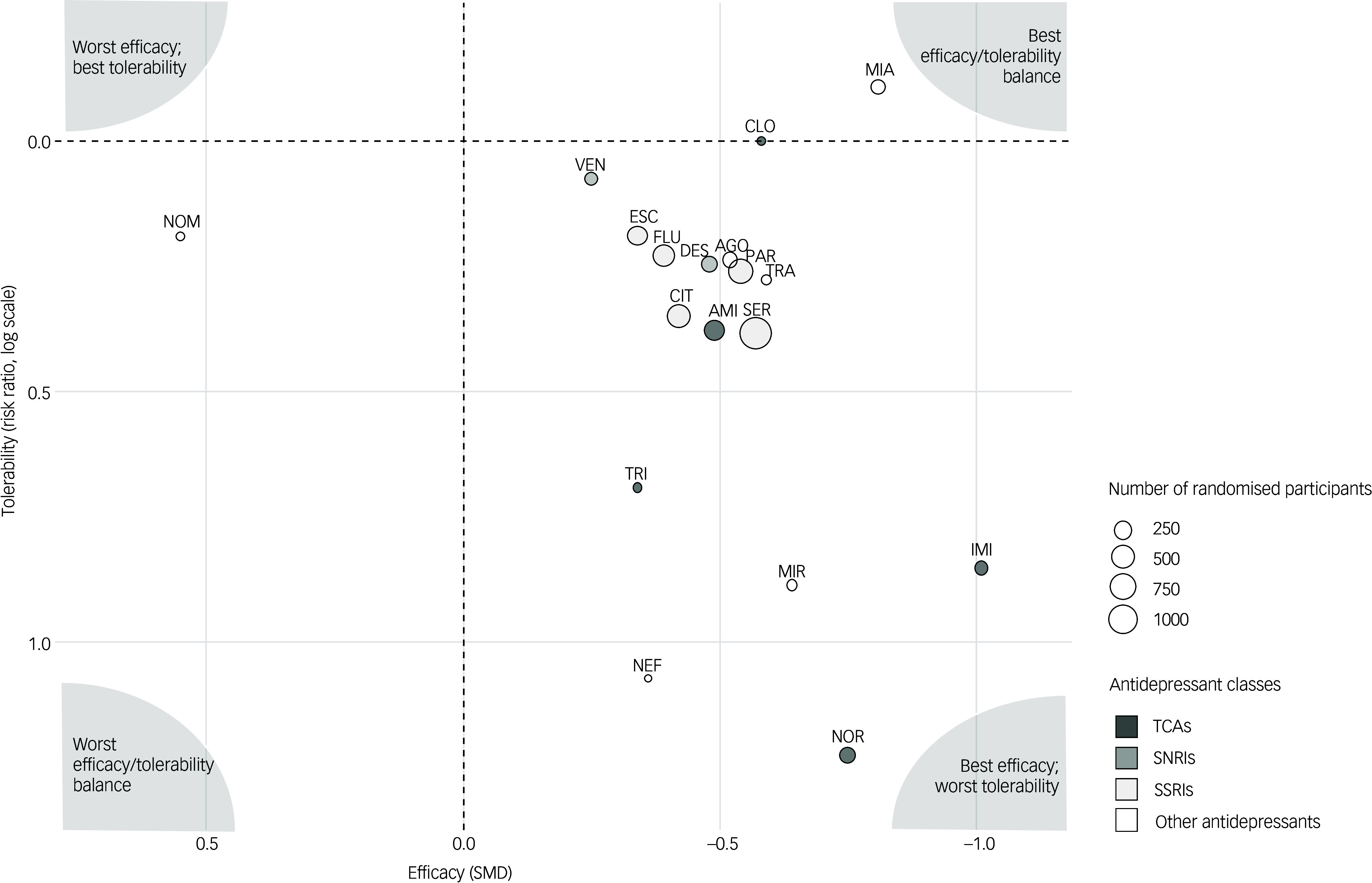
Efficacy/tolerability balance. For each antidepressant, efficacy versus placebo expressed as standardised mean difference is plotted on the *x*-axis (values below 0 indicate better efficacy than placebo), and tolerability versus placebo expressed as risk ratios in logarithm scale are plotted on the *y*-axis (values below 0 indicate better tolerability than placebo). AGO, agomelatine; AMI, amitriptyline; CIT, citalopram; CLO, clomipramine; DES, desipramine; ESC, escitalopram; FLU, fluoxetine; IMI, imipramine; MIA, mianserin; MIR, mirtazapine; NEF, nefazodone; NOM, nomifensine; NOR, nortriptyline; PAR, paroxetine; SER, sertraline; SMD, standardised mean difference; SNRI, serotonin and noradrenaline reuptake inhibitor; SSRI, selective serotonin reuptake inhibitor; TCA, tricyclic antidepressant; TRA, trazodone; TRI, trimipramine; VEN, venlafaxine.

We examined the efficacy/tolerability balance by plotting point estimates of efficacy and tolerability against placebo in a two-dimensional plot (Fig. 4). While SSRIs and SNRIs appeared as relatively consistent groups, being more effective but slightly less tolerable than placebo, for TCAs and other antidepressants results were more heterogeneous, with nortriptyline and imipramine standing out as highly effective but poorly tolerated treatments, and mianserin appearing to be particularly effective and tolerable.

Findings for secondary outcomes are reported in Table 3 and in detail in Supplements K–R (pp. 102–78). In decreasing order of effect size, imipramine, nortriptyline, mianserin, moclobemide, paroxetine, agomelatine, mirtazapine, fluoxetine, citalopram, sertraline and escitalopram were superior to placebo in terms of response. Imipramine, moclobemide, desipramine, mianserin, nortriptyline, paroxetine, citalopram, amitriptyline, fluoxetine, sertraline and escitalopram were superior to placebo in terms of remission. No differences emerged between any antidepressant and placebo in terms of anxiety measured with rating scales, deaths caused by physical conditions and deaths from any cause. Paroxetine outperformed placebo in terms of quality of life; trazodone outperformed placebo in terms of social functioning; mianserin outperformed placebo, and placebo outperformed nortriptyline, in terms of all cause discontinuations; mianserin and citalopram outperformed placebo in terms of discontinuations because of inefficacy. Analyses on anxiety symptoms and social functioning showed substantial heterogeneity; the analysis on quality of life showed moderate heterogeneity, while the remaining analyses did not show relevant heterogeneity. No consistency issues emerged for the secondary analyses (Table 3, Supplements K–R, pp. 102–78).

**Table 3:** Secondary analyses

Outcome	Studies, *N*	Participants, *N*	Treatments, *N*	Significant differences with placebo			Heterogeneity	Inconsistency
				Antidepressant	ESM	Effect size (95% CI)		
Response	90	7612	22	Imipramine	Risk ratio	1.96 (1.44 to 2.66)	τ^2^ = 0.0267 *I* ^2^ = 27.67%	Global: *P* = 0.3 Local: 2/26
				Nortriptyline	Risk ratio	1.86 (1.37 to 2.53)		
				Mianserin	Risk ratio	1.74 (1.37 to 2.53)		
				Moclobemide	Risk ratio	1.59 (1.10 to 2.29)		
				Paroxetine	Risk ratio	1.56 (1.28 to 1.90)		
				Agomelatine	Risk ratio	1.54 (1.15 to 2.06)		
				Mirtazapine	Risk ratio	1.48 (1.07 to 2.04)		
				Fluoxetine	Risk ratio	1.41 (1.19 to 1.68)		
				Citalopram	Risk ratio	1.28 (1.06 to 1.54)		
				Sertraline	Risk ratio	1.26 (1.06 to 1.49)		
				Escitalopram	Risk ratio	1.20 (1.02 to 1.42)		
Remission	78	7246	22	Imipramine	Risk ratio	2.56 (1.72 to 3.81)	τ^2^ = 0.0177 *I* ^2^ = 10.24%	Global: *P* = 0.95 Local: 1/24
				Moclobemide	Risk ratio	2.27 (1.41 to 3.67)		
				Desipramine	Risk ratio	1.96 (1.22 to 3.15)		
				Mianserin	Risk ratio	1.87 (1.13 to 3.09)		
				Nortriptyline	Risk ratio	1.66 (1.09 to 2.52)		
				Paroxetine	Risk ratio	1.48 (1.12 to 1.95)		
				Citalopram	Risk ratio	1.47 (1.15 to 1.88)		
				Amitriptyline	Risk ratio	1.47 (1.03 to 2.09)		
				Fluoxetine	Risk ratio	1.39 (1.10 to 1.76)		
				Sertraline	Risk ratio	1.37 (1.09 to 1.72)		
				Escitalopram	Risk ratio	1.31 (1.02 to 1.68)		
Discontinuations because of inefficacy	55	4542	20	Mianserin	Risk ratio	0.23 (0.07 to 0.77)	τ^2^ = 0 *I* ^2^ = 0%	Global: *P* = 1 Local: 0/28
				Citalopram	Risk ratio	0.38 (0.18 to 0.81)		
Anxiety symptoms (mean change)	22	1313	13	None			*τ* ^2^ = 0.54 *I* ^2^ = 85.6%	Global: *P* = 0.65 Local: 0/13
Quality of life (mean change)	14	707	8	Paroxetine	SMD	0.64 (0.03 to 1.25)	τ^2^ = 0.19 *I* ^2^ = 66.4%	Global: *P* = 0.46 Local: 0/7
Social functioning (mean change)	24	1966	10	Trazodone	SMD	1.13 (0.15 to 2.12)	τ^2^ = 0.50 *I* ^2^ = 89.1%	Global: *P* = 0.19 Local: 0/14
All-cause discontinuation	102	7798	24	Mianserin	Risk ratio	0.42 (0.24 to 0.75)	τ^2^ = 0 *I* ^2^ = 0%	Global: *P* = 0.98 Local: 3/37
				Nortriptyline	Risk ratio	2.83 (1.56 to 5.15)		
Deaths caused by physical condition	82	5851	23	None			τ^2^ = 0 *I* ^2^ = 0%	Global: *P* = 0.68 Local: 0/11
Deaths from any cause	97	7076	23	None			τ^2^ = 0 *I* ^2^ = 0%	Global: *P* = 1 Local: 0/34

ESM, effect size measure; SMD, standardised mean difference.

## Discussion

To our knowledge, this is the largest and most comprehensive systematic review and meta-analysis on the efficacy and tolerability of antidepressants in individuals suffering from depression and physical comorbidities. Overall, we found SSRIs, most of the commonly prescribed TCAs (i.e. imipramine, nortriptyline, amitriptyline and desipramine) and some of the other antidepressants (i.e. mianserin, mirtazapine and agomelatine) to be effective over placebo, although the certainty of evidence according to the CINeMA assessment was ‘low’ or ‘very low’ in most cases, mostly because of relevant within-study bias, imprecision and heterogeneity. Sertraline and paroxetine were effective for the largest number of physical conditions. For other efficacy-related outcomes, such as anxiety symptoms, quality of life, social functioning and inefficacy-related discontinuation, relatively few studies were analysed, providing imprecise results that prevent clear conclusions. In terms of undesirable effects, imipramine, nortriptyline and sertraline were less tolerable than placebo, with ‘low’, ‘moderate’ and ‘high’ certainty, respectively. It is important to note that while the magnitude of sertraline’s effect against placebo was comparable to other SSRIs, the estimate was significantly more precise because of the large number of trials and participants in which it was tested, thereby increasing the certainty of the evidence. As sertraline proved to be effective in many disease-specific subgroup analyses and no relevant differences in tolerability emerged when compared with other SSRIs, we consider that this medication is a reasonable first-line choice in physically ill individuals with depression. After comparing medication classes, SSRIs were more tolerable than TCAs but less tolerable than placebo. Secondary safety outcomes, such as deaths caused by the physical condition and from any cause, did not show differences between antidepressants and placebo.

Compared with the available literature, we found that the benefits of antidepressants in physically ill individuals are overall consistent with those detected in the general population. At the same time, tolerability appeared to be generally worse in the former.^12^ This is particularly evident for some of the most commonly used TCAs, which have up to a 3.4-fold risk of clinically relevant adverse events, but is still relevant for SSRIs, which are generally recommended in people suffering from chronic physical conditions.^17,157,158^ Although most trials involved participants who were older than those recruited in typical antidepressant studies (mean age around 55 years), a meta-regression analysis did not show a moderating effect of age on poorer tolerability of antidepressants.

The results of this NMA should be interpreted in the light of possible limitations. First, we analysed the effect of antidepressants on physically ill individuals, pooling together studies including different physical conditions. This choice might be questionable, as individuals with different diseases might also differ in terms of sociodemographic variables (e.g. distribution of genders) and clinical variables (e.g. level of disability, life expectancy), which can affect the response to antidepressants. Also, depression arising in different physical conditions might be theoretically underpinned by different etiopathological mechanisms (e.g. immunological, inflammatory, endocrinological), which can modulate the response to antidepressants. However, the similar distributions of sociodemographic, clinical and methodological variables across treatments, along with the absence of statistical inconsistency, indicate that the transitivity assumption was upheld in our analyses. In the efficacy analysis, moderate heterogeneity was found, and sensitivity analyses suggest that this was mostly related to the inclusion of placebo-controlled studies, which are generally more likely to deviate from real-world populations, rather than important differences across physical conditions. Further, most heterogeneity likely originated within clusters of studies on the same physical condition, particularly ischemic heart disease, as shown in subgroup analyses. The comparable efficacy of antidepressants across different physical conditions may be explained by their ability to target common underlying pathways, despite differences in the conditions themselves. Second, the quality of included studies was suboptimal because of relatively small sample sizes, bias related to deviations from intended interventions and missing outcome data. Third, although it is recognised that adverse events of antidepressants might differ according to specific vulnerabilities related to each physical condition, we could analyse only generic proxies of tolerability and safety in the overall population, including all conditions together. Further analyses on individual conditions might be useful to detect specific patterns of adverse events, although this aim was beyond the primary objective of our work. Finally, data on quality of life and social functioning were scarce, preventing firm conclusions on such important patient-centred clinical outcomes.

Despite these limitations, the findings of this NMA might have relevant implications for clinical practice, policy and research. According to our results, SSRIs represent a first choice in physically vulnerable populations, with paroxetine and sertraline being investigated in the largest number of physical conditions and being supported by the strongest certainty of evidence. However, a higher vulnerability of comorbid individuals should be recognised also for commonly prescribed antidepressants, such as SSRIs, highlighting the importance of closer clinical monitoring. The use of TCAs remains a highly effective option for the management of depressive symptoms, although, in these populations, the risk of adverse events is arguably higher compared to individuals without comorbid conditions. Thus, this choice should be limited to selected individuals under close clinical monitoring. Other antidepressants, particularly mianserin, mirtazapine and agomelatine, showed relatively large effect sizes in terms of efficacy, although imprecise estimates and overall poor certainty warrant further experimental investigation. Moreover, these medications might effectively target distressing symptoms that are commonly reported in some physical conditions (e.g. insomnia, inappetence), and might have a favourable tolerability profile because of the lack of direct serotonin reuptake inhibition (e.g. lower risk of bleeding and gastrointestinal symptoms). In general, future studies should routinely assess patient-centred outcomes, such as quality of life and social functioning, that are particularly important in these populations.

In conclusion, tailoring treatments to meet individual patient needs, while balancing benefits and risks, poses a significant challenge for practitioners treating individuals with depression and physical comorbidities. Including the best evidence base available within a shared decision-making process is essential to improve the quality of individualised treatments globally.

## Supporting information

De Luca et al. supplementary material De Luca et al. supplementary material

## Data Availability

The database will be made available upon motivated request to the corresponding author, G.O., which will be considered and discussed by the main authors of the study (B.D.L., A.C., C.B., G.V., G.O.).

## Supplementary material

The supplementary material is available online at https://doi.org/10.1192/bjp.2025.18
