# Supplementary material for: Efficacy and tolerability of antidepressants in individuals suffering from physical conditions and depressive disorders: network meta-analysis
Source: Br J Psychiatry. 2025 Aug;227(2):553–66. doi: 10.1192/bjp.2025.18 (PMC12355465; doi:10.1192/bjp.2025.18)
Supplement: De Luca et al. supplementary material [file S0007125025000182sup001.docx]

**Supplementary appendix**

**Index**

|  | PRISMA checklist | p. 2 |
| --- | --- | --- |
|  | **Search strategy** | p. 5 |
|  | **List of studies included/excluded/ongoing/awaiting assessment** | p. 8 |
|  | **Characteristics of included studies** | p. 22 |
|  | **Risk of bias of included studies** | p. 28 |
|  | **Transitivity assessment and meta-regression** | p. 30 |
|  | **Primary outcome: Efficacy (continuous)**   - Pairwise meta-analysis - Network plot - Net league table and forest plot - Global heterogeneity assessment - Consistency assessment (global and local approach) - Assessment of publication bias - CINeMA - Sensitivity analyses - Subgroup analyses by organic pathology | p. 33  p. 34  p. 38  p. 39  p. 41  p. 41  p. 43  p. 44  p. 48  p. 52 |
|  | **Primary outcome: Tolerability (dicothomous)**   - Pairwise meta-analysis - Network plot - Net league table and forest plot - Global heterogeneity assessment - Consistency assessment (global and local approach) - Assessment of publication bias - CINeMA - Sensitivity analyses - Subgroup analyses by organic pathology | p. 60  p. 61  p. 64  p. 65  p. 66  p. 66  p. 68  p. 69  p. 75  p. 79 |
|  | **Secondary analysis: Efficacy by drug classes** | p. 86 |
|  | **Secondary analysis: Tolerability by drug classes** | p. 95 |
|  | **Secondary outcomes:**  - Response  - Remission  - Mean change in anxiety symptoms  - Mean change in quality of life scales  - Mean change in functioning scales  - All-cause discontinuation  - Discontinuations due to inefficacy  - Deaths due to medical condition  - Deaths due to any cause  - Serious adverse events | p. 102  p. 102  p. 112  p. 122  p. 130  p. 136  p. 142  p. 150  p. 157  p. 164  p. 172 |
|  | **Changes to the original protocol** | p. 179 |

**A - PRISMA checklist**

**PRISMA NMA Checklist of Items to Include When Reporting A Systematic Review Involving a Network Meta-analysis**

| **Section/Topic** | **Item #** | **Checklist Item** | **Reported on Page #** |
| --- | --- | --- | --- |
| **TITLE** |  |  |  |
| Title | 1 | Identify the report as a systematic review *incorporating a network meta-analysis (or related form of meta-analysis).* | 1 |
| **ABSTRACT** |  |  |  |
| Structured summary | 2 | Provide a structured summary including, as applicable:  **Background:** main objectives  **Methods:** data sources; study eligibility criteria, participants, and interventions; study appraisal; and *synthesis methods, such as network meta-analysis.*  **Results:** number of studies and participants identified; summary estimates with corresponding confidence/credible intervals; *treatment rankings may also be discussed. Authors may choose to summarize pairwise comparisons against a chosen treatment included in their analyses for brevity.*  **Discussion/Conclusions:** limitations; conclusions and implications of findings.  **Other:** primary source of funding; systematic review registration number with registry name. | 2-3 |
| **INTRODUCTION** |  |  |  |
| Rationale | 3 | Describe the rationale for the review in the context of what is already known*, including mention of why a network meta-analysis has been conducted.* | 4 |
| Objectives | 4 | Provide an explicit statement of questions being addressed, with reference to participants, interventions, comparisons, outcomes, and study design (PICOS). | 4-5 |
| **METHODS** |  |  |  |
| Protocol and registration | 5 | Indicate whether a review protocol exists and if and where it can be accessed (e.g., Web address); and, if available, provide registration information, including registration number. | 5 |
| Eligibility criteria | 6 | Specify study characteristics (e.g., PICOS, length of follow-up) and report characteristics (e.g., years considered, language, publication status) used as criteria for eligibility, giving rationale. *Clearly describe eligible treatments included in the treatment network, and note whether any have been clustered or merged into the same node (with justification).* | 5-6 |
| Information sources | 7 | Describe all information sources (e.g., databases with dates of coverage, contact with study authors to identify additional studies) in the search and date last searched. | 5 |
| Search | 8 | Present full electronic search strategy for at least one database, including any limits used, such that it could be repeated. | 5-6; Suppl. B |
| Study selection | 9 | State the process for selecting studies (i.e., screening, eligibility, included in systematic review, and, if applicable, included in the meta-analysis). | 6-7; Suppl. C |
| Data collection process | 10 | Describe method of data extraction from reports (e.g., piloted forms, independently, in duplicate) and any processes for obtaining and confirming data from investigators. | 6-7 |
| Data items | 11 | List and define all variables for which data were sought (e.g., PICOS, funding sources) and any assumptions and simplifications made. | 5-7 |
| **Geometry of the network** | **S1** | Describe methods used to explore the geometry of the treatment network under study and potential biases related to it. This should include how the evidence base has been graphically summarized for presentation, and what characteristics were compiled and used to describe the evidence base to readers. | 7-8 |
| Risk of bias within individual studies | 12 | Describe methods used for assessing risk of bias of individual studies (including specification of whether this was done at the study or outcome level), and how this information is to be used in any data synthesis. | 6-7 |
| Summary measures | 13 | State the principal summary measures (e.g., risk ratio, difference in means). *Also describe the use of additional summary measures assessed, such as treatment rankings and surface under the cumulative ranking curve (SUCRA) values, as well as modified approaches used to present summary findings from meta-analyses.* | 7-8 |
| Planned methods of analysis | 14 | Describe the methods of handling data and combining results of studies for each network meta-analysis. This should include, but not be limited to:   - *Handling of multi-arm trials;* - *Selection of variance structure;* - *Selection of prior distributions in Bayesian analyses; and* - *Assessment of model fit.* | 7-9 |
| **Assessment of Inconsistency** | **S2** | Describe the statistical methods used to evaluate the agreement of direct and indirect evidence in the treatment network(s) studied. Describe efforts taken to address its presence when found. | 8 |
| Risk of bias across studies | 15 | Specify any assessment of risk of bias that may affect the cumulative evidence (e.g., publication bias, selective reporting within studies). | 9 |
| Additional analyses | 16 | Describe methods of additional analyses if done, indicating which were pre-specified. This may include, but not be limited to, the following:   - Sensitivity or subgroup analyses; - Meta-regression analyses; - *Alternative formulations of the treatment network; and* - *Use of alternative prior distributions for Bayesian analyses (if applicable).* | 9 |
| **RESULTS†** |  |  |  |
| Study selection | 17 | Give numbers of studies screened, assessed for eligibility, and included in the review, with reasons for exclusions at each stage, ideally with a flow diagram. | 9-10 |
| **Presentation of network structure** | **S3** | Provide a network graph of the included studies to enable visualization of the geometry of the treatment network. | 10 |
| **Summary of network geometry** | **S4** | Provide a brief overview of characteristics of the treatment network. This may include commentary on the abundance of trials and randomized subjects for the different interventions and pairwise comparisons in the network, gaps of evidence in the treatment network, and potential biases reflected by the network structure. | 10 |
| Study characteristics | 18 | For each study, present characteristics for which data were extracted (e.g., study size, PICOS, follow-up period) and provide the citations. | 9-10; Suppl. D |
| Risk of bias within studies | 19 | Present data on risk of bias of each study and, if available, any outcome level assessment. | 10; Suppl. E |
| Results of individual studies | 20 | For all outcomes considered (benefits or harms), present, for each study: 1) simple summary data for each intervention group, and 2) effect estimates and confidence intervals. *Modified approaches may be needed to deal with information from larger networks.* | Suppl. D, G, H |
| Synthesis of results | 21 | Present results of each meta-analysis done, including confidence/credible intervals. *In larger networks, authors may focus on comparisons versus a particular comparator (e.g. placebo or standard care), with full findings presented in an appendix. League tables and forest plots may be considered to summarize pairwise comparisons.* If additional summary measures were explored (such as treatment rankings), these should also be presented. | 10-13; Suppl. F, G, H, I, J, K |
| **Exploration for inconsistency** | **S5** | Describe results from investigations of inconsistency. This may include such information as measures of model fit to compare consistency and inconsistency models, *P* values from statistical tests, or summary of inconsistency estimates from different parts of the treatment network. | 10-12 |
| Risk of bias across studies | 22 | Present results of any assessment of risk of bias across studies for the evidence base being studied. | 11-12; Suppl. G, H, I, J, K |
| Results of additional analyses | 23 | Give results of additional analyses, if done (e.g., sensitivity or subgroup analyses, meta-regression analyses*, alternative network geometries studied, alternative choice of prior distributions for Bayesian analyses,* and so forth). | 11-13 |
| **DISCUSSION** |  |  |  |
| Summary of evidence | 24 | Summarize the main findings, including the strength of evidence for each main outcome; consider their relevance to key groups (e.g., healthcare providers, users, and policy-makers). | 13-15 |
| Limitations | 25 | Discuss limitations at study and outcome level (e.g., risk of bias), and at review level (e.g., incomplete retrieval of identified research, reporting bias). *Comment on the validity of the assumptions, such as transitivity and consistency. Comment on any concerns regarding network geometry (e.g., avoidance of certain comparisons).* | 15-16 |
| Conclusions | 26 | Provide a general interpretation of the results in the context of other evidence, and implications for future research. | 16 |
| **FUNDING** |  |  |  |
| Funding | 27 | Describe sources of funding for the systematic review and other support (e.g., supply of data); role of funders for the systematic review. This should also include information regarding whether funding has been received from manufacturers of treatments in the network and/or whether some of the authors are content experts with professional conflicts of interest that could affect use of treatments in the network. | 17 |

**B - Search strategy**

Only results from the year 2000 were considered.

**Pubmed (30/04/2024; n=1281)**

("neuralgia" OR ("myocardial ischemia" OR "coronary artery disease") OR "coronary artery disease" OR "neoplasms" OR "neoplasms" OR "renal insufficiency" OR "rheumatic diseases" OR "immune system diseases" OR "skin and connective tissue diseases" OR "digestive system diseases" OR "respiratory tract diseases" OR "endocrine system diseases" OR "virus diseases" OR "hemic and lymphatic diseases" OR "stroke" OR "nervous system diseases" OR "nutritional and metabolic diseases" OR "occupational diseases") AND ("depressive disorder" OR ("depressive disorder" OR "depression") OR "depress*"[Title/Abstract]) AND ("desipramine"[Title/Abstract] OR "imipramine"[Title/Abstract] OR "clomipramine"[Title/Abstract] OR "opipramol"[Title/Abstract] OR "trimipramine"[Title/Abstract] OR "lofepramine"[Title/Abstract] OR "dibenzepin"[Title/Abstract] OR "amitriptyline"[Title/Abstract] OR "nortriptyline"[Title/Abstract] OR "protriptyline"[Title/Abstract] OR "doxepin"[Title/Abstract] OR "iprindole"[Title/Abstract] OR "melitracen"[Title/Abstract] OR "butriptyline"[Title/Abstract] OR "dosulepin"[Title/Abstract] OR "amoxapine"[Title/Abstract] OR "dimetacrine"[Title/Abstract] OR "amineptine"[Title/Abstract] OR "maprotiline"[Title/Abstract] OR "quinupramine"[Title/Abstract] OR "zimeldine"[Title/Abstract] OR "fluoxetine"[Title/Abstract] OR "citalopram"[Title/Abstract] OR "paroxetine"[Title/Abstract] OR "sertraline"[Title/Abstract] OR "alaproclate"[Title/Abstract] OR "fluvoxamine"[Title/Abstract] OR "etoperidone"[Title/Abstract] OR "escitalopram"[Title/Abstract] OR "isocarboxazid"[Title/Abstract] OR "nialamide"[Title/Abstract] OR "phenelzine"[Title/Abstract] OR "tranylcypromine"[Title/Abstract] OR "iproniazide"[Title/Abstract] OR "iproclozide"[Title/Abstract] OR "moclobemide"[Title/Abstract] OR "toloxatone"[Title/Abstract] OR "oxitriptan"[Title/Abstract] OR "tryptophan"[Title/Abstract] OR "mianserin"[Title/Abstract] OR "nomifensine"[Title/Abstract] OR "trazodone"[Title/Abstract] OR "nefazodone"[Title/Abstract] OR "minaprine"[Title/Abstract] OR "bifemelane"[Title/Abstract] OR "viloxazine"[Title/Abstract] OR "oxaflozane"[Title/Abstract] OR "mirtazapine"[Title/Abstract] OR "bupropion"[Title/Abstract] OR "medifoxamine"[Title/Abstract] OR "tianeptine"[Title/Abstract] OR "pivagabine"[Title/Abstract] OR "venlafaxine"[Title/Abstract] OR "milnacipran"[Title/Abstract] OR "reboxetine"[Title/Abstract] OR "gepirone"[Title/Abstract] OR "duloxetine"[Title/Abstract] OR "agomelatine"[Title/Abstract] OR "desvenlafaxine"[Title/Abstract] OR "vilazodone"[Title/Abstract] OR "vortioxetine"[Title/Abstract] OR "ketamine"[Title/Abstract] OR "esketamine"[Title/Abstract] OR "hyperici herba"[Title/Abstract] OR "hypericum perforatum"[Title/Abstract] OR "st john wort*"[Title/Abstract] OR (("saint s"[All Fields] OR "sainte"[All Fields] OR "saints" OR "saints"[All Fields] OR "saint"[All Fields]) AND "john wort*"[Title/Abstract]) OR "trycyclic*"[Title/Abstract] OR "tca"[Title/Abstract] OR "heterocyclic*"[Title/Abstract] OR "serotonin uptake"[Title/Abstract] OR "ssri*"[Title/Abstract] OR "snri*"[Title/Abstract] OR "monoamine oxidase inhibitor*"[Title/Abstract] OR "maoi*"[Title/Abstract]) AND ("randomized controlled trial"[Title/Abstract] OR "controlled clinical trial"[Title/Abstract] OR "random*"[Title/Abstract] OR "placebo"[Title/Abstract] OR "trial"[Title/Abstract])

**CINHAL** **(30/04/2024; n=21)**

AB ( Myocard* or Coronary or Vascular* or Cardiovascular or Cancer or Tumor or Neoplasm or Lymphoma or Leukemia or Lymphatic or Renal or Rheumatic or Immun* or Connective or Skin or Dermatol* or Digestive or Gastric or Gastroenteric or Gastrointestinal or Enteric or Respiratory or asthma or COPD or Endocrine or Metabolic or dismetabolic or trauma* or Virus or Viral or Neurolog* or Neuralgia or Neuropathic or Stroke or Multiple sclerosis or Parkinson or dementia or medical illness or medically ill pr medical disease or genetic diasease ) AND AB ( depress* or depression or depressive or depressed or mood disorder ) AND AB ( desipramine or imipramine or clomipramine or opipramol or trimipramine or lofepramine or dibenzepin or amitriptyline or nortriptyline or protriptyline or doxepin or iprindole or melitracen or butriptyline or dosulepin or amoxapine or dimetacrine or amineptine or maprotiline or quinupramine or zimeldine or fluoxetine or citalopram or paroxetine or sertraline or alaproclate or fluvoxamine or etoperidone or escitalopram or isocarboxazid or nialamide or phenelzine or tranylcypromine or iproniazide or iproclozide or moclobemide or toloxatone or oxitriptan or tryptophan or mianserin or nomifensine or trazodone or nefazodone or minaprine or bifemelane or viloxazine or oxaflozane or mirtazapine or bupropion or medifoxamine or tianeptine or pivagabine or venlafaxine or milnacipran or etine or gepirone or duloxetine or agomelatine or desvenlafaxine or vilazodone or vortioxetine or ketamine or esketamine or hyperici herba or hypericum perforatum or st john* wort* or saint john* wort* or trycyclic* or TCA* or heterocyclic* or serotonin uptake or SSRI* or SNRI* or monoamine oxidase inhibitor* or MAOI*)

**Psychinfo (30/04/2024; n=707)**

((Myocard* or Coronary or Vascular* or Cardiovascular or Cancer or Tumor or Neoplasm or Lymphoma or Leukemia or Lymphatic or Renal or Rheumatic or Immun* or Connective or Skin or Dermatol* or Digestive or Gastric or Gastroenteric or Gastrointestinal or Enteric or Respiratory or asthma or COPD or Endocrine or Metabolic or dismetabolic or trauma* or Virus or Viral or Neurolog* or Neuralgia or Neuropathic or Stroke or Multiple sclerosis or Parkinson or dementia or medical illness or medically ill pr medical disease or genetic diasease) and depress* and (desipramine or imipramine or clomipramine or opipramol or trimipramine or lofepramine or dibenzepin or amitriptyline or nortriptyline or protriptyline or doxepin or iprindole or melitracen or butriptyline or dosulepin or amoxapine or dimetacrine or amineptine or maprotiline or quinupramine or zimeldine or fluoxetine or citalopram or paroxetine or sertraline or alaproclate or fluvoxamine or etoperidone or escitalopram or isocarboxazid or nialamide or phenelzine or tranylcypromine or iproniazide or iproclozide or moclobemide or toloxatone or oxitriptan or tryptophan or mianserin or nomifensine or trazodone or nefazodone or minaprine or bifemelane or viloxazine or oxaflozane or mirtazapine or bupropion or medifoxamine or tianeptine or pivagabine or venlafaxine or milnacipran or reboxetine or gepirone or duloxetine or agomelatine or desvenlafaxine or vilazodone or vortioxetine or ketamine or esketamine or hyperici herba or hypericum perforatum or st john* wort* or saint john* wort* or trycyclic* or TCA* or heterocyclic* or serotonin uptake or SSRI* or SNRI* or monoamine oxidase inhibitor* or MAOI*) and (randomized controlled trial or controlled clinical trial or random* or placebo or trial)).ab.

**Cochrane Library (CENTRAL) (30/04/2024; n=803)**

#1 MeSH descriptor: [Nervous System Diseases] explode all trees

#2 MeSH descriptor: [Cardiovascular Diseases] explode all trees

#3 MeSH descriptor: [Neoplasms] explode all trees

#4 MeSH descriptor: [Infections] explode all trees

#5 MeSH descriptor: [Musculoskeletal Diseases] explode all trees

#6 MeSH descriptor: [Skin and Connective Tissue Diseases] explode all trees

#7 MeSH descriptor: [Endocrine System Diseases] explode all trees

#8 MeSH descriptor: [Nutritional and Metabolic Diseases] explode all trees

#9 MeSH descriptor: [Respiratory Tract Diseases] explode all trees

#10 MeSH descriptor: [Urogenital Diseases] explode all trees

#11 MeSH descriptor: [Wounds and Injuries] explode all trees

#12 #1 OR #2 OR #3 OR #4 OR #5 OR #6 OR #7 OR #8 OR #9 OR #10 OR #11

#13 (desipramine or imipramine or clomipramine or opipramol or trimipramine or lofepramine or dibenzepin or amitriptyline or nortriptyline or protriptyline or doxepin or iprindole or melitracen or butriptyline or dosulepin or amoxapine or dimetacrine or amineptine or maprotiline or quinupramine or zimeldine or fluoxetine or citalopram or paroxetine or sertraline or alaproclate or fluvoxamine or etoperidone or escitalopram or isocarboxazid or nialamide or phenelzine or tranylcypromine or iproniazide or iproclozide or moclobemide or toloxatone or oxitriptan or tryptophan or mianserin or nomifensine or trazodone or nefazodone or minaprine or bifemelane or viloxazine or oxaflozane or mirtazapine or bupropion or medifoxamine or tianeptine or pivagabine or venlafaxine or milnacipran or reboxetine or gepirone or duloxetine or agomelatine or desvenlafaxine or vilazodone or vortioxetine or ketamine or esketamine or hyperici herba or hypericum perforatum or st john* wort* or saint john* wort* or trycyclic* or TCA* or heterocyclic* or serotonin uptake or SSRI* or SNRI* or monoamine oxidase inhibitor* or MAOI*):ti,ab,kw

#14 (depress*):ti,ab,kw

#15 #12 AND #13 AND #14

**C - List of studies included/excluded/ongoing/awaiting assessment**

**Included studies**

| 1. An H, et al. The Effect of Escitalopram on Mood and Cognition in Depressive Alzheimer’s Disease Subjects. Journal of Alzheimer’s Disease. 2017;55(2):727–735 |
| --- |
| 1. Andersen G, et al. Effective Treatment of Poststroke Depression with the Selective Serotonin Reuptake Inhibitor Citalopram. Stroke. 1994;25(6):1099-104. |
| 1. Andersen J, et al. A controlled trial of the effect of nortriptyline in patients with Parkinson's disease treated with L-DOPA. Acta Neurol Scand. 1980;62(4):210-9. |
| 1. Angermann CE, et al. MOOD-HF Study Investigators and Committee Members. Effect of Escitalopram on All-Cause Mortality and Hospitalization in Patients with Heart Failure and Depression: The MOOD-HF Randomized Clinical Trial. JAMA. 2016;315(24):2683-93. |
| 1. Antonini A, et al. Randomized study of sertraline and low-dose amitriptyline in patients with Parkinson's disease and depression: effect on quality of life. Mov Disord. 2006;21(8):1119-22. |
| 1. Ash G, et al. The effects of dothiepin on subjects with rheumatoid arthritis and depression. Rheumatology (Oxford). 1999;38(10):959-67. |
| 1. Ashman TA, et al. A randomized controlled trial of sertraline for the treatment of depression in persons with traumatic brain injury. Arch Phys Med Rehabil. 2009;90(5):733-40. |
| 1. Avila A, et al. Does nefazodone improve both depression and Parkinson disease? A pilot randomized trial. J Clin Psychopharmacol. 2003;23(5):509-13. |
| 1. Bird H, et al. Paroxetine versus amitriptyline for treatment of depression associated with rheumatoid arthritis: a randomized, double blind, parallel group study. J Rheumatol. 2000;27(12):2791-7. |
| 1. Blumenfield M, et al. Fluoxetine in depressed patients on dialysis. Int J Psychiatry Med. 1997;27(1):71-80. |
| 1. Blumenthal JA, et al. Exercise and pharmacological treatment of depressive symptoms in patients with coronary heart disease: results from the UPBEAT (Understanding the Prognostic Benefits of Exercise and Antidepressant Therapy) study. J Am Coll Cardiol. 2012;60(12):1053-63. |
| 1. Borson S, et al. Improvement in mood, physical symptoms, and function with nortriptyline for depression in patients with chronic obstructive pulmonary disease. Psychosomatics. 1992;33(2):190-201. |
| 1. Brown ES, et al. A Randomized, Double-Blind, Placebo-Controlled Trial of Escitalopram in Patients with Asthma and Major Depressive Disorder. J Allergy Clin Immunol Pract. 2018;6(5):1604-1612. |
| 1. Brown ES, et al. A randomized trial of citalopram versus placebo in outpatients with asthma and major depressive disorder: a proof of concept study. Biol Psychiatry. 2005;58(11):865-70. |
| 1. Brown ES, et al. Escitalopram for severe asthma and major depressive disorder: a randomized, double-blind, placebo-controlled proof-of-concept study. Psychosomatics. 2012;53(1):75-80. |
| 1. Che T, et al. Agomelatine versus fluoxetine in glycemic control and treating depressive and anxiety symptoms in type 2 diabetes mellitus subjects: a single-blind randomized controlled trial. Neuropsychiatr Dis Treat. 2018;14:1527-1533. |
| 1. Costa D, et al. Efficacy and safety of mianserin in the treatment of depression of women with cancer. Acta Psychiatr Scand 1985;320:85-92. |
| 1. Cravello L, et al. The SNRI venlafaxine improves emotional unawareness in patients with post-stroke depression. Hum Psychopharmacol. 2009;24(4):331-6. |
| 1. Dauchy S, et al. A randomized, double-blind, placebo-controlled trial of escitalopram for the treatment of emotional distress during treatment for head and neck cancer. 2014 [EUCTR2008-002159-25-FR] |
| 1. De Carvalho GA, et al. Effects of selective serotonin reuptake inhibitors on thyroid function in depressed patients with primary hypothyroidism or normal thyroid function. Thyroid. 2009;19(7):691-7. |
| 1. De Heer EW, et al. Comparative Effect of Collaborative Care, Pain Medication, and Duloxetine in the Treatment of Major Depressive Disorder and Comorbid (Sub)Chronic Pain: Results of an Exploratory Randomized, Placebo-Controlled, Multicenter Trial (CC:PAINDIP). Front Psychiatry. 2018;9:118. |
| 1. De Vasconcelos Cunha UG, et al. A placebo-controlled double-blind randomized study of venlafaxine in the treatment of depression in dementia. Dement Geriatr Cogn Disord. 2007;24(1):36-41. |
| 1. Devos D, et al. Comparison of desipramine and citalopram treatments for depression in Parkinson's disease: a double-blind, randomized, placebo-controlled study. Mov Disord. 2008;23(6):850-7. |
| 1. Dickens C, et al. The relationship between pain and depression in a trial using paroxetine in sufferers of chronic low back pain. Psychosomatics. 2000;41(6):490-9. |
| 1. Dobkin RD, et al. Depression in Parkinson's disease: symptom improvement and residual symptoms after acute pharmacologic management. Am J Geriatr Psychiatry. 2011;19(3):222-9. |
| 1. Echeverry D, et al. Effect of pharmacological treatment of depression on A1C and quality of life in low-income Hispanics and African Americans with diabetes: a randomized, double-blind, placebo-controlled trial. Diabetes Care. 2009;32(12):2156-60. |
| 1. Ehde DM, et al. Efficacy of paroxetine in treating major depressive disorder in persons with multiple sclerosis. Gen Hosp Psychiatry. 2008;30(1):40-8. |
| 1. Eiser N, et al. Effect of treating depression on quality-of-life and exercise tolerance in severe COPD. COPD. 2005;2(2):233-41. |
| 1. Elliott AJ, et al. Randomized, placebo-controlled trial of paroxetine versus imipramine in depressed HIV-positive outpatients. Am J Psychiatry. 1998;155(3):367-72. |
| 1. Evans M, et al. Placebo-controlled treatment trial of depression in elderly physically ill patients. Int J Geriatr Psychiatry. 1997;12(8):817-24. |
| 1. Fann JR, et al. Venlafaxine extended-release for depression following spinal cord injury: a randomized clinical trial. *JAMA Psychiatry*. 2015;72(3):247-258. |
| 1. Fann JR, et al. Sertraline for Major Depression During the Year Following Traumatic Brain Injury: A Randomized Controlled Trial. J Head Trauma Rehabil. 2017;32(5):332-342. |
| 1. Fraguas R, et al. A double-blind, placebo-controlled treatment trial of citalopram for major depressive disorder in older patients with heart failure: the relevance of the placebo effect and psychological symptoms. Contemp Clin Trials. 2009;30(3):205-11. |
| 1. Friedli K, et al. Sertraline Versus Placebo in Patients with Major Depressive Disorder Undergoing Hemodialysis: A Randomized, Controlled Feasibility Trial. Clin J Am Soc Nephrol. 2017;12(2):280-286. |
| 1. Fruehwald S, et al. Early fluoxetine treatment of post-stroke depression--a three-month double-blind placebo-controlled study with an open-label long-term follow up. J Neurol. 2003;250(3):347-51. |
| 1. Gao J, et al. Different interventions for post-ischaemic stroke depression in different time periods: a single-blind randomized controlled trial with stratification by time after stroke. Clin Rehabil. 2017;31(1):71-81. |
| 1. Glassman AH, et al. Sertraline Antidepressant Heart Attack Randomized Trial (SADHEART) Group. Sertraline treatment of major depression in patients with acute MI or unstable angina. JAMA. 2002;288(6):701-9. |
| 1. Goodkin K, et al. A randomized, double-blind, placebo-controlled trial of trazodone hydrochloride in chronic low back pain syndrome. J Clin Psychopharmacol. 1990;10(4):269-78. |
| 1. Gottlieb SS, et al. A double-blind placebo-controlled pilot study of controlled-release paroxetine on depression and quality of life in chronic heart failure. Am Heart J. 2007;153(5):868-73. |
| 1. Gülseren L, et al. Major Depresif Bozukluğu Olan Diabetes Mellituslu Hastalarda Fluoksetin ve Paroksetinin Depresyon-Anksiyete, Yaşam Kalitesi, Yeti Yitimi ve Metabolik Kontrol Üzerine Etkisi: Tek-Kör, Karşılaştırmalı Bir Çalışma. Klinik Psikofarmakoloji Bülten. 2001;11:1-10. |
| 1. Gülseren L, et al. Comparison of fluoxetine and paroxetine in type II diabetes mellitus patients. Arch Med Res. 2005;36(2):159-65. |
| 1. Hameroff SR, et al. Doxepin's effects on chronic pain and depression: a controlled study. J Clin Psychiatry. 1984;45(3 Pt 2):47-53. |
| 1. He Y, et al. Sertraline hydrochloride treatment for patients with stable chronic obstructive pulmonary disease complicated with depression: a randomized controlled trial. Clin Respir J. 2016;10(3):318-25. |
| 1. Hedayati SS, et al. Effect of Sertraline on Depressive Symptoms in Patients With Chronic Kidney Disease Without Dialysis Dependence: The CAST Randomized Clinical Trial. JAMA. 2017;318(19):1876-1890. |
| 1. Hoare J, et al. Escitalopram treatment of depression in human immunodeficiency virus/acquired immunodeficiency syndrome: a randomized, double-blind, placebo-controlled study. J Nerv Ment Dis. 2014;202(2):133-7. |
| 1. Holland JC, et al. A controlled trial of fluoxetine and desipramine in depressed women with advanced cancer. Psychooncology. 1998;7(4):291-300. |
| 1. Honig A, et al. Treatment of post-myocardial infarction depressive disorder: a randomized, placebo-controlled trial with mirtazapine. Psychosom Med. 2007;69(7):606-13. |
| 1. Kang R, et al. Comparison of paroxetine and agomelatine in depressed type 2 diabetes mellitus patients: a double-blind, randomized, clinical trial. Neuropsychiatr Dis Treat. 2015;11:1307-11. |
| 1. Karaiskos D, et al. Agomelatine and sertraline for the treatment of depression in type 2 diabetes mellitus. Int J Clin Pract. 2013;67(3):257-60. |
| 1. Kennedy S, et al. A double-blind, multicentre, randomised, parallel-group, placebo-controlled study assessing the efficacy and safety of escitalopram in post-myocardial infarction patients suffering from depressive symptoms. 2005. |
| 1. Khazaie H, et al. Treatment of depression in type 2 diabetes with Fluoxetine or Citalopram? Neurosciences (Riyadh). 2011;16(1):42-5. |
| 1. Kim JM, et al. Escitalopram treatment for depressive disorder following acute coronary syndrome: a 24-week double-blind, placebo-controlled trial. J Clin Psychiatry. 2015;76(1):62-8. |
| 1. Kimura M, et al. Treatment of cognitive impairment after poststroke depression : a double-blind treatment trial. Stroke. 2000;31(7):1482-6. |
| 1. Kühn KU, et al. Antidepressive treatment in patients with temporal lobe epilepsy and major depression: a prospective study with three different antidepressants. Epilepsy Behav. 2003;4(6):674-9. |
| 1. Kumar PR, et al. Comparative effect of agomelatine versus escitalopram on glycemic control and symptoms of depression in patients with type 2 diabetes mellitus and depression. IJPSR, 2015;6(10):4304-4309. |
| 1. Lee H, et al. Comparing effects of methylphenidate, sertraline and placebo on neuropsychiatric sequelae in patients with traumatic brain injury. Hum Psychopharmacol. 2005;20(2):97-104. |
| 1. Leentjens AF, et al. SSRIs in the treatment of depression in Parkinson's disease. Int J Geriatr Psychiatry. 2003;18(6):552-4. |
| 1. Lespérance F, et al. Effects of citalopram and interpersonal psychotherapy on depression in patients with coronary artery disease: the Canadian Cardiac Randomized Evaluation of Antidepressant and Psychotherapy Efficacy (CREATE) trial. JAMA. 2007;297(4):367-79. |
| 1. Lipsey JR, et al. Nortriptyline treatment of post-stroke depression: a double-blind study. Lancet. 1984;1(8372):297-300. |
| 1. Lustman PJ, et al. Fluoxetine for depression in diabetes: a randomized double-blind placebo-controlled trial. Diabetes Care. 2000;23(5):618-23. |
| 1. Lyketsos CG, et al. Randomized, placebo-controlled, double-blind clinical trial of sertraline in the treatment of depression complicating Alzheimer's disease: initial results from the Depression in Alzheimer's Disease study. Am J Psychiatry. 2000;157(10):1686-9. |
| 1. Lyketsos CG, et al. Treating depression in Alzheimer disease: efficacy and safety of sertraline therapy, and the benefits of depression reduction: the DIADS. Arch Gen Psychiatry. 2003;60(7):737-46. |
| 1. Macfarlane JG, et al. Trimipramine in rheumatoid arthritis: a randomized double-blind trial in relieving pain and joint tenderness. Curr Med Res Opin. 1986;10(2):89-93. |
| 1. Masoudi M, et al. Effect of sertraline on depression severity and prolactin levels in women with polycystic ovary syndrome: a placebo-controlled randomized trial. Int Clin Psychopharmacol. 2021;36(5):238-243. |
| 1. McFarlane A, et al. Effect of sertraline on the recovery rate of cardiac autonomic function in depressed patients after acute myocardial infarction. Am Heart J. 2001;142(4):617-23. |
| 1. Meghnani Pd, et al. Depression in hospitalized patients of pulmonary tuberculosis and role of anti depressants-a pilot study. Lung India. 1998;6(1):22-25. |
| 1. Miyai I, et al. Effects of Antidepressants on Functional Recovery Following Stroke: A Double-Blind Study. Journal of Neurologic Rehabilitation. 1998;12(1):5-13. |
| 1. Mokhber N, et al. Comparison of sertraline, venlafaxine and desipramine effects on depression, cognition and the daily living activities in Alzheimer patients. Pharmacopsychiatry. 2014;47(4-5):131-40. |
| 1. Munro CA, et al. Depression in Alzheimer’s Disease Study–2 Research Group. Cognitive outcomes after sertaline treatment in patients with depression of Alzheimer disease. Am J Geriatr Psychiatry. 2012;20(12):1036-44. |
| 1. Musselman DL, et al. A double-blind, multicenter, parallel-group study of paroxetine, desipramine, or placebo in breast cancer patients (stages I, II, III, and IV) with major depression. J Clin Psychiatry. 2006;67(2):288-96. |
| 1. NCT00387348. Escitalopram in Treating Depression in Patients With Advanced Lung or Gastrointestinal Cancer. 2012 |
| 1. NCT00621946. The efficacy of citalopram or escitalopram in patients with asthma and major depressive disorder. 2014 |
| 1. Nelson JC, et al. Treatment of major depression with nortriptyline and paroxetine in patients with ischemic heart disease. Am J Psychiatry. 1999;156(7):1024-8. |
| 1. O'Connor CM, et al. Safety and efficacy of sertraline for depression in patients with heart failure: results of the SADHART-CHF (Sertraline Against Depression and Heart Disease in Chronic Heart Failure) trial. J Am Coll Cardiol. 2010;56(9):692-9. |
| 1. Patel S, et al. Escitalopram and mirtazapine for the treatment of depression in hiv patients: a randomized controlled open label trial. ASEAN Journal of Psychiatry. 2014. |
| 1. Peixoto MF, et al. Effects of SSRI medication on heart rate and blood pressure in individuals with hypertension and depression. Clin Exp Hypertens. 2019;41(5):428-433. |
| 1. Petracca G, et al. A double-blind placebo-controlled study of clomipramine in depressed patients with Alzheimer's disease. J Neuropsychiatry Clin Neurosci. 1996;8(3):270-5. |
| 1. Petracca G, et al. A double-blind, placebo-controlled study of fluoxetine in depressed patients with Alzheimer's disease. Int Psychogeriatr. 2001;13(2):233-40. |
| 1. Pezzella G, et al. Treatment of depression in patients with breast cancer: a comparison between paroxetine and amitriptyline. Breast Cancer Res Treat. 2001;70(1):1-10. |
| 1. Pizzi C, et al. Effects of selective serotonin reuptake inhibitor therapy on endothelial function and inflammatory markers in patients with coronary heart disease. Clin Pharmacol Ther. 2009;86(5):527-32. |
| 1. Rabkin JG, et al. Effect of imipramine on mood and enumerative measures of immune status in depressed patients with HIV illness. Am J Psychiatry. 1994;151(4):516-23. |
| 1. Rabkin JG, et al. Fluoxetine treatment for depression in patients with HIV and AIDS: a randomized, placebo-controlled trial. Am J Psychiatry. 1999;156(1):101-7. |
| 1. Rabkin, JG et al. “Testosterone versus fluoxetine for depression and fatigue in HIV/AIDS: a placebo-controlled trial.” *Journal of clinical psychopharmacology* vol. 24,4 (2004): 379-85. |
| 1. Raffaele R, et al. Trazodone therapy of the post-stroke depression. Arch Gerontol Geriatr. 1996;22 Suppl 1:217-20. |
| 1. Rampello L, et al. Evaluation of the prophylactic efficacy of amitriptyline and citalopram, alone or in combination, in patients with comorbidity of depression, migraine, and tension-type headache. Neuropsychobiology. 2004;50(4):322-8. |
| 1. Rampello L, et al. An evaluation of efficacy and safety of reboxetine in elderly patients affected by "retarded" post-stroke depression. A random, placebo-controlled study. Arch Gerontol Geriatr. 2005;40(3):275-85. |
| 1. Rao V; Forest Laboratories. Lexapro for the treatment of traumatic brain injury (TBI) depression & other psychiatric conditions. |
| 1. Razavi D, et al. The effect of fluoxetine on anxiety and depression symptoms in cancer patients. Acta Psychiatr Scand. 1996;94(3):205-10. |
| 1. Richard IH, et al. A randomized, double-blind, placebo-controlled trial of antidepressants in Parkinson disease. Neurology. 2012;78(16):1229-36. |
| 1. Robertson MM, Trimble MR. The treatment of depression in patients with epilepsy. A double-blind trial. J Affect Disord. 1985;9(2):127-36. |
| 1. Robinson LR, et al. Trial of amitriptyline for relief of pain in amputees: results of a randomized controlled study. Arch Phys Med Rehabil. 2004;85(1):1-6. |
| 1. Roose SP, et al. Comparison of paroxetine and nortriptyline in depressed patients with ischemic heart disease. JAMA. 1998;279(4):287-91. |
| 1. Rosenberg PB, et al. Sertraline for the treatment of depression in Alzheimer disease. Am J Geriatr Psychiatry. 2010;18(2):136-45. |
| 1. Roth M, et al. Moclobemide in elderly patients with cognitive decline and depression: an international double-blind, placebo-controlled trial. Br J Psychiatry. 1996;168(2):149-57. |
| 1. Schiffer RB, Wineman NM. Antidepressant pharmacotherapy of depression associated with multiple sclerosis. Am J Psychiatry. 1990;147(11):1493-7. |
| 1. Schwartz JA, McDaniel JS. Double-blind comparison of fluoxetine and desipramine in the treatment of depressed women with advanced HIV disease: a pilot study. Depress Anxiety. 1999;9(2):70-4. |
| 1. Serrano-Dueñas M. A comparison between low doses of amitriptyline and low doses of fluoxetin used in the control of depression in patients suffering from Parkinson's disease. Rev Neurol. 2002 Dec 1-15;35(11):1010-4. |
| 1. Strik JJ, et al. Efficacy and safety of fluoxetine in the treatment of patients with major depression after first myocardial infarction: findings from a double-blind, placebo-controlled trial. Psychosom Med. 2000;62(6):783-9. |
| 1. Tan RS, et al. The effect of low dose lofepramine in depressed elderly patients in general medical wards. Br J Clin Pharmacol. 1994;37(4):321-4. |
| 1. Taraz M, et al. Sertraline decreases serum level of interleukin-6 (IL-6) in hemodialysis patients with depression: results of a randomized double-blind, placebo-controlled clinical trial. Int Immunopharmacol. 2013;17(3):917-23. |
| 1. Targ EF, et al. Structured group therapy and fluoxetine to treat depression in HIV-positive persons. Psychosomatics. 1994;35(2):132-7. |
| 1. Tian X, et al. Effects of paroxetine-mediated inhibition of GRK2 expression on depression and cardiovascular function in patients with myocardial infarction. Neuropsychiatr Dis Treat. 2016;12:2333-2341. |
| 1. Tovilla-Zárate CA, et al. Vortioxetine versus sertraline in metabolic control, distress and depression in Mexican patients with type 2 diabetes. Ann Transl Med. 2019;7(22):656. |
| 1. Van Heeringen K, Zivkov M. Pharmacological treatment of depression in cancer patients. A placebo-controlled study of mianserin. Br J Psychiatry. 1996;169(4):440-3. |
| 1. Ward N, et al. Antidepressants in concomitant chronic back pain and depression: doxepin and desipramine compared. J Clin Psychiatry. 1984;45(3 Pt 2):54-9. |
| 1. Weintraub D, et al. Sertraline for the treatment of depression in Alzheimer disease: week-24 outcomes. Am J Geriatr Psychiatry. 2010;18(4):332-40. |
| 1. Wermuth L et al. Depression in idiopathic Parkinson’s disease treated with citalopram: A placebo-controlled trial. Nordic Journal of Psychiatry. 1998;52(2):163–169. |
| 1. Wiart L, et al. Fluoxetine in early poststroke depression: a double-blind placebo-controlled study. Stroke. 2000;31(8):1829-32. |
| 1. Wohlreich MM, et al. Duloxetine for the treatment of recurrent major depressive disorder in elderly patients: treatment outcomes in patients with comorbid arthritis. Psychosomatics. 2009;50(4):402-12. |
| 1. Wroblewski BA, et al. Antidepressant pharmacotherapy and the treatment of depression in patients with severe traumatic brain injury: a controlled, prospective study. J Clin Psychiatry. 1996;57(12):582-7. |
| 1. Xue H. Paroxetine for Depression in Diabetes: A Randomized Controlled Trial. Chinese Mental Health Journal.2002(12). |
| 1. Yacizi AE, et al. Efficacy and tolerability of escitalopram in depressed patients with end stage renal disease: an open placebo-controlled study. Klinik Psikofarmakoloji Bülteni-Bulletin of Clinical Psychopharmacology. (2012);22(1): 23–30. |
| 1. Zheng AL, et al. Effects of antidepressant therapy in patients with suspected "angina pectoris" and negative coronary angiogram complicating comorbid depression. Zhonghua Xin Xue Guan Bing Za Zhi. 2006;34(12):1097-100. |
| 1. Zisook S, et al. Treatment of major depression in HIV-seropositive men. HIV Neurobehavioral Research Center Group. J Clin Psychiatry. 1998;59(5):217-24. |
| 1. Zöger S, et al. The effects of sertraline on severe tinnitus suffering--a randomized, double-blind, placebo-controlled study. J Clin Psychopharmacol. 2006;26(1):32-9. |

**Excluded studies**

| **Reference** | **Reason for exclusion** |
| --- | --- |
| 1. Alcoff J, et al. Controlled trial of imipramine for chronic low back pain. J Fam Pract. 1982;14(5):841-846. | Not eligible population |
| 1. Allen R, et al. Clinical experience with desvenlafaxine in treatment of pain associated with diabetic peripheral neuropathy. J Pain Res. 2014;7:339-351. Published 2014 Jun 23. | Not eligible population |
| 1. Alves TC, et al. Effects of antidepressant treatment on cognitive performance in elderly subjects with heart failure and comorbid major depression: an exploratory study. Psychosomatics. 2007;48(1):22-30. | Not eligible study design |
| 1. Amsterdam JD, et al. Safety and efficacy of s-citalopram in patients with co-morbid major depression and diabetes mellitus. Neuropsychobiology. 2006;54(4):208-214. | Not eligible study design |
| 1. Angermann CE, et al. Rationale and design of a randomised, controlled, multicenter trial investigating the effects of selective serotonin re-uptake inhibition on morbidity, mortality and mood in depressed heart failure patients (MOOD-HF). Eur J Heart Fail. 2007;9(12):1212-1222. | Not eligible study design |
| 1. Ashman T, et al. Comparison of cognitive behavioral therapy and supportive psychotherapy for the treatment of depression following traumatic brain injury: a randomized controlled trial. J Head Trauma Rehabil. 2014;29(6):467-478. | Not eligible intervention |
| 1. Atkinson JH, et al. A placebo-controlled randomized clinical trial of nortriptyline for chronic low back pain. Pain. 1998;76(3):287-296. | Not eligible population |
| 1. Atkinson JH, et al. Effects of noradrenergic and serotonergic antidepressants on chronic low back pain intensity. Pain. 1999;83(2):137-145. | Not eligible population |
| 1. Baños JH, et al. Impact of early administration of sertraline on cognitive and behavioral recovery in the first year after moderate to severe traumatic brain injury. J Head Trauma Rehabil. 2010;25(5):357-361. | Not eligible population |
| 1. Barone P, et al. Pramipexole versus sertraline in the treatment of depression in Parkinson's disease: a national multicenter parallel-group randomized study. J Neurol. 2006;253(5):601-607. | Not eligible comparison |
| 1. Barragán-Rodríguez L, et al. Efficacy and safety of oral magnesium supplementation in the treatment of depression in the elderly with type 2 diabetes: a randomized, equivalent trial. Magnes Res. 2008;21(4):218-223. | Not eligible comparison |
| 1. Batebi S, et al. A randomized clinical trial of metacognitive therapy and nortriptyline for anxiety, depression, and difficulties in emotion regulation of patients with functional dyspepsia. Res Psychother. 2020;23(2):448. Published 2020 Sep 17. | Not eligible population |
| 1. Beglinger LJ, et al. Randomized controlled trial of atomoxetine for cognitive dysfunction in early Huntington disease. J Clin Psychopharmacol. 2009;29(5):484-487. | Not eligible intervention |
| 1. Beglinger LJ, et al. Results of the citalopram to enhance cognition in Huntington disease trial. Mov Disord. 2014;29(3):401-405. | Not eligible population |
| 1. Blackwell AD, et al. The effects of modafinil on mood and cognition in Huntington's disease. Psychopharmacology (Berl). 2008;199(1):29-36. | Not eligible intervention |
| 1. Boekhout AH, et al. Management of hot flashes in patients who have breast cancer with venlafaxine and clonidine: a randomized, double-blind, placebo-controlled trial. J Clin Oncol. 2011;29(29):3862-3868. | Not eligible population |
| 1. Boggio PS, et al. Effect of repetitive TMS and fluoxetine on cognitive function in patients with Parkinson's disease and concurrent depression. Mov Disord. 2005;20(9):1178-1184. | Not eligible comparison |
| 1. Brown ES, et al. Bupropion in the treatment of outpatients with asthma and major depressive disorder. Int J Psychiatry Med. 2007;37(1):23-28. | Not eligible study design |
| 1. Brusa L, et al. Treatment of the symptoms of Huntington's disease: preliminary results comparing aripiprazole and tetrabenazine. Mov Disord. 2009;24(1):126-129. | Not eligible intervention |
| 1. Cardenas DD, et al. Efficacy of amitriptyline for relief of pain in spinal cord injury: results of a randomized controlled trial. Pain. 2002;96(3):365-373. | Not eligible population |
| 1. Chocron S, et al. Antidepressant therapy in patients undergoing coronary artery bypass grafting: the MOTIV-CABG trial. Ann Thorac Surg. 2013;95(5):1609-1618. | Not eligible population |
| 1. Como PG, et al. A controlled trial of fluoxetine in nondepressed patients with Huntington's disease. Mov Disord. 1997;12(3):397-401. | Not eligible population |
| 1. Cumbo E, et al. Treatment Effects of Vortioxetine on Cognitive Functions in Mild Alzheimer's Disease Patients with Depressive Symptoms: A 12 Month, Open-Label, Observational Study. J Prev Alzheimers Dis. 2019;6(3):192-197. | Not eligible comparison |
| 1. Daghaghzadeh H, et al. Efficacy of duloxetine add on in treatment of inflammatory bowel disease patients: A double-blind controlled study. J Res Med Sci. 2015;20(6):595-601. | Not eligible population |
| 1. Dinan TG, Mobayed M. Treatment resistance of depression after head injury: a preliminary study of amitriptyline response. Acta Psychiatr Scand. 1992;85(4):292-294. | Not eligible study design |
| 1. Duff K, et al. Risperidone and the treatment of psychiatric, motor, and cognitive symptoms in Huntington's disease. Ann Clin Psychiatry. 2008;20(1):1-3. | Not eligible intervention |
| 1. Eija K, Tiina T, J NP. Amitriptyline effectively relieves neuropathic pain following treatment of breast cancer. Pain. 1996;64(2):293-302. | Not eligible population |
| 1. Eisenberg MJ, et al. Bupropion for smoking cessation in patients hospitalized with acute myocardial infarction: a randomized, placebo-controlled trial. J Am Coll Cardiol. 2013;61(5):524-532. | Not eligible population |
| 1. Fann JR, et al. Sertraline in the treatment of major depression following mild traumatic brain injury. J Neuropsychiatry Clin Neurosci. 2000;12(2):226-232. | Not eligible study design |
| 1. Feng B-L, Wang Q-C, Zheng-Yuan LI. Influence of Jieyu Huoxue decoction on rehabilitation of patients with depression after cerebral infarction. Journal of Chinese Integrated Medicine 2004;2(3):182-4. | Not eligible intervention |
| 1. Feng Y, et al. Clinical research of acupuncture on malignant tumor patients for improving depression and sleep quality. J Tradit Chin Med. 2011;31(3):199-202. | Not eligible study design |
| 1. Filipcić I, et al. Depression treatment and its impact upon the quality of life in patients with diabetes type 2 - the Croatian study. Psychiatr Danub. 2010;22(2):231-235. | Not eligible study design |
| 1. Fisch MJ, et al. Fluoxetine versus placebo in advanced cancer outpatients: a double-blinded trial of the Hoosier Oncology Group. J Clin Oncol. 2003;21(10):1937-1943. | Not eligible population |
| 1. Follick MJ, et al. Quality of life post-myocardial infarction: Effects of a transtelephonic coronary intervention system. Health Psychology. 1988;7(2):169–182. | Not eligible intervention |
| 1. Forssell H, et al. Venlafaxine in the treatment of atypical facial pain: a randomized controlled trial. J Orofac Pain. 2004;18(2):131-137. | Not eligible population |
| 1. Fowler PD, et al. Imipramine, rheumatoid arthritis and rheumatoid factor. Curr Med Res Opin. 1977;5(3):241-246. | Not eligible population |
| 1. Fregni F, et al. Repetitive transcranial magnetic stimulation is as effective as fluoxetine in the treatment of depression in patients with Parkinson's disease. J Neurol Neurosurg Psychiatry. 2004;75(8):1171-1174. | Not eligible comparison |
| 1. Fuchs A, et al. Video rating analysis of effect of maprotiline in patients with dementia and depression. Pharmacopsychiatry. 1993;26(2):37-41. | Not eligible population |
| 1. Gao Y, et al. Duloxetine versus placebo in the treatment of patients with diabetic neuropathic pain in China. Chin Med J (Engl). 2010;123(22):3184-3192. | Not eligible population |
| 1. Gao Y, et al. Treatment of patients with diabetic peripheral neuropathic pain in China: a double-blind randomised trial of duloxetine vs. placebo. Int J Clin Pract. 2015;69(9):957-966. | Not eligible population |
| 1. Gaynor PJ, et al. Duloxetine versus placebo in the treatment of major depressive disorder and associated painful physical symptoms: a replication study. Curr Med Res Opin. 2011;27(10):1859-1867. | Not eligible population |
| 1. Gilliam FG, et al. A Trial of Sertraline or Cognitive Behavior Therapy for Depression in Epilepsy. Ann Neurol. 2019;86(4):552-560. | Not eligible comparison |
| 1. Gois C, et al. Treatment response in type 2 diabetes patients with major depression. Clin Psychol Psychother. 2014;21(1):39-48. | Not eligible comparison |
| 1. Goldstein DJ, et al. Duloxetine vs. placebo in patients with painful diabetic neuropathy. Pain. 2005;116(1-2):109-118. | Not eligible population |
| 1. Goodhand JR, et al. Do antidepressants influence the disease course in inflammatory bowel disease? A retrospective case-matched observational study. Inflamm Bowel Dis. 2012;18(7):1232-1239. | Not eligible population |
| 1. Graff-Radford SB, et al. Amitriptyline and fluphenazine in the treatment of postherpetic neuralgia. Clin J Pain. 2000;16(3):188-192. | Not eligible population |
| 1. Guerdjikova AI, et al. Duloxetine in the treatment of binge eating disorder with depressive disorders: a placebo-controlled trial. Int J Eat Disord. 2012;45(2):281-289. | Not eligible population |
| 1. Hammack JE, et al. Phase III evaluation of nortriptyline for alleviation of symptoms of cis-platinum-induced peripheral neuropathy. Pain. 2002;98(1-2):195-203. | Not eligible population |
| 1. Hansen BH, et al. Effects of escitalopram in prevention of depression in patients with acute coronary syndrome (DECARD). J Psychosom Res. 2012;72(1):11-16. | Not eligible population |
| 1. Harrison T, et al. Experience and challenges presented by a multicenter crossover study of combination analgesic therapy for the treatment of painful HIV-associated polyneuropathies. Pain Med. 2013;14(7):1039-1047. | Not eligible population |
| 1. Hart S, et al. Treatment for depression and its relationship to improvement in quality of life and psychological well-being in multiple sclerosis patients. Qual Life Res. 2005;14(3):695-703. | Not eligible comparison |
| 1. He L, et al. Moving cupping at Hechelu combined with rubbing method for depression of diabetes mellitus. Zhongguo Zhen Jiu. 2016;36(3):245-249. | Not eligible comparison |
| 1. Heras P, et al. The role of paroxetine in fatigue and depression of patients under chemotherapeutic treatment. Am J Ther. 2013;20(3):254-256. | Not eligible population |
| 1. Holbech JV, et al. Imipramine and pregabalin combination for painful polyneuropathy: a randomized controlled trial. Pain. 2015;156(5):958-966. | Not eligible population |
| 1. Holl AK, et al. Combating depression in Huntington's disease: effective antidepressive treatment with venlafaxine XR. Int Clin Psychopharmacol. 2010;25(1):46-50. | Not eligible study design |
| 1. Hosseini SH, et al. Citalopram versus psychological training for depression and anxiety symptoms in hemodialysis patients. Iran J Kidney Dis. 2012;6(6):446-451. | Not eligible comparison |
| 1. Hovorka J, et al. Treatment of Interictal Depression with Citalopram in Patients with Epilepsy. Epilepsy Behav. 2000;1(6):444-447. | Not eligible study design |
| 1. Hyer L, et al. Randomized double-blind placebo trial of duloxetine in perioperative spine patients. J Opioid Manag. 2015;11(2):147-155. | Not eligible population |
| 1. Iosifescu DV, et al. The impact of medical comorbidity on acute treatment in major depressive disorder. Am J Psychiatry. 2003;160(12):2122-2127. | Not eligible study design |
| 1. Jacobsen PL, et al. Safety and tolerability of vortioxetine (15 and 20 mg) in patients with major depressive disorder: results of an open-label, flexible-dose, 52-week extension study. Int Clin Psychopharmacol. 2015;30(5):255-264. | Not eligible study design |
| 1. Jain R, et al. A randomized, double-blind, placebo-controlled 6-wk trial of the efficacy and tolerability of 5 mg vortioxetine in adults with major depressive disorder. Int J Neuropsychopharmacol. | Not eligible population |
| 1. Jenkins DG, et al. Tofranil in the treatment of low back pain. J Int Med Res. 1976;4(2 Suppl):28-40. | Not eligible population |
| 1. Jiang W, et al. Effect of escitalopram on mental stress-induced myocardial ischemia: results of the REMIT trial. JAMA. 2013;309(20):2139-2149. | Not eligible population |
| 1. Jorge RE, et al. Sertraline for Preventing Mood Disorders Following Traumatic Brain Injury: A Randomized Clinical Trial. JAMA Psychiatry. 2016;73(10):1041-1047. | Not eligible population |
| 1. Kanner AM, et al. The Use of Sertraline in Patients with Epilepsy: Is It Safe?. Epilepsy Behav. 2000;1(2):100-105. | Not eligible study design |
| 1. Karabacak IY, et al. Treatment effect of sibutramine compared to fluoxetine on leptin levels in polycystic ovary disease. Gynecol Endocrinol. 2004;19(4):196-201. | Not eligible population |
| 1. Kautio AL, et al. Amitriptyline in the treatment of chemotherapy-induced neuropathic symptoms. J Pain Symptom Manage. 2008;35(1):31-39. | Not eligible population |
| 1. Khoromi S, et al. Morphine, nortriptyline and their combination vs. placebo in patients with chronic lumbar root pain. Pain. 2007;130(1-2):66-75. | Not eligible population |
| 1. Kimmick GG, et al. Randomized, double-blind, placebo-controlled, crossover study of sertraline (Zoloft) for the treatment of hot flashes in women with early stage breast cancer taking tamoxifen. Breast J. 2006;12(2):114-122. | Not eligible population |
| 1. Kishore-Kumar R, et al. Desipramine relieves postherpetic neuralgia. Clin Pharmacol Ther. 1990;47(3):305-312. | Not eligible population |
| 1. Komorousova J, et al. Glycemic control improvement through treatment of depression using antidepressant drugs in patients with diabetes mellitus type 1. Neuro Endocrinol Lett. 2010;31(6):801-806. | Not eligible population |
| 1. Koo JR, et al. Treatment of depression and effect of antidepression treatment on nutritional status in chronic hemodialysis patients. Am J Med Sci. 2005;329(1):1-5. | Not eligible intervention |
| 1. Kostić V, et al. “Fluoxetine does not impair motor function in patients with Parkinson's disease: correlation between mood and motor functions with plasma concentrations of fluoxetine/norfluoxetine.” Vojnosanitetski pregled vol. 69,12 (2012): 1067-75. | Not eligible study design |
| 1. Kraus MR, et al. Therapy of interferon-induced depression in chronic hepatitis C with citalopram: a randomised, double-blind, placebo-controlled study. Gut. 2008;57(4):531-536. | Not eligible population |
| 1. Lacasse Y, et al. Randomized trial of paroxetine in end-stage COPD. Monaldi Arch Chest Dis. 2004;61(3):140-147. | Not eligible population |
| 1. Lakshmanan M, et al. Effective low dose tricyclic antidepressant treatment for depressed geriatric rehabilitation patients. A double-blind study. J Am Geriatr Soc. 1986;34(6):421-426. | Not eligible study design |
| 1. Lanctôt KL, et al. Genetic predictors of response to treatment with citalopram in depression secondary to traumatic brain injury. Brain Inj. 2010;24(7-8):959-969. | Not eligible study design |
| 1. Lara-Munoz MDC, et al. La amitriptilina como coanalgésico en pacientes con càncer. Salud Mental. 1990;13(4) | Not eligible population |
| 1. Li HC, et al. Effect of Modified Guipi Decoction on Blood Pressure and Quality of Life in Hypertension Patients Complicated Depression. Zhongguo Zhong Xi Yi Jie He Za Zhi. 2016;36(2):172-178. | Not eligible comparison |
| 1. Liang Z, et al. Agomelatine might be more appropriate for elderly, depressed, type 2 diabetes mellitus patients than paroxetine/fluoxetine. Aging (Albany NY). 2021;13(19):22934-22946. | Not eligible study design |
| 1. Liebowitz MR, et al. A double-blind, randomized, placebo-controlled study assessing the efficacy and tolerability of desvenlafaxine 10 and 50 mg/day in adult outpatients with major depressive disorder. BMC Psychiatry. 2013;13:94. Published 2013 Mar 22. | Not eligible population |
| 1. Light RW, et al. Doxepin treatment of depressed patients with chronic obstructive pulmonary disease. Arch Intern Med. 1986;146(7):1377-1380. | Not eligible study design |
| 1. Lustman PJ, et al. Effects of nortriptyline on depression and glycemic control in diabetes: results of a double-blind, placebo-controlled trial. Psychosom Med. 1997;59(3):241-250. | Not eligible population |
| 1. Lydiatt WM, et al. A randomized, placebo-controlled trial of citalopram for the prevention of major depression during treatment for head and neck cancer. Arch Otolaryngol Head Neck Surg. 2008;134(5):528-535. | Not eligible population |
| 1. Lydiatt WM, et al. Prevention of depression with escitalopram in patients undergoing treatment for head and neck cancer: randomized, double-blind, placebo-controlled clinical trial. JAMA Otolaryngol Head Neck Surg. 2013;139(7):678-686. | Not eligible population |
| 1. Mahableshwarkar AR, et al. A randomized, double-blind, duloxetine-referenced study comparing efficacy and tolerability of 2 fixed doses of vortioxetine in the acute treatment of adults with MDD. Psychopharmacology (Berl). 2015;232(12):2061-2070. | Not eligible population |
| 1. Max MB, et al. Amitriptyline relieves diabetic neuropathy pain in patients with normal or depressed mood. Neurology. 1987;37(4):589-596. | Not eligible population |
| 1. Max MB, et al. Efficacy of desipramine in painful diabetic neuropathy: a placebo-controlled trial. Pain. 1991;45(1):3-9. | Not eligible population |
| 1. McIntyre RS et al. Effect of metabolic syndrome and thyroid hormone on efficacy of desvenlafaxine 50 and 100 mg/d in major depressive disorder. Curr Med Res Opin. 2016;32(3):587-599. | Not eligible study design |
| 1. Mehrotra R, et al. Comparative Efficacy of Therapies for Treatment of Depression for Patients Undergoing Maintenance Hemodialysis: A Randomized Clinical Trial. Ann Intern Med. 2019;170(6):369-379. | Not eligible comparison |
| 1. Menza M, et al. The impact of treatment of depression on quality of life, disability and relapse in patients with Parkinson's disease. Mov Disord. 2009;24(9):1325-1332. | Not eligible population |
| 1. Mikocka-Walus A, et al. Adjuvant therapy with antidepressants for the management of inflammatory bowel disease. Cochrane Database Syst Rev. 2019;4(4):CD012680. Published 2019 Apr 12. | Not eligible population |
| 1. Mohr DC, et al. Brain lesion volume and neuropsychological function predict efficacy of treatment for depression in multiple sclerosis. J Consult Clin Psychol. 2003;71(6):1017-1024. | Not eligible comparison |
| 1. Mohr DC, et al. Comparative outcomes for individual cognitive-behavior therapy, supportive-expressive group psychotherapy, and sertraline for the treatment of depression in multiple sclerosis. J Consult Clin Psychol. 2001;69(6):942-949. | Not eligible comparison |
| 1. Mohr DC, et al. Effects of treatment for depression on fatigue in multiple sclerosis. Psychosom Med. 2003;65(4):542-547. | Not eligible comparison |
| 1. Mohr DC, et al. The relationship between social support, depression and treatment for depression in people with multiple sclerosis. Psychol Med. 2004;34(3):533-541. | Not eligible comparison |
| 1. Morrow GR, et al. Differential effects of paroxetine on fatigue and depression: a randomized, double-blind trial from the University of Rochester Cancer Center Community Clinical Oncology Program. J Clin Oncol. 2003;21(24):4635-4641. | Not eligible population |
| 1. Mulvahill JS, et al. Effect of Metabolic Syndrome on Late-Life Depression: Associations with Disease Severity and Treatment Resistance. Journal of the American Geriatrics Society. 2017;65(12):2651-2658. | Not eligible study design |
| 1. Murray V, et al. Double-blind comparison of sertraline and placebo in stroke patients with minor depression and less severe major depression. J Clin Psychiatry. 2005;66(6):708-716. | Not eligible population |
| 1. Musselman D, et al. The impact of escitalopram on IL-2-induced neuroendocrine, immune, and behavioral changes in patients with malignant melanoma: preliminary findings. Neuropsychopharmacology. 2013;38(10):1921-1928. | Not eligible population |
| 1. Musselman DL, et al. Paroxetine for the prevention of depression induced by high-dose interferon alfa. N Engl J Med. 2001;344(13):961-966. | Not eligible population |
| 1. Navan P, et al. Randomized, double-blind, 3-month parallel study of the effects of pramipexole, pergolide, and placebo on Parkinsonian tremor. Mov Disord. 2003;18(11):1324-1331. | Not eligible population |
| 1. Navari RM, et al. Treatment of depressive symptoms in patients with early stage breast cancer undergoing adjuvant therapy. Breast Cancer Res Treat. 2008;112(1):197-201. | Not eligible population |
| 1. NCT00026637 | Not eligible comparison |
| 1. NCT01777581 | Not eligible population |
| 1. NCT02358343 | Not eligible comparison |
| 1. NCT03464383 | Not eligible comparison |
| 1. NCT05047952 | Not eligible population |
| 1. Newburn G, et al. Moclobemide in the treatment of major depressive disorder (DSM-3) following traumatic brain injury. Brain Inj. 1999;13(8):637-642. | Not eligible study design |
| 1. Nicolau J, et al. Treatment of depression in type 2 diabetic patients: effects on depressive symptoms, quality of life and metabolic control. Diabetes Res Clin Pract. 2013;101(2):148-152. | Not eligible study design |
| 1. Novack TA, et al. Impact of early administration of sertraline on depressive symptoms in the first year after traumatic brain injury. J Neurotrauma. 2009;26(11):1921-1928. | Not eligible population |
| 1. Nuñez GR, et al. Bupropion for control of hot flashes in breast cancer survivors: a prospective, double-blind, randomized, crossover, pilot phase II trial. J Pain Symptom Manage. 2013;45(6):969-979. | Not eligible population |
| 1. Ondo WG, et al. Memantine for non-motor features of Parkinson's disease: a double-blind placebo controlled exploratory pilot trial. Parkinsonism Relat Disord. 2011;17(3):156-159. | Not eligible population |
| 1. Orjuela-Rojas JM, et al. Treatment of depression in patients with temporal lobe epilepsy: A pilot study of cognitive behavioral therapy vs. selective serotonin reuptake inhibitors. Epilepsy Behav. 2015;51:176-181. | Not eligible comparison |
| 1. Pae CU, et al. Paroxetine in the treatment of depressed patients with haematological malignancy: an open-label study. Hum Psychopharmacol Clin Exp. 2004;19:25-29. | Not eligible study design |
| 1. Paile-Hyvärinen M, et al. Quality of life and metabolic status in mildly depressed patients with type 2 diabetes treated with paroxetine: a double-blind randomised placebo controlled 6-month trial. BMC Fam Pract. 2007;8:34. Published 2007 Jun 15. | Not eligible population |
| 1. Paile-Hyvärinen M, et al. Quality of life and metabolic status in mildly depressed women with type 2 diabetes treated with paroxetine: a single-blind randomised placebo controlled trial. BMC Fam Pract. 2003;4:7. | Not eligible population |
| 1. Paleacu D, et al. Olanzapine in Huntington's disease. Acta Neurol Scand. 2002;105(6):441-444. | Not eligible intervention |
| 1. Pan XL, et al. Cognitive Function Recovery in Patients with Non-Depressed Ischemic Stroke: An Open Randomized Controlled Study. Brain Impairment. 2018; 29(3): 228-234. | Not eligible population |
| 1. Parker JC, et al. Management of depression in rheumatoid arthritis: a combined pharmacologic and cognitive-behavioral approach. Arthritis Rheum. 2003;49(6):766-777. | Not eligible comparison |
| 1. Perino C, et al. Mood and behavioural disorders following traumatic brain injury: clinical evaluation and pharmacological management. Brain Inj. 2001;15(2):139-148. | Not eligible study design |
| 1. Petrak F, et al. Cognitive Behavioral Therapy Versus Sertraline in Patients With Depression and Poorly Controlled Diabetes: The Diabetes and Depression (DAD) Study: A Randomized Controlled Multicenter Trial. Diabetes Care. 2015;38(5):767-775. | Not eligible comparison |
| 1. Pheasant H, et al. Amitriptyline and chronic low back pain. A randomized double-blind crossover study. Spine. 1983;8(5):552-557 | Not eligible population |
| 1. Raja SN, et al. Opioids versus antidepressants in postherpetic neuralgia: a randomized, placebo-controlled trial. Neurology. 2002;59(7):1015-1021. | Not eligible population |
| 1. Rapoport MJ, et al. A randomized controlled trial of antidepressant continuation for major depression following traumatic brain injury. J Clin Psychiatry. 2010;71(9):1125-1130. | Not eligible study design |
| 1. Rapoport MJ, et al. An open-label study of citalopram for major depression following traumatic brain injury. J Psychopharmacol. 2008;22(8):860-864. | Not eligible study design |
| 1. Raskin J, et al. A double-blind, randomized multicenter trial comparing duloxetine with placebo in the management of diabetic peripheral neuropathic pain. Pain Med. 2005;6(5):346-356. | Not eligible population |
| 1. Reding MJ, et al. Antidepressant therapy after stroke. Arch Neurol. 1986;43:763-765 | Not eligible population |
| 1. Reifler BV, et al. Double-blind trial of imipramine in Alzheimer's disease patients with and without depression. Am J Psychiatry. 1989;146(1):45-49. | Not eligible population |
| 1. Rektorová I, et al. Pramipexole and pergolide in the treatment of depression in Parkinson's disease: a national multicentre prospective randomized study. Eur J Neurol. 2003;10(4):399-406. | Not eligible population |
| 1. Rigotti NA, et al. Bupropion for smokers hospitalized with acute cardiovascular disease. Am J Med. 2006;119(12):1080-1087. | Not eligible population |
| 1. Rios Romenets S, et al. Doxepin and cognitive behavioural therapy for insomnia in patients with Parkinson's disease -- a randomized study. Parkinsonism Relat Disord. 2013;19(7):670-675. | Not eligible population |
| 1. Ripley DL, et al. Atomoxetine for attention deficits following traumatic brain injury: results from a randomized controlled trial. Brain Inj. 2014;28(12):1514-1522. | Not eligible population |
| 1. Robinson RG, et al. Nortriptyline versus fluoxetine in the treatment of depression and in short-term recovery after stroke: a placebo-controlled, double-blind study. Am J Psychiatry. 2000;157(3):351-359. | Not eligible population |
| 1. Roscoe JA, et al. Effect of paroxetine hydrochloride (Paxil) on fatigue and depression in breast cancer patients receiving chemotherapy. Breast Cancer Res Treat. 2005;89(3):243-249. | Not eligible population |
| 1. Rowbotham MC, et al. Venlafaxine extended release in the treatment of painful diabetic neuropathy: a double-blind, placebo-controlled study [published correction appears in Pain. 2005 Jan;113(1-2):248]. Pain. 2004;110(3):697-706. | Not eligible population |
| 1. Sarzi Puttini P, et al. A comparison of dothiepin versus placebo in the treatment of pain in rheumatoid arthritis and the association of pain with depression. J Int Med Res. 1988;16(5):331-337. | Not eligible population |
| 1. Schukro RP, et al. Efficacy of duloxetine in chronic low back pain with a neuropathic component: a randomized, double-blind, placebo-controlled crossover trial. Anesthesiology 2016;124:150–8. | Not eligible population |
| 1. Semenchuk MR, et al. Double-blind, randomized trial of bupropion SR for the treatment of neuropathic pain. Neurology. 2001;57(9):1583-1588. | Not eligible population |
| 1. Sheikh JI, et al. Efficacy, safety, and tolerability of sertraline in patients with late-life depression and comorbid medical illness [published correction appears in J Am Geriatr Soc. 2004 Jul;52(7):1228]. J Am Geriatr Soc. 2004;52(1):86-92. | Not eligible population |
| 1. Simpson DA. Gabapentin and venlafaxine for the treatment of painful diabetic neuropathy. J Clin Neuromuscul Dis 2001;3:53–62. | Not eligible population |
| 1. Smith EM, et al. Alliance for Clinical Trials in Oncology. Effect of duloxetine on pain, function, and quality of life among patients with chemotherapy-induced painful peripheral neuropathy: a randomized clinical trial. JAMA 2013;309:1359–67. | Not eligible population |
| 1. Spaans HP, et al. Vascular risk factors in older patients with depression: outcome of electroconvulsive therapy versus medication. International journal of geriatric psychiatry. 2018;33(2):371-378. | Not eligible population |
| 1. Specchio LM, et al. Citalopram as treatment of depression in patients with epilepsy. Clin Neuropharmacol. 2004;27(3):133-136. | Not eligible study design |
| 1. Squitieri F, et al. Short-term effects of olanzapine in Huntington disease. Neuropsychiatry Neuropsychol Behav Neurol. 2001;14(1):69-72. | Not eligible intervention |
| 1. Sullivan M, et al. A randomized trial of nortriptyline for severe chronic tinnitus. Effects on depression, disability, and tinnitus symptoms. Arch Intern Med. 1993;153(19):2251-2259. | Not eligible population |
| 1. Takahashi M, et al. Antidepressants for Depression, Apathy, and Gait Instability in Parkinson's Disease: A Multicenter Randomized Study. Intern Med. 2019;58(3):361-368. | Not eligible population |
| 1. Tammiala-Salonen T, Forssell H. Trazodone in burning mouth pain: a placebo-controlled, double-blind study. J Orofac Pain 1999;13:83–8. | Not eligible population |
| 1. Tasmuth T, et al. Venlafaxine in neuropathic pain following treatment of breast cancer. Eur J Pain 2002;6:17–24. | Not eligible population |
| 1. Thomé-Souza MS, et al. Sertraline and fluoxetine: safe treatments for children and adolescents with epilepsy and depression. Epilepsy Behav. 2007;10(3):417-425. | Not eligible population |
| 1. Torta R et al. Duloxetine for the treatment of mood disorder in cancer patients: a 12-week case-control clinical trial. Hum Psychopharmacol. 2011;26(4-5):291-299. | Not eligible study design |
| 1. Tsai AC, et al. Directly observed antidepressant medication treatment and HIV outcomes among homeless and marginally housed HIV-positive adults: a randomized controlled trial.American journal of public health. 2013; 103(2): 308-15. | Not eligible population |
| 1. Turner-Stokes L, et al. Managing depression in brain injury rehabilitation: the use of an integrated care pathway and preliminary report of response to sertraline. Clin Rehabil. 2002;16(3):261-268. | Not eligible study design |
| 1. Vollmer TL, et al. A randomized, double-blind, placebo-controlled trial of duloxetine for the treatment of pain in patients with multiple sclerosis. Pain Pract 2014;14:732–44. | Not eligible population |
| 1. Volpe FM. An 8-week, open-label trial of duloxetine for comorbid major depressive disorder and chronic headache. J Clin Psychiatry. 2008;69(9):1449-1454 | Not eligible study design |
| 1. Vranken JH, et al. Duloxetine in patients with central neuropathic pain caused by spinal cord injury or stroke: a randomized, double-blind, placebo-controlled trial. PAIN 2011;152:267–73. | Not eligible population |
| 1. Vrethem M, et al. A comparison of amitriptyline and maprotiline in the treatment of painful polyneuropathy in diabetics and nondiabetics. Clin J Pain 1997;13:313–23. | Not eligible population |
| 1. Weintraub D, et al. Atomoxetine for depression and other neuropsychiatric symptoms in Parkinson disease. Neurology. 2010;75(5):448-455. | Not eligible population |
| 1. Werneck AL, et al. The use of an antagonist 5-HT2a/c for depression and motor function in Parkinson' disease. Arq Neuropsiquiatr. 2009;67(2B):407-412. | Not eligible population |
| 1. Wernicke JF, et al. A randomized controlled trial of duloxetine in diabetic peripheral neuropathic pain. Neurology 2006;67:1411–20. | Not eligible population |
| 1. Yang J, et al. Controlled study on antidepressant treatment of patients with post-stroke depression. Chinise Mental Health Journal. 2002;16(12):871-872 | Not eligible population |
| 1. Yasuda H, et al. Superiority of duloxetine to placebo in improving diabetic neuropathic pain: results of a randomized controlled trial in Japan. J Diabetes Investig 2011;2:132–9. | Not eligible population |
| 1. Yi Z, et al. Clinical observation of flupentixol and melitracen combined with specific immunotherapy for treatment of allergic rhinitis in patients with anxiety and depression. J Clin Otorhinolaryngol Head Neck Surg. 2017;31(1):34-42 | Not eligible population |
| 1. Yu YY, et al. Efficacy and safety of esomeprazole with flupentixol/melitracen in treating gastroesophageal reflux disease patients with emotional disorders. J Gastroenterol Hepatol. 2014;29(6):1200-1206. | Not eligible population |
| 1. Zeng W, et al. The influence of antidepressive therapy on short-term prognosis in elderly patients with unstable angina and depression. Chin J Intern Med. 2001;40(12):809-810 | Not eligible comparison |
| 1. Zhang M, et al. Clinical study of duloxetine hydrochloride combined with doxazosin for the treatment of pain disorder in chronic prostatitis/chronic pelvic pain syndrome: An observational study. Medicine (Baltimore). 2017;96(10):e6243. | Not eligible population |
| 1. Zhang WT, Wang YF. Efficacy of methylphenidate for the treatment of mental sequelae after traumatic brain injury. Medicine (Baltimore). 2017;96(25):e6960. | Not eligible intervention |
| 1. Zheng AL, et al. Effects of antidepressant therapy in patients with suspected "angina pectoris" and negative coronary angiogram complicating comorbid depression. Chin J Cardiol. 2006;34(12):1097-1100. |  |
| 1. Zhou Y, et al. Ketamine Alleviates Depressive Symptoms in Patients Undergoing Intracranial Tumor Resection: A Randomized Controlled Trial. Anesth Analg. 2021;133(6):1588-1597. | Not eligible intervention |

**Ongoing studies**

| **Reference** | **Notes** |
| --- | --- |
| 1. NCT00009191 | Recruitment Status: Completed. No results posted. |
| 1. NCT00229333 | Recruitment Status: Unknown. No results posted. |
| 1. NCT00595699 | Recruitment Status: Completed. No results posted. |
| 1. NCT01153165 | Recruitment Status: Unknown. No results posted. |
| 1. NCT01644916 | Recruitment Status: Completed. No results posted. |
| 1. NCT02238977 | Insufficient data. Study stopped due to difficulty recruiting. |
| 1. NCT02463110 | Recruitment Status: Completed. No results posted. |
| 1. NCT02813447 | Recruitment Status: Completed. No results posted. |
| 1. NCT02845349 | Recruitment Status: Withdrawn. No results posted. |
| 1. NCT03652870 | Recruitment Status: Completed. No results posted. |
| 1. NCT03996265 | Recruitment Status: Recruiting. No results posted. |
| 1. NCT04162743 | Recruitment Status: Completed. No results posted. |
| 1. NCT04422652 | Recruitment Status: Recruiting. No results posted. |
| 1. NCT04944017 | Recruitment Status: Recruiting. No results posted. |
| 1. NCT05004987 | Recruitment Status: Recruiting. No results posted. |

**Studies awaiting assessment**

| **Reference** | **Notes** |
| --- | --- |
| 1. Ansari A, et al. Role of sertraline in posttraumatic brain injury depression and quality-of-life in TBI. Asian J Neurosurg. 2014;9(4):182-188. | Awaiting assessement |
| 1. Ece Çetin, Fatma et al. “Effıcacy of cıtalopram on stroke recurrence: A randomızed clınıcal trıal.” Journal of clinical neuroscience : official journal of the Neurosurgical Society of Australasia vol. 101 (2022): 168-174. | Awaiting assessement |
| 1. Duan CC. Clinical study of sertraline hydrochloride combined with mesalazine in the treatment of ulcerative colitis patients with depression. Chronic Pathematol J 2016; 17: 560–562. | Not available after online research |
| 1. Feng XQ, He HD. Study on the application of sertraline hydrochloride in the treatment of ulcerative colitis patients with depression. Ningxia Med J 2017; 39: 20170605. | Not available after online research |
| 1. Jia L, et al. Influences of escitalopram on cognitive function and cardiac function in elderly patients with chronic heart failure complicated with depression disorder. Chongqing Yixue. 2017;46:2652-2654 | Not available after online research |
| 1. Jia W. Effect of early intervention on recovery of motor function and recurrent stroke in patients with post-stroke depression. Chinese Journal of Clinical Rehabilitation 2005;9(12):4-5. | Not available after online research |
| 1. Li Z. Clinical efficacy of paroxetine in the treatment of post-stroke depression. Modern Journal of Integrated Traditional Chinese and Western Medicine 2007;16(34):5103-4. | Not available after online research |
| 1. Liu JD, Zheng H. Efficacy of fluoxetine in the treatment of depression in patients with acute myocardial infarction. Journal of Clinical Psychological Medicine 1999;9:210-1. | Not available after online research |
| 1. Marui L, et al. Efficacy of antidepressive treatment in affective disorders associated to ischemic vascular disease. Neurologia 1988;3 Suppl 3:10. | Not available after online research |
| 1. Mauri MC, et al. A double blind study on fluvoxamine vs. placebo in depressed HIV positive patients: short-term and perspective results. Integrative Psychiatry 1994;10:199-201. | Not available after online research |
| 1. Meara RJ, et al. The treatment of depression after stroke with the selective serotonin reuptake inhibitor sertraline. Cerebrovascular Diseases 1998;8 Suppl 4:90. | Not available after online research |
| 1. Munro CA, et al. Cognitive response to pharmacological treatment for depression in Alzheimer disease: secondary outcomes from the depression in Alzheimer's disease study (DIADS). Am J Geriatr Psychiatry. 2004;12(5):491-498. | Not available after online research |
| 1. Pogosova GV, et al. Clinical efficiency of tianeptine in patients with ischemic heart disease and comorbid depression. Kardiologiia. 2004;3:20-24 | Not available after online research |
| 1. Xie R, Liu J, Quan H. A prospective random clinical contrast study of treatment with sertraline in elderly patients with post-stroke depression. Chinese Journal of Clinical Neuroscience 2005;13(3):294-7. | Not available after online research |
| 1. Yang J, et al. Controlled study on antidepressant treatment of patients with post-stroke depression. Chinese Mental Health Journal 2002;16(12):871-2. | Not available after online research |
| 1. Zhu S, et al. Short-term efficacy of venlafaxine treating the depression in epilepsy patients. Chinese Journal of Rehabilitation. 2004;19(2):101. | Not available after online research |

**D – Characteristics of included studies**

| **First author** | **Year** | **Number of patients** | **Treatment 1 (mean dose)** | **Treatment 2 (mean dose)** | **Treatment 3 (mean dose)** | **Women (%)** | **Mean age (SD)** | **Mean baseline severity** | **Blinding** | **Sponsor by industry** | **Setting** | **Country** | **Mean follow-up (weeks)** |
| --- | --- | --- | --- | --- | --- | --- | --- | --- | --- | --- | --- | --- | --- |
| An | 2017 | 84 | Escitalopram 5-15mg | Placebo |  | 0,79 | 75,1 (7,1) |  | Yes | Yes | Outpatient | South Korea | 12 |
| Andersen | 1980 | 19 | Nortriptyline 25-150mg | Placebo |  | 0,33 | 59 |  | Yes | Yes | Outpatient | Denmark | 8 |
| Andersen | 1994 | 66 | Citalopram 10-20mg | Placebo |  | 0,56 | 67 (10,65) | 19,2 | Yes | Yes | Mixed | Denmark | 6 |
| Angermann | 2016 | 372 | Escitalopram 15,8mg | Placebo |  | 0,24 | 62,3 (11,9) | 16,5 | Yes | No | Outpatient | Germany | 96 |
| Antonini | 2006 | 31 | Amitriptyline 25mg | Sertraline 50mg |  | 0,55 | 70,2 (6,6) | 20 | Yes | Yes | Outpatient | Italy | 12 |
| Ash | 1999 | 48 | Dothiepin 75-150mg | Placebo |  |  | 60 (9,3) | 15,5 | Yes | Yes | Outpatient | US | 12 |
| Ashman | 2009 | 52 | Sertraline 25-200mg | Placebo |  | 0,46 | 49,6 (10,5) | 25,9 | Yes | No | Outpatient | US | 10 |
| Avila | 2003 | 16 | Fluoxetine 25mg | Nefazodone 200mg |  | 0,56 | 70,4 (6,4) | 18,7 | Yes | No | Outpatient | Spain | 12 |
| Bird | 2000 | 188 | Amitriptyline 75-150mg | Paroxetine 20-40mg |  | 0,72 | 54,7 (9,2) | 19 | Yes | No | Outpatient | Multicenter (EU) | 8 |
| Blumenfield | 1997 | 14 | Fluoxetine 20mg | Placebo |  |  |  |  | Yes | Yes | Outpatient | US | 8 |
| Blumenthal | 2012 | 101 | Sertraline 50-200mg | Placebo |  | 0,25 | 63,4 (10,7) | 14,4 | Yes | Yes | Outpatient | US | 16 |
| Borson | 1992 | 36 | Notriptyline (serum level) | Placebo |  |  | 60,9 (9,1) | 29,5 | Yes | No | Outpatient | US | 12 |
| Brown | 2005 | 82 | Citalopram 20-60mg | Placebo |  | 0,8 | 41,6 (10,5) | 23,7 | Yes | No | Outpatient | US | 12 |
| Brown | 2012 | 25 | Escitalopram 10-20mg | Placebo |  | 0,56 | 45,6 (11,3) | 26,5 | Yes | No | Outpatient | US | 12 |
| Brown | 2018 | 99 | Escitalopram 10-20mg | Placebo |  | 0,57 | 45,7 (11,41) | 24,9 | Yes | No | Outpatient | US | 12 |
| Che | 2018 | 84 | Agomelatine 39,32 | Fluoxetine 34,5mg |  | 0,57 | 52,2 (10,6) | 23,8 | Yes | No | Outpatient | China | 12 |
| Costa | 1985 | 73 | Mianserin 10-20mg | Placebo |  | 1 | 51,5 (10,4) | 20,7 | Yes | No | Mixed | Romania | 4 |
| Cravello | 2009 | 50 | Fluoxetine 34,4mg | Venlafaxine 132mg |  | 0,6 | 65 (13,4) | 17,9 | No | No | Inpatient | Italy | 8 |
| Dauchy | 2014 | 38 | Escitalopram 10mg | Placebo |  |  |  |  | Yes | No |  | France | 12 |
| De Carvalho | 2009 | 28 | Fluoxetine >20mg | Sertraline >50mg |  | 0,92 | 42,9 (12) | 23,3 | No | No | Outpatient | Brazil | 12 |
| De Heer | 2018 | 41 | Duloxetine 60-90mg | Placebo |  | 0,61 | 41,5 (21,6) |  | Yes | Yes | Outpatient | Germany | 12 |
| De Vasconcelos | 2007 | 31 | Venlafaxine 37,5-131,25 | Placebo |  | 0,74 | 77,6 (6,5) | 19 | Yes | No | Outpatient | Brasil | 6 |
| Devos | 2008 | 48 | Citalopram 20mg | Desipramine 75mg | Placebo | 0,55 | 62 (10,2) | 21 | Yes | No | Outpatient | France | 4 |
| Dickens | 2000 | 92 | Paroxetine 20mg | Placebo |  | 0,55 | 45 (10,2) | 21 | Yes | No | Outpatient | England | 8 |
| Dobkin | 2011 | 52 | Nortriptyline 48,5mg | Paroxetine 28,4mg | Placebo | 0,48 | 62,1 (8,7) | 19,7 | Yes | No | Outpatient | US | 8 |
| Echeverry | 2009 | 89 | Sertraline 50-100mg | Placebo |  | 0,73 | 52,5 (9) | 17,5 | Yes | No | Outpatient | US | 26 |
| Ehde | 2008 | 42 | Paroxetine 10-40mg | Placebo |  | 0,48 |  | 18,1 | Yes | No | Outpatient | US | 12 |
| Eiser | 2005 | 28 | Paroxetine 20-40mg | Placebo |  |  |  | 17,5 | Yes | No | Outpatient | England | 6 |
| Elliot | 1998 | 75 | Imipramine 162,5mg | Paroxetine 33,9mg | Placebo |  |  | 21 | Yes | Yes | Outpatient | US | 12 |
| Evans | 1997 | 82 | Fluoxetine 20mg | Placebo |  | 0,51 | 80,4 (6,6) | 20,8 | Yes | Yes | Outpatient | England | 8 |
| Fann | 2015 | 133 | Venlafaxine 186mg | Placebo |  | 0,26 | 40,1 (11,6) | 19,5 | Yes | No | Mixed | US | 12 |
| Fann | 2017 | 31 | Sertraline 25-200mg | Placebo |  | 0,25 | 37,5 (12,7) | 22,9 | Yes | No | Inpatient | US | 12 |
| Fraguas | 2009 | 37 | Citalopram 20-40mg | Placebo |  | 0,51 | 75,5 (5,3) | 16,2 | Yes | No | Outpatient | Brazil | 8 |
| Friedli | 2017 | 30 | Sertraline 50-150mg | Placebo |  | 0,24 | 59,1 (13,8) | 19 | Yes | No | Outpatient | England | 24 |
| Fruehwald | 2003 | 54 | Fluoxetine 20mg | Placebo |  | 0,41 | 64,4 (14,1) | 31,6 | Yes | No | Inpatient | Austria | 12 |
| Gao | 2017 | 182 | Citalopram 20mg | Placebo |  | 0,48 | 66,6 (8,5) | 17,2 | Yes | No | Mixed | China | 12 |
| Glassman | 2002 | 369 | Sertraline 50-200mg | Placebo |  | 0,37 | 57,2 (10,7) | 19,6 | Yes | Yes | Outpatient | Multicenter | 24 |
| Goodkin | 1990 | 42 | Trazodone 201mg | Placebo |  | 0,38 | 53,7 (12,9) | 13,5 | Yes | No | Outpatient | US | 6 |
| Gottlieb | 2007 | 28 | Paroxetine 25mg | Placebo |  | 0,14 | 62 (10,7) | 15,5 | Yes | No | Inpatient | US | 12 |
| Gulseren | 2001 | 21 | Fluoxetine 26,1mg | Paroxetine 20mg |  | 0,8 | 55,5 (13,4) | 17,4 | Yes | No | Outpatient | Turkey | 12 |
| Gulseren | 2004 | 23 | Fluoxetine 20mg | Paroxetine 26,1mg |  | 0,86 | 57,8 (11,4) | 18,1 | Yes | No | Outpatient | Turkey | 12 |
| Hameroff | 1985 | 60 | Doxepin 50-300mg | Placebo |  |  |  |  | Yes |  |  | US | 6 |
| He | 2014 | 120 | Sertraline 50mg | Placebo |  | 0,44 | 70 (7) | 24,8 | Yes | No | Outpatient | China | 6 |
| Hedayati | 2017 | 201 | Sertraline 50-200mg | Placebo |  | 0,27 | 58,4 (13,5) | 18 | Yes | No |  | US | 12 |
| Hoare | 2014 | 105 | Escitalopram 10mg | Placebo |  | 0,88 | 34 | 24 | Yes | Yes |  | South Africa | 6 |
| Holland | 1998 | 38 | Desipramine 25-150mg | Fluoxetine 20-40mg |  |  | 50,3 (9,5) | 23,2 | Yes | No | Outpatient | US | 8 |
| Honig | 2007 | 91 | Mirtazapine 15-45mg | Placebo |  | 0,16 | 57,3 (10,4) | 17,7 | Yes | No | Inpatient | Netherlands | 24 |
| Kang | 2015 | 116 | Agomelatine 30-50mg | Paroxetine 20-40mg |  | 0,46 | 51,7 (10,9) | 24,1 | Yes | No | Outpatient | China | 12 |
| Karaiskos | 2013 | 40 | Agomelatine 31mg | Sertraline 75mg |  |  | 53,3 (12) | 20,5 | No | No | Outpatient | Greece | 16 |
| Kennedy | 2005 | 19 | Escitalopram 10-20mg | Placebo |  | 0,53 | 57,5 |  | Yes | No |  | Multicenter | 24 |
| Khazaie | 2011 | 40 | Citalopram 20-40mg | Fluoxetine 20-40mg |  | 0,4 | 49,7 (8,5) | 19,3 | Yes | No | Outpatient | Iran | 12 |
| Kim | 2015 | 217 | Escitalopram 5-20mg | Placebo |  | 0,4 | 59,3 (10,7) | 15,5 | Yes | Yes | Inpatient | Korea | 24 |
| Kimura | 2000 | 47 | Nortriptyline 100mg | Placebo |  | 0,42 | 60,2 (10,5) | 17,7 | Yes | No | Inpatient | US | 9 |
| Kuhn | 2003 | 75 | Citalopram | Mirtazapine | Reboxetine | 0,6 | 40,1 (10,7) | 20,2 | Yes | No | Outpatient | Germany | 4 |
| Kumar | 2015 | 42 | Escitalopram 10mg | Agomelatine 25mg |  |  | 49,1 (12,2) | 17,8 | No | No | Outpatient | India | 8 |
| Lee | 2005 | 20 | Sertraline 100mg | Placebo |  | 0,25 | 34,6 (9,7) | 21 | Yes | No | Inpatient | Korea | 4 |
| Leentjens | 2003 | 12 | Sertraline 50-100mg | Placebo |  | 0,33 | 67 (7,8) | 15,5 | Yes | Yes | Outpatient | Netherlands | 10 |
| Lesperance | 2007 | 284 | Citalopram 20-40mg | Placebo |  | 0,29 | 58,1 (9,2) | 21,5 | Yes | No | Outpatient | Canada | 12 |
| Lipsey | 1984 | 39 | Nortriptyline 100mg | Placebo |  | 0,35 | 61 (23,8) | 15,3 | Yes | No | Mixed | US | 6 |
| Lustman | 2000 | 60 | Fluoxetine 20-40mg | Placebo |  | 0,7 | 46,3 (12,3) | 19,8 | Yes | Yes | Outpatient | US | 8 |
| Lyketsos | 2003 | 44 | Sertraline 108mg | Placebo |  | 0,68 | 77,5 (7,5) | 22,8 | Yes | No | Outpatient | US | 12 |
| Lyketsos | 2000 | 22 | Sertraline 81mg | Placebo |  | 0,59 | 77 (8,4) | 23,2 | Yes | No | Outpatient | US | 12 |
| MacFarlane | 1986 | 36 | Trimipramine 25-75mg | Placebo |  | 0,75 | 59,1 (12,2) |  | Yes | No |  | Canada | 12 |
| Masoudi | 2021 | 74 | Sertraline 50mg | Placebo |  | 1 | 32,5 (6,9) | 15,8 | Yes | No | Outpatient | Iran | 6 |
| McFarlane | 2001 | 38 | Sertraline 50mg | Placebo |  | 0,4 | 56 (11,5) |  | Yes | No | Inpatient | Canada | 22 |
| Meghnani | 1988 | 59 | Imipramine 75mg | Placebo |  |  |  |  | Yes | No | Inpatient | India | 4 |
| Miyai | 1998 | 24 | Desipramine | Fluoxetine | Trazodone | 0,25 | 74 (9) | 22,6 | Yes | No | Inpatient | US | 4 |
| Mokhber | 2014 | 59 | Desipramine 25-150mg | Sertraline 25-150mg | Venlafaxine 37,5-150mg | 0,42 | 67,6 (4) | 23 | Yes | No | Outpatient | Iran | 12 |
| Munro | 2012 | 131 | Sertraline 25-125mg | Placebo |  | 0,54 | 77,3 (8) |  | Yes | No | Outpatient | US | 24 |
| Musselman | 2006 | 35 | Desipramine 113mg | Paroxetine 31mg | Placebo | 1 | 54,5 (11,3) | 19,8 | Yes | Yes | Outpatient | US | 24 |
| NCT00387348 | 2012 | 24 | Escitalopram 10mg | Placebo |  | 0,59 | 58 (9,9) |  | Yes | No |  | US | 4 |
| NCT00621946 | 2014 | 26 | Escitalopram 10-20mg | Placebo |  | 0,56 | 45,5 (11,3) | 26,5 | Yes | Yes |  | US | 12 |
| Nelson | 1999 | 81 | Nortriptyline 73mg | Paroxetine 22mg |  | 0,17 | 57,9 (11,8) | 22,6 | Yes | Yes | Outpatient | US | 6 |
| O’Connor | 2010 | 469 | Sertraline 50mg | Placebo |  | 0,41 | 62,2 (10,8) | 18,3 | Yes | Yes |  | US | 12 |
| Patel | 2013 | 70 | Escitalopram 7,5-20mg | Mirtazapine 5-30mg |  | 0,47 | 37,3 (8,0) | 37 | No | No |  | India | 8 |
| Peixoto | 2019 | 30 | Escitalopram 20mg | Placebo |  | 0,77 | 55,6 (6,9) | 26,3 | Yes | No | Outpatient | Brazil | 8 |
| Petracca | 1996 | 24 | Clomipramine 100mg | Placebo |  | 0,91 | 72 (7,2) | 17,5 | Yes | No | Outpatient | US | 6 |
| Petracca | 2001 | 41 | Fluoxetine 40mg | Placebo |  | 0,61 | 70,8 (6,7) | 16,6 | Yes | No | Oupatient | Argentina | 6 |
| Pezzella | 2001 | 179 | Amitriptyline 75-150mg | Paroxetine 20-40mg |  | 1 | 51,5 |  | Yes | No | Outpatient | Italy | 8 |
| Pizzi | 2009 | 100 | Sertraline 70mg | Placebo |  | 0,51 | 56,8 (8,5) | 17,5 | Yes | No | Outpatient | Italy | 20 |
| Rabkin | 1994 | 97 | Imipramine 200-300mg | Placebo |  | 0,05 |  | 16 | Yes | No | Outpatient | US | 6 |
| Rabkin | 1999 | 120 | Fluoxetine 20-40mg | Placebo |  | 0,02 | 39 (9) | 17,5 | Yes | No |  | US | 8 |
| Rabkin | 2004 | 85 | Fluoxetine 34,4mg | Placebo |  | 0 | 40,5 (8,9) | 16,5 | Yes | No | Outpatient | US | 8 |
| Raffaele | 1996 | 22 | Trazodone 300mg | Placebo |  | 0,41 | 70 (2,7) |  | Yes | No |  | Italy | 6 |
| Rampello | 2005 | 31 | Reboxetine 8mg | Placebo |  | 0,55 | 77,4 (3,8) | 24 | Yes | No | Outpatient | Italy | 16 |
| Rampello | 2004 | 88 | Amitriptyline 50mg | Citalopram 20mg |  | 0,63 | 39,9 (65,9) | 28,2 | No | No |  | Italy | 16 |
| Rao | 2020 | 14 | Escitalopram 10-20mg | Placebo |  | 0,38 |  | 24,5 | Yes | No |  | US | 12 |
| Razavi | 1996 | 91 | Fluoxetine 20 mg | Placebo |  | 0,80 | 52,9 (11,3) | 19,5 | Yes | Yes |  | France | 5 |
| Richard | 2012 | 115 | Paroxetine 24mg | Venlafaxine 121mg | Placebo | 0,37 | 63,6 (10,7) | 21,6 | Yes | No |  | Multicenter | 12 |
| Robertson | 1985 | 39 | Amitriptyline 25mg | Nomifesine 25mg | Placebo | 0,67 | 36,1 (11,2) | 18,5 | Yes | No | Outpatient | UK | 6 |
| Robinson | 2004 | 39 | Amitriptyline 25-125mg | Placebo |  | 0,13 | 44,9 (11,4) |  | Yes | No | Outpatient |  | 6 |
| Roose | 1998 | 81 | Nortriptyline 74mg | Paroxetine 22mg |  | 0,17 | 58 (12) | 23 | Yes | No | Outpatient | US | 6 |
| Rosemberg | 2010 | 131 | Sertraline 100mg | Placebo |  | 0,54 | 77,3 (8) |  | Yes | Yes |  | US | 12 |
| Roth | 1996 | 726 | Moclobenide 400mg | Placebo |  | 0,77 | 73,6 (8,4) | 24,2 | Yes | No | Mixed | Multicenter | 6 |
| Schiffer | 1990 | 28 | Imipramine 123mg | Placebo |  | 0,83 | 38,4 | 26,6 | Yes | No | Outpatient | US | 6 |
| Schwartz | 1999 | 14 | Desipramine 75-100mg | Fluoxetine 20-40mg |  | 1 | 35,7 | 18,7 | Yes | Yes | Outpatient | US | 6 |
| Serrano-Dueno | 2002 | 77 | Amitriptyline 35mg | Fluoxetine 27mg |  | 0,44 | 68,2 (4,6) | 43,1 | No | No |  | Ecuador | 48 |
| Strik | 2000 | 54 | Fluoxetine 47,3mg | Placebo |  | 0,3 | 56,4 (10,9) | 21,6 | Yes | Yes | Outpatient | Netherlands | 25 |
| Tan | 1994 | 63 | Lofepramine 70mg | Placebo |  | 0,66 | 80 (7) | 13,5 | Yes | Yes | Inpatient | UK | 4 |
| Taraz | 2013 | 50 | Sertraline 50-100mg | Placebo |  | 0,42 | 62,5 (23,3) | 17,5 | Yes | No | Outpatient | Iran | 12 |
| Targ | 1994 | 20 | Fluoxetine 20mg | Placebo |  | 0 | 33 (33,7) | 20,3 | Yes | No | Outpatient | US | 12 |
| Tian | 2016 | 46 | Fluoxetine 10-20mg | Paroxetine 10-20mg |  | 0,46 | 62,5 (10,5) | 27,3 | Yes | No | Inpatient | China | 8 |
| Tovilla-Zarate | 2019 | 50 | Sertraline 75mg | Vortioxetine 10mg |  | 0,71 | 48,5 (11) | 25,8 | Yes | No | Outpatient | Mexico | 8 |
| Van Heeringen | 1996 | 55 | Mianserin 60mg | Placebo |  | 1 | 52 (8) | 18,7 | Yes | Yes |  | Belgium | 6 |
| Ward | 1984 | 36 | Desipramine 173mg | Doxepin 188mg |  | 0,54 | 42,5 | 26,4 | Yes | No | Inpatient | US | 4 |
| Weintraub | 2010 | 131 | Sertraline 100mg |  |  | 0,54 | 77,3 (8) |  | Yes | No | Outpatient | US | 24 |
| Wermuth | 1998 | 37 | Citalopram 10-20mg | Placebo |  | 0,57 | 64,7 | 16,4 | Yes | Yes | Outpatient | Denmark | 52 |
| Wiart | 2000 | 31 | Fluoxetine 20mg | Placebo |  | 0,52 | 67,6 (9,4) | 21,8 | Yes | Yes | Inpatient | France | 6,4 |
| Wohlreich | 2009 | 172 | Duloxetine 60mg | Placebo |  | 0,59 | 72,9 (5,7) | 18,8 | Yes | Yes | Outpatient | US | 6 |
| Wroblewski | 1996 | 10 | Desipramine 150mg | Placebo |  | 0,3 | 32,1 |  | Yes | Yes | Outpatient | US | 4 |
| Xue | 2004 | 53 | Paroxetine 20-40mg | Placebo |  | 0,58 | 47,3 (12,2) | 18 | Yes | No |  | China | 8 |
| Yazici | 2011 | 62 | Escitalopram 10-20mg | Placebo |  |  | 51 (11) | 29 | Yes | No | Outpatient | Turkey | 8 |
| Zheng | 2006 | 81 | Fluoxetine 20mg | Placebo |  |  |  | 27,8 | Yes | No |  | China | 4 |
| Zisook | 1998 | 47 | Fluoxetine 20-60mg | Placebo |  | 0 | 35,5 (7,6) | 20,3 | Yes | No |  | US | 7 |
| Zoger | 2006 | 76 | Sertraline | Placebo |  | 0,43 | 43 (12,5) | 19 | Yes | No | Outpatient | Sweden | 16 |

**E - Risk of bias of included studies**

*Outcome: efficacy*


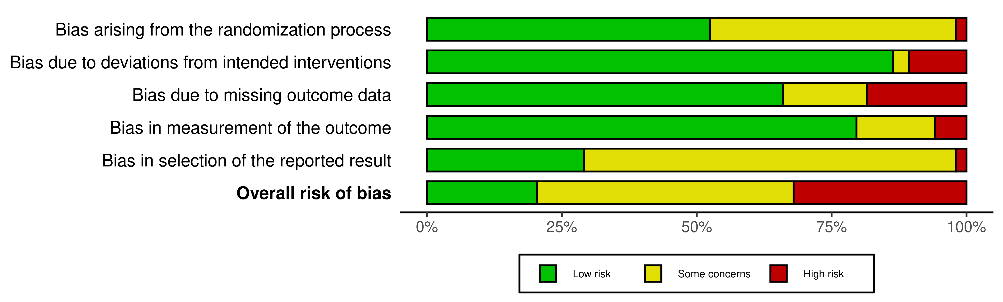

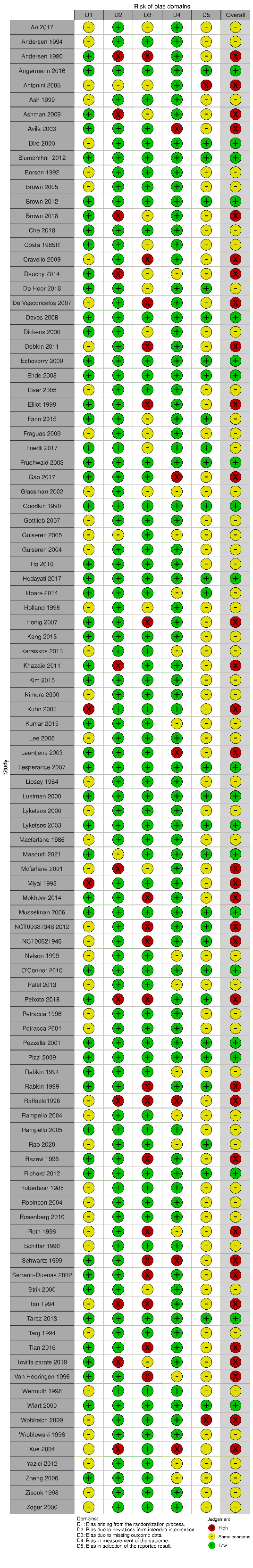


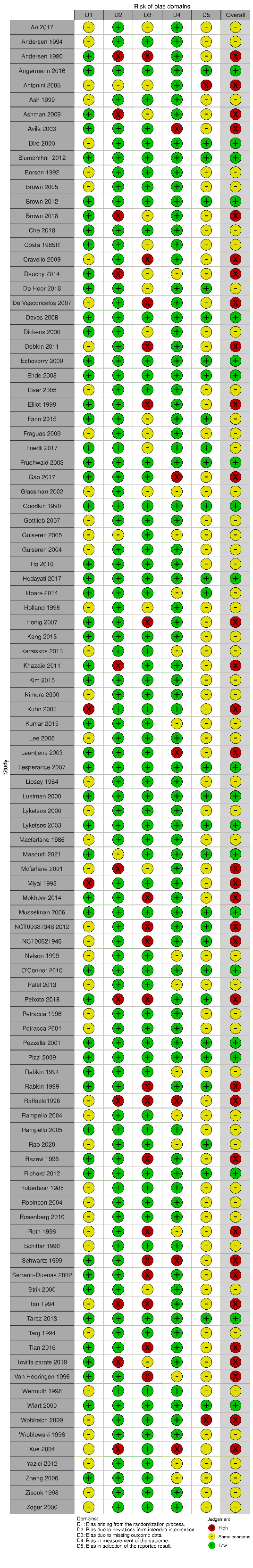


*Outcome: tolerability*


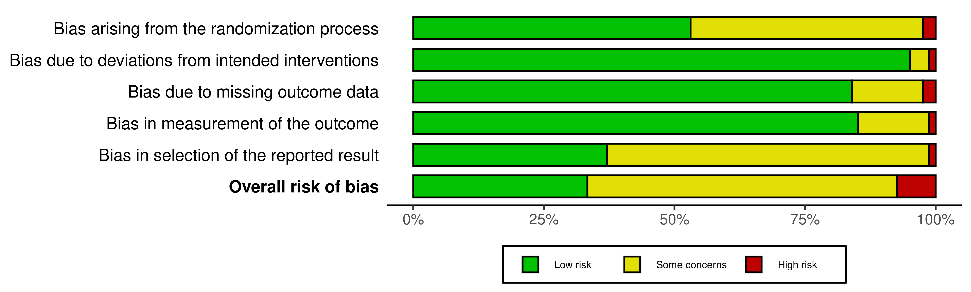

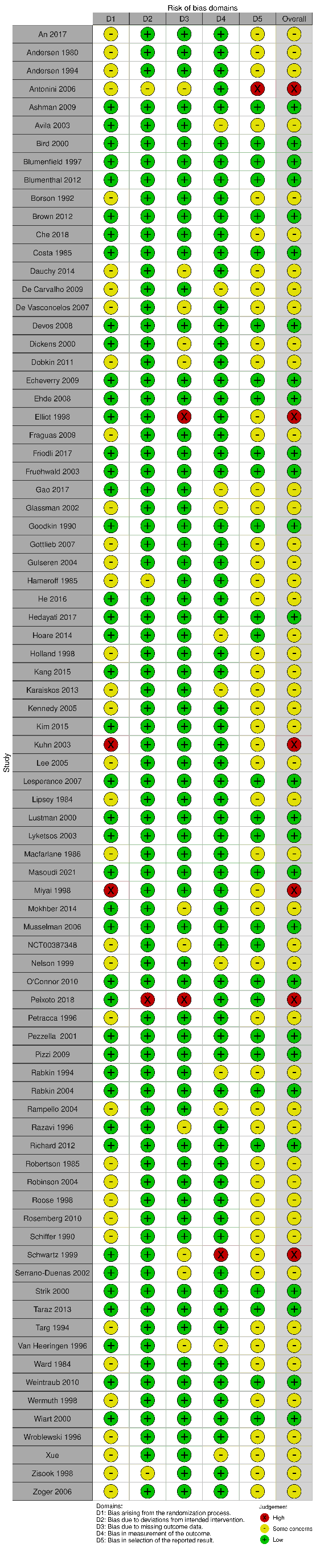


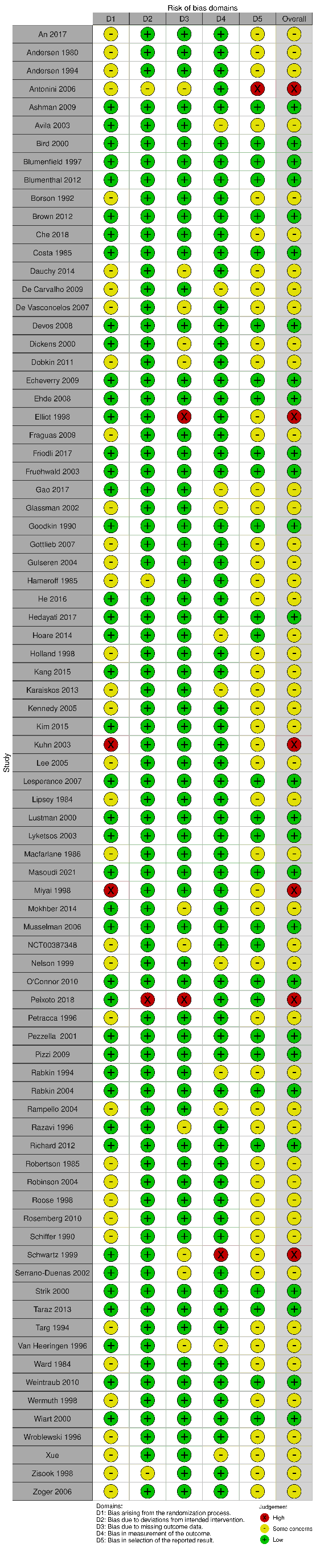


**F - Transitivity assessment and meta-regression analyses**

**Continuous variables**

We represented the distribution of the variable within each treatment strategy as a boxplots and performed the Kruskal-Wallis equality-of-populations rank test. The results of the meta-regression analyses are also reported.

The variables “mean duration of depression” and “number of medical comorbidities” could not be performed, as they were seldom reported by original studies.

| **Potential effect modifiers** | **Boxplot** | **Kruskal-Wallis equality-of-populations rank test** | **Meta-regression analysis** |
| --- | --- | --- | --- |
| Year of publication | 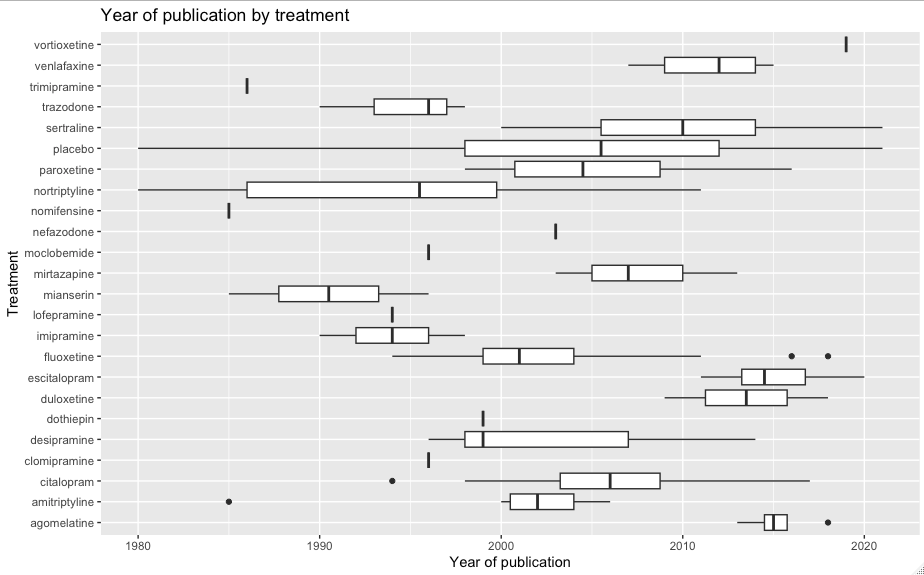 | chi-squared with ties = 76.234 with 23 d.f.  p = 1.279e-07 | EFFICACY  Coeff. = 0.006  SE = 0.006  p = 0.346  TOLERABILITY  Coeff. = -0.007  SE = 0.013  p = 0.608 |
| Mean baseline severity of depressive symptoms | 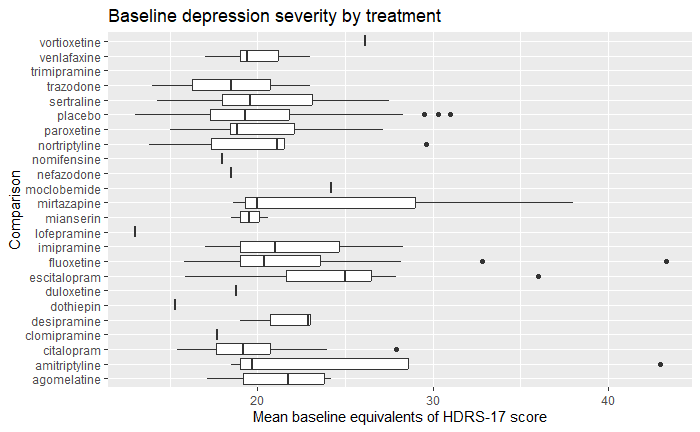 | chi-squared with ties = 22.01  with 22 d.f.  p = 0.459 | EFFICACY  Coeff. = -0.021  SE = 0.015  p = 0.167  TOLERABILITY  Coeff. = -0.015  SE = 0.046  p = 0.731 |
| Mean age | 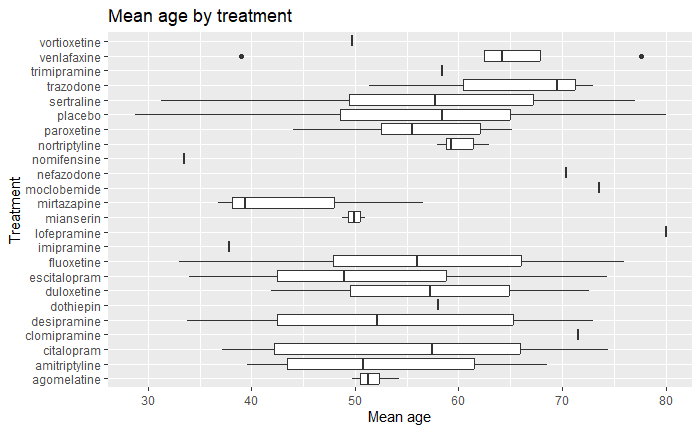 | chi-squared with ties = 26.04  with 23 d.f.  p = 0.299 | EFFICACY  Coeff. = -0.007  SE = 0.004  p = 0.133  TOLERABILITY  Coeff. = 0.010  SE = 0.013  p = 0.436 |
| Proportion of female participants | 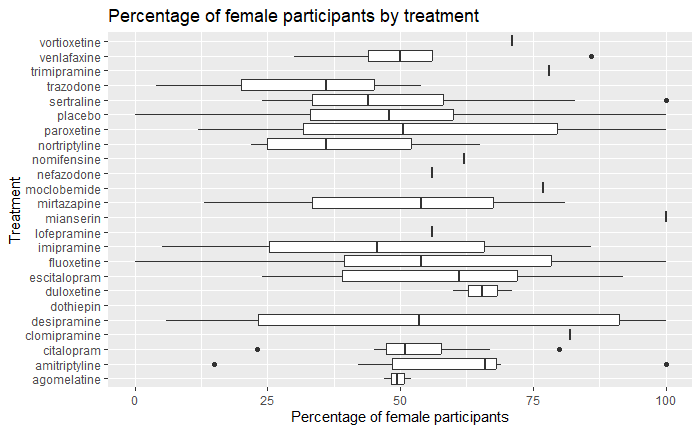 | chi-squared with ties = 18.84  with 22 d.f.  p = 0.655 | EFFICACY  Coeff. = 0.039  SE = 0.217  p = 0.856  TOLERABILITY  Coeff. = -0.301  SE = 0.530  p = 0.570 |
| Mean baseline severity of medical illness (according to a classification derived from the Severity of Illness Instrument*) | **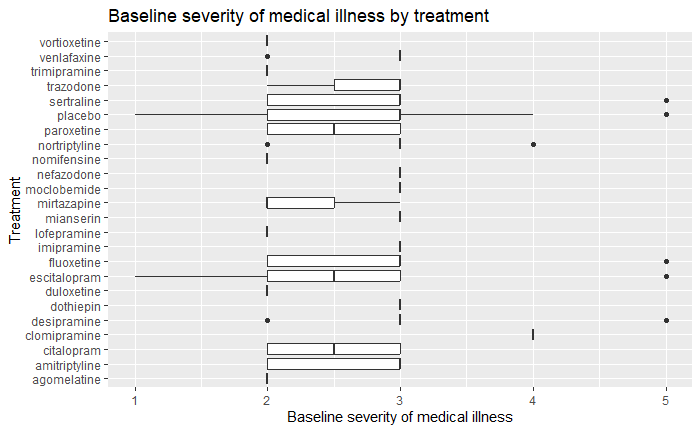**  * 1=minor manifestations; 2=minor/moderate; 3=moderate; 4=moderate/major; 5=major; 6=major/extreme; 7=extreme | chi-squared with ties = 30.84  with 23 d.f.  p = 0.127 | EFFICACY  Coeff. = -0.178  SE = 0.086  p = 0.039  TOLERABILITY  Coeff. = 0.087  SE = 0.195  p = 0.655 |
| Sample size | **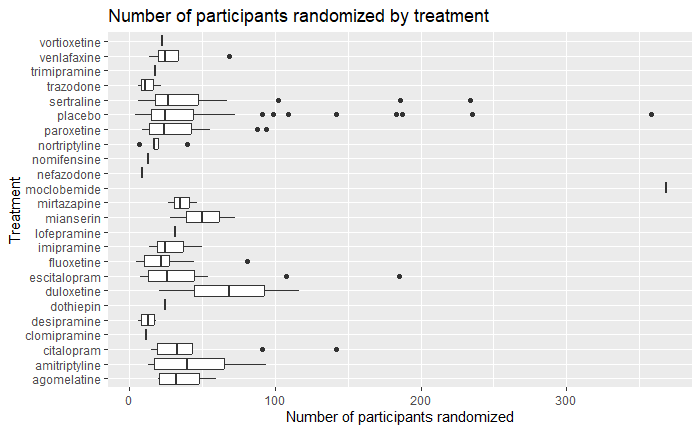** | chi-squared with ties = 31.09  with 23 d.f.  p = 0.120 | EFFICACY  Coeff. = 0.0004  SE = 0.0004  p = 0.306  TOLERABILITY  Coeff. = -0.0001  SE = 0.0007  p = 0.825 |
| Mean follow-up length (weeks) | **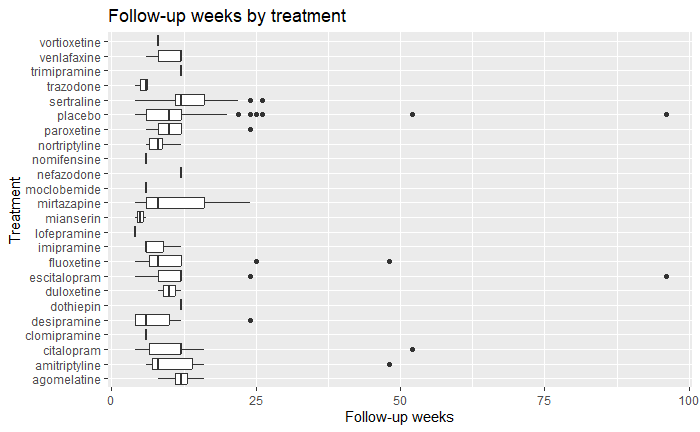** | chi-squared with ties = 29.37  with 23 d.f.  p = 0.168 | EFFICACY  Coeff. = 0.004  SE = 0.004  p = 0.321  TOLERABILITY  Coeff. = -0.011  SE = 0.018  p = 0.534 |

**Categorical variables**

| **Potential effect modifiers** | **Boxplot** | **Kruskal-Wallis equality-of-populations rank test** | **Meta-regression analysis** |
| --- | --- | --- | --- |
| Blinding (blind vs. not blind) | 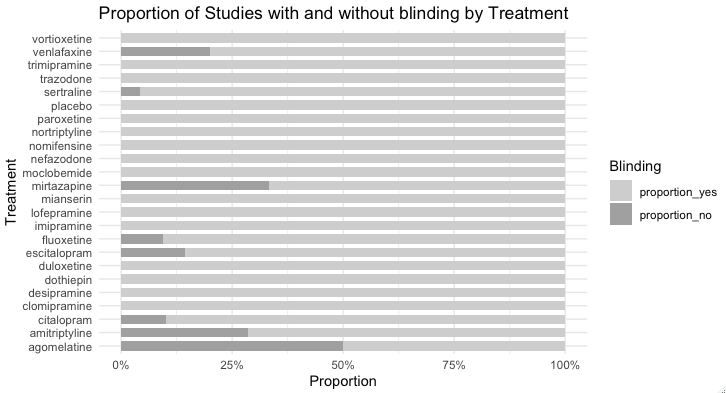 | chi-squared with ties = 38.578  with 23 d.f.  p = 0.022 | Coeff. = -0.468  SE = 12.90  p = 0.971 |
| Setting | 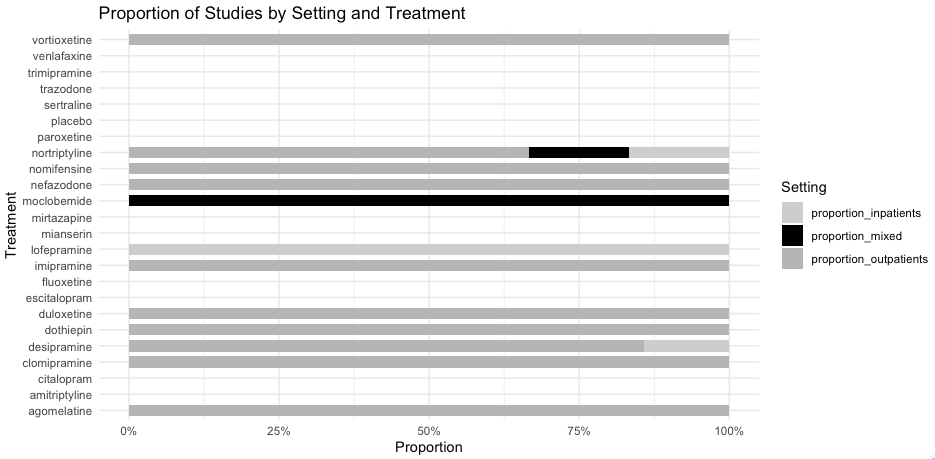 | chi-squared with ties = 18.234  with 22 d.f.  p = 0.692 | Insufficient observations |
| Sponsorship | 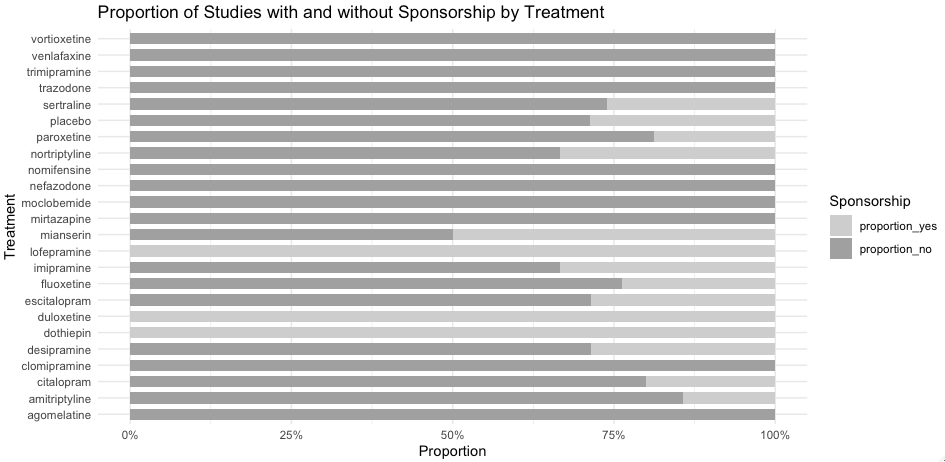 | chi-squared with ties = 21.428  with 23 d.f.  p = 0.555 | Coeff. = 0.1218  SE = 0.125  p = 0.329 |
| Depression as primary outcome | 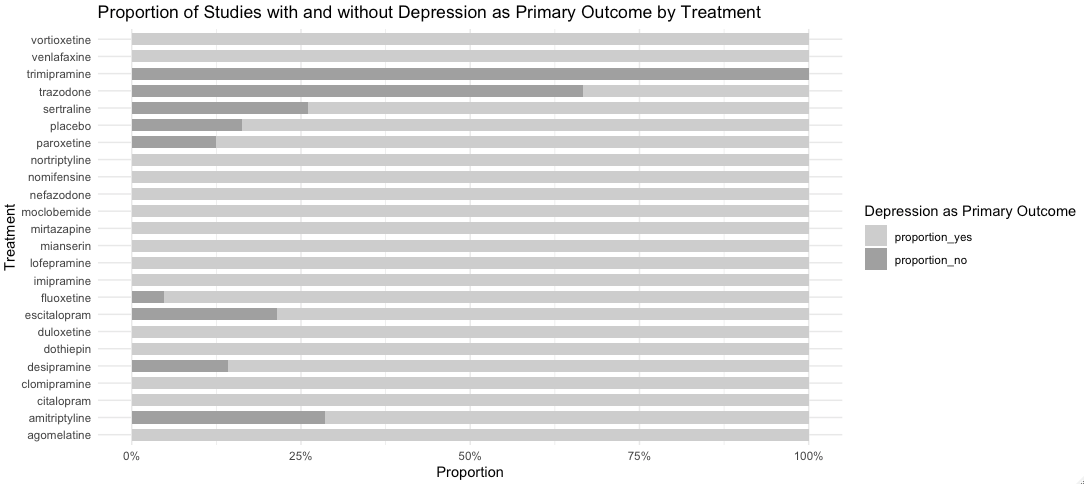 | chi-squared with ties = 25.512  with 23 d.f.  p = 0.324 | Coeff. = -0.0232  SE = 0.147  p = 0.875 |

**G - Primary outcome: efficacy (mean change at rating scales measuring depression)**

**Characteristics of the network**

Number of treatments:

24

Number of studies:

104

Number of individuals included in the RCTs:

8386

Number of individuals contributing to this analysis:

7714

Number of individuals randomized to each treatment:

Treatment name N. individuals randomized
1 agomelatine 145
2 amitriptyline 313
3 citalopram 456
4 clomipramine 12
5 desipramine 89
6 dothiepin 25
7 duloxetine 138
8 escitalopram 601
9 fluoxetine 531
10 imipramine 89
11 lofepramine 32
12 mianserin 101
13 mirtazapine 109
14 moclobemide 368
15 nefazodone 9
16 nomifensine 13
17 nortriptyline 120
18 paroxetine 540
19 placebo 3350
20 sertraline 1103
21 trazodone 39
22 trimipramine 18
23 venlafaxine 162
24 vortioxetine 23

**Pairwise meta-analysis**
Standardized mean differences below 0 favor the first treatment of the comparison.

**
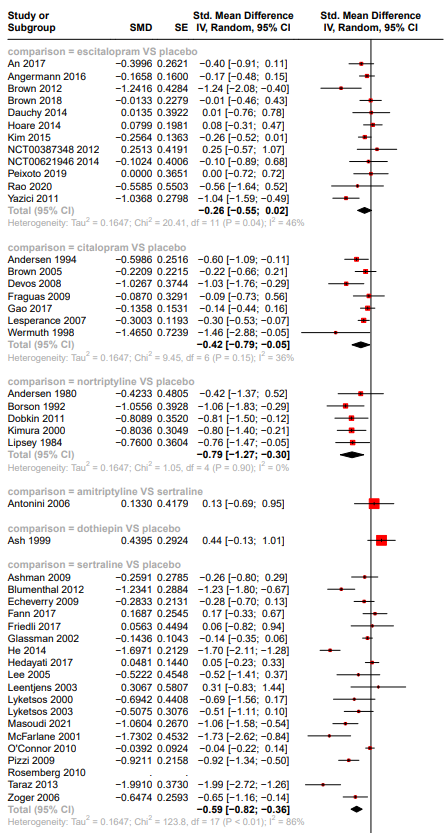
**


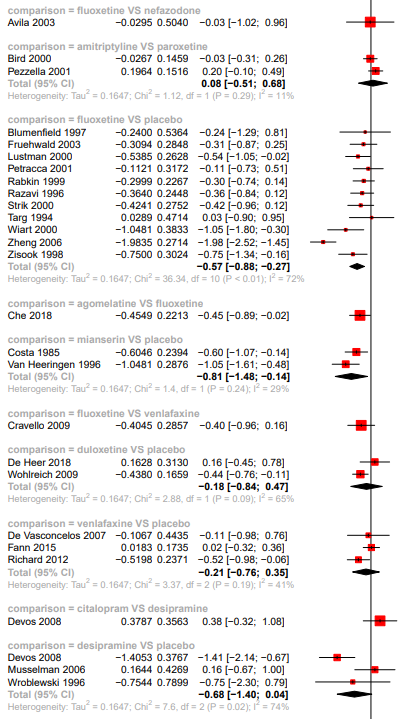


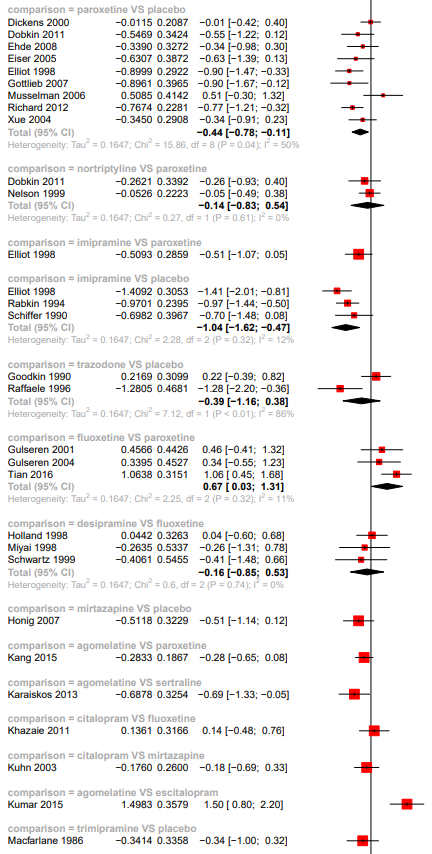


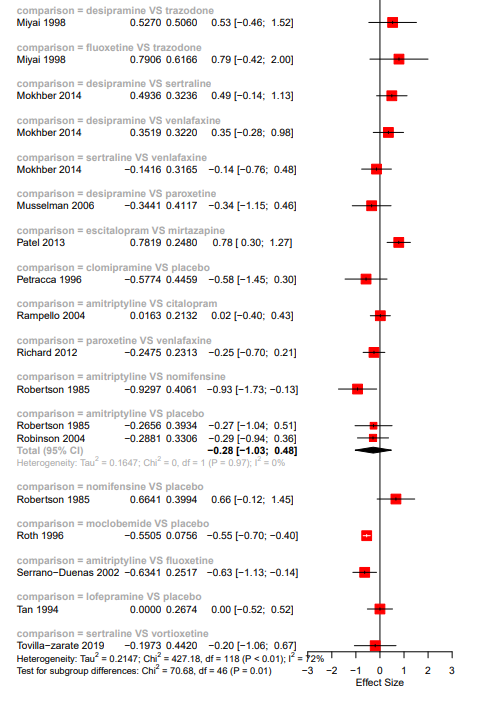


**Network map**

The thickness of lines is proportional to the number of studies comparing the two treatments and the size of circles is proportional to the number of individuals for each treatment.


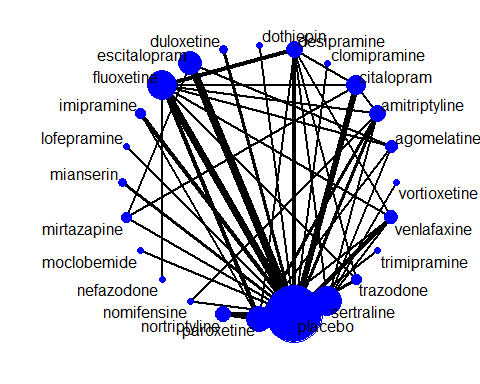


**Netleague table**

Standardized mean differences (SMDs) and 95% confidence intervals (CIs) are reported. Results of the network meta-analysis are reported in the lower left part of the table and results from the pairwise meta-analysis are reported in the upper right part of the table. SMDs lower than 1 favour the column-defining treatment.

| agomelatine | . | . | . | . | . | . | 1.50 ( 0.45 to 2.55) | -0.45 (-1.35 to 0.44) | . | . | . | . | . | . | . | . | -0.28 (-1.15 to 0.58) | . | -0.69 (-1.70 to 0.32) | . | . | . | . |
| --- | --- | --- | --- | --- | --- | --- | --- | --- | --- | --- | --- | --- | --- | --- | --- | --- | --- | --- | --- | --- | --- | --- | --- |
| -0.03 (-0.63 to 0.56) | amitriptyline | 0.02 (-0.87 to 0.90) | . | . | . | . | . | -0.63 (-1.56 to 0.29) | . | . | . | . | . | . | -0.93 (-2.05 to 0.19) | . | 0.08 (-0.51 to 0.68) | -0.28 (-1.02 to 0.47) | 0.13 (-1.00 to 1.27) | . | . | . | . |
| -0.10 (-0.68 to 0.47) | -0.07 (-0.52 to 0.38) | citalopram | . | 0.38 (-0.67 to 1.43) | . | . | . | 0.14 (-0.86 to 1.14) | . | . | . | -0.18 (-1.11 to 0.76) | . | . | . | . | . | -0.42 (-0.78 to -0.05) | . | . | . | . | . |
| 0.06 (-1.22 to 1.33) | 0.09 (-1.14 to 1.32) | 0.16 (-1.05 to 1.37) | clomipramine | . | . | . | . | . | . | . | . | . | . | . | . | . | . | -0.58 (-1.75 to 0.60) | . | . | . | . | . |
| -0.05 (-0.67 to 0.58) | -0.01 (-0.56 to 0.53) | 0.06 (-0.44 to 0.55) | -0.10 (-1.35 to 1.14) | desipramine | . | . | . | -0.16 (-0.85 to 0.52) | . | . | . | . | . | . | . | . | -0.34 (-1.47 to 0.78) | -0.68 (-1.40 to 0.03) | 0.49 (-0.51 to 1.50) | 0.53 (-0.74 to 1.79) | . | 0.35 (-0.65 to 1.36) | . |
| -0.96 (-2.05 to 0.13) | -0.93 (-1.97 to 0.11) | -0.86 (-1.88 to 0.16) | -1.02 (-2.54 to 0.51) | -0.92 (-1.97 to 0.14) | dothiepin | . | . | . | . | . | . | . | . | . | . | . | . | 0.44 (-0.53 to 1.41) | . | . | . | . | . |
| -0.34 (-1.15 to 0.48) | -0.30 (-1.05 to 0.45) | -0.23 (-0.95 to 0.48) | -0.39 (-1.73 to 0.95) | -0.29 (-1.06 to 0.48) | 0.62 (-0.54 to 1.79) | duloxetine | . | . | . | . | . | . | . | . | . | . | . | -0.19 (-0.83 to 0.46) | . | . | . | . | . |
| -0.18 (-0.71 to 0.35) | -0.15 (-0.61 to 0.31) | -0.08 (-0.48 to 0.33) | -0.24 (-1.44 to 0.97) | -0.13 (-0.63 to 0.36) | 0.78 (-0.23 to 1.79) | 0.16 (-0.54 to 0.86) | escitalopram | . | . | . | . | 0.78 (-0.14 to 1.70) | . | . | . | . | . | -0.26 (-0.55 to 0.02) | . | . | . | . | . |
| -0.14 (-0.64 to 0.37) | -0.10 (-0.51 to 0.31) | -0.03 (-0.40 to 0.33) | -0.19 (-1.39 to 1.00) | -0.09 (-0.52 to 0.34) | 0.82 (-0.17 to 1.82) | 0.20 (-0.49 to 0.89) | 0.04 (-0.31 to 0.40) | fluoxetine | . | . | . | . | . | -0.03 (-1.29 to 1.23) | . | . | 0.67 ( 0.03 to 1.31) | -0.58 (-0.88 to -0.27) | . | 0.79 (-0.65 to 2.23) | . | -0.40 (-1.37 to 0.56) | . |
| 0.49 (-0.24 to 1.21) | 0.52 (-0.13 to 1.17) | 0.59 (-0.03 to 1.21) | 0.43 (-0.86 to 1.72) | 0.53 (-0.15 to 1.21) | **1.45 ( 0.34 to 2.56)** | 0.82 (-0.02 to 1.67) | 0.67 ( 0.06 to 1.27) | **0.62 ( 0.03 to 1.21)** | imipramine | . | . | . | . | . | . | . | -0.51 (-1.47 to 0.45) | -1.05 (-1.62 to -0.47) | . | . | . | . | . |
| -0.52 (-1.58 to 0.54) | -0.49 (-1.50 to 0.53) | -0.42 (-1.41 to 0.57) | -0.58 (-2.08 to 0.93) | -0.48 (-1.51 to 0.55) | 0.44 (-0.91 to 1.79) | -0.19 (-1.33 to 0.96) | -0.34 (-1.32 to 0.64) | -0.38 (-1.36 to 0.59) | -1.01 (-2.10 to 0.08) | lofepramine | . | . | . | . | . | . | . | 0.00 (-0.94 to 0.94) | . | . | . | . | . |
| 0.29 (-0.53 to 1.12) | 0.33 (-0.44 to 1.09) | 0.40 (-0.34 to 1.13) | 0.24 (-1.11 to 1.58) | 0.34 (-0.45 to 1.12) | **1.25 ( 0.08 to 2.43)** | 0.63 (-0.30 to 1.55) | 0.47 (-0.24 to 1.19) | 0.43 (-0.28 to 1.13) | -0.19 (-1.05 to 0.66) | 0.81 (-0.34 to 1.97) | mianserin | . | . | . | . | . | . | -0.81 (-1.48 to -0.15) | . | . | . | . | . |
| 0.11 (-0.63 to 0.86) | 0.15 (-0.52 to 0.82) | 0.22 (-0.37 to 0.81) | 0.06 (-1.25 to 1.36) | 0.16 (-0.54 to 0.86) | 1.08 (-0.05 to 2.20) | 0.45 (-0.41 to 1.31) | 0.29 (-0.29 to 0.88) | 0.25 (-0.36 to 0.86) | -0.37 (-1.16 to 0.41) | 0.64 (-0.46 to 1.74) | -0.18 (-1.05 to 0.70) | mirtazapine | . | . | . | . | . | -0.51 (-1.52 to 0.49) | . | . | . | . | . |
| 0.03 (-0.91 to 0.97) | 0.06 (-0.82 to 0.94) | 0.13 (-0.72 to 0.99) | -0.03 (-1.45 to 1.39) | 0.07 (-0.82 to 0.97) | 0.99 (-0.27 to 2.25) | 0.37 (-0.66 to 1.39) | 0.21 (-0.63 to 1.05) | 0.17 (-0.67 to 1.00) | -0.46 (-1.42 to 0.51) | 0.55 (-0.68 to 1.78) | -0.26 (-1.30 to 0.77) | -0.09 (-1.06 to 0.89) | moclobemide | . | . | . | . | -0.55 (-1.35 to 0.25) | . | . | . | . | . |
| -0.17 (-1.53 to 1.19) | -0.13 (-1.46 to 1.19) | -0.06 (-1.38 to 1.25) | -0.22 (-1.96 to 1.52) | -0.12 (-1.45 to 1.21) | 0.79 (-0.81 to 2.40) | 0.17 (-1.27 to 1.61) | 0.01 (-1.30 to 1.32) | -0.03 (-1.29 to 1.23) | -0.65 (-2.04 to 0.74) | 0.36 (-1.24 to 1.95) | -0.46 (-1.90 to 0.99) | -0.28 (-1.68 to 1.12) | -0.20 (-1.71 to 1.31) | nefazodone | . | . | . | . | . | . | . | . | . |
| -1.08 (-2.17 to 0.02) | **-1.04 (-2.03 to -0.06)** | -0.97 (-2.00 to 0.05) | -1.13 (-2.66 to 0.40) | -1.03 (-2.10 to 0.03) | -0.12 (-1.50 to 1.27) | -0.74 (-1.92 to 0.44) | -0.90 (-1.92 to 0.12) | -0.94 (-1.94 to 0.07) | **-1.56 (-2.68 to -0.44)** | -0.55 (-1.92 to 0.81) | **-1.37 (-2.56 to -0.18)** | **-1.19 (-2.32 to -0.06)** | -1.11 (-2.37 to 0.16) | -0.91 (-2.52 to 0.70) | nomifensine | . | . | 0.66 (-0.44 to 1.77) | . | . | . | . | . |
| 0.22 (-0.41 to 0.86) | 0.26 (-0.29 to 0.81) | 0.33 (-0.19 to 0.84) | 0.17 (-1.08 to 1.41) | 0.27 (-0.32 to 0.85) | **1.18 ( 0.13 to 2.24)** | 0.56 (-0.21 to 1.33) | 0.40 (-0.09 to 0.90) | 0.36 (-0.11 to 0.83) | -0.26 (-0.94 to 0.42) | 0.75 (-0.29 to 1.78) | -0.07 (-0.85 to 0.72) | 0.11 (-0.60 to 0.82) | 0.19 (-0.71 to 1.10) | 0.39 (-0.96 to 1.74) | **1.30 (0.23 to 2.37)** | nortriptyline | -0.14 (-0.82 to 0.53) | -0.79 (-1.27 to -0.31) | . | . | . | . | . |
| 0.02 (-0.49 to 0.53) | 0.05 (-0.34 to 0.45) | 0.12 (-0.27 to 0.51) | -0.04 (-1.24 to 1.16) | 0.06 (-0.40 to 0.53) | 0.98 (-0.02 to 1.98) | 0.35 (-0.34 to 1.05) | 0.20 (-0.17 to 0.57) | 0.16 (-0.15 to 0.47) | -0.47 (-1.04 to 0.11) | 0.54 (-0.44 to 1.52) | -0.27 (-0.98 to 0.44) | -0.10 (-0.72 to 0.52) | -0.01 (-0.85 to 0.83) | 0.19 (-1.11 to 1.48) | **1.10 ( 0.09 to 2.10)** | -0.21 (-0.65 to 0.24) | paroxetine | -0.44 (-0.78 to -0.11) | . | . | . | -0.25 (-1.15 to 0.66) | . |
| **-0.52 (-1.01 to -0.03)** | **-0.49 (-0.87 to -0.11)** | **-0.42 (-0.73 to -0.11)** | -0.58 (-1.75 to 0.60) | **-0.48 (-0.89 to -0.06)** | 0.44 (-0.53 to 1.41) | -0.19 (-0.83 to 0.46) | **-0.34 (-0.61 to -0.07)** | **-0.38 (-0.62 to -0.15)** | **-1.01 (-1.55 to -0.46)** | 0.00 (-0.94 to 0.94) | **-0.81 (-1.48 to -0.15)** | **-0.64 (-1.20 to -0.07)** | -0.55 (-1.35 to 0.25) | -0.36 (-1.64 to 0.93) | 0.55 (-0.43 to 1.54) | **-0.75 (-1.16 to -0.33)** | **-0.54 (-0.80 to -0.28)** | placebo | 0.59 ( 0.37 to 0.82) | 0.39 (-0.38 to 1.15) | 0.34 (-0.68 to 1.36) | 0.21 (-0.34 to 0.76) | . |
| 0.04 (-0.47 to 0.56) | 0.08 (-0.34 to 0.50) | 0.15 (-0.22 to 0.52) | -0.01 (-1.20 to 1.18) | 0.09 (-0.36 to 0.54) | **1.01 ( 0.01 to 2.00)** | 0.38 (-0.30 to 1.06) | 0.22 (-0.12 to 0.57) | 0.18 (-0.13 to 0.49) | -0.44 (-1.02 to 0.14) | 0.57 (-0.40 to 1.53) | -0.25 (-0.95 to 0.45) | -0.07 (-0.68 to 0.54) | 0.02 (-0.81 to 0.84) | 0.21 (-1.09 to 1.51) | **1.12 ( 0.12 to 2.12)** | -0.18 (-0.65 to 0.29) | 0.03 (-0.30 to 0.35) | **0.57 ( 0.35 to 0.78)** | sertraline | . | . | -0.14 (-1.14 to 0.86) | -0.20 (-1.36 to 0.97) |
| 0.07 (-0.74 to 0.88) | 0.10 (-0.64 to 0.85) | 0.17 (-0.54 to 0.88) | 0.01 (-1.33 to 1.35) | 0.11 (-0.61 to 0.84) | 1.03 (-0.14 to 2.20) | 0.40 (-0.51 to 1.32) | 0.25 (-0.45 to 0.95) | 0.20 (-0.47 to 0.88) | -0.42 (-1.26 to 0.43) | 0.59 (-0.55 to 1.73) | -0.22 (-1.15 to 0.70) | -0.05 (-0.91 to 0.81) | 0.04 (-0.99 to 1.07) | 0.23 (-1.20 to 1.66) | 1.14 (-0.03 to 2.32) | -0.16 (-0.93 to 0.61) | 0.05 (-0.64 to 0.74) | 0.59 (-0.06 to 1.24) | 0.02 (-0.66 to 0.70) | trazodone | . | . | . |
| -0.18 (-1.32 to 0.95) | -0.15 (-1.24 to 0.94) | -0.08 (-1.15 to 0.99) | -0.24 (-1.79 to 1.32) | -0.13 (-1.24 to 0.97) | 0.78 (-0.63 to 2.19) | 0.16 (-1.05 to 1.37) | 0.00 (-1.06 to 1.06) | -0.04 (-1.09 to 1.01) | -0.67 (-1.82 to 0.49) | 0.34 (-1.05 to 1.73) | -0.47 (-1.69 to 0.75) | -0.29 (-1.46 to 0.88) | -0.21 (-1.51 to 1.09) | -0.01 (-1.65 to 1.63) | 0.90 (-0.52 to 2.32) | -0.40 (-1.51 to 0.70) | -0.20 (-1.25 to 0.86) | 0.34 (-0.68 to 1.36) | -0.22 (-1.27 to 0.82) | -0.25 (-1.46 to 0.96) | trimipramine | . | . |
| -0.27 (-0.90 to 0.35) | -0.24 (-0.79 to 0.30) | -0.17 (-0.68 to 0.34) | -0.33 (-1.57 to 0.91) | -0.23 (-0.77 to 0.31) | 0.69 (-0.37 to 1.74) | 0.06 (-0.70 to 0.83) | -0.09 (-0.59 to 0.40) | -0.14 (-0.58 to 0.31) | **-0.76 (-1.44 to -0.08)** | 0.25 (-0.78 to 1.28) | -0.57 (-1.35 to 0.21) | -0.39 (-1.09 to 0.31) | -0.30 (-1.20 to 0.59) | -0.11 (-1.44 to 1.23) | 0.80 (-0.26 to 1.87) | -0.50 (-1.08 to 0.08) | -0.29 (-0.75 to 0.17) | 0.25 (-0.17 to 0.66) | -0.32 (-0.77 to 0.13) | -0.34 (-1.10 to 0.42) | -0.09 (-1.20 to 1.01) | venlafaxine | . |
| -0.15 (-1.43 to 1.12) | -0.12 (-1.36 to 1.12) | -0.05 (-1.28 to 1.18) | -0.21 (-1.88 to 1.46) | -0.11 (-1.36 to 1.15) | 0.81 (-0.73 to 2.34) | 0.18 (-1.17 to 1.53) | 0.03 (-1.19 to 1.24) | -0.02 (-1.22 to 1.19) | -0.64 (-1.94 to 0.67) | 0.37 (-1.15 to 1.88) | -0.45 (-1.81 to 0.91) | -0.27 (-1.58 to 1.05) | -0.18 (-1.61 to 1.25) | 0.01 (-1.73 to 1.76) | 0.92 (-0.62 to 2.46) | -0.38 (-1.64 to 0.88) | -0.17 (-1.38 to 1.04) | 0.37 (-0.82 to 1.56) | -0.20 (-1.36 to 0.97) | -0.22 (-1.57 to 1.13) | 0.03 (-1.54 to 1.59) | 0.12 (-1.13 to 1.37) | vortioxetine |

**Forest plot**

Placebo was used as a common comparator. SMDs below 0 favor the treatment over the common comparator.


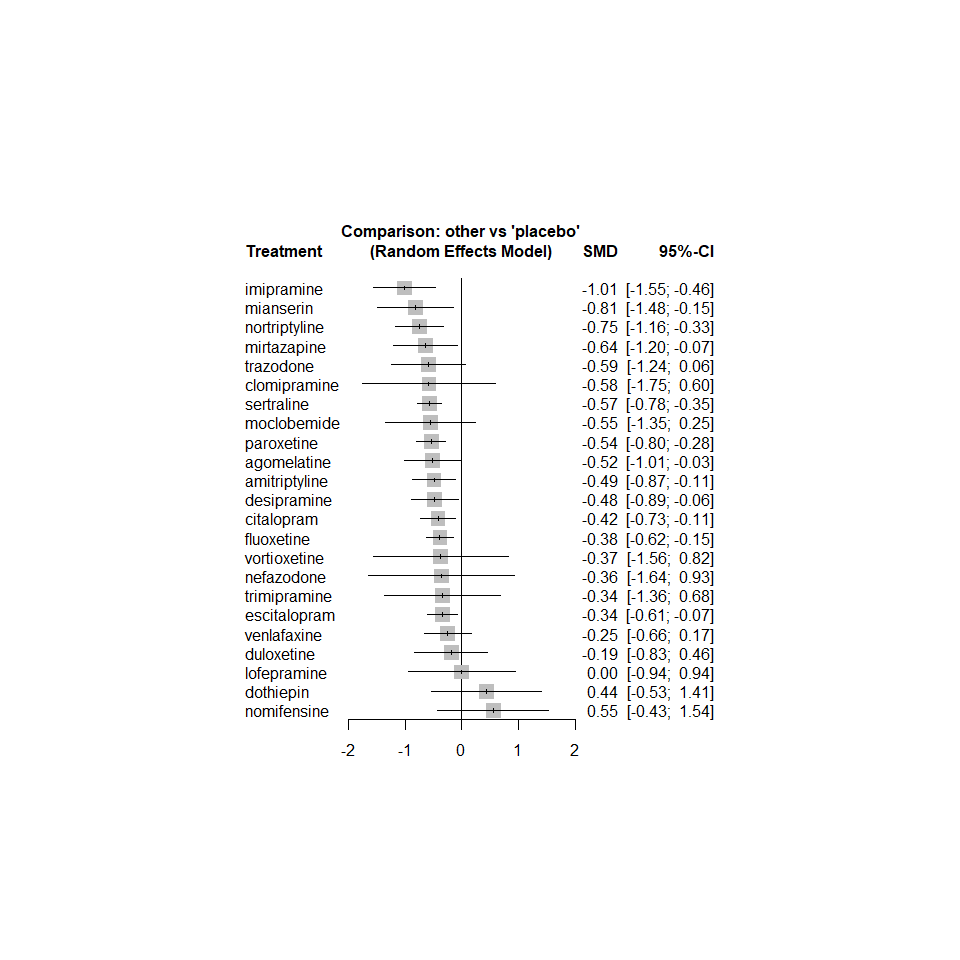


**Assessment of heterogeneity and consistency**

*Global heterogeneity*

We interpreted tau^2 as follows: heterogeneity low with tau^2≤0.010, moderate with 0.010<tau^2≤0.242, high with tau^2>0.242, and I^2 statistics as follows: not important (0%-40%), moderate (30%-60%), substantial (50%-90%), considerable (75%-100%).

tau^2= 0.1595; tau= 0.3994

I^2= 70 % (62.81 % to 75.79 %)

Interpretation: moderate heterogeneity

*Consistency: global approach*

Q statistic to assess consistency under the assumption of
a full design-by-treatment interaction random effects model

 Q df p-value tau.within tau2.within
Between designs 32.41 29 0.3021 0.3967 0.1574

*Consistency: local approach*

Separate indirect from direct evidence (SIDE) using back-calculation method

Random effects model:

 comparison k prop nma direct indir. Diff z p-value
 agomelatine:escitalopram 1 0.26 -0.18 1.50 -0.76 2.26 3.63 0.0003
 agomelatine:fluoxetine 1 0.32 -0.14 -0.45 0.01 -0.47 -0.85 0.3977
 agomelatine:paroxetine 1 0.35 0.02 -0.28 0.18 -0.46 -0.85 0.3965
 agomelatine:sertraline 1 0.26 0.04 -0.69 0.30 -0.99 -1.65 0.0986
 amitriptyline:citalopram 1 0.26 -0.07 0.02 -0.10 0.12 0.22 0.8263
 amitriptyline:fluoxetine 1 0.20 -0.10 -0.63 0.03 -0.66 -1.26 0.2090
 amitriptyline:nomifensine 1 0.78 -1.04 -0.93 -1.44 0.51 0.42 0.6731
 amitriptyline:paroxetine 2 0.44 0.05 0.08 0.03 0.06 0.14 0.8874
 placebo:amitriptyline 2 0.26 0.49 0.28 0.56 -0.28 -0.64 0.5225
 amitriptyline:sertraline 1 0.14 0.08 0.13 0.07 0.06 0.10 0.9184
 citalopram:desipramine 1 0.22 0.06 0.38 -0.03 0.41 0.68 0.4970
 citalopram:fluoxetine 1 0.14 -0.03 0.14 -0.06 0.20 0.36 0.7201
 citalopram:mirtazapine 1 0.40 0.22 -0.18 0.48 -0.66 -1.07 0.2855
 placebo:citalopram 7 0.72 0.42 0.42 0.42 -0.00 -0.01 0.9901
 desipramine:fluoxetine 3 0.40 -0.09 -0.16 -0.05 -0.11 -0.25 0.7988
 desipramine:paroxetine 1 0.17 0.06 -0.34 0.15 -0.49 -0.78 0.4344
 placebo:desipramine 3 0.34 0.48 0.68 0.37 0.32 0.70 0.4823
 desipramine:sertraline 1 0.20 0.09 0.49 -0.01 0.51 0.88 0.3787
 desipramine:trazodone 1 0.33 0.11 0.53 -0.09 0.61 0.78 0.4341
 desipramine:venlafaxine 1 0.29 -0.23 0.35 -0.47 0.82 1.35 0.1786
 escitalopram:mirtazapine 1 0.40 0.29 0.78 -0.03 0.82 1.34 0.1798
 placebo:escitalopram 12 0.89 0.34 0.26 0.99 -0.73 -1.63 0.1035
 fluoxetine:paroxetine 3 0.24 0.16 0.67 -0.00 0.68 1.81 0.0700
 placebo:fluoxetine 11 0.63 0.38 0.58 0.07 0.51 2.02 0.0432
 fluoxetine:trazodone 1 0.22 0.20 0.79 0.04 0.75 0.90 0.3667
 fluoxetine:venlafaxine 1 0.21 -0.14 -0.40 -0.06 -0.34 -0.61 0.5396
 imipramine:paroxetine 1 0.36 -0.47 -0.51 -0.44 -0.07 -0.11 0.9151
 placebo:imipramine 3 0.90 1.01 1.05 0.67 0.38 0.41 0.6839
 placebo:mirtazapine 1 0.32 0.64 0.51 0.69 -0.18 -0.29 0.7695
 placebo:nomifensine 1 0.79 -0.55 -0.66 -0.14 -0.52 -0.42 0.6731
 nortriptyline:paroxetine 2 0.44 -0.21 -0.14 -0.25 0.11 0.24 0.8099
 placebo:nortriptyline 5 0.76 0.75 0.79 0.62 0.17 0.34 0.7364
 placebo:paroxetine 9 0.60 0.54 0.44 0.68 -0.24 -0.89 0.3719
 paroxetine:venlafaxine 1 0.26 -0.29 -0.25 -0.31 0.06 0.11 0.9089
 placebo:sertraline 18 0.88 0.57 0.59 0.36 0.24 0.70 0.4870
 placebo:trazodone 2 0.71 0.59 0.39 1.10 -0.71 -0.97 0.3310
 placebo:venlafaxine 3 0.57 0.25 0.21 0.30 -0.10 -0.23 0.8214
 sertraline:venlafaxine 1 0.20 -0.32 -0.14 -0.36 0.22 0.39 0.6971

Legend:
 comparison - Treatment comparison
 k - Number of studies providing direct evidence
 prop - Direct evidence proportion
 nma - Estimated treatment effect (SMD) in network meta-analysis
 direct - Estimated treatment effect (SMD) derived from direct evidence
 indir. - Estimated treatment effect (SMD) derived from indirect evidence
 Diff - Difference between direct and indirect treatment estimates
 z - z-value of test for disagreement (direct versus indirect)
 p-value - p-value of test for disagreement (direct versus indirect)

**Netrank**

Higher p-score values indicate higher ranking of treatments

P-score
imipramine 0.8999
mianserin 0.7848
nortriptyline 0.7794
mirtazapine 0.6792
sertraline 0.6494
trazodone 0.6356
paroxetine 0.6206
moclobemide 0.5978
clomipramine 0.5965
agomelatine 0.5905
amitriptyline 0.5623
desipramine 0.5512
citalopram 0.4911
vortioxetine 0.4815
nefazodone 0.4764
trimipramine 0.4616
fluoxetine 0.4510
escitalopram 0.4125
venlafaxine 0.3449
duloxetine 0.3310
lofepramine 0.2626
placebo 0.1640
dothiepin 0.1013
nomifensine 0.0749

**Assessment of small-study effect**

We produced funnel plots and performed the Egger’s test only for those comparisons including at least 8 studies.

| **Funnel plot** | **Egger’s test** |
| --- | --- |
| Escitalopram vs. placebo   | Egger's test for small-study effects:  Regress standard normal deviate of intervention  effect estimate against its standard error  .  Number of studies = 12 Root MSE = 1.371  ------------------------------------------------------------------------------  Std_Eff \| Coefficient Std. err. t P>\|t\| [95% conf. interval]  -------------+----------------------------------------------------------------  slope \| -.0973341 .2389691 -0.41 0.692 -.6297904 .4351222  bias \| -.5608655 .974098 -0.58 0.577 -2.731291 1.60956  ------------------------------------------------------------------------------  Test of H0: no small-study effects P = 0.577 |
| Sertraline vs. placebo   | Egger's test for small-study effects:  Regress standard normal deviate of intervention  effect estimate against its standard error  .  Number of studies = 18 Root MSE = 2.352  ------------------------------------------------------------------------------  Std_Eff \| Coefficient Std. err. t P>\|t\| [95% conf. interval]  -------------+----------------------------------------------------------------  slope \| .1202502 .2266587 0.53 0.603 -.3602447 .6007452  bias \| -2.63443 1.110583 -2.37 0.031 -4.98876 -.2800996  ------------------------------------------------------------------------------  Test of H0: no small-study effects P = 0.031 |
| Fluoxetine vs. placebo   | Egger's test for small-study effects:  Regress standard normal deviate of intervention  effect estimate against its standard error  .  Number of studies = 10 Root MSE = 2.088  ------------------------------------------------------------------------------  Std_Eff \| Coefficient Std. err. t P>\|t\| [95% conf. interval]  -------------+----------------------------------------------------------------  slope \| -.6297118 1.027377 -0.61 0.557 -2.998848 1.739424  bias \| .1619143 3.582958 0.05 0.965 -8.100402 8.424231  ------------------------------------------------------------------------------  Test of H0: no small-study effects P = 0.965 |
| Paroxetine vs. placebo   | Egger's test for small-study effects:  Regress standard normal deviate of intervention  effect estimate against its standard error  .  Number of studies = 9 Root MSE = 1.478  ------------------------------------------------------------------------------  Std_Eff \| Coefficient Std. err. t P>\|t\| [95% conf. interval]  -------------+----------------------------------------------------------------  slope \| -.3317418 .6266593 -0.53 0.613 -1.813555 1.150072  bias \| -.3297583 2.112358 -0.16 0.880 -5.324692 4.665175  ------------------------------------------------------------------------------  Test of H0: no small-study effects P = 0.880 |

**CINeMA assessment**

We conducted the analysis with CINeMA according to the following setup:

- Within-study Bias: we summarize risk of bias across contributions for each network estimate according to the “majority” RoB;
- Reporting Bias: we set all comparisons to “low risk”;
- Indirectness: we summarize risk of indirectness across contributions for each network estimate according to the “majority” indirectness;
- Imprecision, heterogeneity, incoherence: we defined relative effect estimates below -0.500 and above 0.500 to be clinically important.

*Final report*

| **Comparison** | **Number of studies** | **Within-study bias** | **Reporting bias** | **Indirectness** | **Imprecision** | **Heterogeneity** | **Incoherence** | **Confidence rating** |
| --- | --- | --- | --- | --- | --- | --- | --- | --- |
| **agomelatine:escitalopram** | 1 | Some concerns | Low risk | No concerns | Some concerns | Some concerns | Major concerns | Very low |
| **agomelatine:fluoxetine** | 1 | Some concerns | Low risk | No concerns | Some concerns | Some concerns | No concerns | Very low |
| **agomelatine:paroxetine** | 1 | Some concerns | Low risk | No concerns | Major concerns | No concerns | No concerns | Very low |
| **agomelatine:sertraline** | 1 | Some concerns | Low risk | No concerns | Major concerns | No concerns | No concerns | Very low |
| **amitriptyline:fluoxetine** | 1 | Major concerns | Low risk | No concerns | Some concerns | Some concerns | No concerns | Very low |
| **amitriptyline:nomifensine** | 1 | Some concerns | Low risk | No concerns | No concerns | Some concerns | No concerns | Low |
| **amitriptyline:paroxetine** | 2 | No concerns | Low risk | No concerns | No concerns | Major concerns | No concerns | Low |
| **amitriptyline:placebo** | 2 | Some concerns | Low risk | No concerns | No concerns | Some concerns | No concerns | Low |
| **amitriptyline:sertraline** | 1 | Some concerns | Low risk | No concerns | No concerns | Major concerns | No concerns | Very low |
| **citalopram:desipramine** | 1 | No concerns | Low risk | No concerns | Some concerns | Some concerns | No concerns | Low |
| **citalopram:fluoxetine** | 1 | Some concerns | Low risk | No concerns | Some concerns | Some concerns | No concerns | Very low |
| **citalopram:placebo** | 8 | Some concerns | Low risk | No concerns | No concerns | Some concerns | No concerns | Low |
| **clomipramine:placebo** | 1 | Some concerns | Low risk | Some concerns | Major concerns | No concerns | No concerns | Very low |
| **desipramine:fluoxetine** | 3 | Major concerns | Low risk | No concerns | Some concerns | Some concerns | No concerns | Very low |
| **desipramine:paroxetine** | 1 | No concerns | Low risk | No concerns | Some concerns | Some concerns | No concerns | Low |
| **desipramine:placebo** | 3 | No concerns | Low risk | No concerns | No concerns | Some concerns | No concerns | Moderate |
| **desipramine:sertraline** | 1 | Major concerns | Low risk | Some concerns | Some concerns | Some concerns | No concerns | Very low |
| **desipramine:trazodone** | 1 | Major concerns | Low risk | Some concerns | Major concerns | No concerns | No concerns | Very low |
| **desipramine:venlafaxine** | 1 | Major concerns | Low risk | Some concerns | Some concerns | Some concerns | No concerns | Very low |
| **dothiepin:placebo** | 1 | Some concerns | Low risk | No concerns | Major concerns | No concerns | No concerns | Very low |
| **duloxetine:placebo** | 2 | Major concerns | Low risk | No concerns | Some concerns | Some concerns | No concerns | Very low |
| **escitalopram:mirtazapine** | 1 | Some concerns | Low risk | No concerns | Some concerns | Some concerns | No concerns | Very low |
| **escitalopram:placebo** | 12 | Some concerns | Low risk | No concerns | No concerns | Major concerns | No concerns | Very low |
| **fluoxetine:nefazodone** | 1 | Major concerns | Low risk | No concerns | Major concerns | No concerns | No concerns | Very low |
| **fluoxetine:paroxetine** | 3 | Some concerns | Low risk | No concerns | No concerns | Major concerns | Some concerns | Very low |
| **fluoxetine:placebo** | 10 | Some concerns | Low risk | No concerns | No concerns | Some concerns | Some concerns | Very low |
| **fluoxetine:trazodone** | 1 | Major concerns | Low risk | No concerns | Major concerns | No concerns | No concerns | Very low |
| **fluoxetine:venlafaxine** | 1 | Major concerns | Low risk | No concerns | Some concerns | Some concerns | No concerns | Very low |
| **imipramine:paroxetine** | 1 | Major concerns | Low risk | No concerns | Some concerns | Some concerns | No concerns | Very low |
| **imipramine:placebo** | 3 | Some concerns | Low risk | No concerns | No concerns | Some concerns | No concerns | Low |
| **lofepramine:placebo** | 1 | Major concerns | Low risk | No concerns | Major concerns | No concerns | No concerns | Very low |
| **mianserin:placebo** | 2 | Some concerns | Low risk | No concerns | No concerns | Some concerns | No concerns | Low |
| **mirtazapine:placebo** | 1 | Major concerns | Low risk | No concerns | No concerns | Some concerns | No concerns | Very low |
| **moclobemide:placebo** | 1 | Major concerns | Low risk | Some concerns | Some concerns | Some concerns | No concerns | Very low |
| **nomifensine:placebo** | 1 | Some concerns | Low risk | No concerns | Some concerns | Some concerns | No concerns | Very low |
| **nortriptyline:paroxetine** | 2 | Some concerns | Low risk | No concerns | Some concerns | Some concerns | No concerns | Very low |
| **nortriptyline:placebo** | 5 | Some concerns | Low risk | No concerns | No concerns | Some concerns | No concerns | Low |
| **paroxetine:placebo** | 9 | Some concerns | Low risk | No concerns | No concerns | Some concerns | No concerns | Low |
| **paroxetine:venlafaxine** | 1 | No concerns | Low risk | Some concerns | Some concerns | Some concerns | No concerns | Very low |
| **placebo:sertraline** | 19 | Some concerns | Suspected | Some concerns | No concerns | Some concerns | No concerns | Very low |
| **placebo:trazodone** | 2 | Major concerns | Low risk | No concerns | Some concerns | No concerns | No concerns | Very low |
| **placebo:trimipramine** | 1 | Some concerns | Low risk | Some concerns | Major concerns | No concerns | No concerns | Very low |
| **placebo:venlafaxine** | 3 | Major concerns | Low risk | Some concerns | Some concerns | Some concerns | No concerns | Very low |
| **sertraline:venlafaxine** | 1 | Major concerns | Low risk | Some concerns | Some concerns | Some concerns | No concerns | Very low |
| **sertraline:vortioxetine** | 1 | Major concerns | Low risk | No concerns | Major concerns | No concerns | No concerns | Very low |
| **agomelatine:amitriptyline** | 0 | Some concerns | Low risk | No concerns | Major concerns | No concerns | No concerns | Very low |
| **agomelatine:citalopram** | 0 | Some concerns | Low risk | No concerns | Major concerns | No concerns | No concerns | Very low |
| **agomelatine:clomipramine** | 0 | Some concerns | Low risk | Some concerns | Major concerns | No concerns | No concerns | Very low |
| **agomelatine:desipramine** | 0 | Some concerns | Low risk | No concerns | Major concerns | No concerns | No concerns | Very low |
| **agomelatine:dothiepin** | 0 | Some concerns | Low risk | No concerns | Some concerns | No concerns | No concerns | Low |
| **agomelatine:duloxetine** | 0 | Some concerns | Low risk | No concerns | Some concerns | Some concerns | No concerns | Very low |
| **agomelatine:imipramine** | 0 | Some concerns | Low risk | No concerns | Some concerns | Some concerns | No concerns | Very low |
| **agomelatine:lofepramine** | 0 | Some concerns | Low risk | No concerns | Major concerns | No concerns | No concerns | Very low |
| **agomelatine:mianserin** | 0 | Some concerns | Low risk | No concerns | Major concerns | No concerns | No concerns | Very low |
| **agomelatine:mirtazapine** | 0 | Some concerns | Low risk | No concerns | Major concerns | No concerns | No concerns | Very low |
| **agomelatine:moclobemide** | 0 | Some concerns | Low risk | Some concerns | Major concerns | No concerns | No concerns | Very low |
| **agomelatine:nefazodone** | 0 | Some concerns | Low risk | No concerns | Major concerns | No concerns | No concerns | Very low |
| **agomelatine:nomifensine** | 0 | Some concerns | Low risk | No concerns | Some concerns | No concerns | No concerns | Low |
| **agomelatine:nortriptyline** | 0 | Some concerns | Low risk | No concerns | Some concerns | Some concerns | No concerns | Very low |
| **agomelatine:placebo** | 0 | Some concerns | Low risk | No concerns | No concerns | Some concerns | No concerns | Low |
| **agomelatine:trazodone** | 0 | Some concerns | Low risk | No concerns | Major concerns | No concerns | No concerns | Very low |
| **agomelatine:trimipramine** | 0 | Some concerns | Low risk | Some concerns | Major concerns | No concerns | No concerns | Very low |
| **agomelatine:venlafaxine** | 0 | Some concerns | Low risk | Some concerns | Some concerns | Some concerns | No concerns | Very low |
| **agomelatine:vortioxetine** | 0 | Some concerns | Low risk | No concerns | Major concerns | No concerns | No concerns | Very low |
| **amitriptyline:citalopram** | 0 | Some concerns | Low risk | No concerns | Major concerns | No concerns | No concerns | Very low |
| **amitriptyline:clomipramine** | 0 | Some concerns | Low risk | Some concerns | Major concerns | No concerns | No concerns | Very low |
| **amitriptyline:desipramine** | 0 | Major concerns | Low risk | No concerns | Major concerns | No concerns | No concerns | Very low |
| **amitriptyline:dothiepin** | 0 | Some concerns | Low risk | No concerns | Some concerns | No concerns | No concerns | Low |
| **amitriptyline:duloxetine** | 0 | Some concerns | Low risk | No concerns | Some concerns | Some concerns | No concerns | Very low |
| **amitriptyline:escitalopram** | 0 | Some concerns | Low risk | No concerns | Some concerns | Some concerns | No concerns | Very low |
| **amitriptyline:imipramine** | 0 | Some concerns | Low risk | No concerns | Some concerns | Some concerns | No concerns | Very low |
| **amitriptyline:lofepramine** | 0 | Major concerns | Low risk | No concerns | Major concerns | No concerns | No concerns | Very low |
| **amitriptyline:mianserin** | 0 | Some concerns | Low risk | No concerns | Some concerns | Some concerns | No concerns | Very low |
| **amitriptyline:mirtazapine** | 0 | Some concerns | Low risk | No concerns | Some concerns | Some concerns | No concerns | Very low |
| **amitriptyline:moclobemide** | 0 | Major concerns | Low risk | Some concerns | Major concerns | No concerns | No concerns | Very low |
| **amitriptyline:nefazodone** | 0 | Major concerns | Low risk | No concerns | Major concerns | No concerns | No concerns | Very low |
| **amitriptyline:nortriptyline** | 0 | Some concerns | Low risk | No concerns | Some concerns | Some concerns | No concerns | Very low |
| **amitriptyline:trazodone** | 0 | Major concerns | Low risk | No concerns | Major concerns | No concerns | No concerns | Very low |
| **amitriptyline:trimipramine** | 0 | Some concerns | Low risk | Some concerns | Major concerns | No concerns | No concerns | Very low |
| **amitriptyline:venlafaxine** | 0 | Major concerns | Low risk | No concerns | Some concerns | Some concerns | No concerns | Very low |
| **amitriptyline:vortioxetine** | 0 | Major concerns | Low risk | No concerns | Major concerns | No concerns | No concerns | Very low |
| **citalopram:clomipramine** | 0 | Some concerns | Low risk | No concerns | Major concerns | No concerns | No concerns | Very low |
| **citalopram:dothiepin** | 0 | Some concerns | Low risk | No concerns | Some concerns | No concerns | No concerns | Low |
| **citalopram:duloxetine** | 0 | Some concerns | Low risk | No concerns | Some concerns | Some concerns | No concerns | Very low |
| **citalopram:escitalopram** | 0 | Some concerns | Low risk | No concerns | Some concerns | Some concerns | No concerns | Very low |
| **citalopram:imipramine** | 0 | Some concerns | Low risk | No concerns | Some concerns | Some concerns | No concerns | Very low |
| **citalopram:lofepramine** | 0 | Major concerns | Low risk | No concerns | Some concerns | Some concerns | No concerns | Very low |
| **citalopram:mianserin** | 0 | Some concerns | Low risk | No concerns | Some concerns | Some concerns | No concerns | Very low |
| **citalopram:mirtazapine** | 0 | Some concerns | Low risk | No concerns | Some concerns | Some concerns | No concerns | Very low |
| **citalopram:moclobemide** | 0 | Major concerns | Low risk | No concerns | Major concerns | No concerns | No concerns | Very low |
| **citalopram:nefazodone** | 0 | Major concerns | Low risk | No concerns | Major concerns | No concerns | No concerns | Very low |
| **citalopram:nomifensine** | 0 | Some concerns | Low risk | No concerns | Some concerns | No concerns | No concerns | Low |
| **citalopram:nortriptyline** | 0 | Some concerns | Low risk | No concerns | Some concerns | Some concerns | No concerns | Very low |
| **citalopram:paroxetine** | 0 | Some concerns | Low risk | No concerns | No concerns | Major concerns | No concerns | Very low |
| **citalopram:sertraline** | 0 | Some concerns | Low risk | No concerns | No concerns | Major concerns | No concerns | Very low |
| **citalopram:trazodone** | 0 | Major concerns | Low risk | No concerns | Major concerns | No concerns | No concerns | Very low |
| **citalopram:trimipramine** | 0 | Some concerns | Low risk | No concerns | Major concerns | No concerns | No concerns | Very low |
| **citalopram:venlafaxine** | 0 | Major concerns | Low risk | No concerns | Some concerns | Some concerns | No concerns | Very low |
| **citalopram:vortioxetine** | 0 | Major concerns | Low risk | No concerns | Major concerns | No concerns | No concerns | Very low |
| **clomipramine:desipramine** | 0 | Some concerns | Low risk | Some concerns | Major concerns | No concerns | No concerns | Very low |
| **clomipramine:dothiepin** | 0 | Some concerns | Low risk | No concerns | Major concerns | No concerns | No concerns | Very low |
| **clomipramine:duloxetine** | 0 | Some concerns | Low risk | No concerns | Major concerns | No concerns | No concerns | Very low |
| **clomipramine:escitalopram** | 0 | Some concerns | Low risk | Some concerns | Major concerns | No concerns | No concerns | Very low |
| **clomipramine:fluoxetine** | 0 | Some concerns | Low risk | Some concerns | Major concerns | No concerns | No concerns | Very low |
| **clomipramine:imipramine** | 0 | Some concerns | Low risk | No concerns | Major concerns | No concerns | No concerns | Very low |
| **clomipramine:lofepramine** | 0 | Some concerns | Low risk | No concerns | Major concerns | No concerns | No concerns | Very low |
| **clomipramine:mianserin** | 0 | Some concerns | Low risk | No concerns | Major concerns | No concerns | No concerns | Very low |
| **clomipramine:mirtazapine** | 0 | Some concerns | Low risk | No concerns | Major concerns | No concerns | No concerns | Very low |
| **clomipramine:moclobemide** | 0 | Some concerns | Low risk | Some concerns | Major concerns | No concerns | No concerns | Very low |
| **clomipramine:nefazodone** | 0 | Some concerns | Low risk | No concerns | Major concerns | No concerns | No concerns | Very low |
| **clomipramine:nomifensine** | 0 | Some concerns | Low risk | No concerns | Some concerns | Some concerns | No concerns | Very low |
| **clomipramine:nortriptyline** | 0 | Some concerns | Low risk | No concerns | Major concerns | No concerns | No concerns | Very low |
| **clomipramine:paroxetine** | 0 | Some concerns | Low risk | Some concerns | Major concerns | No concerns | No concerns | Very low |
| **clomipramine:sertraline** | 0 | Some concerns | Low risk | Some concerns | Major concerns | No concerns | No concerns | Very low |
| **clomipramine:trazodone** | 0 | Some concerns | Low risk | Some concerns | Major concerns | No concerns | No concerns | Very low |
| **clomipramine:trimipramine** | 0 | Some concerns | Low risk | Some concerns | Major concerns | No concerns | No concerns | Very low |
| **clomipramine:venlafaxine** | 0 | Some concerns | Low risk | Some concerns | Major concerns | No concerns | No concerns | Very low |
| **clomipramine:vortioxetine** | 0 | Some concerns | Low risk | Some concerns | Major concerns | No concerns | No concerns | Very low |
| **desipramine:dothiepin** | 0 | Some concerns | Low risk | No concerns | Some concerns | No concerns | No concerns | Low |
| **desipramine:duloxetine** | 0 | Major concerns | Low risk | No concerns | Some concerns | Some concerns | No concerns | Very low |
| **desipramine:escitalopram** | 0 | Some concerns | Low risk | No concerns | Some concerns | Some concerns | No concerns | Very low |
| **desipramine:imipramine** | 0 | Some concerns | Low risk | No concerns | Some concerns | Some concerns | No concerns | Very low |
| **desipramine:lofepramine** | 0 | Major concerns | Low risk | No concerns | Major concerns | No concerns | No concerns | Very low |
| **desipramine:mianserin** | 0 | Major concerns | Low risk | No concerns | Some concerns | Some concerns | No concerns | Very low |
| **desipramine:mirtazapine** | 0 | Major concerns | Low risk | No concerns | Some concerns | Some concerns | No concerns | Very low |
| **desipramine:moclobemide** | 0 | Major concerns | Low risk | Some concerns | Major concerns | No concerns | No concerns | Very low |
| **desipramine:nefazodone** | 0 | Major concerns | Low risk | No concerns | Major concerns | No concerns | No concerns | Very low |
| **desipramine:nomifensine** | 0 | Some concerns | Low risk | No concerns | Some concerns | No concerns | No concerns | Low |
| **desipramine:nortriptyline** | 0 | Some concerns | Low risk | No concerns | Some concerns | Some concerns | No concerns | Very low |
| **desipramine:trimipramine** | 0 | Some concerns | Low risk | Some concerns | Major concerns | No concerns | No concerns | Very low |
| **desipramine:vortioxetine** | 0 | Major concerns | Low risk | No concerns | Major concerns | No concerns | No concerns | Very low |
| **dothiepin:duloxetine** | 0 | Some concerns | Low risk | No concerns | Major concerns | No concerns | No concerns | Very low |
| **dothiepin:escitalopram** | 0 | Some concerns | Low risk | No concerns | Some concerns | Some concerns | No concerns | Very low |
| **dothiepin:fluoxetine** | 0 | Some concerns | Low risk | No concerns | Some concerns | No concerns | No concerns | Low |
| **dothiepin:imipramine** | 0 | Some concerns | Low risk | No concerns | No concerns | No concerns | No concerns | Moderate |
| **dothiepin:lofepramine** | 0 | Some concerns | Low risk | No concerns | Major concerns | No concerns | No concerns | Very low |
| **dothiepin:mianserin** | 0 | Some concerns | Low risk | No concerns | No concerns | Some concerns | No concerns | Low |
| **dothiepin:mirtazapine** | 0 | Some concerns | Low risk | No concerns | No concerns | Some concerns | No concerns | Low |
| **dothiepin:moclobemide** | 0 | Some concerns | Low risk | No concerns | Some concerns | Some concerns | No concerns | Very low |
| **dothiepin:nefazodone** | 0 | Some concerns | Low risk | No concerns | Major concerns | No concerns | No concerns | Very low |
| **dothiepin:nomifensine** | 0 | Some concerns | Low risk | No concerns | Major concerns | No concerns | No concerns | Very low |
| **dothiepin:nortriptyline** | 0 | Some concerns | Low risk | No concerns | No concerns | Some concerns | No concerns | Low |
| **dothiepin:paroxetine** | 0 | Some concerns | Low risk | No concerns | Some concerns | No concerns | No concerns | Low |
| **dothiepin:sertraline** | 0 | Some concerns | Low risk | No concerns | Some concerns | No concerns | No concerns | Low |
| **dothiepin:trazodone** | 0 | Some concerns | Low risk | No concerns | Some concerns | No concerns | No concerns | Low |
| **dothiepin:trimipramine** | 0 | Some concerns | Low risk | No concerns | Major concerns | No concerns | No concerns | Very low |
| **dothiepin:venlafaxine** | 0 | Some concerns | Low risk | No concerns | Some concerns | Some concerns | No concerns | Very low |
| **dothiepin:vortioxetine** | 0 | Some concerns | Low risk | No concerns | Major concerns | No concerns | No concerns | Very low |
| **duloxetine:escitalopram** | 0 | Major concerns | Low risk | No concerns | Major concerns | No concerns | No concerns | Very low |
| **duloxetine:fluoxetine** | 0 | Major concerns | Low risk | No concerns | Some concerns | Some concerns | No concerns | Very low |
| **duloxetine:imipramine** | 0 | Some concerns | Low risk | No concerns | Some concerns | No concerns | No concerns | Low |
| **duloxetine:lofepramine** | 0 | Major concerns | Low risk | No concerns | Major concerns | No concerns | No concerns | Very low |
| **duloxetine:mianserin** | 0 | Major concerns | Low risk | No concerns | Some concerns | Some concerns | No concerns | Very low |
| **duloxetine:mirtazapine** | 0 | Major concerns | Low risk | No concerns | Some concerns | Some concerns | No concerns | Very low |
| **duloxetine:moclobemide** | 0 | Major concerns | Low risk | No concerns | Major concerns | No concerns | No concerns | Very low |
| **duloxetine:nefazodone** | 0 | Major concerns | Low risk | No concerns | Major concerns | No concerns | No concerns | Very low |
| **duloxetine:nomifensine** | 0 | Some concerns | Low risk | No concerns | Some concerns | Some concerns | No concerns | Very low |
| **duloxetine:nortriptyline** | 0 | Some concerns | Low risk | No concerns | Some concerns | Some concerns | No concerns | Very low |
| **duloxetine:paroxetine** | 0 | Some concerns | Low risk | No concerns | Some concerns | Some concerns | No concerns | Very low |
| **duloxetine:sertraline** | 0 | Some concerns | Low risk | No concerns | Some concerns | Some concerns | No concerns | Very low |
| **duloxetine:trazodone** | 0 | Major concerns | Low risk | No concerns | Major concerns | No concerns | No concerns | Very low |
| **duloxetine:trimipramine** | 0 | Some concerns | Low risk | No concerns | Major concerns | No concerns | No concerns | Very low |
| **duloxetine:venlafaxine** | 0 | Major concerns | Low risk | No concerns | Major concerns | No concerns | No concerns | Very low |
| **duloxetine:vortioxetine** | 0 | Major concerns | Low risk | No concerns | Major concerns | No concerns | No concerns | Very low |
| **escitalopram:fluoxetine** | 0 | Some concerns | Low risk | No concerns | No concerns | Major concerns | No concerns | Very low |
| **escitalopram:imipramine** | 0 | Some concerns | Low risk | No concerns | No concerns | Some concerns | No concerns | Low |
| **escitalopram:lofepramine** | 0 | Major concerns | Low risk | No concerns | Major concerns | No concerns | No concerns | Very low |
| **escitalopram:mianserin** | 0 | Some concerns | Low risk | No concerns | Some concerns | Some concerns | No concerns | Very low |
| **escitalopram:moclobemide** | 0 | Major concerns | Low risk | Some concerns | Major concerns | No concerns | No concerns | Very low |
| **escitalopram:nefazodone** | 0 | Major concerns | Low risk | No concerns | Major concerns | No concerns | No concerns | Very low |
| **escitalopram:nomifensine** | 0 | Some concerns | Low risk | No concerns | Some concerns | No concerns | No concerns | Low |
| **escitalopram:nortriptyline** | 0 | Some concerns | Low risk | No concerns | Some concerns | Some concerns | No concerns | Very low |
| **escitalopram:paroxetine** | 0 | Some concerns | Low risk | No concerns | Some concerns | Some concerns | No concerns | Very low |
| **escitalopram:sertraline** | 0 | Some concerns | Low risk | No concerns | Some concerns | Some concerns | No concerns | Very low |
| **escitalopram:trazodone** | 0 | Major concerns | Low risk | No concerns | Major concerns | No concerns | No concerns | Very low |
| **escitalopram:trimipramine** | 0 | Some concerns | Low risk | Some concerns | Major concerns | No concerns | No concerns | Very low |
| **escitalopram:venlafaxine** | 0 | Some concerns | Low risk | Some concerns | Some concerns | Some concerns | No concerns | Very low |
| **escitalopram:vortioxetine** | 0 | Major concerns | Low risk | No concerns | Major concerns | No concerns | No concerns | Very low |
| **fluoxetine:imipramine** | 0 | Some concerns | Low risk | No concerns | Some concerns | No concerns | No concerns | Low |
| **fluoxetine:lofepramine** | 0 | Major concerns | Low risk | No concerns | Major concerns | No concerns | No concerns | Very low |
| **fluoxetine:mianserin** | 0 | Some concerns | Low risk | No concerns | Some concerns | Some concerns | No concerns | Very low |
| **fluoxetine:mirtazapine** | 0 | Major concerns | Low risk | No concerns | Some concerns | Some concerns | No concerns | Very low |
| **fluoxetine:moclobemide** | 0 | Major concerns | Low risk | Some concerns | Major concerns | No concerns | No concerns | Very low |
| **fluoxetine:nomifensine** | 0 | Some concerns | Low risk | No concerns | Some concerns | No concerns | No concerns | Low |
| **fluoxetine:nortriptyline** | 0 | Some concerns | Low risk | No concerns | Some concerns | Some concerns | No concerns | Very low |
| **fluoxetine:sertraline** | 0 | Some concerns | Low risk | No concerns | No concerns | Major concerns | No concerns | Very low |
| **fluoxetine:trimipramine** | 0 | Some concerns | Low risk | Some concerns | Major concerns | No concerns | No concerns | Very low |
| **fluoxetine:vortioxetine** | 0 | Major concerns | Low risk | No concerns | Major concerns | No concerns | No concerns | Very low |
| **imipramine:lofepramine** | 0 | Major concerns | Low risk | No concerns | Some concerns | No concerns | No concerns | Very low |
| **imipramine:mianserin** | 0 | Some concerns | Low risk | No concerns | Major concerns | No concerns | No concerns | Very low |
| **imipramine:mirtazapine** | 0 | Some concerns | Low risk | No concerns | Major concerns | No concerns | No concerns | Very low |
| **imipramine:moclobemide** | 0 | Major concerns | Low risk | No concerns | Major concerns | No concerns | No concerns | Very low |
| **imipramine:nefazodone** | 0 | Major concerns | Low risk | No concerns | Major concerns | No concerns | No concerns | Very low |
| **imipramine:nomifensine** | 0 | Some concerns | Low risk | No concerns | No concerns | No concerns | No concerns | Moderate |
| **imipramine:nortriptyline** | 0 | Some concerns | Low risk | No concerns | Some concerns | Some concerns | No concerns | Very low |
| **imipramine:sertraline** | 0 | Some concerns | Low risk | No concerns | Some concerns | Some concerns | No concerns | Very low |
| **imipramine:trazodone** | 0 | Major concerns | Low risk | No concerns | Some concerns | Some concerns | No concerns | Very low |
| **imipramine:trimipramine** | 0 | Some concerns | Low risk | No concerns | Major concerns | No concerns | No concerns | Very low |
| **imipramine:venlafaxine** | 0 | Major concerns | Low risk | No concerns | No concerns | Some concerns | No concerns | Very low |
| **imipramine:vortioxetine** | 0 | Major concerns | Low risk | No concerns | Major concerns | No concerns | No concerns | Very low |
| **lofepramine:mianserin** | 0 | Major concerns | Low risk | No concerns | Some concerns | Some concerns | No concerns | Very low |
| **lofepramine:mirtazapine** | 0 | Major concerns | Low risk | No concerns | Some concerns | Some concerns | No concerns | Very low |
| **lofepramine:moclobemide** | 0 | Major concerns | Low risk | No concerns | Major concerns | No concerns | No concerns | Very low |
| **lofepramine:nefazodone** | 0 | Major concerns | Low risk | No concerns | Major concerns | No concerns | No concerns | Very low |
| **lofepramine:nomifensine** | 0 | Some concerns | Low risk | No concerns | Major concerns | No concerns | No concerns | Very low |
| **lofepramine:nortriptyline** | 0 | Major concerns | Low risk | No concerns | Some concerns | Some concerns | No concerns | Very low |
| **lofepramine:paroxetine** | 0 | Major concerns | Low risk | No concerns | Some concerns | Some concerns | No concerns | Very low |
| **lofepramine:sertraline** | 0 | Major concerns | Low risk | No concerns | Some concerns | Some concerns | No concerns | Very low |
| **lofepramine:trazodone** | 0 | Major concerns | Low risk | No concerns | Major concerns | No concerns | No concerns | Very low |
| **lofepramine:trimipramine** | 0 | Some concerns | Low risk | No concerns | Major concerns | No concerns | No concerns | Very low |
| **lofepramine:venlafaxine** | 0 | Major concerns | Low risk | No concerns | Major concerns | No concerns | No concerns | Very low |
| **lofepramine:vortioxetine** | 0 | Major concerns | Low risk | No concerns | Major concerns | No concerns | No concerns | Very low |
| **mianserin:mirtazapine** | 0 | Major concerns | Low risk | No concerns | Major concerns | No concerns | No concerns | Very low |
| **mianserin:moclobemide** | 0 | Major concerns | Low risk | No concerns | Major concerns | No concerns | No concerns | Very low |
| **mianserin:nefazodone** | 0 | Major concerns | Low risk | No concerns | Major concerns | No concerns | No concerns | Very low |
| **mianserin:nomifensine** | 0 | Some concerns | Low risk | No concerns | No concerns | Some concerns | No concerns | Low |
| **mianserin:nortriptyline** | 0 | Some concerns | Low risk | No concerns | Major concerns | No concerns | No concerns | Very low |
| **mianserin:paroxetine** | 0 | Some concerns | Low risk | No concerns | Some concerns | Some concerns | No concerns | Very low |
| **mianserin:sertraline** | 0 | Some concerns | Low risk | No concerns | Some concerns | Some concerns | No concerns | Very low |
| **mianserin:trazodone** | 0 | Major concerns | Low risk | No concerns | Major concerns | No concerns | No concerns | Very low |
| **mianserin:trimipramine** | 0 | Some concerns | Low risk | No concerns | Major concerns | No concerns | No concerns | Very low |
| **mianserin:venlafaxine** | 0 | Major concerns | Low risk | No concerns | Some concerns | Some concerns | No concerns | Very low |
| **mianserin:vortioxetine** | 0 | Major concerns | Low risk | No concerns | Major concerns | No concerns | No concerns | Very low |
| **mirtazapine:moclobemide** | 0 | Major concerns | Low risk | No concerns | Major concerns | No concerns | No concerns | Very low |
| **mirtazapine:nefazodone** | 0 | Major concerns | Low risk | No concerns | Major concerns | No concerns | No concerns | Very low |
| **mirtazapine:nomifensine** | 0 | Some concerns | Low risk | No concerns | No concerns | Some concerns | No concerns | Low |
| **mirtazapine:nortriptyline** | 0 | Some concerns | Low risk | No concerns | Major concerns | No concerns | No concerns | Very low |
| **mirtazapine:paroxetine** | 0 | Major concerns | Low risk | No concerns | Some concerns | Some concerns | No concerns | Very low |
| **mirtazapine:sertraline** | 0 | Some concerns | Low risk | No concerns | Some concerns | Some concerns | No concerns | Very low |
| **mirtazapine:trazodone** | 0 | Major concerns | Low risk | No concerns | Major concerns | No concerns | No concerns | Very low |
| **mirtazapine:trimipramine** | 0 | Some concerns | Low risk | No concerns | Major concerns | No concerns | No concerns | Very low |
| **mirtazapine:venlafaxine** | 0 | Major concerns | Low risk | No concerns | Some concerns | Some concerns | No concerns | Very low |
| **mirtazapine:vortioxetine** | 0 | Major concerns | Low risk | No concerns | Major concerns | No concerns | No concerns | Very low |
| **moclobemide:nefazodone** | 0 | Major concerns | Low risk | No concerns | Major concerns | No concerns | No concerns | Very low |
| **moclobemide:nomifensine** | 0 | Some concerns | Low risk | No concerns | Some concerns | No concerns | No concerns | Low |
| **moclobemide:nortriptyline** | 0 | Major concerns | Low risk | No concerns | Major concerns | No concerns | No concerns | Very low |
| **moclobemide:paroxetine** | 0 | Major concerns | Low risk | Some concerns | Major concerns | No concerns | No concerns | Very low |
| **moclobemide:sertraline** | 0 | Major concerns | Low risk | Some concerns | Major concerns | No concerns | No concerns | Very low |
| **moclobemide:trazodone** | 0 | Major concerns | Low risk | Some concerns | Major concerns | No concerns | No concerns | Very low |
| **moclobemide:trimipramine** | 0 | Some concerns | Low risk | Some concerns | Major concerns | No concerns | No concerns | Very low |
| **moclobemide:venlafaxine** | 0 | Major concerns | Low risk | Some concerns | Major concerns | No concerns | No concerns | Very low |
| **moclobemide:vortioxetine** | 0 | Major concerns | Low risk | Some concerns | Major concerns | No concerns | No concerns | Very low |
| **nefazodone:nomifensine** | 0 | Some concerns | Low risk | No concerns | Major concerns | No concerns | No concerns | Very low |
| **nefazodone:nortriptyline** | 0 | Major concerns | Low risk | No concerns | Major concerns | No concerns | No concerns | Very low |
| **nefazodone:paroxetine** | 0 | Major concerns | Low risk | No concerns | Major concerns | No concerns | No concerns | Very low |
| **nefazodone:placebo** | 0 | Major concerns | Low risk | No concerns | Major concerns | No concerns | No concerns | Very low |
| **nefazodone:sertraline** | 0 | Major concerns | Low risk | No concerns | Major concerns | No concerns | No concerns | Very low |
| **nefazodone:trazodone** | 0 | Major concerns | Low risk | No concerns | Major concerns | No concerns | No concerns | Very low |
| **nefazodone:trimipramine** | 0 | Some concerns | Low risk | No concerns | Major concerns | No concerns | No concerns | Very low |
| **nefazodone:venlafaxine** | 0 | Major concerns | Low risk | No concerns | Major concerns | No concerns | No concerns | Very low |
| **nefazodone:vortioxetine** | 0 | Major concerns | Low risk | No concerns | Major concerns | No concerns | No concerns | Very low |
| **nomifensine:nortriptyline** | 0 | Some concerns | Low risk | No concerns | No concerns | Some concerns | No concerns | Low |
| **nomifensine:paroxetine** | 0 | Some concerns | Low risk | No concerns | No concerns | Some concerns | No concerns | Low |
| **nomifensine:sertraline** | 0 | Some concerns | Low risk | No concerns | No concerns | Some concerns | No concerns | Low |
| **nomifensine:trazodone** | 0 | Some concerns | Low risk | No concerns | Some concerns | No concerns | No concerns | Low |
| **nomifensine:trimipramine** | 0 | Some concerns | Low risk | No concerns | Major concerns | No concerns | No concerns | Very low |
| **nomifensine:venlafaxine** | 0 | Some concerns | Low risk | No concerns | Some concerns | Some concerns | No concerns | Very low |
| **nomifensine:vortioxetine** | 0 | Some concerns | Low risk | No concerns | Major concerns | No concerns | No concerns | Very low |
| **nortriptyline:sertraline** | 0 | Some concerns | Low risk | No concerns | Some concerns | Some concerns | No concerns | Very low |
| **nortriptyline:trazodone** | 0 | Major concerns | Low risk | No concerns | Major concerns | No concerns | No concerns | Very low |
| **nortriptyline:trimipramine** | 0 | Some concerns | Low risk | No concerns | Major concerns | No concerns | No concerns | Very low |
| **nortriptyline:venlafaxine** | 0 | Some concerns | Low risk | No concerns | Some concerns | Some concerns | No concerns | Very low |
| **nortriptyline:vortioxetine** | 0 | Major concerns | Low risk | No concerns | Major concerns | No concerns | No concerns | Very low |
| **paroxetine:sertraline** | 0 | Some concerns | Low risk | No concerns | No concerns | Major concerns | No concerns | Very low |
| **paroxetine:trazodone** | 0 | Major concerns | Low risk | No concerns | Major concerns | No concerns | No concerns | Very low |
| **paroxetine:trimipramine** | 0 | Some concerns | Low risk | Some concerns | Major concerns | No concerns | No concerns | Very low |
| **paroxetine:vortioxetine** | 0 | Major concerns | Low risk | No concerns | Major concerns | No concerns | No concerns | Very low |
| **placebo:vortioxetine** | 0 | Major concerns | Low risk | No concerns | Major concerns | No concerns | No concerns | Very low |
| **sertraline:trazodone** | 0 | No concerns | Low risk | Some concerns | Major concerns | No concerns | No concerns | Very low |
| **sertraline:trimipramine** | 0 | Some concerns | Low risk | Some concerns | Major concerns | No concerns | No concerns | Very low |
| **trazodone:trimipramine** | 0 | Some concerns | Low risk | Some concerns | Major concerns | No concerns | No concerns | Very low |
| **trazodone:venlafaxine** | 0 | Major concerns | Low risk | Some concerns | Some concerns | Some concerns | No concerns | Very low |
| **trazodone:vortioxetine** | 0 | Major concerns | Low risk | No concerns | Major concerns | No concerns | No concerns | Very low |
| **trimipramine:venlafaxine** | 0 | Some concerns | Low risk | Some concerns | Major concerns | No concerns | No concerns | Very low |
| **trimipramine:vortioxetine** | 0 | Some concerns | Low risk | Some concerns | Major concerns | No concerns | No concerns | Very low |
| **venlafaxine:vortioxetine** | 0 | Major concerns | Low risk | No concerns | Major concerns | No concerns | No concerns | Very low |

**Sensitivity analyses (efficacy)**

| **Analysis** | **Network characteristics and Network map** | **Forest plot** | **Heterogeneity** | **Inconsistency** |
| --- | --- | --- | --- | --- |
| Excluding RCTs that were not blind | N=97  n=7345 |  | tau^2= 0.142  I^2= 67.58 % (59.27 % to 74.2 %) | Global approach:  p=0.8751  Local approach (SIDE): 1/31 |
| Excluding RCTs with follow-up <3 months | N=101  n=7636   |  | tau^2=0.1634  I^2=70.87 % (63.82 % to 76.54 %) | Global approach:  p=0.3099  Local approach (SIDE): 2/38 |
| Excluding RCTs with an overall high risk of bias | N=71  n=5845  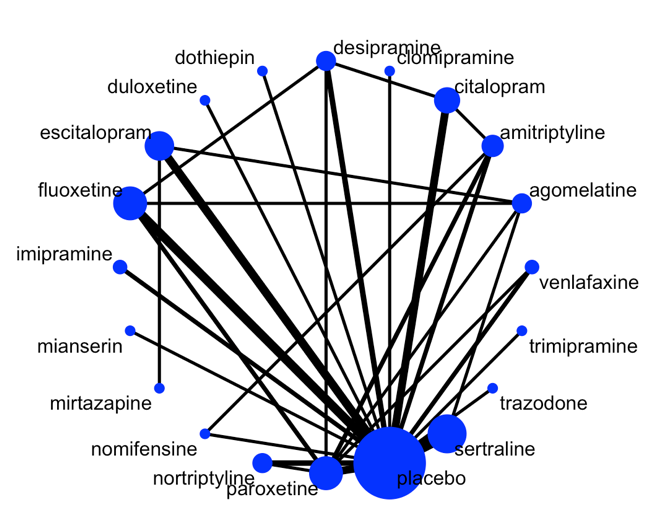 | 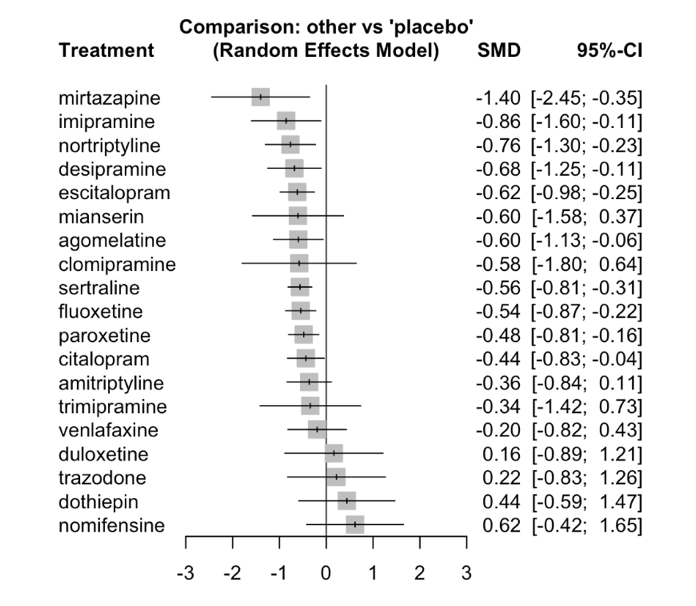 | τ^2^=0.0369  I^2^=75.97%  (65.82 % to 86.12 %) | Global:  P=0.2708  Local (SIDE):  2/24 |
| Only RCTs with a formal diagnosis of MDD (post-hoc) | N=89  n=6970   |  | tau^2=0.0892  I^2=55.85 % (42.8 % to 65.93 %) | Global approach:  p=0.0592  Local approach (SIDE): 1/36 |
| Excluding RCTs with a sample size < 50 (post-hoc) | N=33  n=2762   |  | tau^2=0.0989  I^2=58.34 % (32.48 % to 74.3 %) | Global approach:  p=0.6395  Local approach (SIDE): 1/18 |
| Excluding RCTs where efficacy on depressive symptoms was not the primary study aim | N=88  n=6481   |  | tau^2=0.1361  I^2= 65.42 % (55.76 % to 72.97 %) | Global approach:  p=0.0918  Local approach (SIDE): 1/34 |
| Excluding placebo-controlled trials | N=22  n=1320   |  | tau^2=0.0172  I^2=18.62 % (0 % to 57.72 %) | Global approach:  p=0.2098  Local approach (SIDE): 0/12 |
| Excluding trials recruiting individuals with minor and minor-to-moderate severity of illness | N=66  n=6003  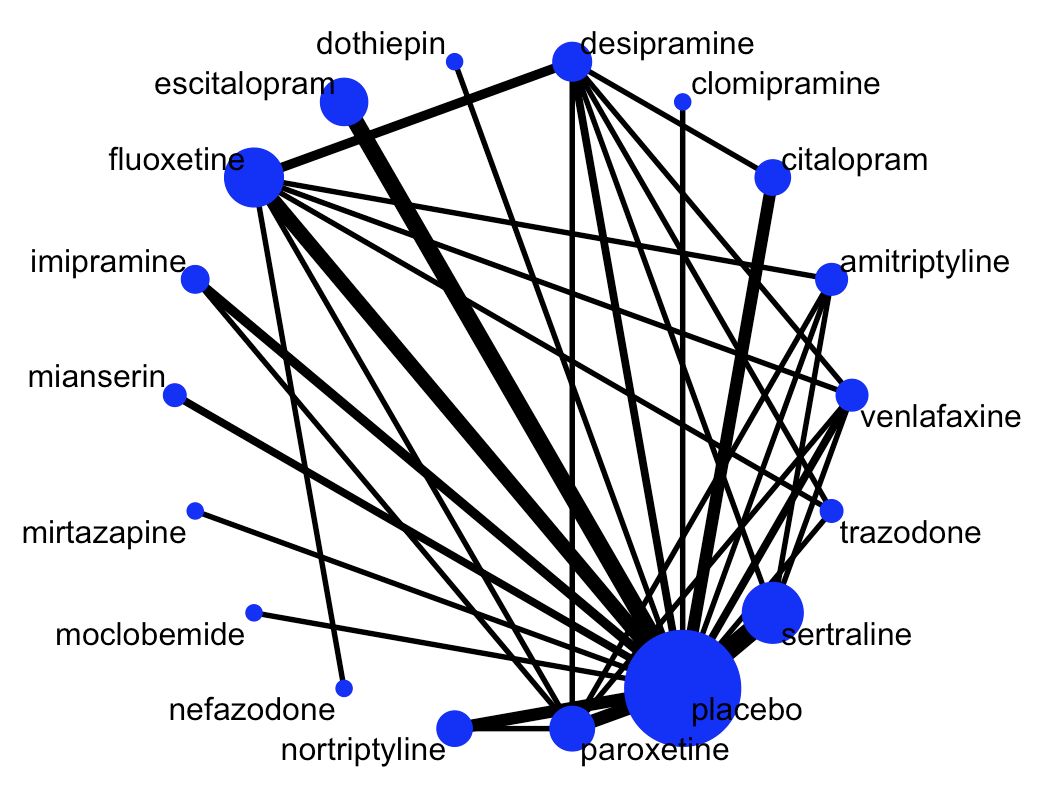 | 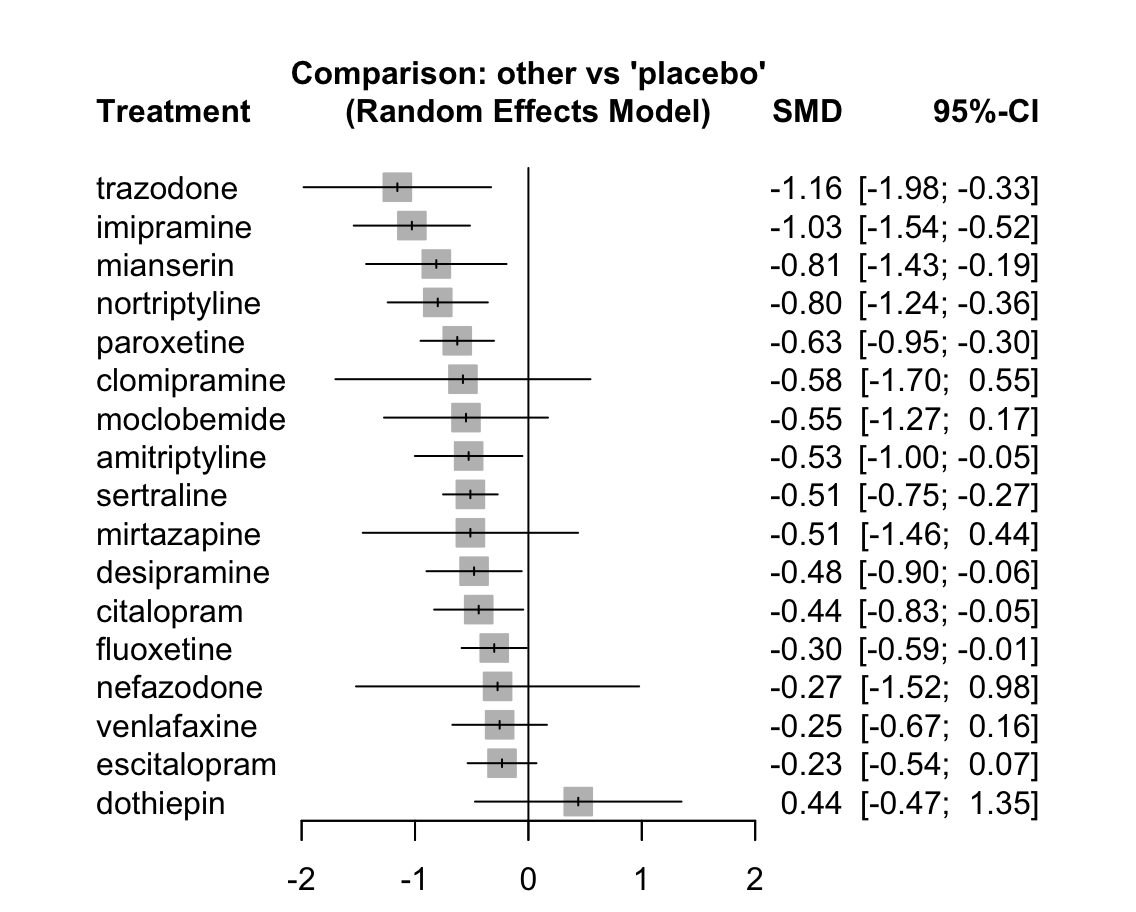 | tau^2=0.1304  I^2= 66.04 % (55.06 % to 74.33 %) | Global approach:  p=0.7400  Local approach (SIDE): 0/26 |

**Subgroup analyses (efficacy)**

| **Analysis** | **Network characteristics and Network map** | **Forest plot** | **Heterogeneity** | **Inconsistency** |
| --- | --- | --- | --- | --- |
| Circulatory System Diseases | N=16, n=2075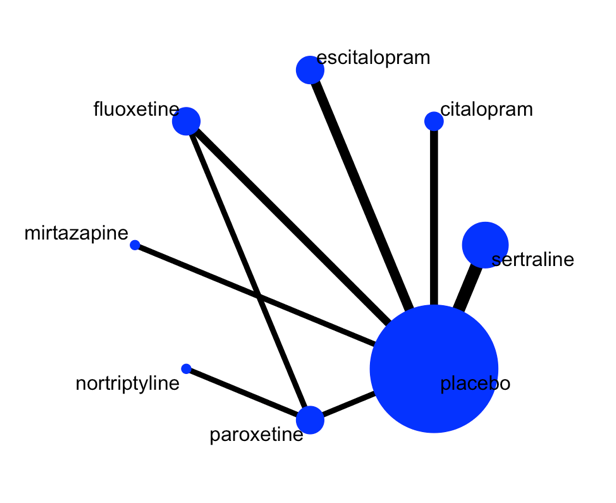 | 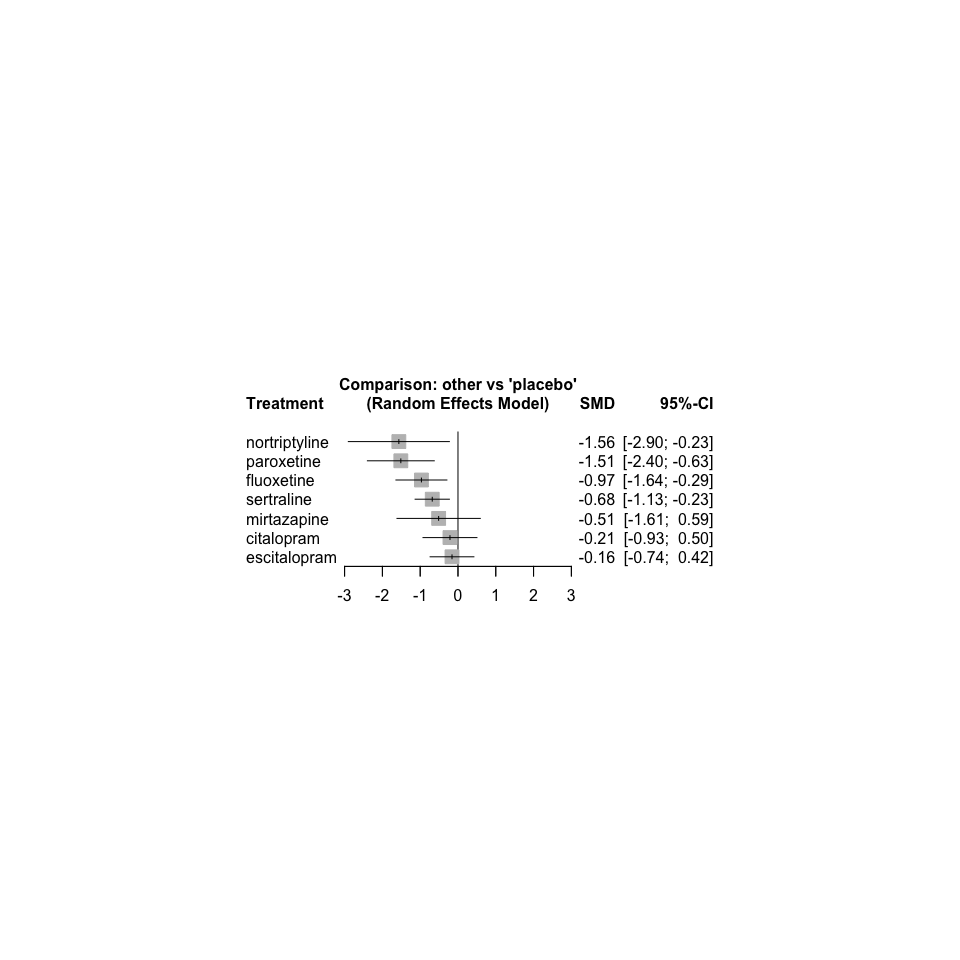 | τ^2^=0.2121  I^2^=85.46% | Global approach:  p=0.1210  Local approach (SIDE):  0/3 |
| Endocrine, Nutritional, Metabolic Diseases | 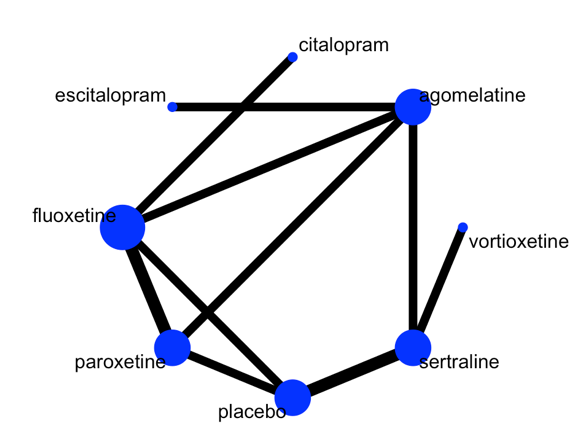N=12, n=643 | 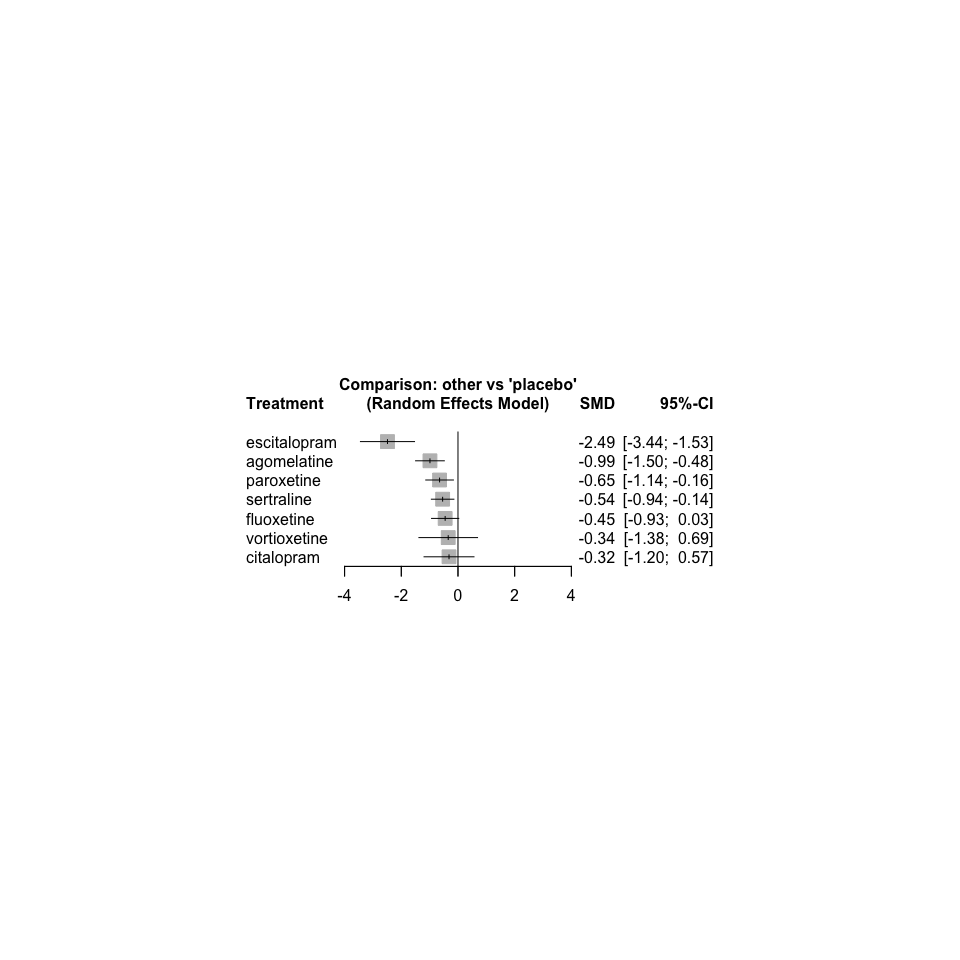 | τ^2^=0.042  I^2^=34.39% | Global approach:  p=0.7879  Local approach (SIDE):  0/7 |
| Infectious Diseases | 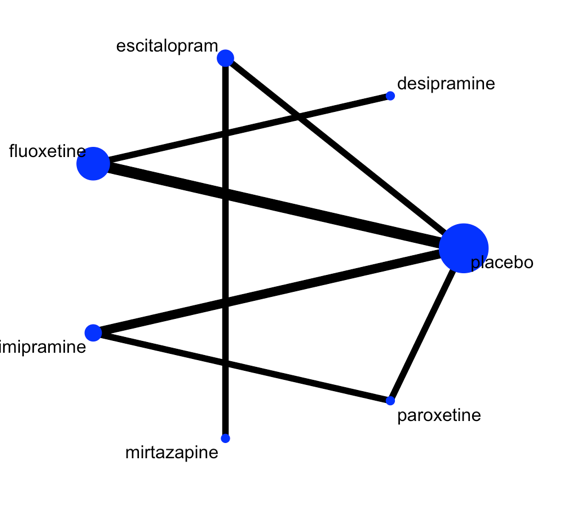N=8. N=491 | 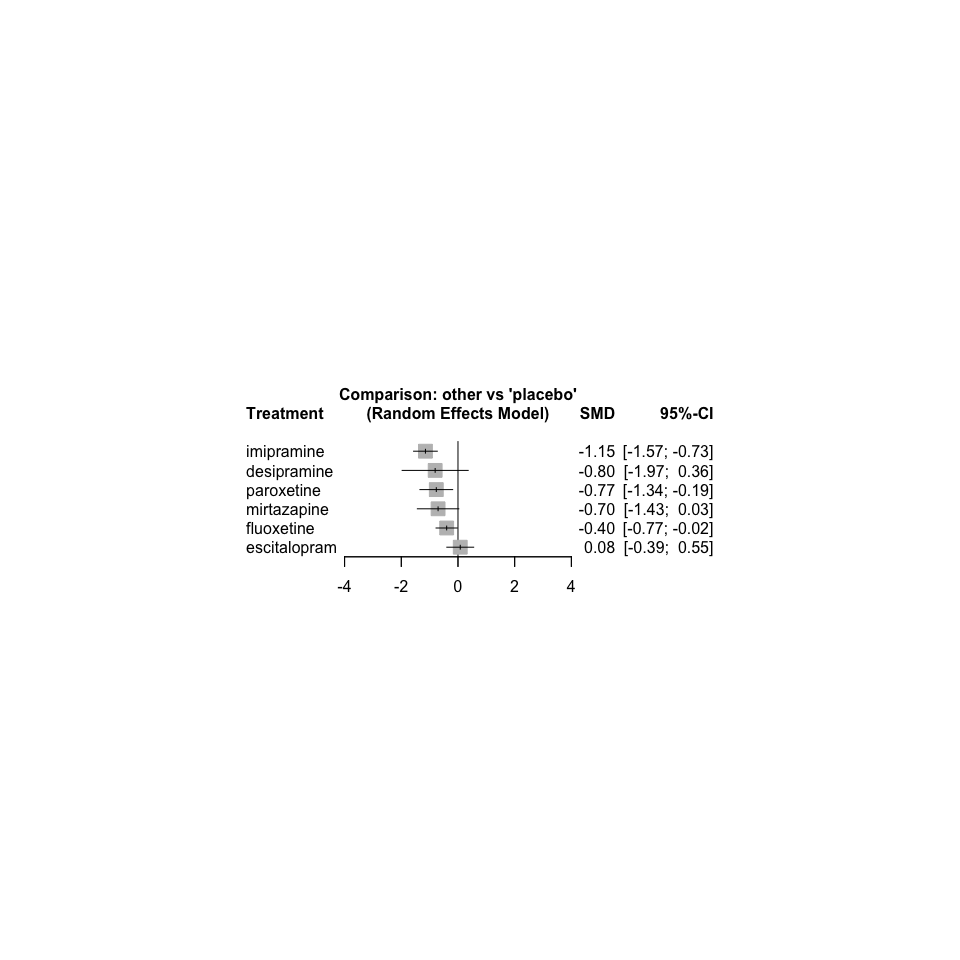 | τ^2^=0.0193  I^2^=17.63% | Global approach:  p=0.3096  Local approach (SIDE):  0/2 |
| Neurological Diseases | 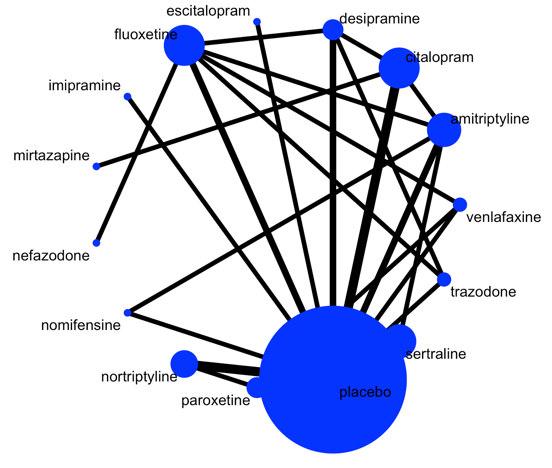N=29, n=1300 | 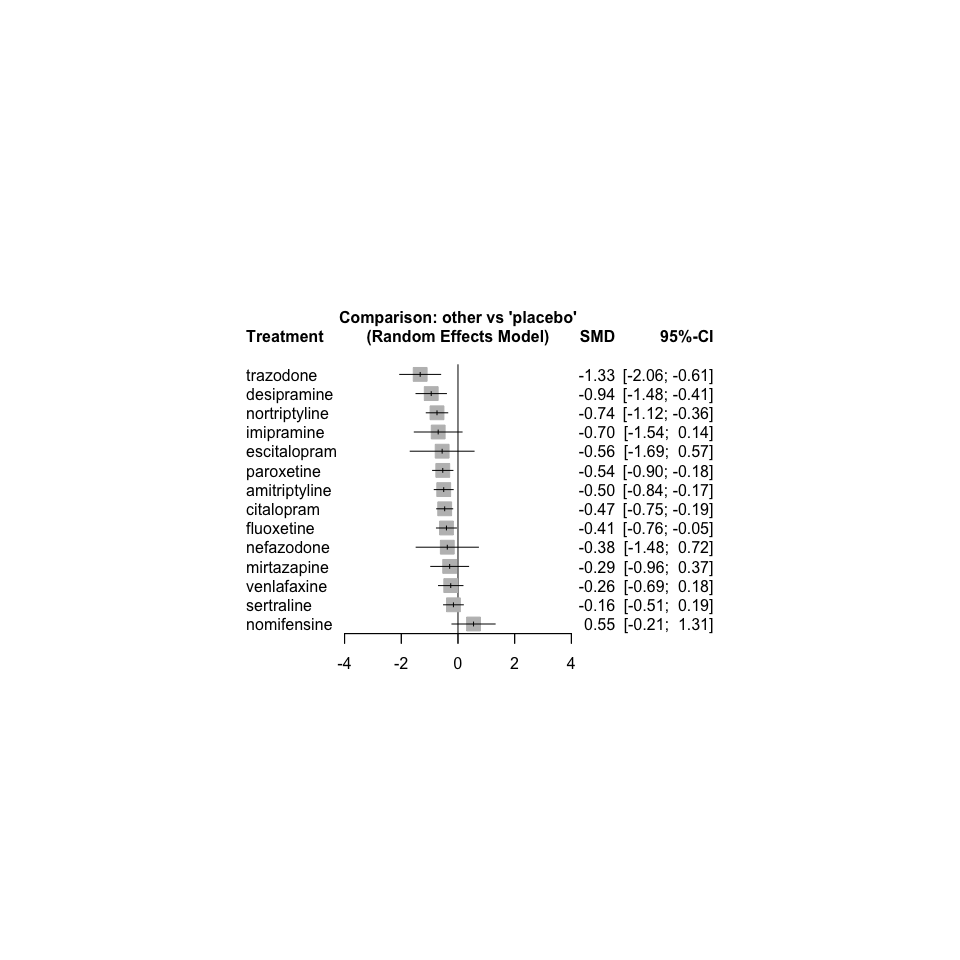 | τ^2^=0.0276  I^2^=20.37% | Global approach:  p=0.6636  Local approach (SIDE):  0/21 |
| Neurocognitive Disorders | 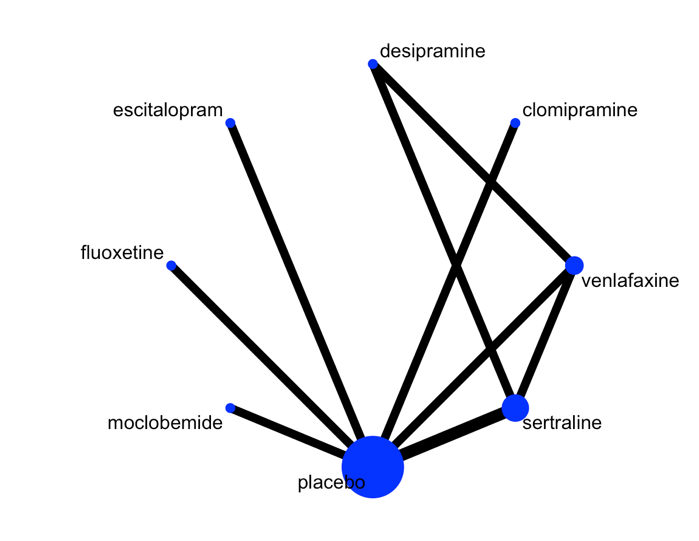N=9, n=1126 | 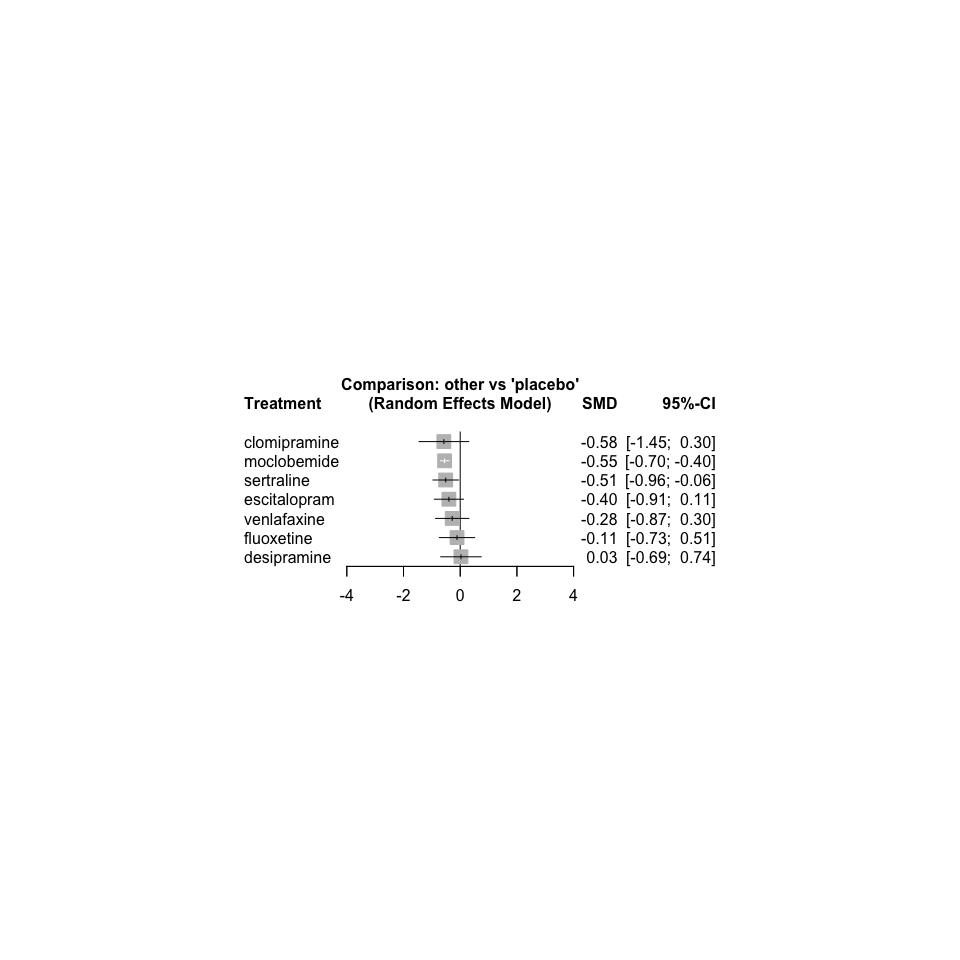 | τ^2^=0  I^2^=0% | Global approach:  p=0.5937  Local approach (SIDE):  0/5 |
| Oncological Diseases | 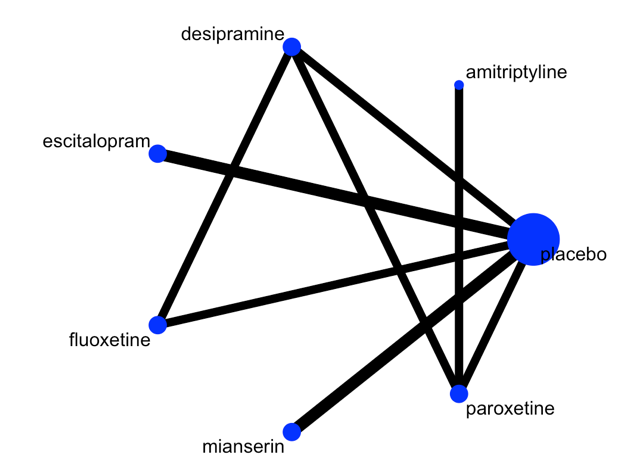N=8, n=602 | 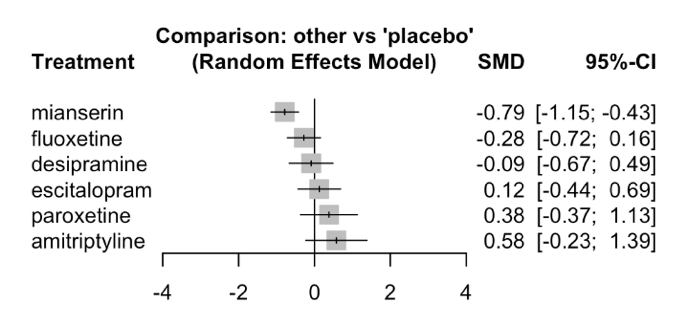 | τ^2^=0  I^2^=0% | Global approach:  p=0..4121  Local approach (SIDE):  0 |
| Renal Diseases | 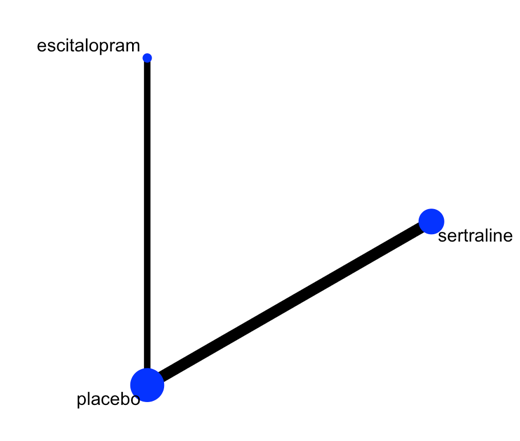N=4, n=315 | 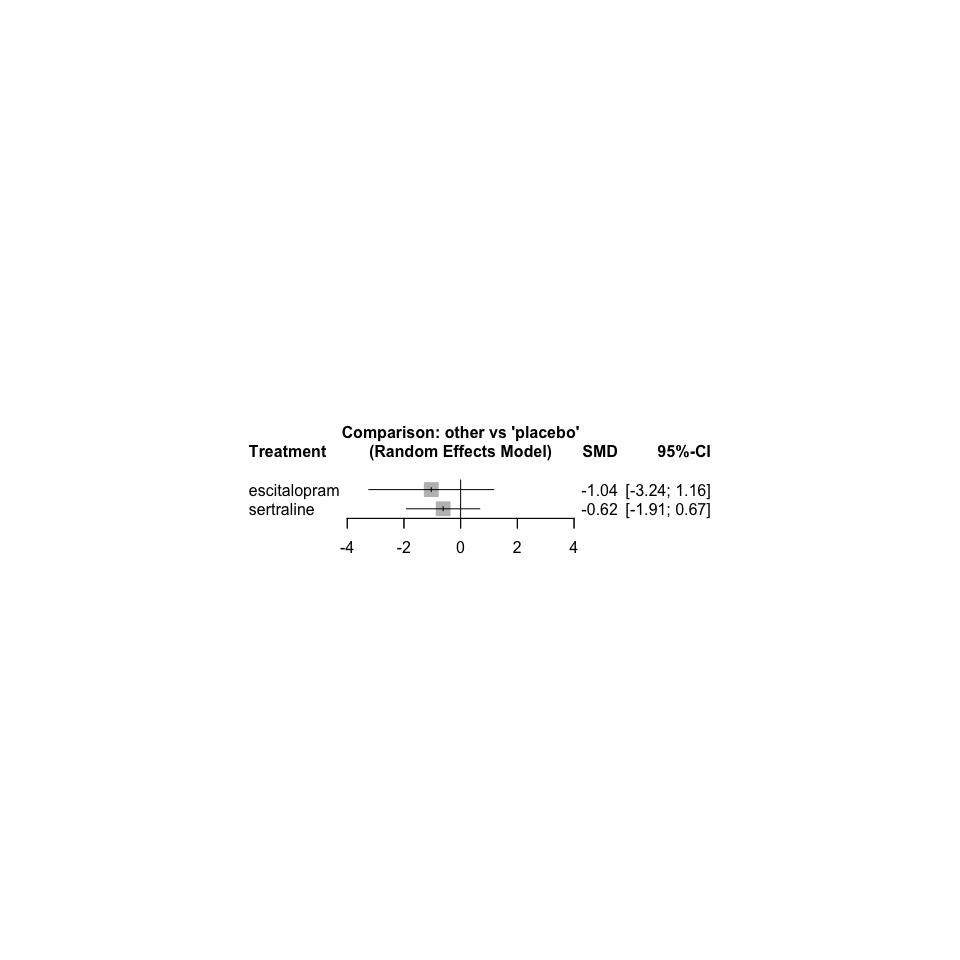 | τ^2^=1.1832  I^2^=92.41% | Global approach:  NA  Local approach (SIDE): NA |
| Respiratory Diseases | 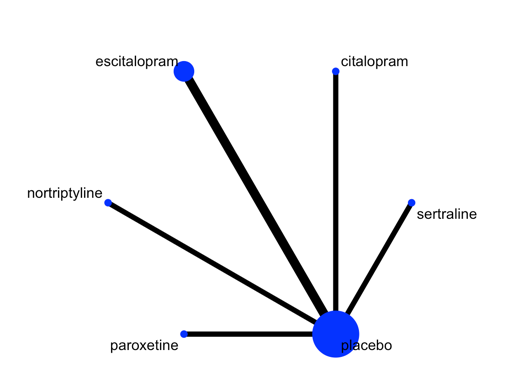N=7, n=396 | 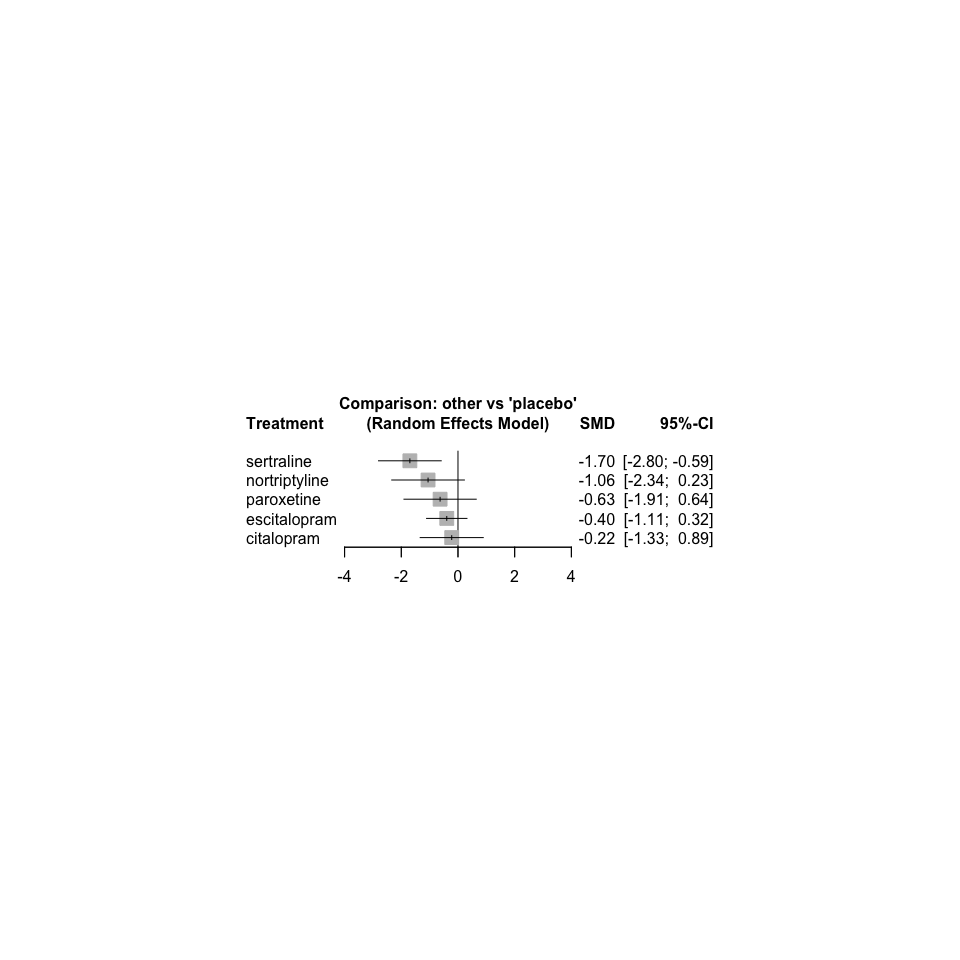 | τ^2^=0.2732  I^2^=69.57% | Global approach:  NA  Local approach (SIDE):  NA |
| Hiv/Aids | 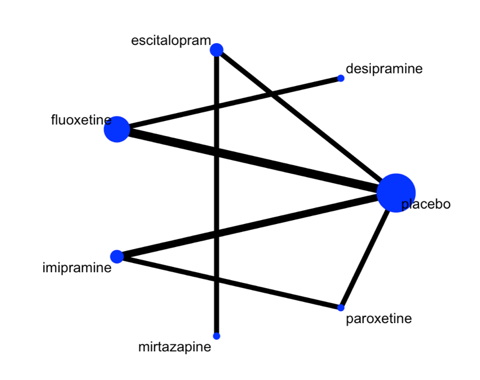N=9, n=566 | 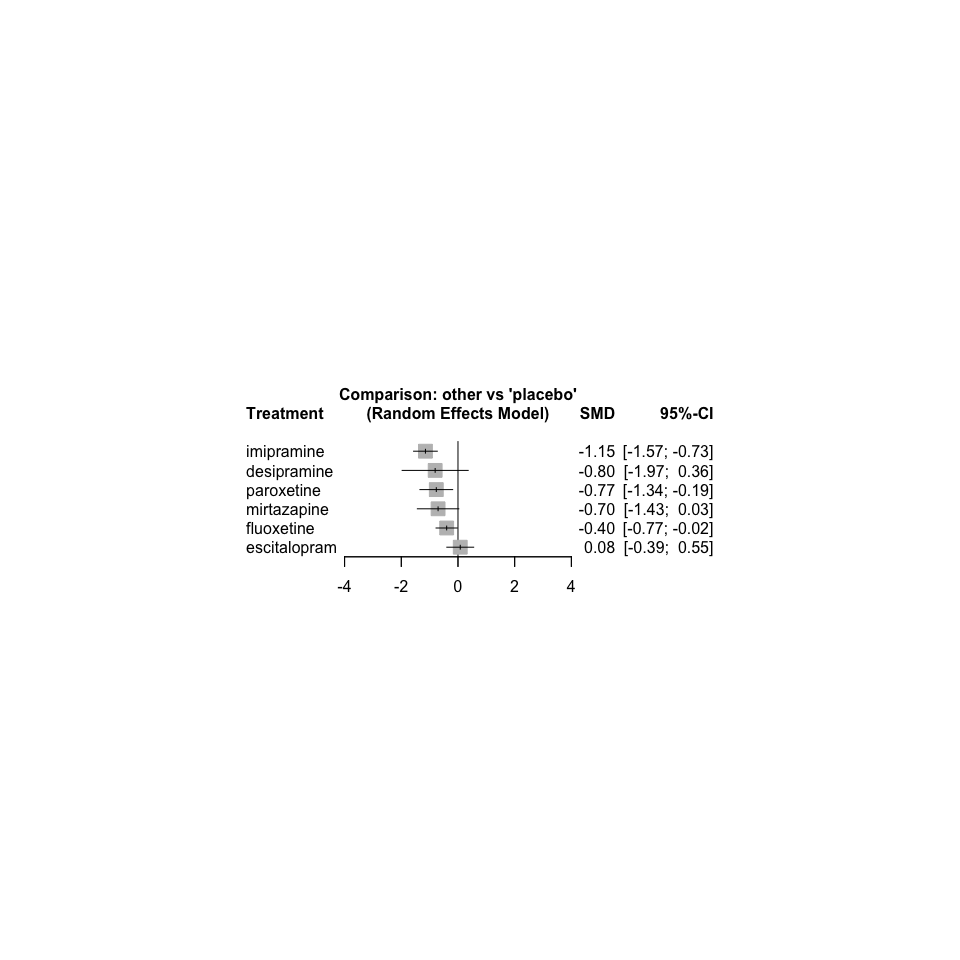 | τ^2^=0.0193  I^2^=17.63% | Global approach:  P=0.3096  Local approach (SIDE):  0/2 |
| Diabetes | 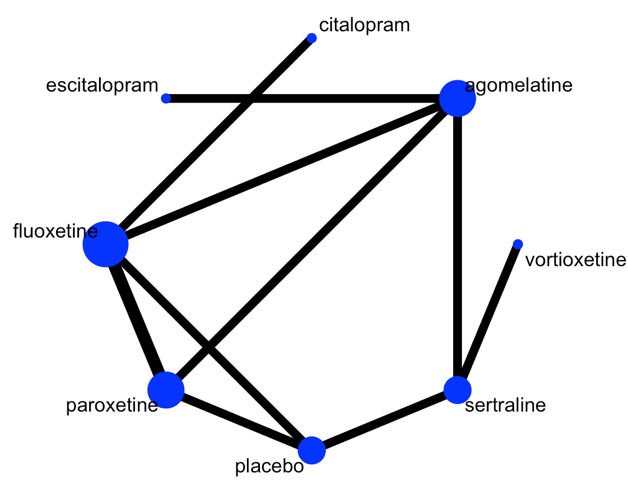N=11, n=579 | 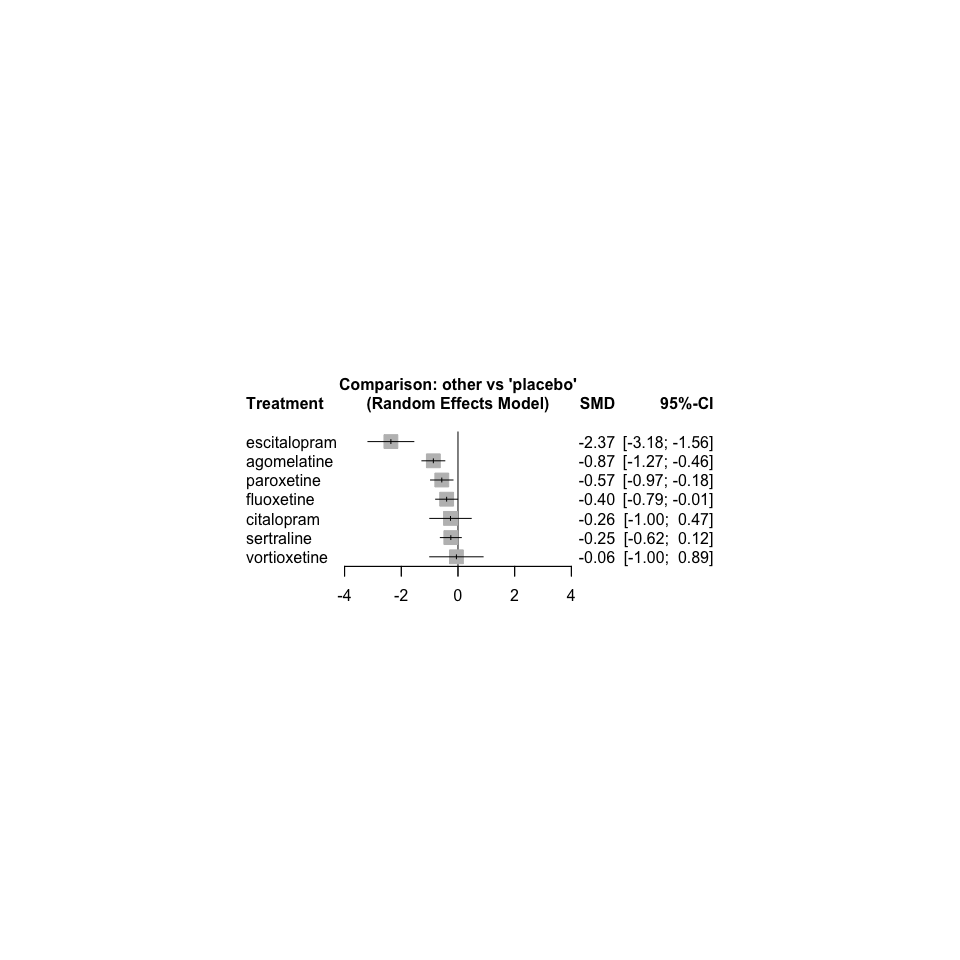 | τ^2^=0  I^2^=0% | Global approach:  P=0.6865  Local approach (SIDE):  0/7 |
| Parkinson  Disease | 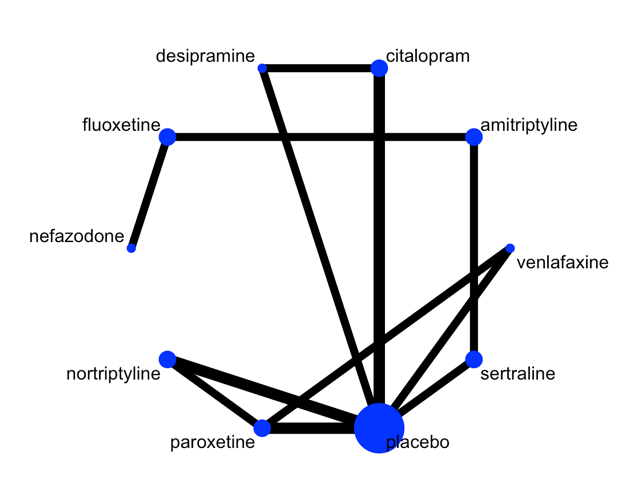N=9, n=362 | 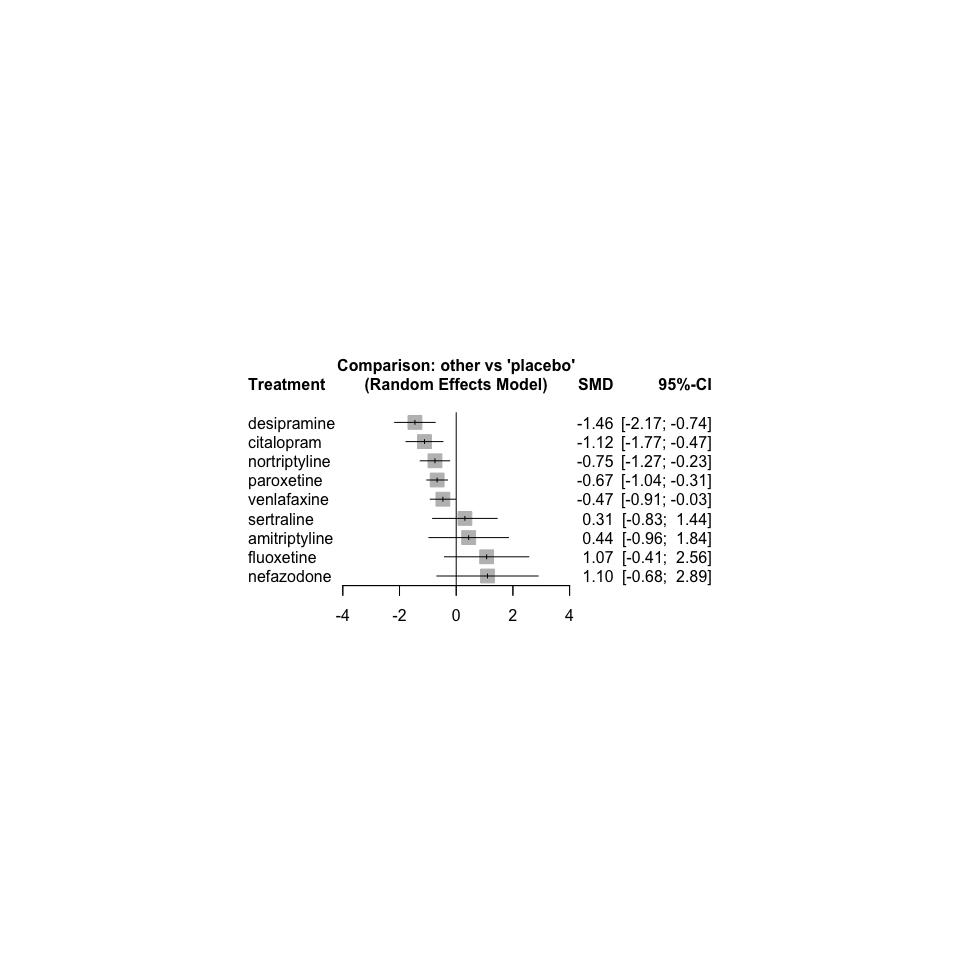 | τ^2^=0  I^2^=0% | Global approach:  P=0.7442  Local approach (SIDE):  0/7 |
| Post-Stroke Depression | 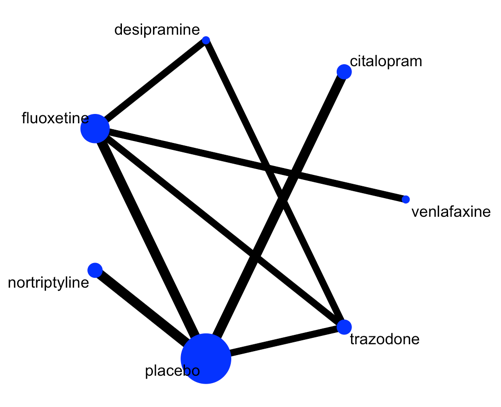N=9, n=494 | 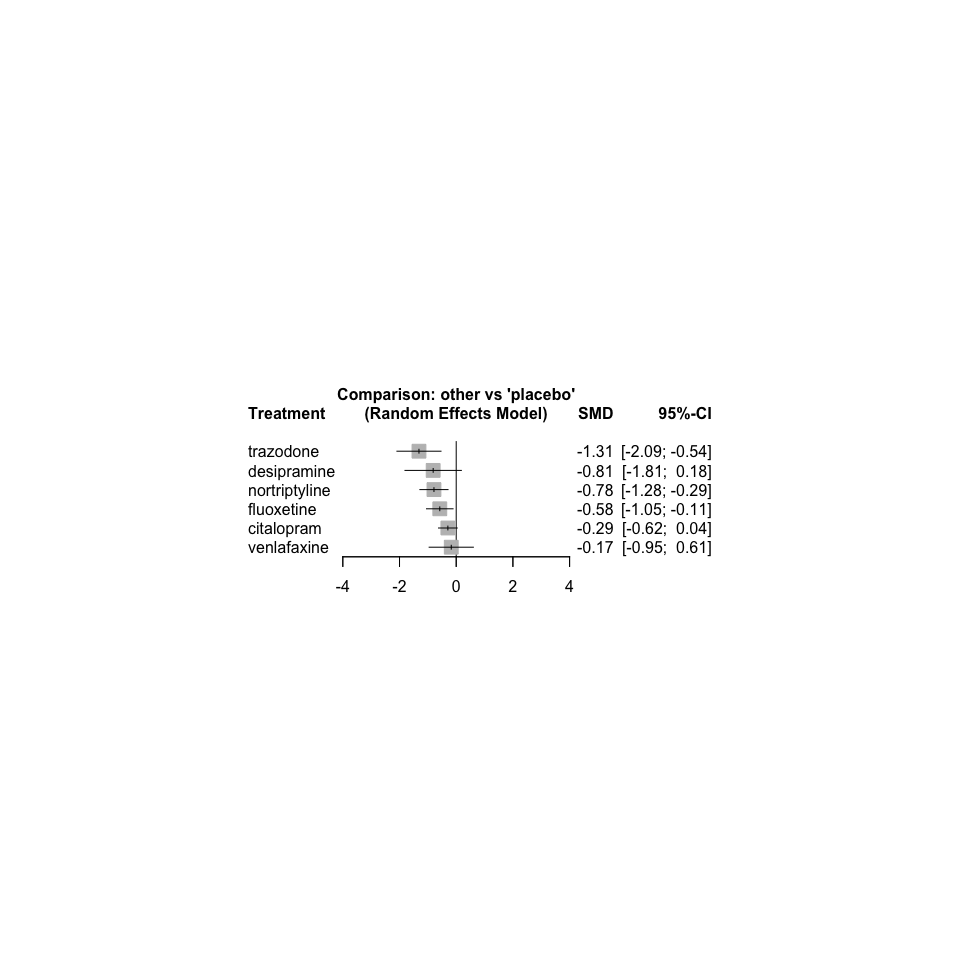 | τ^2^=0.0197  I^2^=18.04% | Global approach:  P=0.8976  Local approach (SIDE):  0/5 |
| Traumatic Brain Injury | 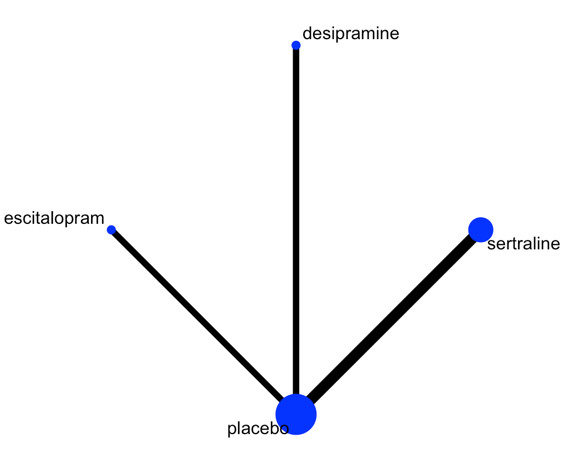N=5, n=155 | 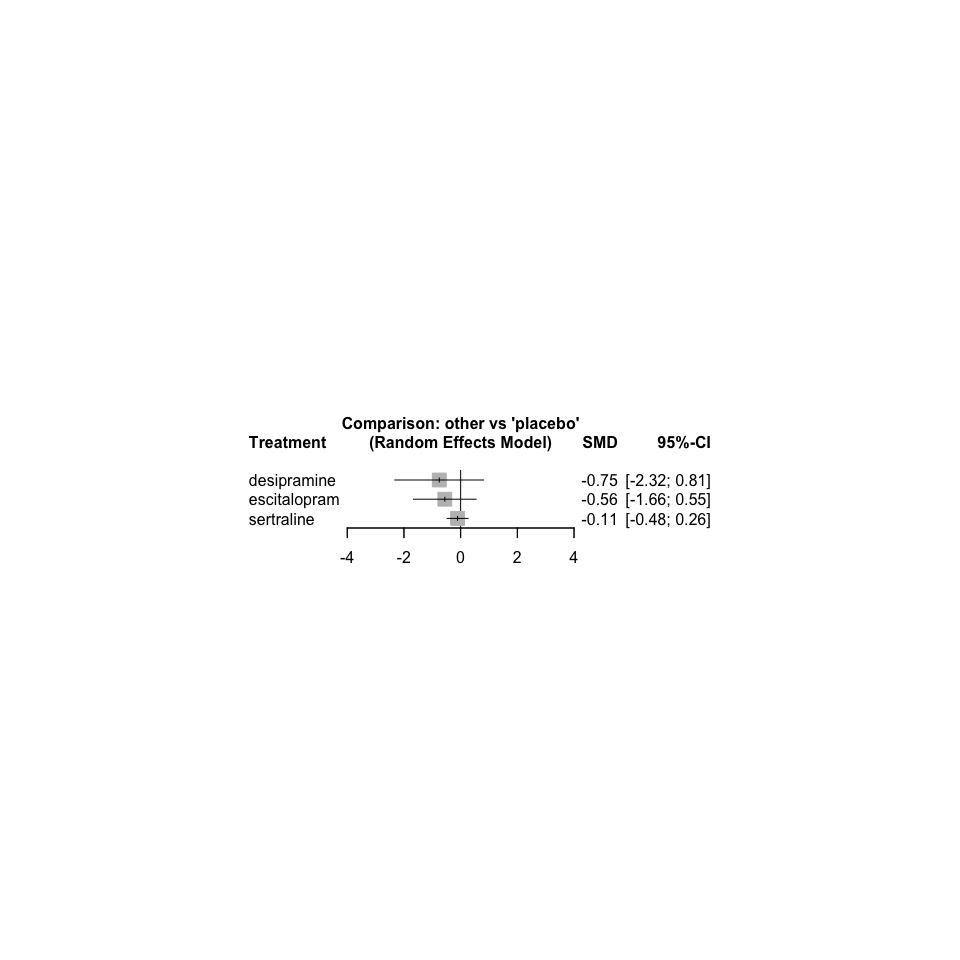 | τ^2^=0.0149  I^2^=13.15% | Global approach:  NA  Local approach (SIDE):  NA |
| Chronic Heart Failure Disease | 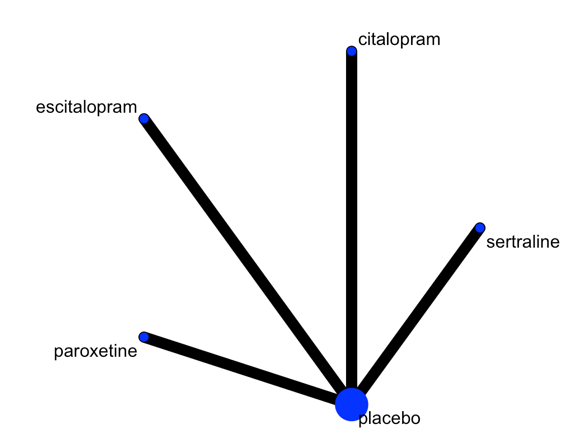N=5, n=691 | 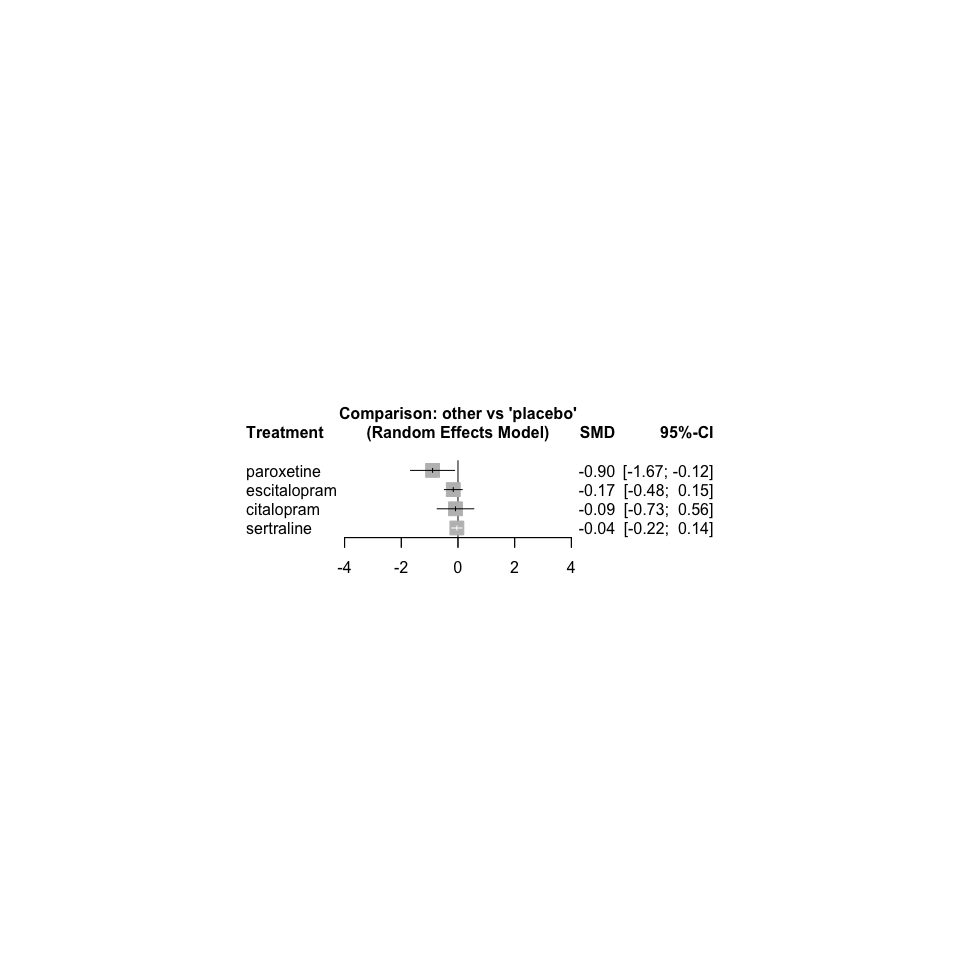 | τ^2^=NA  I^2^=NA | Global approach:  NA  Local approach (SIDE):  NA |
| Post Myocardial Infarction | 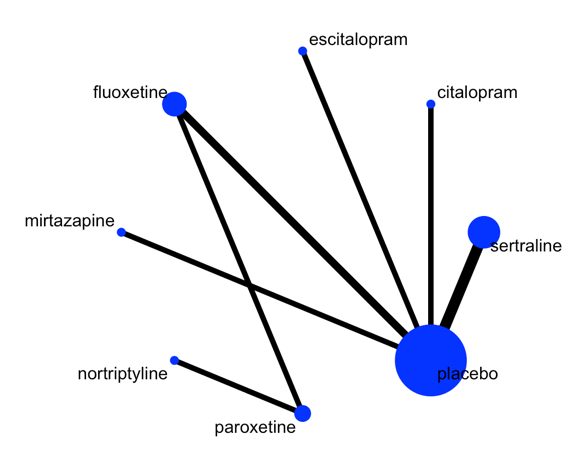N=11, n=1354 | 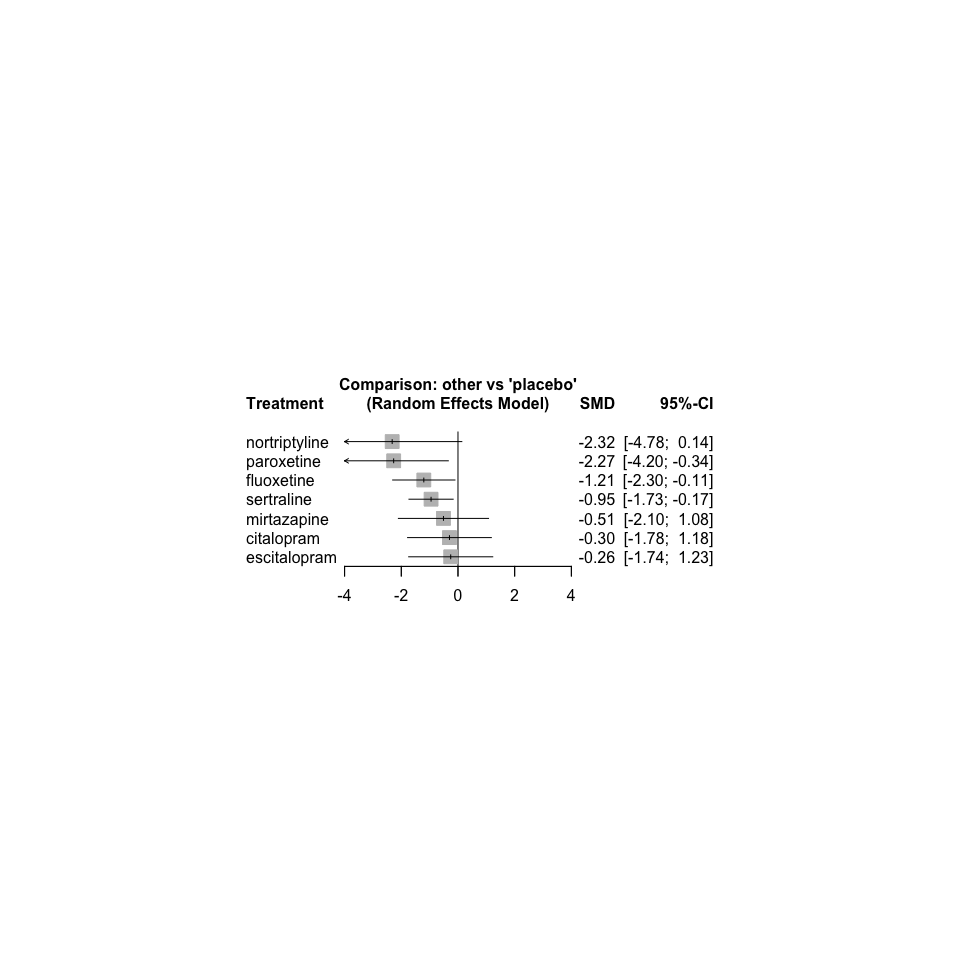 | τ^2^=0.5541  I^2^=91.11% | Global approach:  NA  Local approach (SIDE):  NA |

**H. - Primary outcome: tolerability**

**Characteristics of the network**

Number of treatments:

20

Number of studies:

82

Number of individuals randomized:

6083

Number of individuals randomized to each treatment:

Treatment name N. individuals randomized
1 agomelatine 124
2 amitriptyline 313
3 citalopram 395
4 clomipramine 12
5 desipramine 107
6 doxepin 48
7 escitalopram 284
8 fluoxetine 373
9 imipramine 89
10 mianserin 101
11 mirtazapine 27
12 nefazodone 9
13 nomifensine 13
14 nortriptyline 139
15 paroxetine 533
16 placebo 2325
17 sertraline 1077
18 trazodone 28
19 trimipramine 18
20 venlafaxine 68

**Pairwise meta-analysis**
Standardized mean differences below 0 favor the first treatment of the comparison.

**
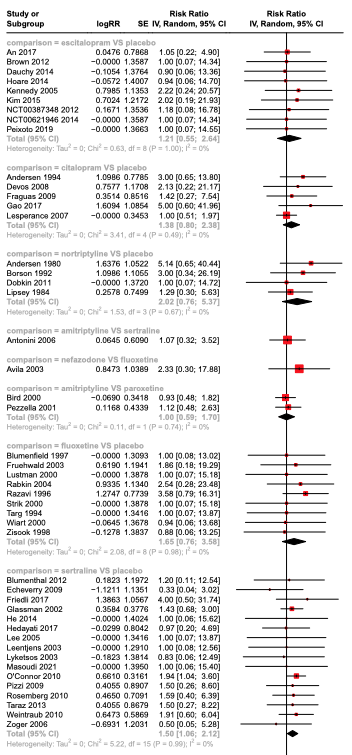
**

**
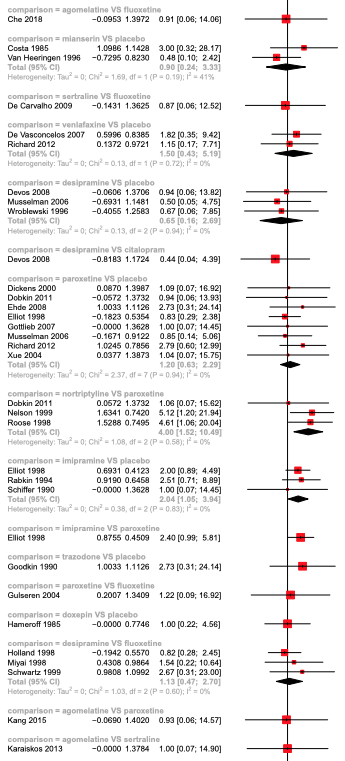
**

**
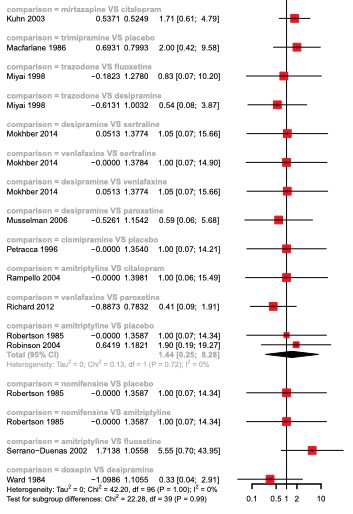
**

**Network map**

The thickness of lines is proportional to the number of studies comparing the two treatments and the size of circles is proportional to the number of individuals for each treatment.


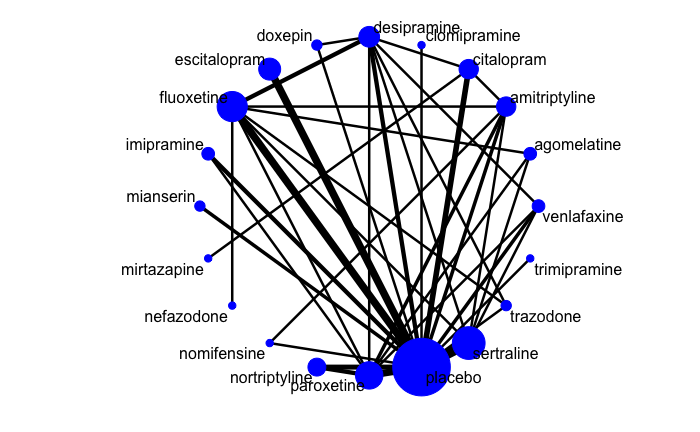


**Netleague table**

Risk ratios (RRs) and 95% confidence intervals (CIs) are reported. Results of the network meta-analysis are reported in the lower left part of the table and results from the pairwise meta-analysis are reported in the upper right part of the table. RRs lower than 1 favour the column-defining treatment.

| agomelatine | . | . | . | . | . | . | 0.91 (0.06 to 14.06) | . | . | . | . | . | . | 0.93 (0.06 to 14.57) | . | 1.00 (0.07 to 14.90) | . | . | . |
| --- | --- | --- | --- | --- | --- | --- | --- | --- | --- | --- | --- | --- | --- | --- | --- | --- | --- | --- | --- |
| 0.87 (0.17 to 4.56) | amitriptyline | 1.00 (0.06 to 15.49) | . | . | . | . | 5.55 (0.70 to 43.95) | . | . | . | . | 1.00 (0.07 to 14.34) | . | 1.00 (0.59 to 1.70) | 1.44 (0.25 to 8.28) | 1.07 (0.32 to 3.52) | . | . | . |
| 0.89 (0.17 to 4.82) | 1.03 (0.48 to 2.20) | citalopram | . | 2.27 (0.23 to 22.56) | . | . | . | . | . | 0.58 (0.21 to 1.63) | . | . | . | . | 1.38 (0.80 to 2.38) | . | . | . | . |
| 1.27 (0.06 to 28.19) | 1.46 (0.10 to 22.01) | 1.42 (0.09 to 21.24) | clomipramine | . | . | . | . | . | . | . | . | . | . | . | 1.00 (0.07 to 14.21) | . | . | . | . |
| 0.99 (0.18 to 5.55) | 1.14 (0.47 to 2.80) | 1.11 (0.46 to 2.69) | 0.78 (0.05 to 12.33) | desipramine | 3.00 (0.34 to 26.19) | . | 1.13 (0.47 to 2.70) | . | . | . | . | . | . | 0.59 (0.06 to 5.68) | 0.65 (0.16 to 2.69) | 1.05 (0.07 to 15.66) | 1.85 (0.26 to 13.19) | . | 1.05 (0.07 to 15.66) |
| 1.68 (0.22 to 12.81) | 1.93 (0.48 to 7.69) | 1.88 (0.48 to 7.38) | 1.33 (0.07 to 25.09) | 1.69 (0.44 to 6.45) | doxepin | . | . | . | . | . | . | . | . | . | 1.00 (0.22 to 4.56) | . | . | . | . |
| 1.05 (0.18 to 6.27) | 1.21 (0.46 to 3.19) | 1.18 (0.46 to 3.02) | 0.83 (0.05 to 13.19) | 1.06 (0.36 to 3.11) | 0.63 (0.14 to 2.77) | escitalopram | . | . | . | . | . | . | . | . | 1.21 (0.55 to 2.64) | . | . | . | . |
| 1.01 (0.20 to 5.16) | 1.16 (0.54 to 2.51) | 1.13 (0.52 to 2.48) | 0.80 (0.05 to 12.09) | 1.02 (0.50 to 2.06) | 0.60 (0.16 to 2.32) | 0.96 (0.36 to 2.57) | fluoxetine | . | . | . | 0.43 (0.06 to 3.28) | . | . | 0.82 (0.06 to 11.33) | 1.65 (0.76 to 3.58) | 1.15 (0.08 to 16.67) | 1.20 (0.10 to 14.69) | . | . |
| 0.54 (0.10 to 2.93) | 0.62 (0.29 to 1.32) | 0.60 (0.27 to 1.33) | 0.43 (0.03 to 6.46) | 0.54 (0.21 to 1.39) | 0.32 (0.08 to 1.30) | 0.51 (0.19 to 1.37) | 0.53 (0.23 to 1.22) | imipramine | . | . | . | . | . | 2.40 (0.99 to 5.81) | 2.04 (1.05 to 3.94) | . | . | . | . |
| 1.41 (0.18 to 11.18) | 1.62 (0.39 to 6.76) | 1.58 (0.38 to 6.47) | 1.11 (0.06 to 21.42) | 1.42 (0.31 to 6.37) | 0.84 (0.14 to 5.18) | 1.34 (0.29 to 6.16) | 1.40 (0.33 to 5.89) | 2.61 (0.62 to 10.99) | mianserin | . | . | . | . | . | 0.90 (0.24 to 3.33) | . | . | . | . |
| 0.52 (0.07 to 3.76) | 0.60 (0.17 to 2.16) | 0.58 (0.21 to 1.63) | 0.41 (0.02 to 7.44) | 0.53 (0.14 to 2.04) | 0.31 (0.06 to 1.72) | 0.50 (0.12 to 2.01) | 0.52 (0.14 to 1.89) | 0.97 (0.26 to 3.54) | 0.37 (0.06 to 2.12) | mirtazapine | . | . | . | . | . | . | . | . | . |
| 0.43 (0.03 to 5.88) | 0.50 (0.06 to 4.39) | 0.48 (0.05 to 4.29) | 0.34 (0.01 to 10.20) | 0.44 (0.05 to 3.75) | 0.26 (0.02 to 2.97) | 0.41 (0.04 to 3.95) | 0.43 (0.06 to 3.28) | 0.80 (0.09 to 7.22) | 0.31 (0.03 to 3.72) | 0.83 (0.07 to 9.25) | nefazodone | . | . | . | . | . | . | . | . |
| 1.05 (0.06 to 17.45) | 1.21 (0.12 to 12.33) | 1.18 (0.11 to 12.71) | 0.83 (0.02 to 28.21) | 1.06 (0.09 to 12.02) | 0.63 (0.04 to 8.80) | 1.00 (0.09 to 11.61) | 1.04 (0.10 to 11.36) | 1.95 (0.18 to 21.18) | 0.75 (0.05 to 10.74) | 2.01 (0.15 to 26.91) | 2.43 (0.11 to 56.11) | nomifensine | . | . | 1.00 (0.07 to 14.34) | . | . | . | . |
| 0.37 (0.07 to 2.12) | **0.43 (0.18 to 0.99)** | 0.42 (0.17 to 1.03) | 0.29 (0.02 to 4.61) | 0.37 (0.13 to 1.05) | **0.22 (0.05 to 0.96)** | 0.35 (0.12 to 1.04) | **0.37 (0.14 to 0.94)** | 0.69 (0.28 to 1.71) | 0.26 (0.06 to 1.19) | 0.71 (0.18 to 2.81) | 0.86 (0.09 to 8.07) | 0.35 (0.03 to 3.99) | nortriptyline | 4.00 (1.52 to 10.49) | 2.02 (0.76 to 5.37) | . | . | . | . |
| 0.98 (0.19 to 4.93) | 1.12 (0.70 to 1.79) | 1.09 (0.54 to 2.21) | 0.77 (0.05 to 11.44) | 0.98 (0.42 to 2.28) | 0.58 (0.15 to 2.24) | 0.93 (0.37 to 2.33) | 0.97 (0.47 to 1.99) | 1.81 (0.94 to 3.49) | 0.69 (0.17 to 2.80) | 1.87 (0.54 to 6.51) | 2.26 (0.26 to 19.57) | 0.93 (0.09 to 9.63) | **2.63 (1.25 to 5.51)** | paroxetine | 1.20 (0.63 to 2.29) | . | . | . | 2.43 (0.52 to 11.27) |
| 1.27 (0.25 to 6.31) | 1.46 (0.82 to 2.59) | 1.42 (0.84 to 2.41) | 1.00 (0.07 to 14.21) | 1.28 (0.61 to 2.67) | 0.75 (0.21 to 2.68) | 1.21 (0.55 to 2.64) | 1.26 (0.69 to 2.29) | **2.35 (1.30 to 4.26)** | 0.90 (0.24 to 3.33) | 2.43 (0.76 to 7.72) | 2.93 (0.35 to 24.48) | 1.21 (0.12 to 12.33) | **3.41 (1.62 to 7.17)** | 1.30 (0.80 to 2.10) | placebo | 0.67 (0.47 to 0.94) | 0.37 (0.04 to 3.25) | 0.50 (0.10 to 2.40) | 0.67 (0.19 to 2.32) |
| 0.86 (0.17 to 4.32) | 0.99 (0.53 to 1.84) | 0.97 (0.52 to 1.80) | 0.68 (0.05 to 9.87) | 0.87 (0.39 to 1.92) | 0.51 (0.14 to 1.90) | 0.82 (0.35 to 1.92) | 0.85 (0.44 to 1.66) | 1.60 (0.82 to 3.13) | 0.61 (0.16 to 2.36) | 1.65 (0.50 to 5.49) | 1.99 (0.23 to 16.98) | 0.82 (0.08 to 8.54) | **2.32 (1.04 to 5.18)** | 0.88 (0.51 to 1.54) | **0.68 (0.49 to 0.95)** | sertraline | . | . | 1.00 (0.07 to 14.90) |
| 0.96 (0.11 to 8.42) | 1.10 (0.23 to 5.40) | 1.08 (0.22 to 5.21) | 0.76 (0.04 to 15.94) | 0.97 (0.22 to 4.32) | 0.57 (0.08 to 3.88) | 0.91 (0.17 to 4.94) | 0.95 (0.21 to 4.39) | 1.78 (0.36 to 8.86) | 0.68 (0.09 to 4.98) | 1.84 (0.28 to 12.11) | 2.22 (0.17 to 28.34) | 0.92 (0.06 to 14.45) | 2.59 (0.49 to 13.64) | 0.98 (0.21 to 4.67) | 0.76 (0.17 to 3.38) | 1.11 (0.24 to 5.13) | trazodone | . | . |
| 0.63 (0.07 to 5.97) | 0.73 (0.14 to 3.86) | 0.71 (0.14 to 3.71) | 0.50 (0.02 to 10.90) | 0.64 (0.11 to 3.61) | 0.38 (0.05 to 2.83) | 0.60 (0.10 to 3.48) | 0.63 (0.12 to 3.36) | 1.17 (0.22 to 6.28) | 0.45 (0.06 to 3.47) | 1.21 (0.17 to 8.51) | 1.47 (0.10 to 20.50) | 0.60 (0.04 to 9.95) | 1.71 (0.30 to 9.66) | 0.65 (0.13 to 3.34) | 0.50 (0.10 to 2.40) | 0.73 (0.15 to 3.64) | 0.66 (0.08 to 5.75) | trimipramine | . |
| 1.18 (0.18 to 7.70) | 1.35 (0.45 to 4.12) | 1.32 (0.42 to 4.13) | 0.93 (0.05 to 15.95) | 1.19 (0.35 to 3.96) | 0.70 (0.14 to 3.52) | 1.12 (0.31 to 4.05) | 1.17 (0.37 to 3.70) | 2.18 (0.69 to 6.93) | 0.84 (0.16 to 4.39) | 2.26 (0.49 to 10.50) | 2.73 (0.26 to 28.28) | 1.12 (0.09 to 14.01) | 3.17 (0.93 to 10.80) | 1.21 (0.42 to 3.44) | 0.93 (0.34 to 2.57) | 1.37 (0.48 to 3.92) | 1.23 (0.20 to 7.34) | 1.86 (0.29 to 12.04) | venlafaxine |

**Forest plot**

Placebo was used as a common comparator. RRs below 1 favor the treatment over the common comparator.


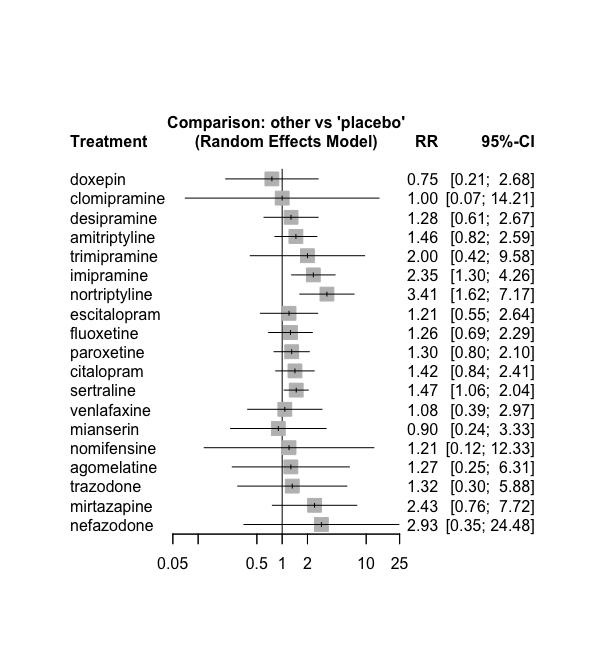


**Assessment of heterogeneity and consistency**

*Global heterogeneity*

We interpreted tau^2 as follows: heterogeneity low with tau^2≤0.010, moderate with 0.010<tau^2≤0.242, high with tau^2>0.242, and I^2 statistics as follows: not important (0%-40%), moderate (30%-60%), substantial (50%-90%), considerable (75%-100%).

tau^2= 0 ; tau= 0

I^2= 0 % (0 % to 28.37 %)

*Consistency: global approach*

Q statistic to assess consistency under the assumption of a full design-by-treatment interaction random effects model

Q df p-value tau.within tau2.within
Between designs 11.26 26 0.9946 0 0

*Consistency: local approach*

Separate indirect from direct evidence (SIDE) using back-calculation method

Random effects model:

comparison k prop nma direct indir. RoR z p-value

agomelatine:fluoxetine 1 0.35 1.01 0.91 1.07 0.85 -0.09 0.9254

agomelatine:paroxetine 1 0.35 0.98 0.93 1.00 0.93 -0.04 0.9673

agomelatine:sertraline 1 0.35 0.86 1.00 0.80 1.26 0.13 0.8943

amitriptyline:citalopram 1 0.08 1.03 1.00 1.03 0.97 -0.02 0.9846

amitriptyline:fluoxetine 1 0.14 1.16 5.55 0.90 6.17 1.60 0.1099

amitriptyline:nomifensine 1 0.76 1.21 1.00 2.20 0.45 -0.28 0.7767

amitriptyline:paroxetine 2 0.79 1.12 1.00 1.72 0.58 -0.92 0.3558

placebo:amitriptyline 2 0.11 0.69 0.69 0.69 1.01 0.01 0.9897

amitriptyline:sertraline 1 0.27 0.99 1.07 0.96 1.11 0.14 0.8880

citalopram:desipramine 1 0.15 1.11 2.27 0.98 2.30 0.66 0.5111

placebo:citalopram 5 0.94 0.70 0.72 0.47 1.55 0.38 0.7025

desipramine:doxepin 1 0.38 1.69 3.00 1.19 2.53 0.66 0.5092

desipramine:fluoxetine 3 0.66 1.02 1.13 0.82 1.37 0.42 0.6749

desipramine:paroxetine 1 0.14 0.98 0.59 1.07 0.55 -0.47 0.6349

placebo:desipramine 3 0.27 0.78 1.53 0.61 2.51 1.09 0.2778

desipramine:sertraline 1 0.09 0.87 1.05 0.85 1.24 0.15 0.8834

desipramine:trazodone 1 0.58 0.97 1.85 0.40 4.64 0.99 0.3206

desipramine:venlafaxine 1 0.20 1.19 1.05 1.22 0.86 -0.10 0.9230

placebo:doxepin 1 0.70 1.33 1.00 2.53 0.40 -0.66 0.5092

fluoxetine:paroxetine 1 0.07 0.97 0.82 0.98 0.83 -0.13 0.8962

placebo:fluoxetine 9 0.59 0.80 0.61 1.18 0.51 -1.08 0.2819

fluoxetine:sertraline 1 0.06 0.85 1.15 0.84 1.38 0.23 0.8202

fluoxetine:trazodone 1 0.37 0.95 1.20 0.83 1.44 0.23 0.8197

imipramine:paroxetine 1 0.55 1.81 2.40 1.28 1.88 0.94 0.3495

placebo:imipramine 3 0.81 0.43 0.49 0.23 2.13 0.97 0.3312

placebo:nomifensine 1 0.76 0.83 1.00 0.45 2.20 0.28 0.7767

nortriptyline:paroxetine 3 0.59 2.63 4.00 1.44 2.78 1.33 0.1832

placebo:nortriptyline 4 0.58 0.29 0.49 0.14 3.47 1.62 0.1054

placebo:paroxetine 8 0.56 0.77 0.83 0.70 1.20 0.36 0.7159

paroxetine:venlafaxine 1 0.47 1.21 2.43 0.66 3.71 1.22 0.2216

placebo:sertraline 16 0.90 0.68 0.67 0.82 0.82 -0.35 0.7261

placebo:trazodone 1 0.47 0.76 0.37 1.44 0.25 -0.90 0.3698

placebo:venlafaxine 2 0.67 0.93 0.67 1.81 0.37 -0.90 0.3674

sertraline:venlafaxine 1 0.15 1.37 1.00 1.45 0.69 -0.25 0.8057
Legend:
 comparison - Treatment comparison
 k - Number of studies providing direct evidence
 prop - Direct evidence proportion
 nma - Estimated treatment effect (RR) in network meta-analysis
 direct - Estimated treatment effect (RR) derived from direct evidence
 indir. - Estimated treatment effect (RR) derived from indirect evidence
 RoR - Ratio of Ratios (direct versus indirect)
 z - z-value of test for disagreement (direct versus indirect)
 p-value - p-value of test for disagreement (direct versus indirect)

**Netrank**

Higher p-score values indicate higher ranking of treatments

P-score

doxepin 0.7685

placebo 0.7401

mianserin 0.6962

venlafaxine 0.6400

clomipramine 0.5968

escitalopram 0.5884

fluoxetine 0.5708

desipramine 0.5575

paroxetine 0.5541

nomifensine 0.5511

agomelatine 0.5459

trazodone 0.5310

citalopram 0.4924

amitriptyline 0.4718

sertraline 0.4601

trimipramine 0.3664

nefazodone 0.2756

mirtazapine 0.2589

imipramine 0.2209

nortriptyline 0.1133

**Assessment of small-study effect**

We produced funnel plots and performed the Egger’s test only for those comparisons including at least 8 studies.

| **Funnel plot** | **Egger’s test** |
| --- | --- |
| Escitalopram vs. placebo   | Egger's test for small-study effects:  Regress standard normal deviate of intervention  effect estimate against its standard error  .  Number of studies = 9 Root MSE = .4474  ------------------------------------------------------------------------------  Std_Eff \| Coefficient Std. err. t P>\|t\| [95% conf. interval]  -------------+----------------------------------------------------------------  slope \| -.0087967 .6552928 -0.01 0.990 -1.558318 1.540725  bias \| .3610936 .480769 0.75 0.477 -.7757446 1.497932  ------------------------------------------------------------------------------  Test of H0: no small-study effects P = 0.477 |
| Sertraline vs. placebo   | Egger's test for small-study effects:  Regress standard normal deviate of intervention  effect estimate against its standard error  .  Number of studies = 16 Root MSE = .6406  ------------------------------------------------------------------------------  Std_Eff \| Coefficient Std. err. t P>\|t\| [95% conf. interval]  -------------+----------------------------------------------------------------  slope \| .6828049 .2101507 3.25 0.006 .2320765 1.133533  bias \| -.4009205 .2860735 -1.40 0.183 -1.014487 .2126461  ------------------------------------------------------------------------------  Test of H0: no small-study effects P = 0.183 |
| Fluoxetine vs. placebo     | Egger's test for small-study effects:  Regress standard normal deviate of intervention  effect estimate against its standard error  .  Number of studies = 9 Root MSE = .5484  ------------------------------------------------------------------------------  Std_Eff \| Coefficient Std. err. t P>\|t\| [95% conf. interval]  -------------+----------------------------------------------------------------  slope \| 2.083418 .7836275 2.66 0.033 .2304332 3.936402  bias \| -1.09736 .5781957 -1.90 0.100 -2.464575 .2698561  ------------------------------------------------------------------------------  Test of H0: no small-study effects P = 0.100 |
| Paroxetine vs. placebo   | Egger's test for small-study effects:  Regress standard normal deviate of intervention  effect estimate against its standard error  .  Number of studies = 8 Root MSE = .6921  ------------------------------------------------------------------------------  Std_Eff \| Coefficient Std. err. t P>\|t\| [95% conf. interval]  -------------+----------------------------------------------------------------  slope \| -.2465769 .5502695 -0.45 0.670 -1.593038 1.099884  bias \| .5683454 .5570889 1.02 0.347 -.794802 1.931493  ------------------------------------------------------------------------------  Test of H0: no small-study effects P = 0.347 |

**CINeMA assessment**

We conducted the analysis with CINeMA according to the following setup:

- Within-study Bias: we summarize risk of bias across contributions for each network estimate according to the “majority” RoB;
- Reporting Bias: we set all comparisons to “low risk”;
- Indirectness: we summarize risk of indirectness across contributions for each network estimate according to the “majority” indirectness;
- Imprecision, heterogeneity, incoherence: we defined relative effect estimates below 0.500 and above 2.000 are considered clinically important.

*Final report*

| Comparison | Number of studies | Within-study bias | Reporting bias | no concerns | major concerns | no concerns | no concerns | Confidence rating |
| --- | --- | --- | --- | --- | --- | --- | --- | --- |
| agomelatine:fluoxetine | 1 | some concerns | low risk | no concerns | major concerns | no concerns | no concerns | Very Low |
| agomelatine:paroxetine | 1 | some concerns | low risk | no concerns | major concerns | no concerns | no concerns | Very Low |
| agomelatine:sertraline | 1 | some concerns | low risk | no concerns | major concerns | no concerns | some concerns | Very Low |
| amitriptyline:fluoxetine | 1 | no concerns | low risk | no concerns | major concerns | no concerns | no concerns | Very Low |
| amitriptyline:nomifensine | 1 | some concerns | low risk | no concerns | no concerns | no concerns | no concerns | Very Low |
| amitriptyline:paroxetine | 2 | no concerns | low risk | no concerns | some concerns | no concerns | no concerns | High |
| amitriptyline:placebo | 2 | no concerns | low risk | no concerns | some concerns | no concerns | no concerns | Moderate |
| amitriptyline:sertraline | 1 | no concerns | low risk | no concerns | major concerns | no concerns | no concerns | Moderate |
| citalopram:desipramine | 1 | no concerns | low risk | no concerns | some concerns | no concerns | no concerns | Low |
| citalopram:mirtazapine | 1 | major concerns | low risk | no concerns | some concerns | no concerns | no concerns | Very Low |
| citalopram:placebo | 7 | no concerns | low risk | some concerns | major concerns | no concerns | no concerns | Moderate |
| clomipramine:placebo | 1 | some concerns | low risk | no concerns | some concerns | some concerns | no concerns | Very Low |
| desipramine:doxepin | 1 | some concerns | low risk | no concerns | major concerns | no concerns | no concerns | Very Low |
| desipramine:fluoxetine | 3 | some concerns | low risk | no concerns | major concerns | no concerns | no concerns | Very Low |
| desipramine:paroxetine | 1 | no concerns | low risk | no concerns | some concerns | no concerns | no concerns | Low |
| desipramine:placebo | 3 | some concerns | low risk | no concerns | major concerns | no concerns | no concerns | Low |
| desipramine:sertraline | 1 | no concerns | low risk | some concerns | major concerns | no concerns | no concerns | Low |
| desipramine:trazodone | 1 | major concerns | low risk | some concerns | major concerns | no concerns | no concerns | Very Low |
| desipramine:venlafaxine | 1 | some concerns | low risk | no concerns | major concerns | no concerns | no concerns | Very Low |
| doxepin:placebo | 1 | some concerns | low risk | no concerns | some concerns | no concerns | no concerns | Very Low |
| escitalopram:placebo | 9 | some concerns | low risk | no concerns | major concerns | no concerns | no concerns | Low |
| fluoxetine:nefazodone | 1 | some concerns | low risk | no concerns | major concerns | no concerns | no concerns | Very Low |
| fluoxetine:paroxetine | 1 | some concerns | low risk | no concerns | some concerns | no concerns | no concerns | Very Low |
| fluoxetine:placebo | 9 | some concerns | low risk | no concerns | major concerns | no concerns | no concerns | Low |
| fluoxetine:sertraline | 1 | no concerns | low risk | some concerns | major concerns | no concerns | no concerns | Low |
| fluoxetine:trazodone | 1 | major concerns | low risk | no concerns | some concerns | no concerns | no concerns | Very Low |
| imipramine:paroxetine | 1 | major concerns | low risk | no concerns | no concerns | no concerns | no concerns | Very Low |
| imipramine:placebo | 3 | major concerns | low risk | no concerns | major concerns | no concerns | no concerns | Low |
| mianserin:placebo | 2 | some concerns | low risk | no concerns | major concerns | no concerns | no concerns | Very Low |
| nomifensine:placebo | 1 | some concerns | low risk | no concerns | no concerns | no concerns | no concerns | Very Low |
| nortriptyline:paroxetine | 3 | some concerns | low risk | no concerns | no concerns | no concerns | no concerns | Moderate |
| nortriptyline:placebo | 4 | some concerns | low risk | no concerns | some concerns | no concerns | no concerns | Moderate |
| paroxetine:placebo | 8 | no concerns | low risk | some concerns | major concerns | no concerns | no concerns | Moderate |
| paroxetine:venlafaxine | 1 | no concerns | low risk | no concerns | no concerns | no concerns | no concerns | Very Low |
| placebo:sertraline | 16 | no concerns | low risk | major concerns | major concerns | no concerns | no concerns | High |
| placebo:trazodone | 1 | no concerns | low risk | some concerns | major concerns | no concerns | no concerns | Very Low |
| placebo:trimipramine | 1 | some concerns | low risk | some concerns | major concerns | no concerns | no concerns | Very Low |
| placebo:venlafaxine | 2 | some concerns | low risk | some concerns | major concerns | no concerns | no concerns | Very Low |
| sertraline:venlafaxine | 1 | some concerns | low risk | no concerns | major concerns | no concerns | no concerns | Very Low |
| agomelatine:amitriptyline | 0 | some concerns | low risk | no concerns | major concerns | no concerns | no concerns | Very Low |
| agomelatine:citalopram | 0 | some concerns | low risk | some concerns | major concerns | no concerns | no concerns | Very Low |
| agomelatine:clomipramine | 0 | some concerns | low risk | no concerns | major concerns | no concerns | no concerns | Very Low |
| agomelatine:desipramine | 0 | some concerns | low risk | no concerns | major concerns | no concerns | no concerns | Very Low |
| agomelatine:doxepin | 0 | some concerns | low risk | no concerns | major concerns | no concerns | no concerns | Very Low |
| agomelatine:escitalopram | 0 | some concerns | low risk | no concerns | major concerns | no concerns | no concerns | Very Low |
| agomelatine:imipramine | 0 | some concerns | low risk | no concerns | major concerns | no concerns | no concerns | Very Low |
| agomelatine:mianserin | 0 | some concerns | low risk | no concerns | major concerns | no concerns | no concerns | Very Low |
| agomelatine:mirtazapine | 0 | some concerns | low risk | no concerns | major concerns | no concerns | no concerns | Very Low |
| agomelatine:nefazodone | 0 | some concerns | low risk | no concerns | major concerns | no concerns | no concerns | Very Low |
| agomelatine:nomifensine | 0 | some concerns | low risk | no concerns | major concerns | no concerns | no concerns | Very Low |
| agomelatine:nortriptyline | 0 | some concerns | low risk | no concerns | major concerns | no concerns | no concerns | Very Low |
| agomelatine:placebo | 0 | some concerns | low risk | some concerns | major concerns | no concerns | no concerns | Very Low |
| agomelatine:trazodone | 0 | some concerns | low risk | some concerns | major concerns | no concerns | no concerns | Very Low |
| agomelatine:trimipramine | 0 | some concerns | low risk | some concerns | major concerns | no concerns | no concerns | Very Low |
| agomelatine:venlafaxine | 0 | some concerns | low risk | no concerns | some concerns | no concerns | no concerns | Very Low |
| amitriptyline:citalopram | 0 | no concerns | low risk | some concerns | major concerns | no concerns | no concerns | Moderate |
| amitriptyline:clomipramine | 0 | some concerns | low risk | no concerns | major concerns | no concerns | no concerns | Very Low |
| amitriptyline:desipramine | 0 | no concerns | low risk | no concerns | some concerns | some concerns | no concerns | Low |
| amitriptyline:doxepin | 0 | some concerns | low risk | no concerns | major concerns | no concerns | no concerns | Very Low |
| amitriptyline:escitalopram | 0 | some concerns | low risk | no concerns | some concerns | no concerns | no concerns | Very Low |
| amitriptyline:imipramine | 0 | major concerns | low risk | no concerns | major concerns | no concerns | no concerns | Very Low |
| amitriptyline:mianserin | 0 | no concerns | low risk | no concerns | major concerns | no concerns | no concerns | Low |
| amitriptyline:mirtazapine | 0 | no concerns | low risk | no concerns | major concerns | no concerns | no concerns | Low |
| amitriptyline:nefazodone | 0 | some concerns | low risk | no concerns | no concerns | no concerns | no concerns | Very Low |
| amitriptyline:nortriptyline | 0 | some concerns | low risk | no concerns | major concerns | no concerns | no concerns | Moderate |
| amitriptyline:trazodone | 0 | no concerns | low risk | some concerns | major concerns | no concerns | no concerns | Low |
| amitriptyline:trimipramine | 0 | some concerns | low risk | some concerns | major concerns | no concerns | no concerns | Very Low |
| amitriptyline:venlafaxine | 0 | no concerns | low risk | no concerns | major concerns | no concerns | no concerns | Very Low |
| citalopram:clomipramine | 0 | some concerns | low risk | no concerns | major concerns | no concerns | no concerns | Very Low |
| citalopram:doxepin | 0 | some concerns | low risk | no concerns | major concerns | no concerns | no concerns | Very Low |
| citalopram:escitalopram | 0 | some concerns | low risk | no concerns | major concerns | no concerns | no concerns | Very Low |
| citalopram:fluoxetine | 0 | no concerns | low risk | no concerns | some concerns | no concerns | no concerns | Low |
| citalopram:imipramine | 0 | no concerns | low risk | no concerns | major concerns | no concerns | no concerns | Moderate |
| citalopram:mianserin | 0 | no concerns | low risk | no concerns | major concerns | no concerns | no concerns | Low |
| citalopram:nefazodone | 0 | some concerns | low risk | no concerns | major concerns | no concerns | no concerns | Very Low |
| citalopram:nomifensine | 0 | some concerns | low risk | no concerns | no concerns | no concerns | no concerns | Very Low |
| citalopram:nortriptyline | 0 | some concerns | low risk | no concerns | major concerns | no concerns | no concerns | Moderate |
| citalopram:paroxetine | 0 | no concerns | low risk | no concerns | no concerns | no concerns | no concerns | Low |
| citalopram:sertraline | 0 | no concerns | low risk | no concerns | major concerns | no concerns | no concerns | High |
| citalopram:trazodone | 0 | no concerns | low risk | no concerns | major concerns | no concerns | no concerns | Low |
| citalopram:trimipramine | 0 | some concerns | low risk | no concerns | major concerns | no concerns | no concerns | Very Low |
| citalopram:venlafaxine | 0 | no concerns | low risk | some concerns | major concerns | no concerns | no concerns | Low |
| clomipramine:desipramine | 0 | some concerns | low risk | no concerns | major concerns | no concerns | no concerns | Very Low |
| clomipramine:doxepin | 0 | some concerns | low risk | some concerns | major concerns | no concerns | no concerns | Very Low |
| clomipramine:escitalopram | 0 | some concerns | low risk | some concerns | major concerns | no concerns | no concerns | Very Low |
| clomipramine:fluoxetine | 0 | some concerns | low risk | no concerns | major concerns | no concerns | no concerns | Very Low |
| clomipramine:imipramine | 0 | some concerns | low risk | no concerns | major concerns | no concerns | no concerns | Very Low |
| clomipramine:mianserin | 0 | some concerns | low risk | no concerns | major concerns | no concerns | no concerns | Very Low |
| clomipramine:mirtazapine | 0 | some concerns | low risk | no concerns | major concerns | no concerns | no concerns | Very Low |
| clomipramine:nefazodone | 0 | some concerns | low risk | no concerns | major concerns | no concerns | no concerns | Very Low |
| clomipramine:nomifensine | 0 | some concerns | low risk | no concerns | major concerns | no concerns | no concerns | Very Low |
| clomipramine:nortriptyline | 0 | some concerns | low risk | some concerns | major concerns | no concerns | no concerns | Very Low |
| clomipramine:paroxetine | 0 | some concerns | low risk | some concerns | major concerns | no concerns | no concerns | Very Low |
| clomipramine:sertraline | 0 | some concerns | low risk | some concerns | major concerns | no concerns | no concerns | Very Low |
| clomipramine:trazodone | 0 | some concerns | low risk | some concerns | major concerns | no concerns | no concerns | Very Low |
| clomipramine:trimipramine | 0 | some concerns | low risk | some concerns | major concerns | no concerns | no concerns | Very Low |
| clomipramine:venlafaxine | 0 | some concerns | low risk | no concerns | major concerns | no concerns | no concerns | Very Low |
| desipramine:escitalopram | 0 | some concerns | low risk | no concerns | some concerns | no concerns | no concerns | Very Low |
| desipramine:imipramine | 0 | some concerns | low risk | no concerns | major concerns | no concerns | no concerns | Low |
| desipramine:mianserin | 0 | some concerns | low risk | no concerns | major concerns | no concerns | no concerns | Very Low |
| desipramine:mirtazapine | 0 | no concerns | low risk | no concerns | major concerns | no concerns | no concerns | Low |
| desipramine:nefazodone | 0 | some concerns | low risk | no concerns | major concerns | no concerns | no concerns | Very Low |
| desipramine:nomifensine | 0 | some concerns | low risk | no concerns | some concerns | no concerns | no concerns | Very Low |
| desipramine:nortriptyline | 0 | some concerns | low risk | some concerns | major concerns | no concerns | no concerns | Low |
| desipramine:trimipramine | 0 | some concerns | low risk | no concerns | major concerns | no concerns | no concerns | Very Low |
| doxepin:escitalopram | 0 | some concerns | low risk | no concerns | major concerns | no concerns | no concerns | Very Low |
| doxepin:fluoxetine | 0 | some concerns | low risk | no concerns | some concerns | no concerns | no concerns | Very Low |
| doxepin:imipramine | 0 | some concerns | low risk | no concerns | major concerns | no concerns | no concerns | Low |
| doxepin:mianserin | 0 | some concerns | low risk | no concerns | some concerns | no concerns | no concerns | Very Low |
| doxepin:mirtazapine | 0 | some concerns | low risk | no concerns | major concerns | no concerns | no concerns | Low |
| doxepin:nefazodone | 0 | some concerns | low risk | no concerns | major concerns | no concerns | no concerns | Very Low |
| doxepin:nomifensine | 0 | some concerns | low risk | no concerns | no concerns | no concerns | no concerns | Very Low |
| doxepin:nortriptyline | 0 | some concerns | low risk | no concerns | major concerns | no concerns | no concerns | Moderate |
| doxepin:paroxetine | 0 | some concerns | low risk | no concerns | major concerns | no concerns | no concerns | Very Low |
| doxepin:sertraline | 0 | some concerns | low risk | no concerns | major concerns | no concerns | no concerns | Very Low |
| doxepin:trazodone | 0 | some concerns | low risk | no concerns | major concerns | no concerns | no concerns | Very Low |
| doxepin:trimipramine | 0 | some concerns | low risk | no concerns | major concerns | no concerns | no concerns | Very Low |
| doxepin:venlafaxine | 0 | some concerns | low risk | no concerns | major concerns | no concerns | no concerns | Very Low |
| escitalopram:fluoxetine | 0 | some concerns | low risk | no concerns | some concerns | no concerns | no concerns | Very Low |
| escitalopram:imipramine | 0 | some concerns | low risk | no concerns | major concerns | no concerns | no concerns | Low |
| escitalopram:mianserin | 0 | some concerns | low risk | no concerns | major concerns | no concerns | no concerns | Very Low |
| escitalopram:mirtazapine | 0 | some concerns | low risk | no concerns | major concerns | no concerns | no concerns | Very Low |
| escitalopram:nefazodone | 0 | some concerns | low risk | no concerns | major concerns | no concerns | no concerns | Very Low |
| escitalopram:nomifensine | 0 | some concerns | low risk | no concerns | some concerns | no concerns | no concerns | Very Low |
| escitalopram:nortriptyline | 0 | some concerns | low risk | no concerns | major concerns | no concerns | no concerns | Low |
| escitalopram:paroxetine | 0 | some concerns | low risk | no concerns | major concerns | no concerns | no concerns | Very Low |
| escitalopram:sertraline | 0 | some concerns | low risk | no concerns | major concerns | no concerns | no concerns | Very Low |
| escitalopram:trazodone | 0 | some concerns | low risk | some concerns | major concerns | no concerns | no concerns | Very Low |
| escitalopram:trimipramine | 0 | some concerns | low risk | some concerns | major concerns | no concerns | no concerns | Very Low |
| escitalopram:venlafaxine | 0 | some concerns | low risk | no concerns | some concerns | no concerns | no concerns | Very Low |
| fluoxetine:imipramine | 0 | some concerns | low risk | no concerns | major concerns | no concerns | no concerns | Low |
| fluoxetine:mianserin | 0 | some concerns | low risk | no concerns | major concerns | no concerns | no concerns | Very Low |
| fluoxetine:mirtazapine | 0 | no concerns | low risk | no concerns | major concerns | no concerns | no concerns | Low |
| fluoxetine:nomifensine | 0 | some concerns | low risk | no concerns | some concerns | no concerns | no concerns | Very Low |
| fluoxetine:nortriptyline | 0 | some concerns | low risk | some concerns | major concerns | no concerns | no concerns | Low |
| fluoxetine:trimipramine | 0 | some concerns | low risk | some concerns | major concerns | no concerns | no concerns | Very Low |
| fluoxetine:venlafaxine | 0 | some concerns | low risk | no concerns | some concerns | no concerns | no concerns | Very Low |
| imipramine:mianserin | 0 | some concerns | low risk | no concerns | major concerns | no concerns | no concerns | Low |
| imipramine:mirtazapine | 0 | major concerns | low risk | no concerns | major concerns | no concerns | no concerns | Very Low |
| imipramine:nefazodone | 0 | some concerns | low risk | no concerns | major concerns | no concerns | no concerns | Very Low |
| imipramine:nomifensine | 0 | some concerns | low risk | no concerns | some concerns | no concerns | no concerns | Very Low |
| imipramine:nortriptyline | 0 | some concerns | low risk | no concerns | some concerns | no concerns | no concerns | Low |
| imipramine:sertraline | 0 | major concerns | low risk | no concerns | major concerns | no concerns | no concerns | Very Low |
| imipramine:trazodone | 0 | major concerns | low risk | no concerns | major concerns | no concerns | no concerns | Very Low |
| imipramine:trimipramine | 0 | some concerns | low risk | no concerns | some concerns | no concerns | no concerns | Very Low |
| imipramine:venlafaxine | 0 | major concerns | low risk | no concerns | major concerns | no concerns | no concerns | Very Low |
| mianserin:mirtazapine | 0 | no concerns | low risk | no concerns | major concerns | no concerns | no concerns | Low |
| mianserin:nefazodone | 0 | some concerns | low risk | no concerns | major concerns | no concerns | no concerns | Very Low |
| mianserin:nomifensine | 0 | some concerns | low risk | no concerns | no concerns | some concerns | no concerns | Very Low |
| mianserin:nortriptyline | 0 | some concerns | low risk | no concerns | major concerns | no concerns | no concerns | Low |
| mianserin:paroxetine | 0 | some concerns | low risk | no concerns | major concerns | no concerns | no concerns | Very Low |
| mianserin:sertraline | 0 | some concerns | low risk | no concerns | major concerns | no concerns | no concerns | Very Low |
| mianserin:trazodone | 0 | no concerns | low risk | no concerns | major concerns | no concerns | no concerns | Low |
| mianserin:trimipramine | 0 | some concerns | low risk | no concerns | major concerns | no concerns | no concerns | Very Low |
| mianserin:venlafaxine | 0 | some concerns | low risk | no concerns | major concerns | no concerns | no concerns | Very Low |
| mirtazapine:nefazodone | 0 | some concerns | low risk | no concerns | major concerns | no concerns | no concerns | Very Low |
| mirtazapine:nomifensine | 0 | some concerns | low risk | no concerns | major concerns | no concerns | no concerns | Very Low |
| mirtazapine:nortriptyline | 0 | some concerns | low risk | no concerns | major concerns | no concerns | no concerns | Very Low |
| mirtazapine:paroxetine | 0 | major concerns | low risk | no concerns | some concerns | no concerns | no concerns | Very Low |
| mirtazapine:placebo | 0 | major concerns | low risk | no concerns | major concerns | no concerns | no concerns | Very Low |
| mirtazapine:sertraline | 0 | no concerns | low risk | no concerns | major concerns | no concerns | no concerns | Low |
| mirtazapine:trazodone | 0 | major concerns | low risk | no concerns | major concerns | no concerns | no concerns | Very Low |
| mirtazapine:trimipramine | 0 | some concerns | low risk | no concerns | major concerns | no concerns | no concerns | Very Low |
| mirtazapine:venlafaxine | 0 | no concerns | low risk | no concerns | major concerns | no concerns | no concerns | Low |
| nefazodone:nomifensine | 0 | some concerns | low risk | no concerns | major concerns | no concerns | no concerns | Very Low |
| nefazodone:nortriptyline | 0 | some concerns | low risk | no concerns | major concerns | no concerns | no concerns | Very Low |
| nefazodone:paroxetine | 0 | some concerns | low risk | no concerns | major concerns | no concerns | no concerns | Very Low |
| nefazodone:placebo | 0 | some concerns | low risk | no concerns | major concerns | no concerns | no concerns | Very Low |
| nefazodone:sertraline | 0 | some concerns | low risk | no concerns | major concerns | no concerns | no concerns | Very Low |
| nefazodone:trazodone | 0 | some concerns | low risk | no concerns | major concerns | no concerns | no concerns | Very Low |
| nefazodone:trimipramine | 0 | some concerns | low risk | no concerns | major concerns | no concerns | no concerns | Very Low |
| nefazodone:venlafaxine | 0 | some concerns | low risk | no concerns | major concerns | no concerns | no concerns | Very Low |
| nomifensine:nortriptyline | 0 | some concerns | low risk | no concerns | major concerns | no concerns | no concerns | Very Low |
| nomifensine:paroxetine | 0 | some concerns | low risk | no concerns | major concerns | no concerns | no concerns | Very Low |
| nomifensine:sertraline | 0 | some concerns | low risk | no concerns | major concerns | no concerns | no concerns | Very Low |
| nomifensine:trazodone | 0 | some concerns | low risk | no concerns | major concerns | no concerns | no concerns | Very Low |
| nomifensine:trimipramine | 0 | some concerns | low risk | no concerns | major concerns | no concerns | no concerns | Very Low |
| nomifensine:venlafaxine | 0 | some concerns | low risk | no concerns | no concerns | no concerns | no concerns | Very Low |
| nortriptyline:sertraline | 0 | some concerns | low risk | no concerns | major concerns | no concerns | no concerns | Moderate |
| nortriptyline:trazodone | 0 | some concerns | low risk | no concerns | major concerns | no concerns | no concerns | Very Low |
| nortriptyline:trimipramine | 0 | some concerns | low risk | no concerns | no concerns | no concerns | no concerns | Very Low |
| nortriptyline:venlafaxine | 0 | some concerns | low risk | no concerns | no concerns | no concerns | no concerns | Moderate |
| paroxetine:sertraline | 0 | no concerns | low risk | no concerns | major concerns | no concerns | no concerns | High |
| paroxetine:trazodone | 0 | no concerns | low risk | some concerns | major concerns | no concerns | no concerns | Low |
| paroxetine:trimipramine | 0 | some concerns | low risk | some concerns | major concerns | no concerns | no concerns | Very Low |
| sertraline:trazodone | 0 | no concerns | low risk | some concerns | major concerns | no concerns | no concerns | Very Low |
| sertraline:trimipramine | 0 | some concerns | low risk | some concerns | major concerns | no concerns | no concerns | Very Low |
| trazodone:trimipramine | 0 | some concerns | low risk | some concerns | major concerns | no concerns | no concerns | Very Low |
| trazodone:venlafaxine | 0 | no concerns | low risk | some concerns | major concerns | no concerns | no concerns | Very Low |
| trimipramine:venlafaxine | 0 | some concerns | low risk | no concerns | major concerns | no concerns | no concerns | Very Low |

**Sensitivity analyses (tolerability)**

| **Analysis** | **Network characteristics and Network map** | **Forest plot** | **Heterogeneity** | **Inconsistency** |
| --- | --- | --- | --- | --- |
| Main analysis | N=82, n=6123   |  | tau^2=0  I^2=0 % (0 % to 28.37 %) | Global approach:  p=0.9948  Local approach (SIDE): 0/34 |
| Excluding RCTs that were not blind | N=78, n=5890   |  | tau^2=0  I^2=0 % (0 % to 29.09 %) | Global approach:  p=0.9951  Local approach (SIDE): 0/30 |
| Excluding RCTs with follow-up <3 months | N=55, n=3776   |  | tau^2=0  I^2=0 % (0 % to 35.13 %) | Global approach:  p=0.9115  Local approach (SIDE): 0/20 |
| Excluding RCTs with an overall high risk of bias | N=76, n=5889  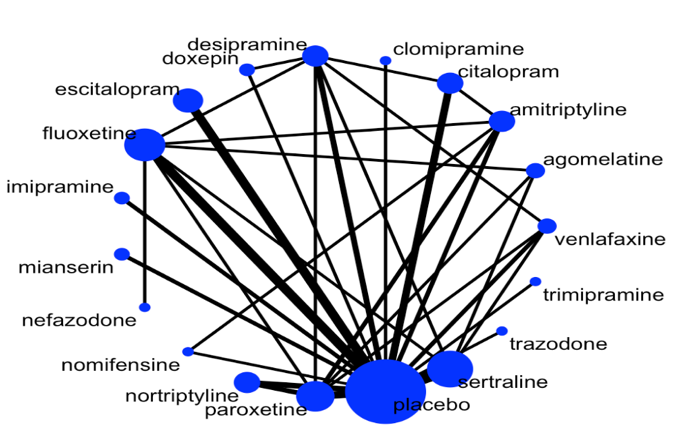 | 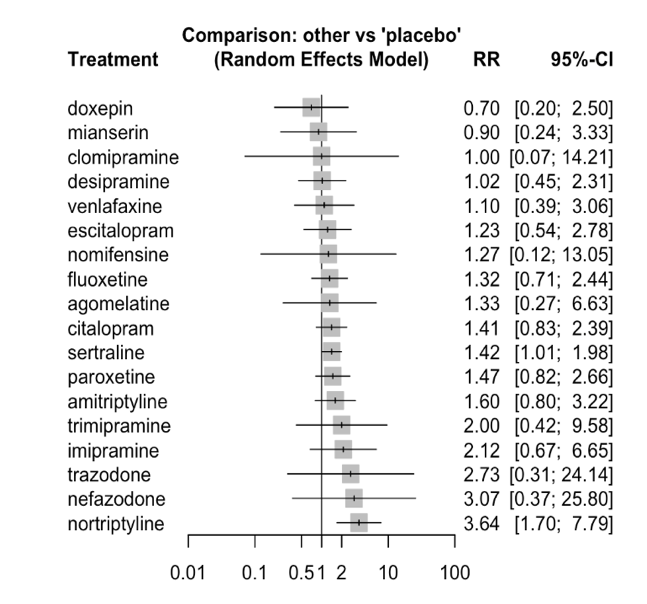 | τ^2^=0  I^2^=0% | Global:  P=0.9888  Local (SIDE):  0/21 |
| Only RCTs with a formal diagnosis of MDD (post-hoc) | N=69, n=5392   |  | tau^2=0  I^2=0 % (0 % to 30.73 %) | Global approach:  p=0.9968  Local approach (SIDE): 0/29 |
| Excluding RCTs with a sample size < 50 (post-hoc) | N=27, n=1989   |  | tau^2=0  I^2=0 % (0 % to 51.09 %) | Global approach:  p=0.8587  Local approach (SIDE): 0/9 |
| Excluding RCTs where efficacy on depressive symptoms was not the primary study aim | N=68, n=4991   |  | tau^2=0  I^2=0 % (0 % to 30.95 %) | Global approach:  p=0.9760  Local approach (SIDE): 0/29 |
| Excluding placebo-controlled trials | N=18, n=1196   |  | tau^2=0  I^2=0 % (0 % to 62.37 %) | Global approach:  p=0.9480  Local approach (SIDE): 0/14 |
| Excluding trials recruiting individuals with minor and minor-to-moderate severity of illness | N=55  n=4291  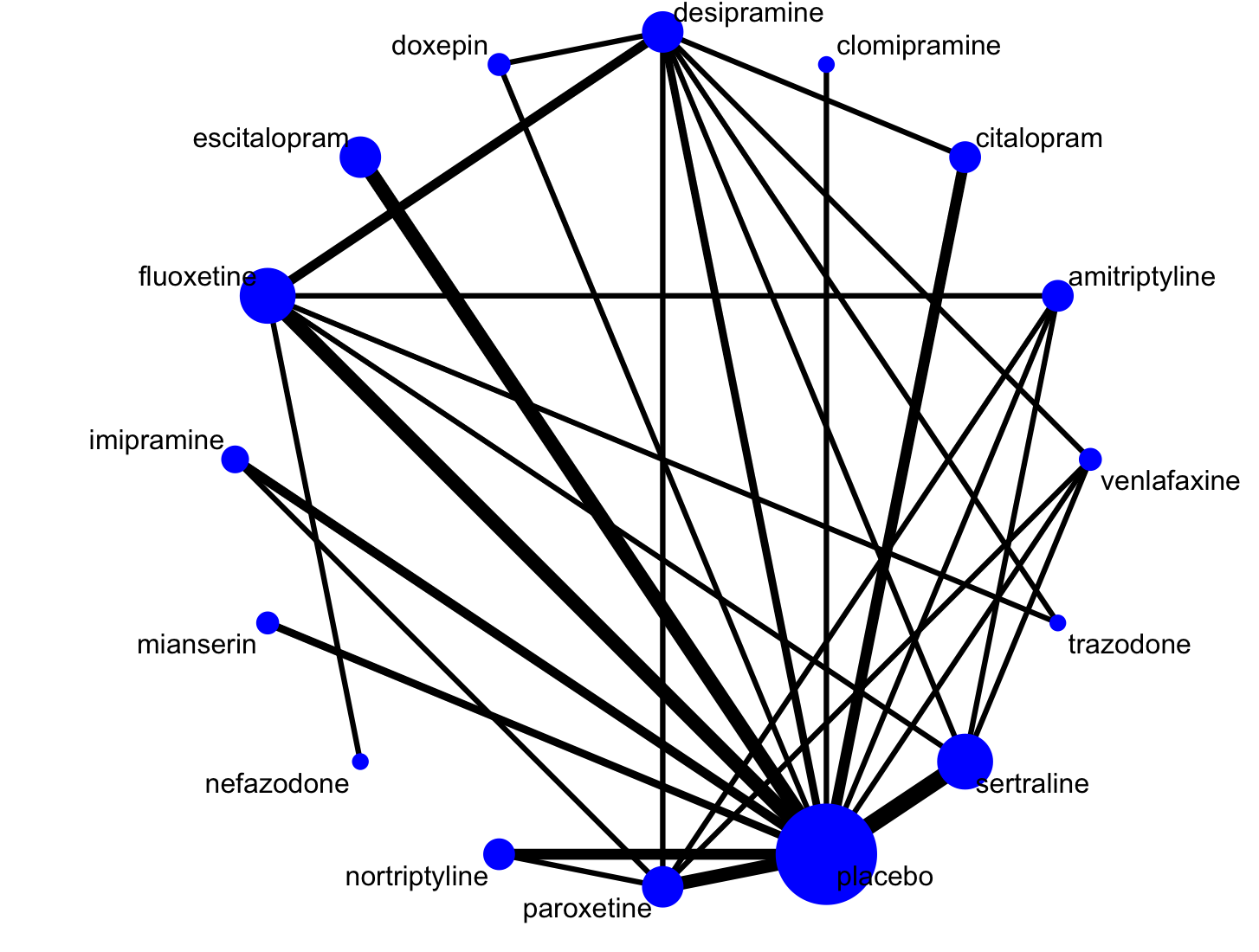 | 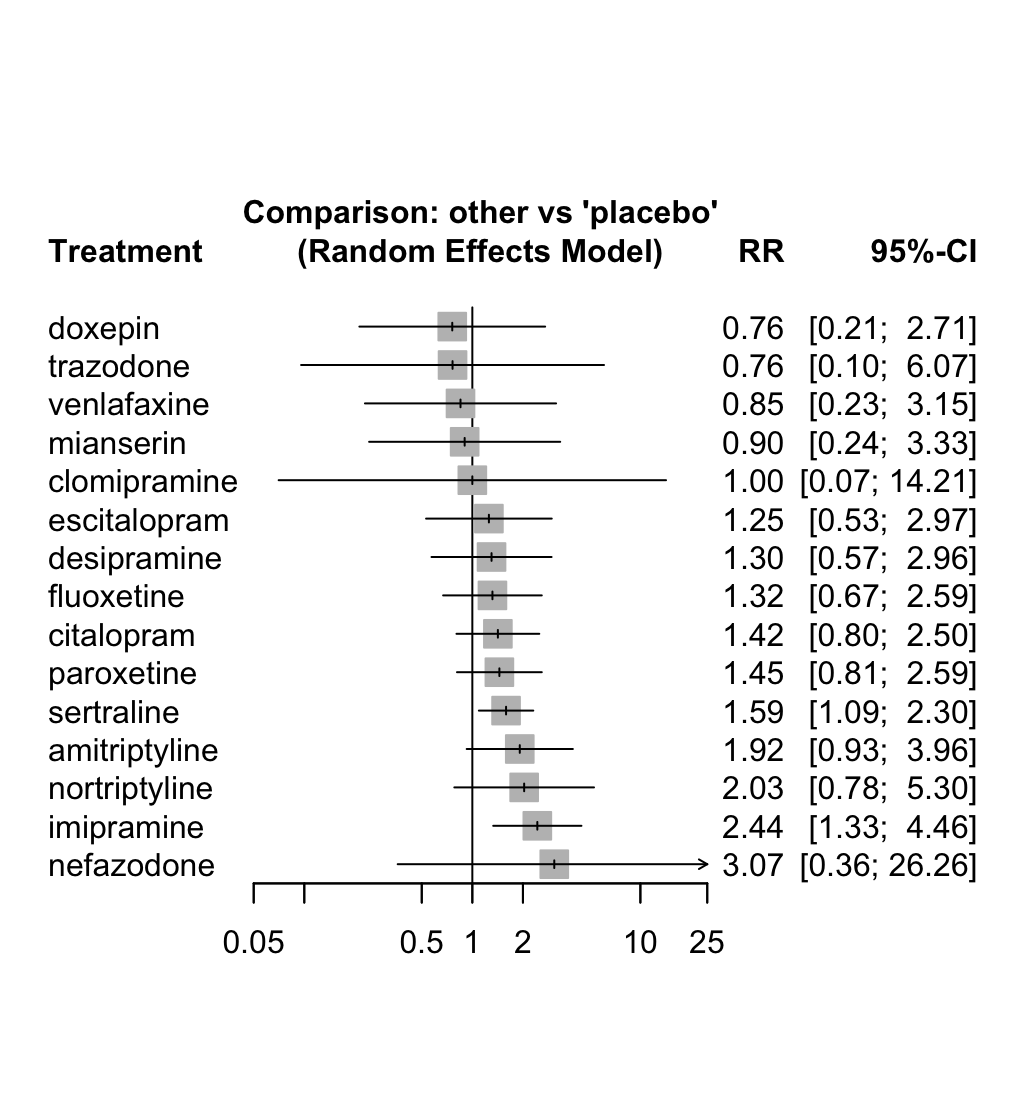 | tau^2=0  I^2=0 % (0 % to 33.84 %) | Global approach:  p=0.9869  Local approach (SIDE): 0/26 |

**Subgroup analyses (tolerability)**

| **Analysis** | **Network map** | **Forest plot** | **Heterogeneity** | **Consistency** |
| --- | --- | --- | --- | --- |
| Circulatory System Diseases | N=11, n=1971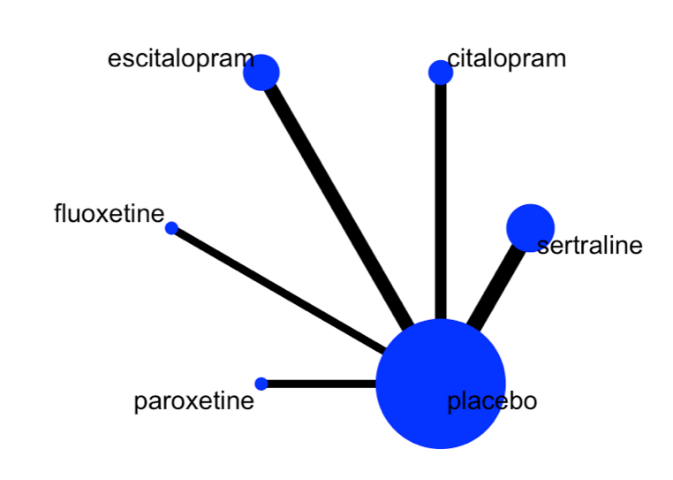 | 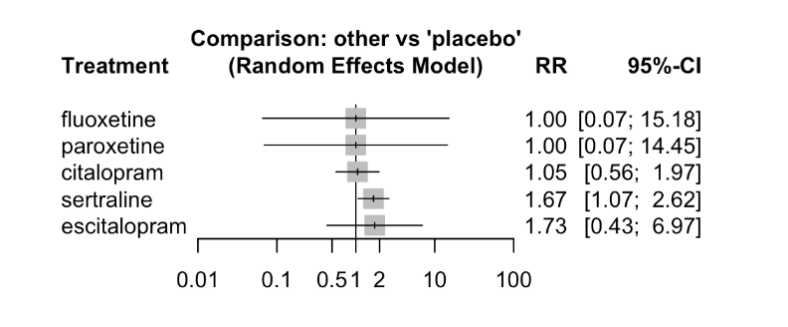 | τ^2^=0  I^2^=0% | Global approach:  NA  Local approach (SIDE):  NA |
| Endocrine,  Nutritional, Metabolic Diseases | N=9, n=558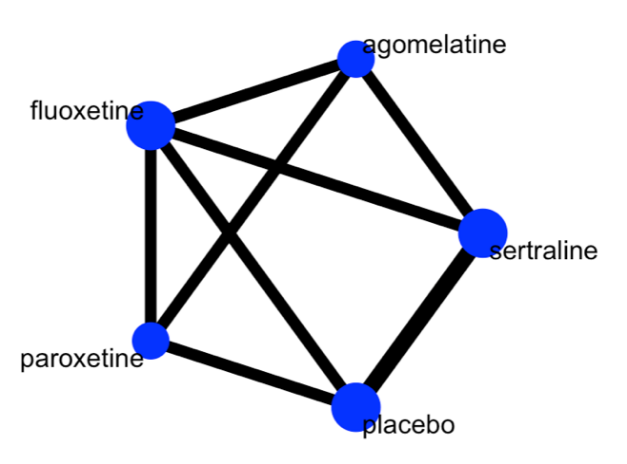 | 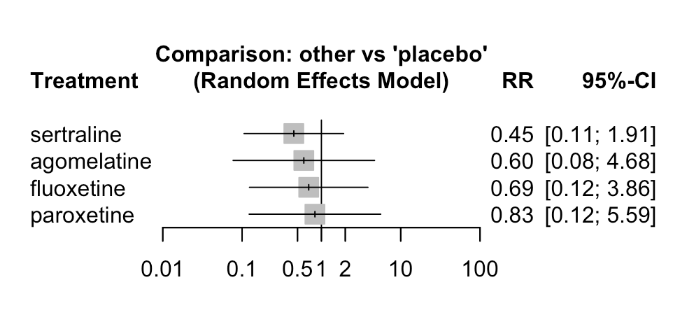 | τ^2^=0  I^2^=0% | Global approach:  p=0.9905  Local approach (SIDE):  0/8 |
| Infectious Diseases | N=7. N=443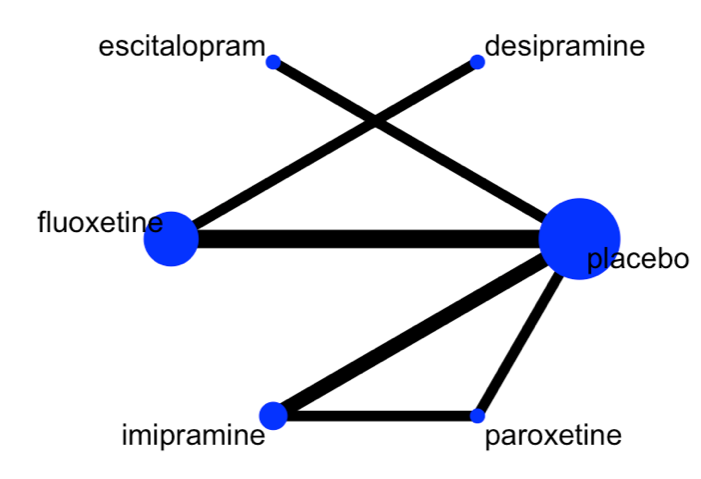 | 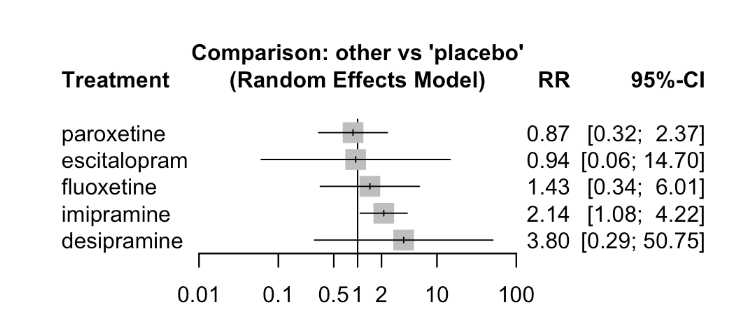 | τ^2^=0  I^2^=0% | Global approach:  p=0.7682  Local approach (SIDE):  0/2 |
| Nervous System Diseases | N=23, n=1150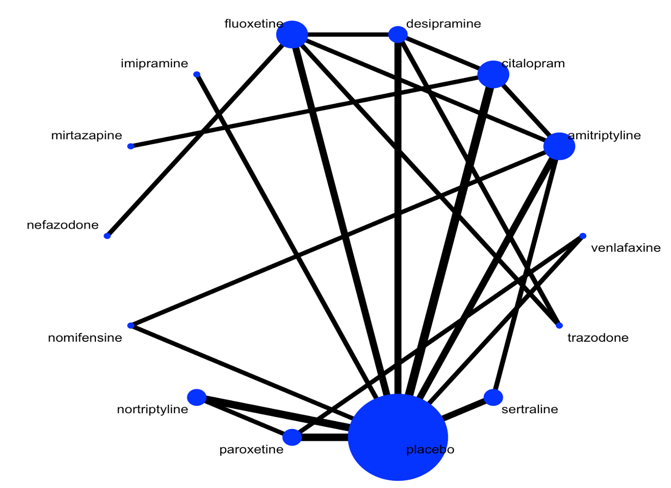 | 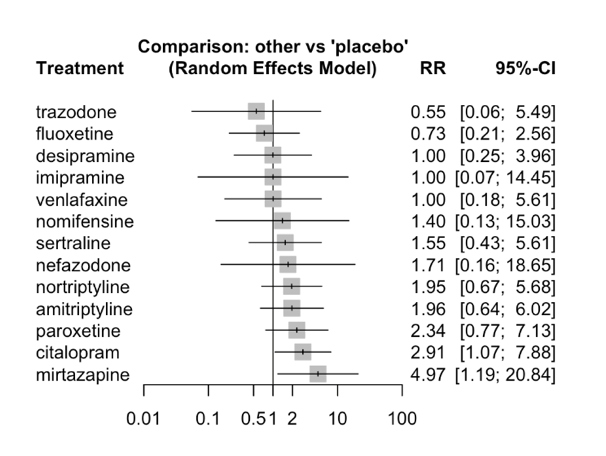 | τ^2^=0  I^2^=0% | Global approach:  p=0.9917  Local approach (SIDE):  0/19 |
| Neurocognitive Disorders | 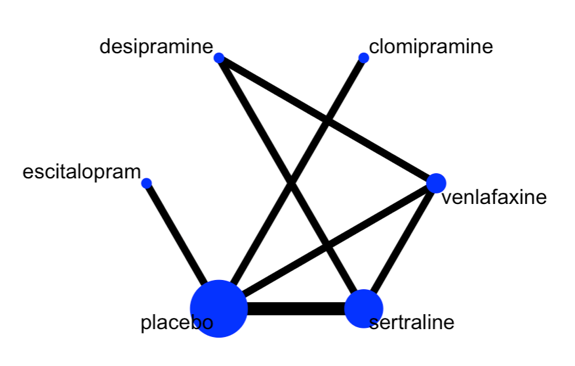N=7, n=504 | 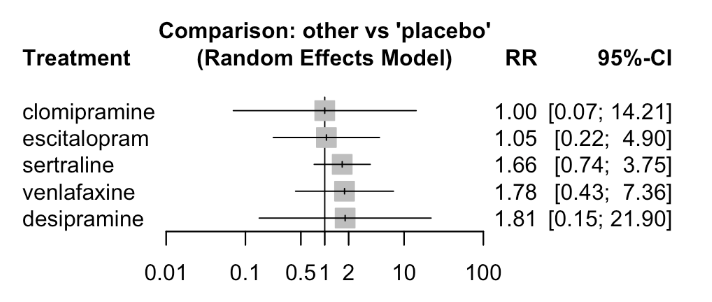 | τ^2^=0  I^2^=0% | Global approach:  p=0.9525  Local approach (SIDE):  0/5 |
| Oncological Diseases | 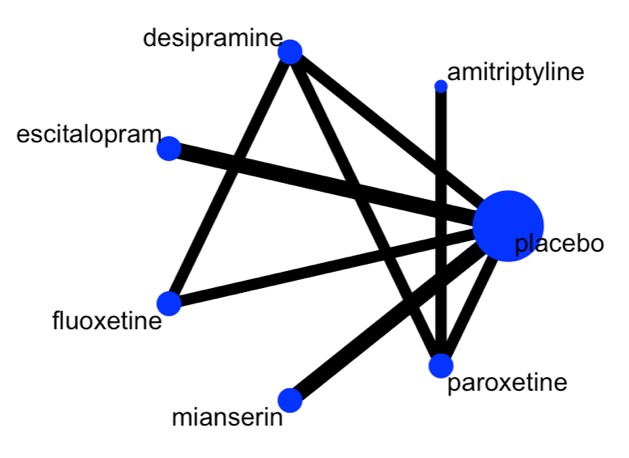N=8, n=602 | 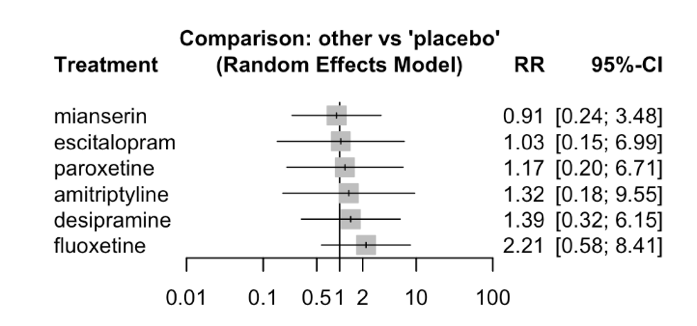 | τ^2^=0.0407  I^2^=3.77% | Global approach:  p=0.2346  Local approach (SIDE):  0/5 |
| Renal Diseases | 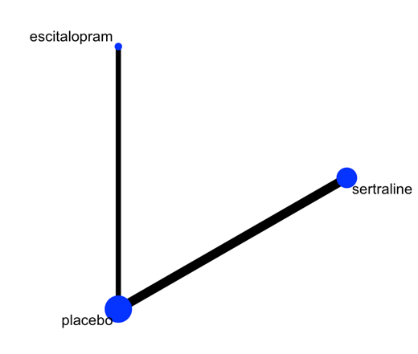N=4, n=315 | 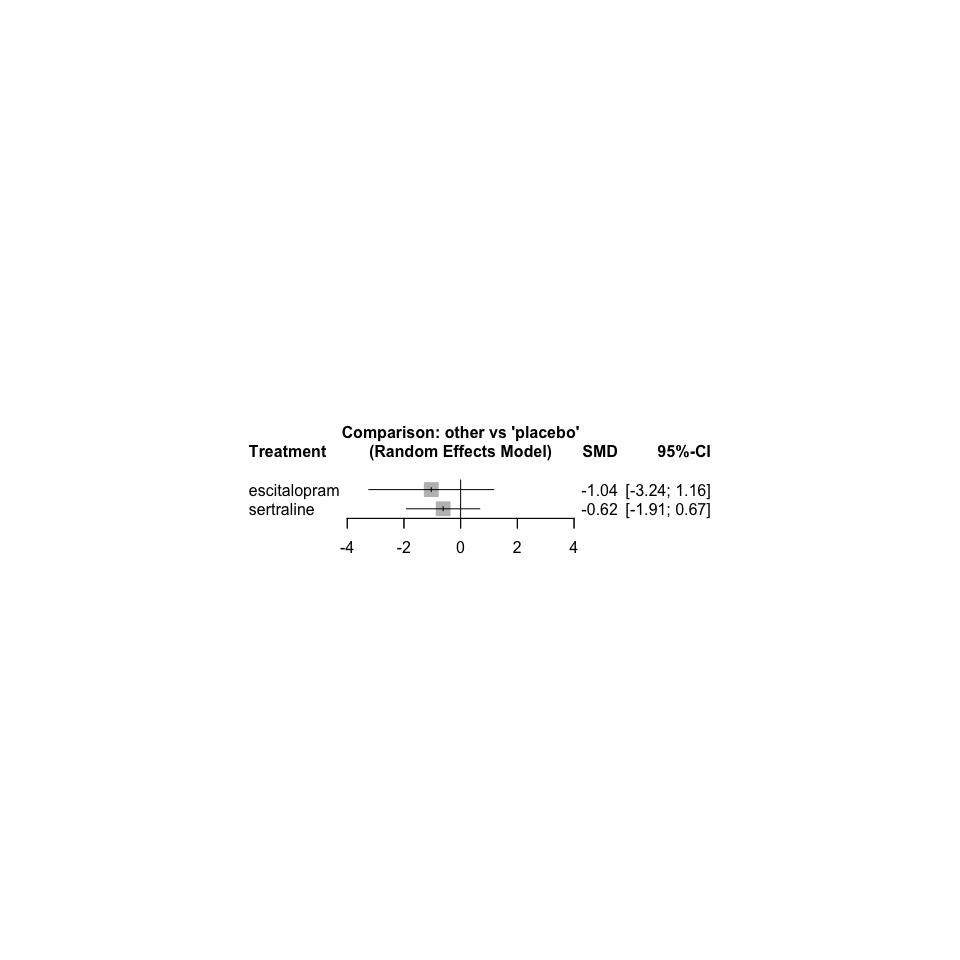 | τ^2^=1.1832  I^2^=92.41% | Global approach:  NA  Local approach (SIDE): NA |
| Respiratory Diseases | 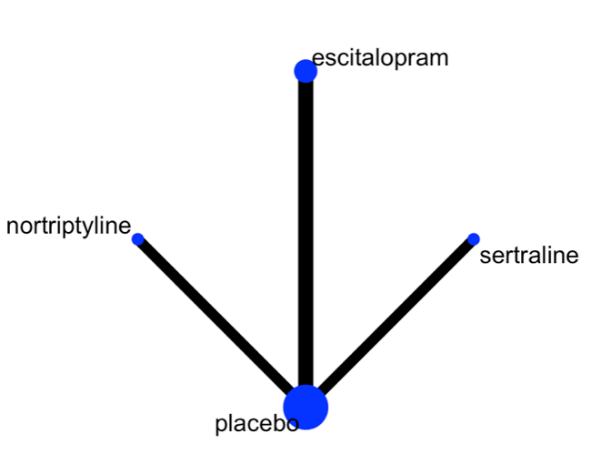N=4, n=208 | 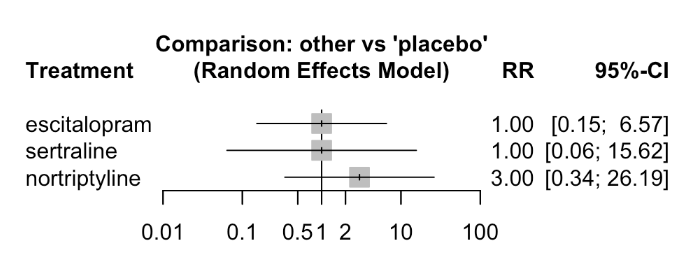 | τ^2^=0  I^2^=0% | Global approach:  NA  Local approach (SIDE):  NA |
| Hiv/Aids | N=7, n=443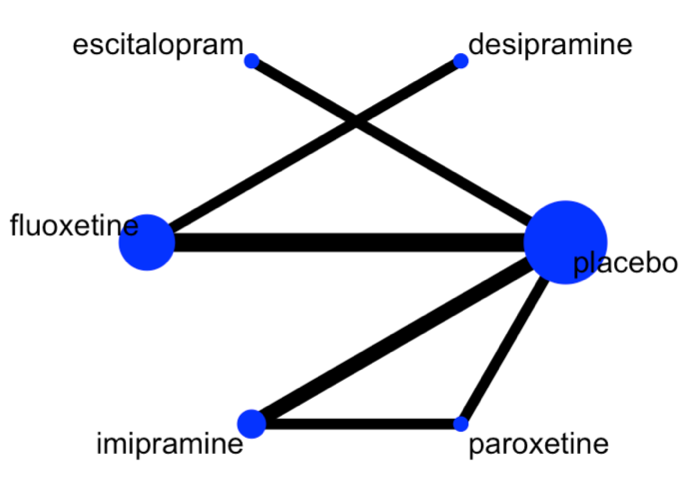 | 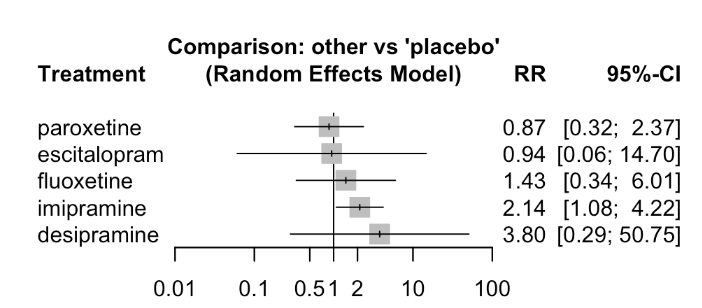 | τ^2^=0  I^2^=0% | Global approach:  P=0.7682  Local approach (SIDE):  0/2 |
| Diabetes | N=7, n=456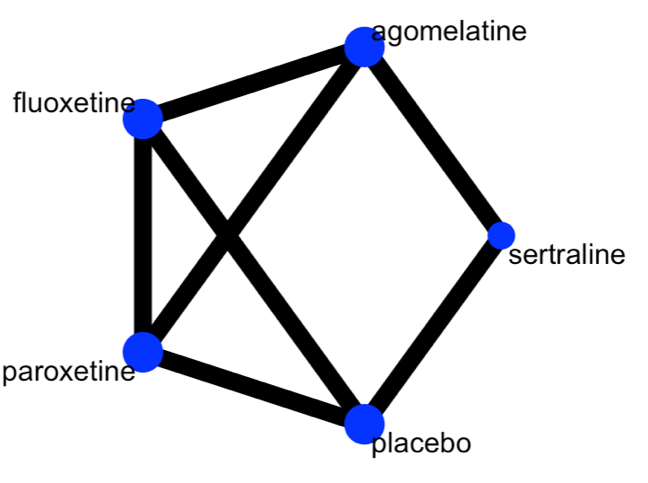9 | 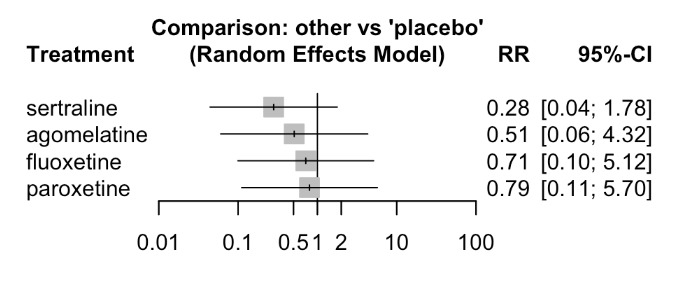 | τ^2^=0  I^2^=0% | Global approach:  P=0.9182  Local approach (SIDE):  0/7 |
| Post-Stroke | 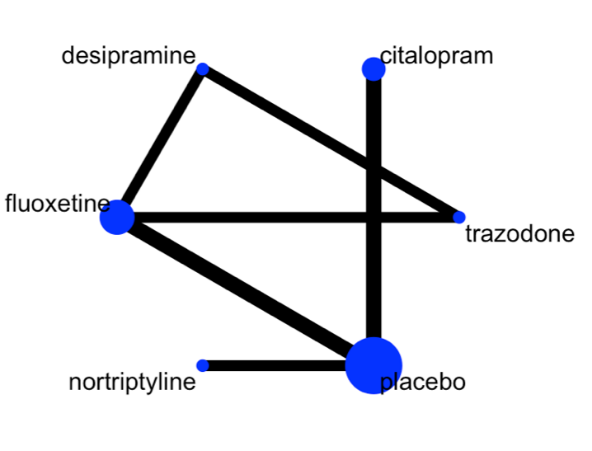N=6, n=396 | 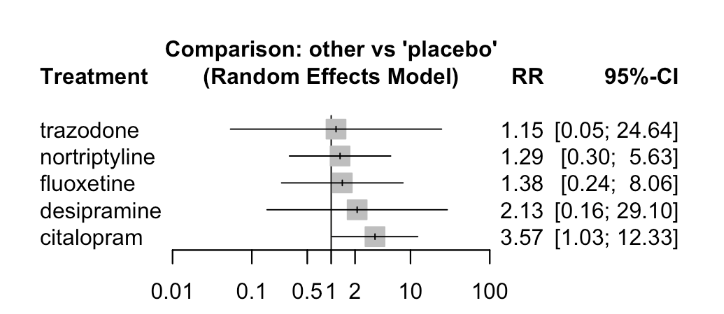 | τ^2^=0  I^2^=0% | Global approach:  NA  Local approach (SIDE):  NA |
| Post Myocardial Infarction | N=7, n=1107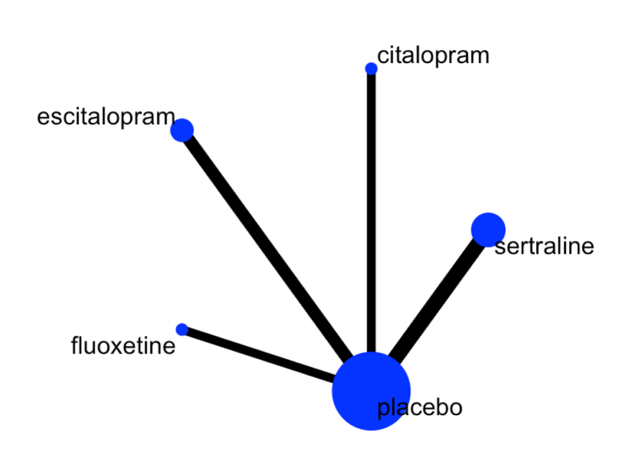 | 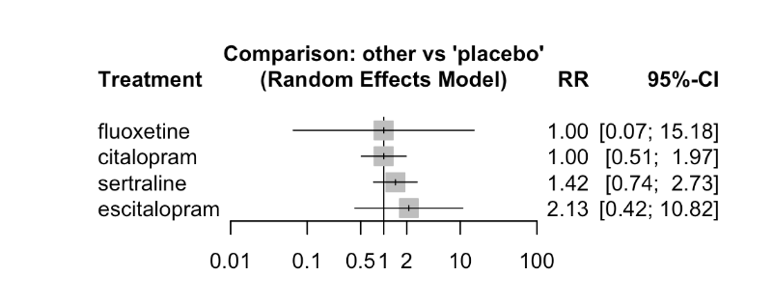 | τ^2^=0  I^2^=0% | Global approach:  NA  Local approach (SIDE):  NA |

**I. – Secondary analysis: Efficacy by drug classes**

**Transitivity assessment**


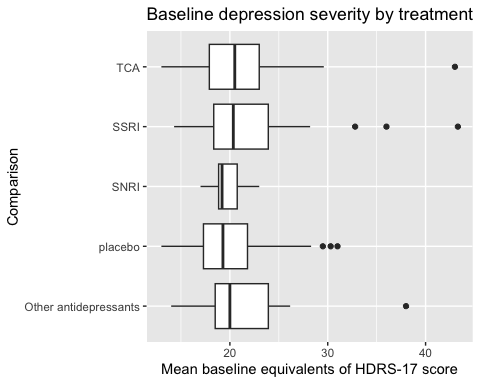


Kruskal-Wallis chi-squared = 3.1451, df = 4, p-value = 0.5338


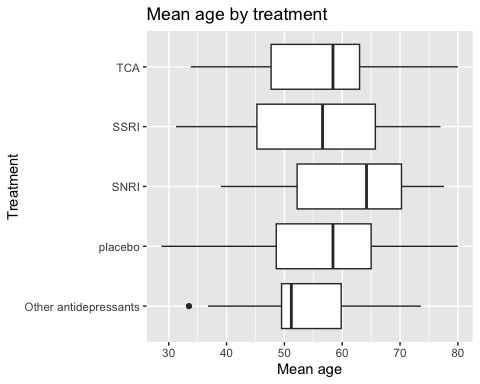


Kruskal-Wallis chi-squared = 2.7728, df = 4, p-value = 0.5965


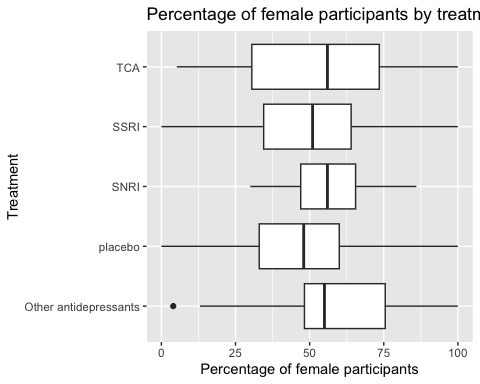


Kruskal-Wallis chi-squared = 3.0759, df = 4, p-value = 0.5452


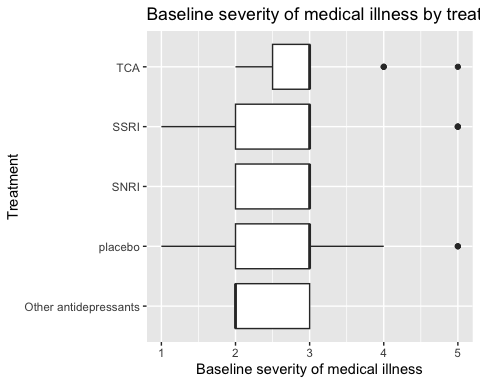


Kruskal-Wallis chi-squared = 5.5764, df = 4, p-value = 0.2331


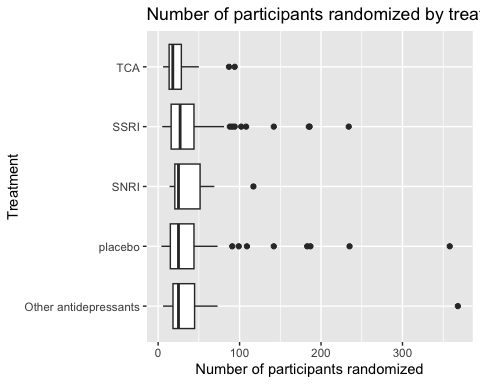


Kruskal-Wallis chi-squared = 5.1662, df = 4, p-value = 0.2707


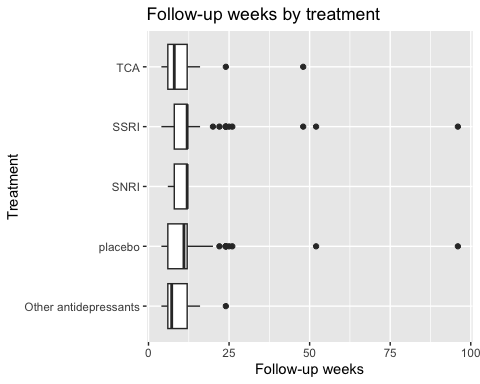


Kruskal-Wallis chi-squared = 7.4234, df = 4, p-value = 0.1151

**Secondary analysis: Efficacy by drug classes**

Characteristics of the network

Number of treatments:

5

Number of studies:

99

Number of individuals included:

8245

Number of individuals contributing to this analysis:

7573

Number of individuals randomized to each treatment:

Treatment name N. individuals randomized
1 Other antidepressants 807
2 placebo 3343
3 SNRI 300
4 SSRI 3097
5 TCA 698

**Pairwise meta-analysis**
Standardized mean differences below 0 favor the first treatment of the comparison.

**
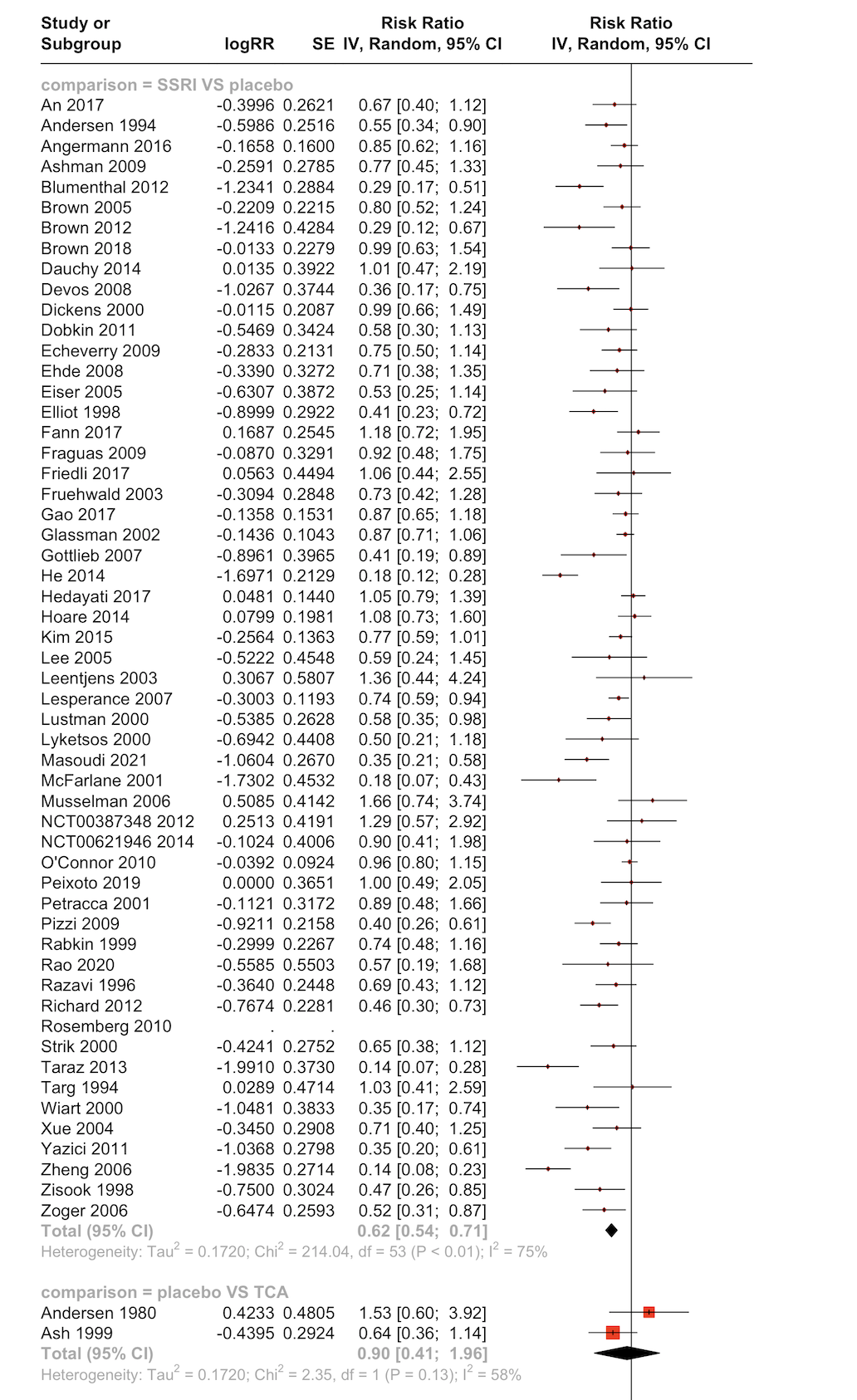
**

**
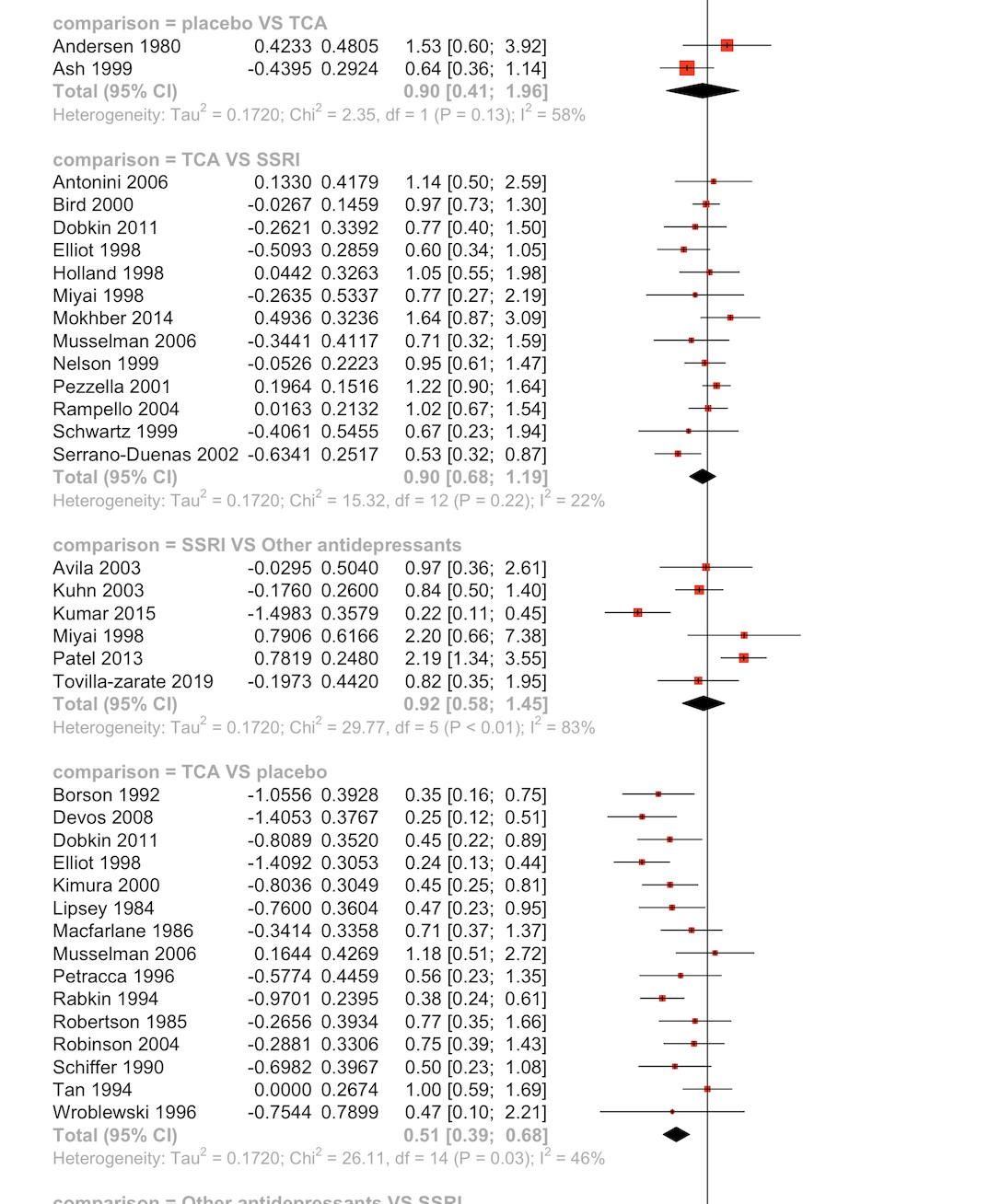
**

**
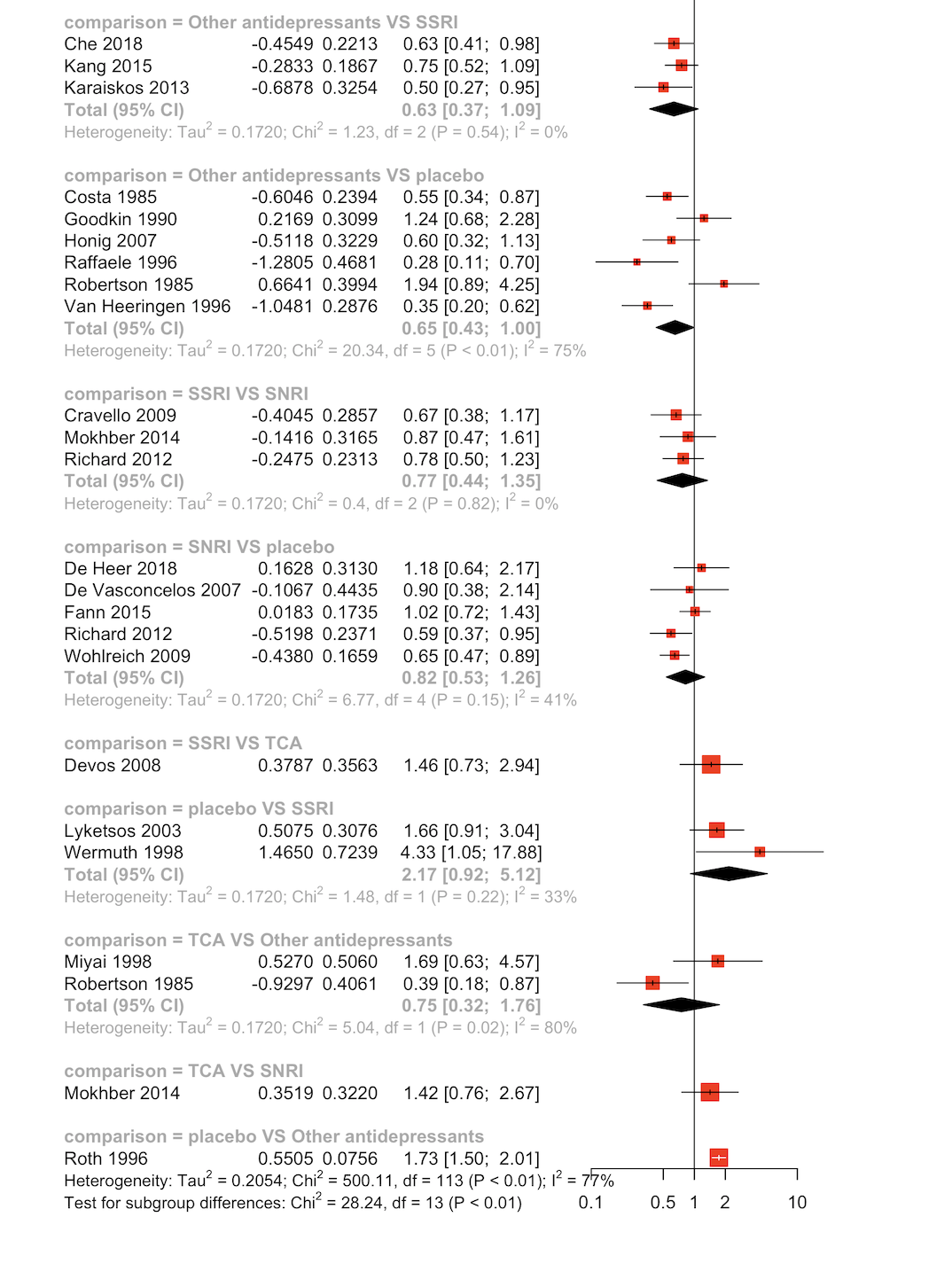
**

**Forest plot**

Placebo was used as a common comparator. SMDs below 0 favor the treatment over the common comparator.


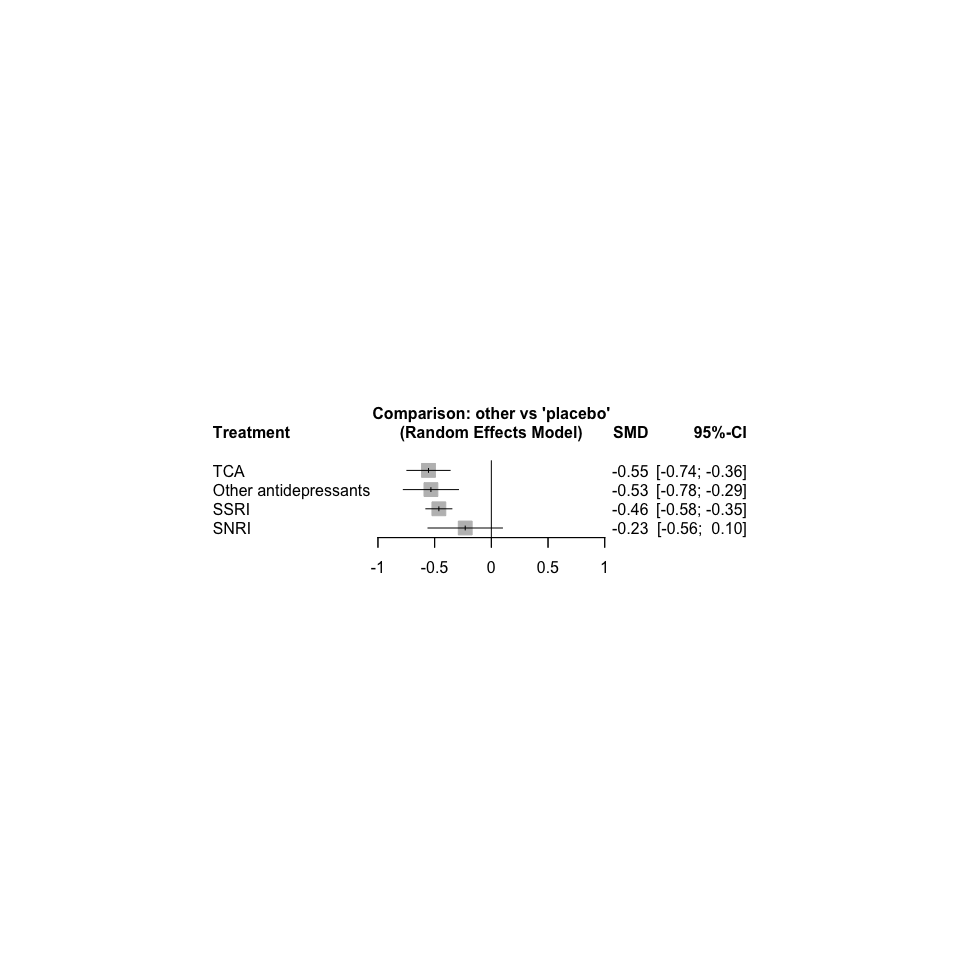


**Network map**

The thickness of lines is proportional to the number of studies comparing the two treatments and the size of circles is proportional to the number of individuals for each treatment.

**Netleague table**

Standardized mean differences (SMDs) and 95% confidence intervals (CIs) are reported. Results of the network meta-analysis are reported in the lower left part of the table and results from the pairwise meta-analysis are reported in the upper right part of the table. SMDs lower than 1 favour the column-defining treatment.

*Global heterogeneity*

We interpreted tau^2 as follows: heterogeneity low with tau^2≤0.010, moderate with 0.010<tau^2≤0.242, high with tau^2>0.242, and I^2 statistics as follows: not important (0%-40%), moderate (30%-60%), substantial (50%-90%), considerable (75%-100%).

tau^2= 0.1381; tau= 0.3716

I^2= 68.87 % (61.93 % to 74.55 %)

*Consistency: global approach*

Q statistic to assess consistency under the assumption of a full design-by-treatment interaction random effects model

Q df p-value tau.within tau2.within
Between designs 11.86 13 0.5395 0.3807 0.1449

*Consistency: local approach*

Separate indirect from direct evidence (SIDE) using back-calculation method

Random effects model:

 comparison k prop nma direct indir. Diff z p-value
 placebo:Other antidepressants 7 0.48 0.53 0.46 0.60 -0.15 -0.60 0.5513
 Other antidepressants:SSRI 9 0.55 -0.07 -0.14 0.02 -0.16 -0.65 0.5154
 Other antidepressants:TCA 2 0.13 0.02 0.30 -0.02 0.32 0.71 0.4762
 placebo:SNRI 5 0.68 0.23 0.20 0.30 -0.10 -0.28 0.7817
 placebo:SSRI 56 0.85 0.46 0.48 0.39 0.09 0.53 0.5947
 placebo:TCA 17 0.59 0.55 0.58 0.52 0.06 0.32 0.7488
 SNRI:SSRI 3 0.41 0.23 0.27 0.21 0.06 0.17 0.8673
 SNRI:TCA 1 0.15 0.32 -0.35 0.44 -0.79 -1.49 0.1371
 SSRI:TCA 14 0.57 0.09 0.12 0.05 0.06 0.32 0.7466

Legend:
 comparison - Treatment comparison
 k - Number of studies providing direct evidence
 prop - Direct evidence proportion
 nma - Estimated treatment effect (SMD) in network meta-analysis
 direct - Estimated treatment effect (SMD) derived from direct evidence
 indir. - Estimated treatment effect (SMD) derived from indirect evidence
 Diff - Difference between direct and indirect treatment estimates
 z - z-value of test for disagreement (direct versus indirect)
 p-value - p-value of test for disagreement (direct versus indirect)

**Netrank**

Higher p-score values indicate higher ranking of treatments

P-score
TCA 0.8350
Other antidepressants 0.7721
SSRI 0.5932
SNRI 0.2786
placebo 0.0211

**J. – Secondary analysis: Tolerability by drug classes**

**Characteristics of the network**

Number of treatments:

5

Number of studies:

79

Number of individuals included:

6039

Number of individuals randomized to each treatment:

Treatment name N. individuals randomized
1 Other antidepressants 302
2 placebo 2345

3 SNRI 68
4 SSRI 2634

5 TCA 690

**Pairwise meta-analysis**
Risk Ratio below 1 favor the first treatment of the comparison.

**Forest plot**

Placebo was used as a common comparator. SMDs below 0 favor the treatment over the common comparator.

**Network map**

The thickness of lines is proportional to the number of studies comparing the two treatments and the size of circles is proportional to the number of individuals for each treatment.

**Netleague table**

Standardized mean differences (SMDs) and 95% confidence intervals (CIs) are reported. Results of the network meta-analysis are reported in the lower left part of the table and results from the pairwise meta-analysis are reported in the upper right part of the table. SMDs lower than 1 favour the column-defining treatment.

*Global heterogeneity*

We interpreted tau^2 as follows: heterogeneity low with tau^2≤0.010, moderate with 0.010<tau^2≤0.242, high with tau^2>0.242, and I^2 statistics as follows: not important (0%-40%), moderate (30%-60%), substantial (50%-90%), considerable (75%-100%).

tau^2= 0; tau=0

I^2= 0 % (0 % to 26.51 %)

*Consistency: global approach*

Q statistic to assess consistency under the assumption of a full design-by-treatment interaction random effects model

Q df p-value tau.within tau2.within
Between designs 3.62 12 0.9893 0 0

*Consistency: local approach*

Separate indirect from direct evidence (SIDE) using back-calculation method
Random effects model:
 comparison k prop nma direct indir. RoR z p-value
 placebo:Other antidepressants 4 0.35 0.61 0.85 0.51 1.67 0.78 0.4353
 Other antidepressants:SSRI 6 0.63 1.20 1.46 0.86 1.70 0.84 0.4016
 Other antidepressants:TCA 2 0.16 0.89 0.67 0.94 0.71 -0.38 0.7028
 placebo:SNRI 2 0.65 0.89 0.67 1.52 0.44 -0.76 0.4453
 placebo:SSRI 47 0.85 0.73 0.72 0.79 0.91 -0.30 0.7657
 placebo:TCA 15 0.49 0.54 0.61 0.49 1.24 0.69 0.4929
 SNRI:SSRI 2 0.57 0.82 0.51 1.54 0.33 -1.06 0.2873
 SNRI:TCA 1 0.15 0.61 0.95 0.57 1.67 0.34 0.7301
 SSRI:TCA 15 0.72 0.74 0.72 0.82 0.87 -0.43 0.6658

Legend:
 comparison - Treatment comparison
 k - Number of studies providing direct evidence
 prop - Direct evidence proportion
 nma - Estimated treatment effect (RR) in network meta-analysis
 direct - Estimated treatment effect (RR) derived from direct evidence
 indir. - Estimated treatment effect (RR) derived from indirect evidence
 RoR - Ratio of Ratios (direct versus indirect)
 z - z-value of test for disagreement (direct versus indirect)
 p-value - p-value of test for disagreement (direct versus indirect)

**Netrank**

Higher p-score values indicate higher ranking of treatments

P-score
placebo 0.8824
SNRI 0.6547
SSRI 0.5143
Other antidepressants 0.3090
TCA 0.1396

**K. Secondary outcomes:**

**Response**

The definition provided by each study was considered. In case these data were not available, the number was imputed according to commonly used cut-off scores at validated rating scales measuring psychopathology (namely, a baseline score decrease of HAMD-21 ≥ 47%, HAMD-17 ≥ 57%, MADRS ≥ 46%; BDI ≥ 47%) (1) using a validated methodology (2)

1. Riedel M, Möller HJ, Obermeier M, Schennach-Wolff R, Bauer M, Adli M, et al. Response and remission criteria in major depression--a validation of current practice. Journal of psychiatric research. 2010;44(15):1063-8
2. Furukawa TA, Cipriani A, Barbui C, Brambilla P, Watanabe N. Imputing response rates from means and standard deviations in meta-analyses. Int Clin Psychopharmacol. 2005;20(1):49-52.

**Characteristics of the network**

Number of treatments:

22

Number of studies:

90

Number of individuals included:

7612

Number of individuals contributing to this analysis:

7612

Number of individuals randomized to each treatment:

Treatment name N. individuals randomized
1 agomelatine 145
2 amitriptyline 260
3 citalopram 456
4 clomipramine 12
5 desipramine 57
6 dothiepin 25
7 duloxetine 117
8 escitalopram 540
9 fluoxetine 535
10 imipramine 119
11 lofepramine 32
12 mianserin 101
13 mirtazapine 109
14 moclobemide 368
15 nefazodone 9
16 nortriptyline 136
17 paroxetine 558
18 placebo 3012
19 sertraline 848
20 trazodone 22
21 venlafaxine 128
22 vortioxetine 23

**Pairwise meta-analysis**
Standardized mean differences below 0 favor the first treatment of the comparison.

**Forest plot** Placebo was used as a common comparator. SMDs below 0 favor the treatment over the common comparator.

**Network map** The thickness of lines is proportional to the number of studies comparing the two treatments and the size of circles is proportional to the number of individuals for each treatment.

**Netleague table** Risk Ratio and 95% confidence intervals (CIs) are reported. Results of the network meta-analysis are reported in the lower left part of the table and results from the pairwise meta-analysis are reported in the upper right part of the table. RRs higher than 1 favour the column-defining treatment.

*Global heterogeneity*

We interpreted tau^2 as follows: heterogeneity low with tau^2≤0.010, moderate with 0.010<tau^2≤0.242, high with tau^2>0.242, and I^2 statistics as follows: not important (0%-40%), moderate (30%-60%), substantial (50%-90%), considerable (75%-100%).

tau^2= 0.0267 ; tau= 0.1635

I^2= 27.67 % ( 3.21 % to 45.94 %)

*Consistency: global approach*

Q statistic to assess consistency under the assumption of a full design-by-treatment interaction

Q df p-value tau.within tau2.within
Between designs 25.92 23 0.3047 0.1651 0.0273

*Consistency: local approach*

Separate indirect from direct evidence (SIDE) using back-calculation method

Random effects model:

 comparison k prop nma direct indir. RoR z p-value
 **agomelatine:escitalopram 1 0.03 1.28 0.08 1.39 0.06 -2.75 0.0059**
 agomelatine:fluoxetine 1 0.29 1.09 1.18 1.06 1.11 0.31 0.7541
 agomelatine:paroxetine 1 0.47 0.99 1.04 0.94 1.11 0.33 0.7400
 agomelatine:sertraline 1 0.49 1.23 1.27 1.19 1.06 0.20 0.8390
 amitriptyline:citalopram 1 0.51 1.00 1.00 1.00 1.00 0.00 0.9968
 amitriptyline:paroxetine 2 0.63 0.82 0.87 0.74 1.17 0.57 0.5676
 placebo:amitriptyline 1 0.01 0.78 2.11 0.77 2.73 0.83 0.4039
 amitriptyline:sertraline 1 0.19 1.02 0.85 1.06 0.80 -0.56 0.5766
 citalopram:desipramine 1 0.40 0.97 0.85 1.07 0.80 -0.57 0.5690
 citalopram:fluoxetine 1 0.17 0.90 0.92 0.90 1.03 0.08 0.9382
 citalopram:mirtazapine 1 0.17 0.86 0.52 0.96 0.54 -1.27 0.2055
 placebo:citalopram 7 0.71 0.78 0.74 0.90 0.81 -0.98 0.3288
 desipramine:fluoxetine 2 0.29 0.93 0.94 0.92 1.02 0.05 0.9576
 desipramine:paroxetine 1 0.17 0.84 1.18 0.79 1.50 0.73 0.4663
 placebo:desipramine 3 0.49 0.76 0.86 0.68 1.28 0.65 0.5153
 escitalopram:mirtazapine 1 0.71 0.81 0.94 0.57 1.63 1.43 0.1526
 **placebo:escitalopram 10 0.93 0.83 0.88 0.42 2.09 2.25 0.0247** fluoxetine:paroxetine 2 0.21 0.91 0.66 0.99 0.67 -1.38 0.1682
 placebo:fluoxetine 12 0.69 0.71 0.66 0.82 0.81 -1.12 0.2617
 fluoxetine:venlafaxine 1 0.25 1.17 1.18 1.17 1.01 0.02 0.9841
 imipramine:paroxetine 1 0.24 1.25 1.44 1.20 1.20 0.44 0.6607
 placebo:imipramine 4 0.92 0.51 0.53 0.37 1.44 0.65 0.5172
 placebo:mirtazapine 1 0.22 0.68 0.56 0.71 0.79 -0.61 0.5398
 nortriptyline:paroxetine 3 0.77 1.19 1.09 1.61 0.68 -1.16 0.2476
 placebo:nortriptyline 3 0.36 0.54 0.40 0.63 0.64 -1.35 0.1774
 placebo:paroxetine 8 0.43 0.64 0.75 0.57 1.31 1.33 0.1837
 paroxetine:venlafaxine 1 0.38 1.29 1.16 1.38 0.84 -0.48 0.6320
 placebo:sertraline 16 0.86 0.80 0.80 0.76 1.05 0.19 0.8484
 placebo:venlafaxine 2 0.70 0.83 0.86 0.77 1.11 0.30 0.7647

Legend:
 comparison - Treatment comparison
 k - Number of studies providing direct evidence
 prop - Direct evidence proportion
 nma - Estimated treatment effect (RR) in network meta-analysis
 direct - Estimated treatment effect (RR) derived from direct evidence
 indir. - Estimated treatment effect (RR) derived from indirect evidence
 RoR - Ratio of Ratios (direct versus indirect)
 z - z-value of test for disagreement (direct versus indirect)
 p-value - p-value of test for disagreement (direct versus indirect)

**Netrank** Higher p-score values indicate higher ranking of treatments

P-score
imipramine 0.8830
nortriptyline 0.8523
mianserin 0.7602
paroxetine 0.6960
moclobemide 0.6883
agomelatine 0.6720
duloxetine 0.6625
clomipramine 0.6374
mirtazapine 0.6190
fluoxetine 0.5690
desipramine 0.4685
amitriptyline 0.4254
citalopram 0.4228
sertraline 0.3976
nefazodone 0.3963
venlafaxine 0.3587
trazodone 0.3434
escitalopram 0.3403
vortioxetine 0.3249
lofepramine 0.2768
placebo 0.1483
dothiepin 0.0574

**Secondary outcome: Remission**

The definition provided by each study was considered. In case these data were not available, the number was imputed according to commonly used cut-off scores at validated rating scales measuring psychopathology (namely, a baseline score decrease of HAMD-21 ≤ 7

HAMD-17 ≤6, MADRS ≤7; BDI ≤ 12) (1) using a validated methodology (2)

1. Riedel M, Möller HJ, Obermeier M, Schennach-Wolff R, Bauer M, Adli M, et al. Response and remission criteria in major depression--a validation of current practice. Journal of psychiatric research. 2010;44(15):1063-8
2. Furukawa TA, Cipriani A, Barbui C, Brambilla P, Watanabe N. Imputing response rates from means and standard deviations in meta-analyses. Int Clin Psychopharmacol. 2005;20(1):49-52.

**Characteristics of the network**

Number of treatments:

22

Number of studies:

78

Number of individuals included:

7246

Number of individuals contributing to this analysis:

7246

Number of individuals randomized to each treatment:

Treatment name N. individuals randomized
1 agomelatine 104
2 amitriptyline 196
3 citalopram 412
4 clomipramine 12
5 desipramine 51
6 dothiepin 25
7 duloxetine 117
8 escitalopram 493
9 fluoxetine 479
10 imipramine 105
11 lofepramine 32
12 mianserin 101
13 mirtazapine 109
14 moclobemide 368
15 nefazodone 9
16 nortriptyline 78
17 paroxetine 531
18 placebo 2956
19 sertraline 881
20 trazodone 22
21 venlafaxine 142
22 vortioxetine 23

**Pairwise meta-analysis**
Standardized mean differences below 0 favor the first treatment of the comparison.

**Forest plot** Placebo was used as a common comparator. SMDs below 0 favor the treatment over the common comparator.

**Network map** The thickness of lines is proportional to the number of studies comparing the two treatments and the size of circles is proportional to the number of individuals for each treatment.

**Netleague table** Risk Ratio and 95% confidence intervals (CIs) are reported. Results of the network meta-analysis are reported in the lower left part of the table and results from the pairwise meta-analysis are reported in the upper right part of the table. RRs higher than 1 favour the column-defining treatment.

*Global heterogeneity*

We interpreted tau^2 as follows: heterogeneity low with tau^2≤0.010, moderate with 0.010<tau^2≤0.242, high with tau^2>0.242, and I^2 statistics as follows: not important (0%-40%), moderate (30%-60%), substantial (50%-90%), considerable (75%-100%).

tau^2= 0.0177 ; tau= 0.1331

I^2= 10.24 % ( 0 % to 34.78 %)

*Consistency: global approach*

Q statistic to assess consistency under the assumption of a full design-by-treatment interaction

Q df p-value tau.within tau2.within
Between designs 9.15 18 0.9563 0.2210 0.0489

*Consistency: local approach*

Separate indirect from direct evidence (SIDE) using back-calculation method

Random effects model:

 comparison k prop nma direct indir. RoR z p-value
 agomelatine:fluoxetine 1 0.37 1.15 1.11 1.17 0.95 -0.11 0.9151
 agomelatine:paroxetine 1 0.75 1.09 1.10 1.04 1.06 0.11 0.9151
 amitriptyline:sertraline 1 0.17 1.07 0.89 1.12 0.80 -0.41 0.6819
 citalopram:desipramine 1 0.58 0.75 0.76 0.74 1.01 0.03 0.9758
 citalopram:fluoxetine 1 0.22 1.06 1.00 1.07 0.93 -0.18 0.8595
 citalopram:mirtazapine 1 0.07 1.26 1.64 1.23 1.33 0.23 0.8219
 placebo:citalopram 7 0.83 0.68 0.66 0.78 0.85 -0.47 0.6386
 desipramine:fluoxetine 2 0.17 1.41 1.01 1.50 0.68 -0.57 0.5676
 desipramine:paroxetine 1 0.19 1.33 1.97 1.21 1.63 0.71 0.4791
 placebo:desipramine 2 0.39 0.51 0.47 0.54 0.88 -0.25 0.7992
 escitalopram:mirtazapine 1 0.81 1.13 1.42 0.43 3.29 1.63 0.1023
 placebo:escitalopram 8 0.97 0.76 0.79 0.24 3.29 1.63 0.1023
 fluoxetine:paroxetine 2 0.17 0.95 0.85 0.96 0.88 -0.27 0.7849
 placebo:fluoxetine 9 0.72 0.72 0.71 0.72 0.99 -0.04 0.9673
 fluoxetine:venlafaxine 1 0.10 1.20 0.80 1.25 0.64 -0.68 0.4948
 imipramine:paroxetine 1 0.31 1.74 1.57 1.81 0.87 -0.29 0.7732
 placebo:imipramine 3 0.84 0.39 0.38 0.48 0.77 -0.46 0.6470
  **placebo:mirtazapine 1 0.16 0.86 0.21 1.12 0.19 -2.00 0.0453**
 nortriptyline:paroxetine 2 0.80 1.12 1.06 1.45 0.73 -0.67 0.5042
 placebo:nortriptyline 2 0.32 0.60 0.47 0.68 0.69 -0.80 0.4221
 placebo:paroxetine 9 0.55 0.68 0.69 0.66 1.04 0.14 0.8895
 paroxetine:venlafaxine 1 0.32 1.27 1.10 1.35 0.81 -0.48 0.6299
 placebo:sertraline 15 0.95 0.73 0.74 0.59 1.25 0.41 0.6819
 placebo:venlafaxine 3 0.88 0.86 0.91 0.58 1.58 0.93 0.3531

Legend:
 comparison - Treatment comparison
 k - Number of studies providing direct evidence
 prop - Direct evidence proportion
 nma - Estimated treatment effect (RR) in network meta-analysis
 direct - Estimated treatment effect (RR) derived from direct evidence
 indir. - Estimated treatment effect (RR) derived from indirect evidence
 RoR - Ratio of Ratios (direct versus indirect)
 z - z-value of test for disagreement (direct versus indirect)
 p-value - p-value of test for disagreement (direct versus indirect)

**Netrank** Higher p-score values indicate higher ranking of treatments

P-score
imipramine 0.9147
moclobemide 0.8525
desipramine 0.7711
mianserin 0.7323
nortriptyline 0.6519
duloxetine 0.6515
agomelatine 0.6173
clomipramine 0.5979
nefazodone 0.5752
citalopram 0.5447
paroxetine 0.5444
amitriptyline 0.5387
fluoxetine 0.4840
sertraline 0.4655
lofepramine 0.4414
escitalopram 0.4241
mirtazapine 0.3536
venlafaxine 0.3136
placebo 0.1866
trazodone 0.1736
vortioxetine 0.1023
dothiepin 0.0632

**Mean change in anxiety symptoms**

**Characteristics of the network**

Number of treatments:

13

Number of studies:

22

Number of individuals included:

1501

Number of individuals contributing to this analysis:

1313

Number of individuals randomized to each treatment:

Treatment name N. individuals randomized
1 agomelatine 124
2 amitriptyline 13
3 citalopram 15
4 desipramine 28
5 dothiepin 25
6 escitalopram 224
7 fluoxetine 174
8 nomifensine 13
9 nortriptyline 18
10 paroxetine 145
11 placebo 573
12 sertraline 115
13 venlafaxine 34

**Pairwise meta-analysis**
Standardized mean differences below 0 favor the first treatment of the comparison.

**Network map**

The thickness of lines is proportional to the number of studies comparing the two treatments and the size of circles is proportional to the number of individuals for each treatment.

**Netleague table**

Standardized mean differences (SMDs) and 95% confidence intervals (CIs) are reported. Results of the network meta-analysis are reported in the lower left part of the table and results from the pairwise meta-analysis are reported in the upper right part of the table. SMDs lower than 1 favour the column-defining treatment.

**Forest plot**

Placebo was used as a common comparator. SMDs below 0 favor the treatment over the common comparator.

**Assessment of heterogeneity and consistency**

*Global heterogeneity*

We interpreted tau^2 as follows: heterogeneity low with tau^2≤0.010, moderate with 0.010<tau^2≤0.242, high with tau^2>0.242, and I^2 statistics as follows: not important (0%-40%), moderate (30%-60%), substantial (50%-90%), considerable (75%-100%).

tau^2= 0.5435; tau= 0.7372

I^2= 85.65 % (77.86 % to 90.7 %)

Judgment: high heterogeneity

*Consistency: global approach*

Q statistic to assess consistency under the assumption of a full design-by-treatment interaction random effects model

Q df p-value tau.within tau2.within
Between designs 4.22 6 0.6475 0.7976 0.6361

*Consistency: local approach*

Separate indirect from direct evidence (SIDE) using back-calculation method

Random effects model:

 comparison k prop nma direct indir. Diff z p-value
 agomelatine:fluoxetine 1 0.44 -0.24 -0.47 -0.07 -0.40 -0.38 0.7005
 agomelatine:paroxetine 1 0.46 -0.09 -0.04 -0.13 0.09 0.08 0.9323
 agomelatine:sertraline 1 0.48 -0.62 -0.43 -0.79 0.36 0.32 0.7461
 citalopram:desipramine 1 0.87 -0.06 0.00 -0.44 0.44 0.19 0.8487
 placebo:citalopram 1 0.86 0.39 0.45 0.02 0.43 0.19 0.8487
 desipramine:paroxetine 1 0.52 0.37 -0.09 0.87 -0.95 -0.78 0.4338
 placebo:desipramine 2 0.89 0.34 0.07 2.42 -2.35 -1.32 0.1863
 fluoxetine:paroxetine 2 0.43 0.15 -0.00 0.27 -0.27 -0.34 0.7362
 placebo:fluoxetine 4 0.71 0.55 0.55 0.57 -0.02 -0.03 0.9741
 placebo:paroxetine 3 0.61 0.71 0.76 0.62 0.14 0.19 0.8516
 paroxetine:venlafaxine 1 0.81 -0.03 -0.67 2.64 -3.30 -1.88 0.0606
 placebo:sertraline 3 0.83 0.18 0.12 0.48 -0.36 -0.32 0.7461
 placebo:venlafaxine 1 0.80 0.68 1.33 -1.93 3.26 1.88 0.0606

Legend:
 comparison - Treatment comparison
 k - Number of studies providing direct evidence
 prop - Direct evidence proportion
 nma - Estimated treatment effect (SMD) in network meta-analysis
 direct - Estimated treatment effect (SMD) derived from direct evidence
 indir. - Estimated treatment effect (SMD) derived from indirect evidence
 Diff - Difference between direct and indirect treatment estimates
 z - z-value of test for disagreement (direct versus indirect)
 p-value - p-value of test for disagreement (direct versus indirect)

**Netrank**

Higher p-score values indicate higher ranking of treatments

P-score
agomelatine 0.7260
escitalopram 0.7128
paroxetine 0.6977
venlafaxine 0.6463
nortriptyline 0.6462
fluoxetine 0.6114
citalopram 0.5236
desipramine 0.4939
sertraline 0.4065
dothiepin 0.3272
placebo 0.2878
amitriptyline 0.2611
nomifensine 0.1598

**L. Secondary outcome: Mean Change in quality of life scales**

**Characteristics of the network**

Number of treatments:

8

Number of studies:

14

Number of individuals included:

811

Number of individuals contributing to this analysis:

707

Number of individuals randomized to each treatment:

Treatment name N. individuals randomized
1 amitriptyline 122
2 citalopram 41
3 dothiepin 25
4 escitalopram 8
5 fluoxetine 136
6 paroxetine 127
7 placebo 269
8 sertraline 83

**Pairwise meta-analysis**
Standardized mean differences below 0 favor the first treatment of the comparison.

**Network map**

The thickness of lines is proportional to the number of studies comparing the two treatments and the size of circles is proportional to the number of individuals for each treatment.

**Netleague table**

Standardized mean differences (SMDs) and 95% confidence intervals (CIs) are reported. Results of the network meta-analysis are reported in the lower left part of the table and results from the pairwise meta-analysis are reported in the upper right part of the table. SMDs higher than 1 favour the column-defining treatment.

**Forest plot**

Placebo was used as a common comparator. SMDs above 0 favor the treatment over the common comparator.

**Assessment of heterogeneity and consistency**

*Global heterogeneity*

We interpreted tau^2 as follows: heterogeneity low with tau^2≤0.010, moderate with 0.010<tau^2≤0.242, high with tau^2>0.242, and I^2 statistics as follows: not important (0%-40%), moderate (30%-60%), substantial (50%-90%), considerable (75%-100%).

tau^2=0.1906; tau= 0.4366

I^2= 66.45 % (28.97 % to 84.15 %)

Judgment: moderate heterogeneity

*Consistency: global approach*

Q statistic to assess consistency under the assumption of a full design-by-treatment interaction random effects model

Q df p-value tau.within tau2.within
Between designs 2.60 3 0.4570 0.4857 0.2359

*Consistency: local approach*

Separate indirect from direct evidence (SIDE) using back-calculation method

Random effects model:

 comparison k prop nma direct indir. Diff z p-value
 amitriptyline:paroxetine 1 0.59 -0.16 -0.34 0.10 -0.43 -0.60 0.5496
 placebo:amitriptyline 1 0.40 -0.48 -0.09 -0.74 0.65 0.92 0.3556
 amitriptyline:sertraline 1 0.41 0.07 0.88 -0.49 1.37 1.71 0.0877
 fluoxetine:paroxetine 1 0.38 -0.15 -0.05 -0.21 0.16 0.20 0.8392
 placebo:fluoxetine 2 0.81 -0.49 -0.46 -0.62 0.16 0.20 0.8392
 placebo:paroxetine 2 0.57 -0.64 -0.54 -0.77 0.22 0.35 0.7231
 placebo:sertraline 3 0.84 -0.42 -0.63 0.74 -1.37 -1.71 0.0877

Legend:
 comparison - Treatment comparison
 k - Number of studies providing direct evidence
 prop - Direct evidence proportion
 nma - Estimated treatment effect (SMD) in network meta-analysis
 direct - Estimated treatment effect (SMD) derived from direct evidence
 indir. - Estimated treatment effect (SMD) derived from indirect evidence
 Diff - Difference between direct and indirect treatment estimates
 z - z-value of test for disagreement (direct versus indirect)
 p-value - p-value of test for disagreement (direct versus indirect)

**Netrank**

Higher p-score values indicate higher ranking of treatments

P-score
paroxetine 0.7842
fluoxetine 0.6676
amitriptyline 0.6525
sertraline 0.6095
escitalopram 0.4055
dothiepin 0.3643
citalopram 0.2709
placebo 0.2454

**M. Secondary outcome: Mean change in functioning scales**

**Characteristics of the network**

Number of treatments:

10

Number of studies:

24

Number of individuals included:

2167

Number of individuals contributing to this analysis:

1966

Number of individuals randomized to each treatment:

Treatment name N. individuals randomized
1 amitriptyline 35
2 citalopram 233
3 desipramine 32
4 escitalopram 396
5 fluoxetine 66
6 paroxetine 78
7 placebo 1002
8 sertraline 163
9 trazodone 39
10 venlafaxine 123

**Pairwise meta-analysis**
Standardized mean differences below 0 favor the first treatment of the comparison.

**Network map**

The thickness of lines is proportional to the number of studies comparing the two treatments and the size of circles is proportional to the number of individuals for each treatment.

**Netleague table**

Standardized mean differences (SMDs) and 95% confidence intervals (CIs) are reported. Results of the network meta-analysis are reported in the lower left part of the table and results from the pairwise meta-analysis are reported in the upper right part of the table. SMDs lower than 1 favour the column-defining treatment.

**Forest plot**

Placebo was used as a common comparator. SMDs below 0 favor the treatment over the common comparator.

**Assessment of heterogeneity and consistency**

*Global heterogeneity*

We interpreted tau^2 as follows: heterogeneity low with tau^2≤0.010, moderate with 0.010<tau^2≤0.242, high with tau^2>0.242, and I^2 statistics as follows: not important (0%-40%), moderate (30%-60%), substantial (50%-90%), considerable (75%-100%).

tau^2= 0.5038; tau= 0.7098

I^2= 89.1 % (84.47 % to 92.35 %)

Judgment: high heterogenity

*Consistency: global approach*

Q statistic to assess consistency under the assumption of a full design-by-treatment interaction random effects model

Q df p-value tau.within tau2.within
Between designs 8.72 6 0.1901 0.6744 0.4548

*Consistency: local approach*

Separate indirect from direct evidence (SIDE) using back-calculation method

Random effects model:

 comparison k prop nma direct indir. Diff z p-value
 placebo:amitriptyline 1 0.56 0.35 1.17 -0.70 1.87 1.56 0.1184
 amitriptyline:sertraline 1 0.52 -0.35 0.54 -1.33 1.87 1.56 0.1184
 desipramine:fluoxetine 1 0.46 -0.83 -1.65 -0.13 -1.53 -1.22 0.2221
 desipramine:sertraline 1 0.56 -0.50 -0.02 -1.11 1.09 0.93 0.3523
 desipramine:trazodone 1 0.53 -1.63 -1.40 -1.90 0.50 0.38 0.7016
 desipramine:venlafaxine 1 0.60 0.06 -0.02 0.18 -0.19 -0.16 0.8752
 placebo:fluoxetine 3 0.81 -0.33 -0.04 -1.59 1.55 1.49 0.1371
 fluoxetine:trazodone 1 0.39 -0.81 0.25 -1.49 1.75 1.46 0.1449
 placebo:paroxetine 3 0.93 0.29 0.38 -0.87 1.24 0.73 0.4673
 paroxetine:venlafaxine 1 0.52 0.27 0.18 0.36 -0.18 -0.17 0.8642
 placebo:sertraline 4 0.75 0.00 -0.36 1.08 -1.44 -1.88 0.0602
 placebo:trazodone 2 0.68 -1.13 -1.49 -0.35 -1.15 -1.06 0.2880
 placebo:venlafaxine 2 0.65 0.56 0.92 -0.09 1.01 1.14 0.2539
 sertraline:venlafaxine 1 0.39 0.56 0.00 0.91 -0.91 -0.92 0.3602

Legend:
 comparison - Treatment comparison
 k - Number of studies providing direct evidence
 prop - Direct evidence proportion
 nma - Estimated treatment effect (SMD) in network meta-analysis
 direct - Estimated treatment effect (SMD) derived from direct evidence
 indir. - Estimated treatment effect (SMD) derived from indirect evidence
 Diff - Difference between direct and indirect treatment estimates
 z - z-value of test for disagreement (direct versus indirect)
 p-value - p-value of test for disagreement (direct versus indirect)

**Netrank**

Higher p-score values indicate higher ranking of treatments

P-score
trazodone 0.9739
fluoxetine 0.7444
placebo 0.5747
sertraline 0.5634
citalopram 0.4884
escitalopram 0.4520
paroxetine 0.3693
amitriptyline 0.3532
desipramine 0.2689
venlafaxine 0.2119

**N. Secondary outcome: All-cause discontinuation**

**Primary outcome: tolerability**

**Characteristics of the network**

Number of treatments:

24

Number of studies:

102

Number of individuals included:

7798

Number of individuals randomized to each treatment:

Treatment name N. individuals randomized
1 agomelatine 145
2 amitriptyline 313
3 citalopram 436
4 clomipramine 12
5 desipramine 83
6 dothiepin 25
7 doxepin 30
8 duloxetine 21
9 escitalopram 610
10 fluoxetine 533
11 imipramine 89
12 lofepramine 32
13 mianserin 101
14 mirtazapine 109
15 nefazodone 9
16 nomifensine 13
17 nortriptyline 139
18 paroxetine 570
19 placebo 3082
20 sertraline 1240
21 trazodone 28
22 trimipramine 18
23 venlafaxine 137
24 vortioxetine 23

**Pairwise meta-analysis**
Standardized mean differences below 0 favor the first treatment of the comparison.

**Network map**

The thickness of lines is proportional to the number of studies comparing the two treatments and the size of circles is proportional to the number of individuals for each treatment.

**Netleague table**

Standardized mean differences (SMDs) and 95% confidence intervals (CIs) are reported. Results of the network meta-analysis are reported in the lower left part of the table and results from the pairwise meta-analysis are reported in the upper right part of the table. SMDs lower than 1 favour the column-defining treatment.

**Forest plot**

Placebo was used as a common comparator. SMDs below 0 favor the treatment over the common comparator.

**Assessment of heterogeneity and consistency**

*Global heterogeneity*

We interpreted tau^2 as follows: heterogeneity low with tau^2≤0.010, moderate with 0.010<tau^2≤0.242, high with tau^2>0.242, and I^2 statistics as follows: not important (0%-40%), moderate (30%-60%), substantial (50%-90%), considerable (75%-100%).

tau^2= 0 ; tau= 0

I^2= 0 % ( 0 % to 25.97 %)

Judgment: no heterogeneity

*Consistency: global approach*

Q statistic to assess consistency under the assumption of a full design-by-treatment interaction random effects model

Q df p-value tau.within tau2.within
Between designs 14.14 27 0.9799 0 0

*Consistency: local approach*

Separate indirect from direct evidence (SIDE) using back-calculation method

Random effects model:

 comparison k prop nma direct indir. RoR z p-value
 agomelatine:escitalopram 1 0.26 1.00 1.00 1.00 1.00 -0.00 0.9984
 agomelatine:fluoxetine 1 0.25 0.98 0.91 1.01 0.90 -0.07 0.9474
 agomelatine:paroxetine 1 0.25 0.91 0.93 0.91 1.03 0.02 0.9849
 agomelatine:sertraline 1 0.26 0.94 1.00 0.93 1.08 0.05 0.9612
 amitriptyline:citalopram 1 0.03 1.67 1.00 1.70 0.59 -0.37 0.7096
 amitriptyline:fluoxetine 1 0.31 1.26 1.59 1.13 1.40 0.67 0.5035
 amitriptyline:nomifensine 1 0.76 1.17 1.00 1.90 0.53 -0.23 0.8153
 amitriptyline:paroxetine 2 0.75 1.17 1.12 1.30 0.87 -0.34 0.7331
 placebo:amitriptyline 2 0.06 0.73 0.69 0.73 0.95 -0.06 0.9524
 amitriptyline:sertraline 1 0.13 1.21 1.07 1.23 0.87 -0.22 0.8294
 citalopram:desipramine 1 0.07 0.63 2.27 0.57 3.98 1.14 0.2553
 citalopram:mirtazapine 1 0.62 0.89 0.65 1.49 0.44 -2.38 0.0174
 placebo:citalopram 7 0.86 1.22 1.11 2.18 0.51 -2.03 0.0426
 desipramine:fluoxetine 3 0.54 1.20 1.57 0.88 1.79 1.02 0.3072
 desipramine:paroxetine 1 0.34 1.12 0.95 1.21 0.78 -0.38 0.7024
 placebo:desipramine 2 0.35 0.76 1.22 0.59 2.06 1.21 0.2274
 desipramine:sertraline 1 0.05 1.15 1.05 1.16 0.91 -0.07 0.9454
 desipramine:trazodone 1 0.26 0.80 1.85 0.59 3.13 0.98 0.3291
 desipramine:venlafaxine 1 0.11 1.31 1.05 1.34 0.78 -0.17 0.8675
 escitalopram:mirtazapine 1 0.07 1.17 1.67 1.14 1.47 0.54 0.5915
 placebo:escitalopram 13 0.99 0.94 0.94 0.68 1.38 0.49 0.6232
 fluoxetine:paroxetine 2 0.04 0.93 0.90 0.93 0.97 -0.03 0.9770
 placebo:fluoxetine 12 0.80 0.92 0.85 1.25 0.68 -1.06 0.2899
 fluoxetine:sertraline 1 0.01 0.96 1.15 0.96 1.21 0.14 0.8911
 fluoxetine:trazodone 1 0.14 0.66 1.20 0.60 2.00 0.50 0.6151
 imipramine:paroxetine 1 0.76 1.16 1.07 1.51 0.71 -0.70 0.4843
 placebo:imipramine 3 0.82 0.73 0.71 0.85 0.83 -0.34 0.7366
 placebo:mirtazapine 1 0.45 1.09 1.64 0.78 2.09 2.13 0.0332
 placebo:nomifensine 1 0.76 0.85 1.00 0.53 1.90 0.23 0.8153
 nortriptyline:paroxetine 3 0.67 2.41 3.19 1.36 2.34 1.36 0.1732
 placebo:nortriptyline 4 0.43 0.35 0.60 0.23 2.58 1.54 0.1229
 placebo:paroxetine 8 0.67 0.85 0.89 0.79 1.12 0.34 0.7335
 paroxetine:venlafaxine 1 0.44 1.17 1.62 0.91 1.78 0.76 0.4478
 placebo:sertraline 20 0.98 0.88 0.88 0.84 1.05 0.10 0.9233
 placebo:trazodone 1 0.80 0.61 0.49 1.42 0.34 -0.93 0.3506
 placebo:venlafaxine 3 0.82 1.00 0.97 1.16 0.83 -0.20 0.8447
 sertraline:venlafaxine 1 0.07 1.13 1.00 1.14 0.87 -0.09 0.9249

Legend:
 comparison - Treatment comparison
 k - Number of studies providing direct evidence
 prop - Direct evidence proportion
 nma - Estimated treatment effect (RR) in network meta-analysis
 direct - Estimated treatment effect (RR) derived from direct evidence
 indir. - Estimated treatment effect (RR) derived from indirect evidence
 RoR - Ratio of Ratios (direct versus indirect)
 z - z-value of test for disagreement (direct versus indirect)
 p-value - p-value of test for disagreement (direct versus indirect)

**Netrank**

Higher p-score values indicate higher ranking of treatments

P-score
mianserin 0.9292
dothiepin 0.8158
doxepin 0.8149
citalopram 0.7340
clomipramine 0.7246
vortioxetine 0.6562
mirtazapine 0.6308
placebo 0.5786
venlafaxine 0.5383
duloxetine 0.5070
agomelatine 0.4929
escitalopram 0.4867
fluoxetine 0.4757
lofepramine 0.4711
nomifensine 0.4674
sertraline 0.4167
trimipramine 0.4120
paroxetine 0.4053
desipramine 0.3386
imipramine 0.2880
amitriptyline 0.2857
trazodone 0.2523
nefazodone 0.2226
nortriptyline 0.0555

**O. Secondary outcome: Discontinuations due to inefficacy**

**Characteristics of the network**

Number of treatments:

20

Number of studies:

55

Number of individuals included:

4542

Number of individuals randomized to each treatment:

Treatment name N. individuals randomized
1 agomelatine 124
2 amitriptyline 199
3 citalopram 314
4 clomipramine 12
5 desipramine 53
6 escitalopram 156
7 fluoxetine 261
8 imipramine 39
9 mianserin 101
10 moclobemide 368
11 nefazodone 9
12 nomifensine 13
13 nortriptyline 75
14 paroxetine 372
15 placebo 1747
16 sertraline 568
17 trazodone 22
18 trimipramine 18
19 venlafaxine 68
20 vortioxetine 23

**Pairwise meta-analysis**
Standardized mean differences below 0 favor the first treatment of the comparison.

**Network map**

The thickness of lines is proportional to the number of studies comparing the two treatments and the size of circles is proportional to the number of individuals for each treatment.

**Netleague table**

Standardized mean differences (SMDs) and 95% confidence intervals (CIs) are reported. Results of the network meta-analysis are reported in the lower left part of the table and results from the pairwise meta-analysis are reported in the upper right part of the table. SMDs lower than 1 favour the column-defining treatment.

**Forest plot** Placebo was used as a common comparator. SMDs below 0 favor the treatment over the common comparator.

**Assessment of heterogeneity and consistency**

*Global heterogeneity*

We interpreted tau^2 as follows: heterogeneity low with tau^2≤0.010, moderate with 0.010<tau^2≤0.242, high with tau^2>0.242, and I^2 statistics as follows: not important (0%-40%), moderate (30%-60%), substantial (50%-90%), considerable (75%-100%).

tau^2= 0 ; tau= 0

I^2= 0 % ( 0 % to 35.13 %)

Judgment: no heterogenity

*Consistency: global approach*

Q statistic to assess consistency under the assumption of a full design-by-treatment interaction random effects model

Q df p-value tau.within tau2.within
Between designs 2.88 19 1.0000 0 0

*Consistency: local approach*

Separate indirect from direct evidence (SIDE) using back-calculation method

Random effects model:

 comparison k prop nma direct indir. RoR z p-value
 agomelatine:fluoxetine 1 0.37 0.90 0.91 0.89 1.02 0.01 0.9899
 agomelatine:paroxetine 1 0.37 1.01 0.93 1.06 0.88 -0.07 0.9407
 agomelatine:sertraline 1 0.37 0.94 1.00 0.90 1.11 0.06 0.9514
 amitriptyline:citalopram 1 0.24 1.67 1.00 1.97 0.51 -0.42 0.6742
 amitriptyline:fluoxetine 1 0.31 0.71 0.46 0.87 0.53 -0.43 0.6655
 amitriptyline:nomifensine 1 0.80 0.79 1.00 0.31 3.24 0.38 0.7003
 amitriptyline:paroxetine 1 0.23 0.81 1.01 0.75 1.34 0.18 0.8542
 placebo:amitriptyline 1 0.21 1.59 1.00 1.80 0.56 -0.38 0.7003
 amitriptyline:sertraline 1 0.22 0.74 1.07 0.67 1.59 0.30 0.7650
 placebo:citalopram 4 0.94 2.65 2.77 1.41 1.97 0.42 0.6742
 desipramine:fluoxetine 2 0.43 1.25 1.29 1.22 1.05 0.04 0.9665
 desipramine:paroxetine 1 0.46 1.41 1.18 1.64 0.72 -0.27 0.7910
 placebo:desipramine 1 0.27 0.91 0.50 1.13 0.44 -0.61 0.5424
 desipramine:sertraline 1 0.20 1.30 1.05 1.37 0.77 -0.17 0.8627
 desipramine:venlafaxine 1 0.38 1.24 1.05 1.37 0.77 -0.15 0.8803
 fluoxetine:paroxetine 1 0.13 1.13 0.82 1.19 0.69 -0.26 0.7953
 placebo:fluoxetine 6 0.62 1.13 1.17 1.07 1.09 0.11 0.9149
 fluoxetine:sertraline 1 0.10 1.04 1.15 1.03 1.12 0.08 0.9379
 imipramine:paroxetine 1 0.60 0.44 0.33 0.69 0.48 -0.41 0.6825
 placebo:imipramine 2 0.98 2.89 3.10 0.03 97.51 0.69 0.4923
 placebo:nomifensine 1 0.80 1.26 1.00 3.24 0.31 -0.38 0.7003
 nortriptyline:paroxetine 2 0.63 1.16 1.04 1.38 0.75 -0.17 0.8615
 placebo:nortriptyline 2 0.64 1.11 1.00 1.34 0.75 -0.18 0.8571
 placebo:paroxetine 7 0.77 1.28 1.36 1.06 1.29 0.29 0.7705
 paroxetine:venlafaxine 1 0.30 0.88 0.81 0.91 0.89 -0.07 0.9448
 placebo:sertraline 9 0.90 1.18 1.17 1.32 0.88 -0.15 0.8784
 placebo:venlafaxine 2 0.63 1.13 1.26 0.93 1.35 0.20 0.8413
 sertraline:venlafaxine 1 0.29 0.95 1.00 0.93 1.07 0.04 0.9659

Legend:
 comparison - Treatment comparison
 k - Number of studies providing direct evidence
 prop - Direct evidence proportion
 nma - Estimated treatment effect (RR) in network meta-analysis
 direct - Estimated treatment effect (RR) derived from direct evidence
 indir. - Estimated treatment effect (RR) derived from indirect evidence
 RoR - Ratio of Ratios (direct versus indirect)
 z - z-value of test for disagreement (direct versus indirect)
 p-value - p-value of test for disagreement (direct versus indirect)

**Netrank**

Higher p-score values indicate higher ranking of treatments

P-score
mianserin 0.8818
citalopram 0.7917
imipramine 0.7558
amitriptyline 0.5780
nefazodone 0.5227
paroxetine 0.4936
moclobemide 0.4907
nomifensine 0.4833
agomelatine 0.4824
sertraline 0.4512
trazodone 0.4499
venlafaxine 0.4367
nortriptyline 0.4329
vortioxetine 0.4287
fluoxetine 0.4279
clomipramine 0.4247
trimipramine 0.4053
escitalopram 0.3859
desipramine 0.3404
placebo 0.3365

**P. Secondary outcome: Deaths due to medical condition**

**Characteristics of the network**

Number of treatments:

22

Number of studies:

82

Number of individuals included:

5851

Number of individuals randomized to each treatment:

Treatment name N. individuals randomized
1 agomelatine 145
2 amitriptyline 144
3 citalopram 456
4 clomipramine 12
5 desipramine 107
6 dothiepin 25
7 doxepin 18
8 duloxetine 21
9 escitalopram 490
10 fluoxetine 380
11 imipramine 14
12 mianserin 28
13 mirtazapine 109
14 nefazodone 9
15 nomifensine 13
16 nortriptyline 139
17 paroxetine 425
18 placebo 2271
19 sertraline 862
20 trazodone 28
21 trimipramine 18
22 venlafaxine 137

**Pairwise meta-analysis**
Standardized mean differences below 0 favor the first treatment of the comparison.

**Network map** The thickness of lines is proportional to the number of studies comparing the two treatments and the size of circles is proportional to the number of individuals for each treatment.

**Netleague table**

Standardized mean differences (SMDs) and 95% confidence intervals (CIs) are reported. Results of the network meta-analysis are reported in the lower left part of the table and results from the pairwise meta-analysis are reported in the upper right part of the table. SMDs lower than 1 favour the column-defining treatment.

**Forest plot** Placebo was used as a common comparator. SMDs below 0 favor the treatment over the common comparator.

**Assessment of heterogeneity and consistency**

*Global heterogeneity*

We interpreted tau^2 as follows: heterogeneity low with tau^2≤0.010, moderate with 0.010<tau^2≤0.242, high with tau^2>0.242, and I^2 statistics as follows: not important (0%-40%), moderate (30%-60%), substantial (50%-90%), considerable (75%-100%).

tau^2= 0 ; tau= 0

I^2= 0 % (0 % to 28.72 %)

Judgment: no heterogeneity

*Consistency: global approach*

Q statistic to assess consistency under the assumption of a full design-by-treatment interaction random effects model

Q df p-value tau.within tau2.within
Between designs 0.94 24 1.0000 0 0

*Consistency: local approach*

Separate indirect from direct evidence (SIDE) using back-calculation method

Random effects model:

 comparison k prop nma direct indir. RoR z p-value
 agomelatine:escitalopram 1 0.29 0.99 1.00 0.99 1.01 0.01 0.9945
 agomelatine:fluoxetine 1 0.29 1.01 0.91 1.06 0.86 -0.09 0.9271
 agomelatine:paroxetine 1 0.30 1.00 0.93 1.03 0.91 -0.06 0.9539
 agomelatine:sertraline 1 0.29 0.85 1.00 0.80 1.26 0.14 0.8890
 amitriptyline:citalopram 1 0.38 1.01 1.00 1.02 0.98 -0.01 0.9913
 amitriptyline:nomifensine 1 0.84 1.00 1.00 1.01 0.99 -0.00 0.9986
 amitriptyline:paroxetine 1 0.37 1.00 1.01 0.99 1.02 0.01 0.9898
 placebo:amitriptyline 1 0.37 1.00 1.00 1.00 1.00 0.00 0.9986
 citalopram:desipramine 1 0.21 1.10 1.13 1.09 1.04 0.02 0.9809
 citalopram:fluoxetine 1 0.16 1.00 1.00 1.00 1.00 0.00 0.9991
 citalopram:mirtazapine 1 0.39 0.95 0.82 1.04 0.79 -0.13 0.8943
 placebo:citalopram 7 0.74 1.01 0.99 1.07 0.93 -0.07 0.9420
 desipramine:fluoxetine 3 0.47 0.91 0.85 0.96 0.89 -0.12 0.9081
 desipramine:paroxetine 1 0.19 0.89 1.18 0.84 1.41 0.23 0.8190
 placebo:desipramine 3 0.43 1.11 1.18 1.06 1.11 0.11 0.9147
 desipramine:sertraline 1 0.15 0.76 1.05 0.72 1.47 0.26 0.7981
 desipramine:trazodone 1 0.50 0.82 0.46 1.47 0.31 -0.62 0.5362
 desipramine:venlafaxine 1 0.31 0.83 1.05 0.75 1.41 0.21 0.8368
 escitalopram:mirtazapine 1 0.37 0.97 1.00 0.95 1.06 0.03 0.9754
 placebo:escitalopram 10 0.93 0.99 0.99 0.97 1.02 0.02 0.9869
 fluoxetine:paroxetine 2 0.26 0.99 0.90 1.02 0.88 -0.11 0.9132
 placebo:fluoxetine 7 0.53 1.01 1.06 0.95 1.11 0.14 0.8876
 fluoxetine:sertraline 1 0.10 0.84 1.15 0.81 1.43 0.25 0.8053
 fluoxetine:trazodone 1 0.50 0.91 1.20 0.69 1.75 0.31 0.7578
 placebo:mirtazapine 1 0.35 0.96 1.07 0.90 1.19 0.10 0.9210
 placebo:nomifensine 1 0.84 1.00 1.00 0.99 1.01 0.00 0.9986
 nortriptyline:paroxetine 3 0.55 1.15 1.04 1.31 0.79 -0.20 0.8432
 placebo:nortriptyline 4 0.70 0.86 0.82 0.99 0.82 -0.15 0.8769
 placebo:paroxetine 7 0.60 1.00 1.05 0.92 1.13 0.15 0.8801
 paroxetine:venlafaxine 1 0.27 0.93 0.81 0.98 0.83 -0.12 0.9077
 placebo:sertraline 16 0.91 0.85 0.82 1.12 0.73 -0.35 0.7247
 placebo:trazodone 1 0.42 0.92 1.10 0.80 1.37 0.17 0.8623
 placebo:venlafaxine 3 0.66 0.92 0.92 0.94 0.97 -0.02 0.9839
 sertraline:venlafaxine 1 0.24 1.09 1.00 1.12 0.89 -0.07 0.9412

Legend:
 comparison - Treatment comparison
 k - Number of studies providing direct evidence
 prop - Direct evidence proportion
 nma - Estimated treatment effect (RR) in network meta-analysis
 direct - Estimated treatment effect (RR) derived from direct evidence
 indir. - Estimated treatment effect (RR) derived from indirect evidence
 RoR - Ratio of Ratios (direct versus indirect)
 z - z-value of test for disagreement (direct versus indirect)
 p-value - p-value of test for disagreement (direct versus indirect)

**Netrank**

Higher p-score values indicate higher ranking of treatments

P-score
nefazodone 0.5666
desipramine 0.5548
doxepin 0.5260
dothiepin 0.5212
duloxetine 0.5125
placebo 0.5107
fluoxetine 0.5097
citalopram 0.5094
mianserin 0.5093
paroxetine 0.5032
amitriptyline 0.5010
agomelatine 0.5009
clomipramine 0.5002
imipramine 0.5002
trimipramine 0.5001
nomifensine 0.5001
escitalopram 0.4999
mirtazapine 0.4851
trazodone 0.4712
venlafaxine 0.4705
nortriptyline 0.4386
sertraline 0.4090

**Q. Secondary outcome: Deaths due to any cause**

**Characteristics of the network**

Number of treatments:

23

Number of studies:

97

Number of individuals included:

7076

Number of individuals randomized to each treatment:

Treatment name N. individuals randomized
1 agomelatine 145
2 amitriptyline 159
3 citalopram 456
4 clomipramine 12
5 desipramine 107
6 dothiepin 25
7 doxepin 18
8 duloxetine 21
9 escitalopram 544
10 fluoxetine 516
11 imipramine 89
12 lofepramine 32
13 mianserin 101
14 mirtazapine 109
15 nefazodone 9
16 nomifensine 13
17 nortriptyline 139
18 paroxetine 450
19 placebo 2846
20 sertraline 1091
21 trazodone 39
22 trimipramine 18
23 venlafaxine 137

**Pairwise meta-analysis**
Standardized mean differences below 0 favor the first treatment of the comparison.

**Network map** The thickness of lines is proportional to the number of studies comparing the two treatments and the size of circles is proportional to the number of individuals for each treatment.

**Netleague table**

Standardized mean differences (SMDs) and 95% confidence intervals (CIs) are reported. Results of the network meta-analysis are reported in the lower left part of the table and results from the pairwise meta-analysis are reported in the upper right part of the table. SMDs lower than 1 favour the column-defining treatment.

**Forest plot**

Placebo was used as a common comparator. SMDs below 0 favor the treatment over the common comparator.

**Assessment of heterogeneity and consistency**

*Global heterogeneity*

We interpreted tau^2 as follows: heterogeneity low with tau^2≤0.010, moderate with 0.010<tau^2≤0.242, high with tau^2>0.242, and I^2 statistics as follows: not important (0%-40%), moderate (30%-60%), substantial (50%-90%), considerable (75%-100%).

tau^2= 0 ; tau= 0

I^2= 0 % (0 % to 26.37 %)

Judgment: no heterogeneity

*Consistency: global approach*

Q statistic to assess consistency under the assumption of a full design-by-treatment interaction random effects model

Q df p-value tau.within tau2.within
Between designs 0.84 27 1.0000 0 0

*Consistency: local approach*

Separate indirect from direct evidence (SIDE) using back-calculation method

Random effects model:

 comparison k prop nma direct indir. RoR z p-value
 agomelatine:escitalopram 1 0.28 0.87 1.00 0.83 1.21 0.12 0.9063
 agomelatine:fluoxetine 1 0.28 1.00 0.91 1.03 0.88 -0.08 0.9388
 agomelatine:paroxetine 1 0.29 0.98 0.93 1.00 0.93 -0.04 0.9663
 agomelatine:sertraline 1 0.28 1.00 1.00 1.00 1.00 -0.00 0.9999
 amitriptyline:citalopram 1 0.30 1.02 1.00 1.03 0.97 -0.02 0.9870
 amitriptyline:nomifensine 1 0.82 1.01 1.00 1.03 0.97 -0.01 0.9917
 amitriptyline:paroxetine 1 0.29 1.01 1.01 1.01 1.00 -0.00 0.9989
 placebo:amitriptyline 1 0.27 0.99 1.00 0.98 1.02 0.01 0.9917
 amitriptyline:sertraline 1 0.28 1.03 1.07 1.02 1.05 0.03 0.9779
 citalopram:desipramine 1 0.20 1.13 1.13 1.13 1.01 0.00 0.9972
 citalopram:fluoxetine 1 0.13 1.01 1.00 1.01 0.99 -0.01 0.9943
 citalopram:mirtazapine 1 0.38 0.91 0.82 0.98 0.84 -0.10 0.9189
 placebo:citalopram 7 0.75 1.01 0.99 1.06 0.94 -0.06 0.9497
 desipramine:fluoxetine 3 0.44 0.89 0.85 0.93 0.91 -0.09 0.9304
 desipramine:paroxetine 1 0.19 0.88 1.18 0.82 1.44 0.24 0.8098
 placebo:desipramine 3 0.41 1.14 1.18 1.11 1.07 0.07 0.9453
 desipramine:sertraline 1 0.15 0.90 1.05 0.87 1.20 0.12 0.9006
 desipramine:trazodone 1 0.39 0.83 0.46 1.21 0.38 -0.57 0.5694
 desipramine:venlafaxine 1 0.31 0.84 1.05 0.76 1.39 0.20 0.8435
 escitalopram:mirtazapine 1 0.36 1.04 1.00 1.06 0.95 -0.03 0.9749
 placebo:escitalopram 11 0.95 0.89 0.88 1.01 0.88 -0.11 0.9134
 fluoxetine:paroxetine 2 0.21 0.99 0.90 1.01 0.89 -0.10 0.9176
 placebo:fluoxetine 12 0.70 1.02 1.02 1.00 1.02 0.04 0.9702
 fluoxetine:sertraline 1 0.07 1.00 1.15 0.99 1.16 0.11 0.9157
 fluoxetine:trazodone 1 0.36 0.93 1.20 0.81 1.49 0.25 0.8041
 imipramine:paroxetine 1 0.35 0.98 1.00 0.97 1.03 0.02 0.9877
 placebo:imipramine 3 0.91 1.02 1.02 1.00 1.02 0.01 0.9941
 placebo:mirtazapine 1 0.35 0.92 1.07 0.85 1.25 0.13 0.8958
 placebo:nomifensine 1 0.82 0.99 1.00 0.97 1.03 0.01 0.9917
 nortriptyline:paroxetine 3 0.54 1.16 1.04 1.32 0.79 -0.20 0.8399
 placebo:nortriptyline 4 0.69 0.87 0.82 0.99 0.82 -0.16 0.8732
 placebo:paroxetine 8 0.61 1.00 1.04 0.94 1.10 0.13 0.9002
 paroxetine:venlafaxine 1 0.27 0.95 0.81 1.01 0.80 -0.14 0.8914
 placebo:sertraline 19 0.89 1.02 1.01 1.09 0.93 -0.10 0.9231
 placebo:trazodone 2 0.59 0.95 1.05 0.82 1.28 0.17 0.8685
 placebo:venlafaxine 3 0.66 0.95 0.92 1.03 0.89 -0.09 0.9316
 sertraline:venlafaxine 1 0.24 0.93 1.00 0.91 1.09 0.06 0.9551

Legend:
 comparison - Treatment comparison
 k - Number of studies providing direct evidence
 prop - Direct evidence proportion
 nma - Estimated treatment effect (RR) in network meta-analysis
 direct - Estimated treatment effect (RR) derived from direct evidence
 indir. - Estimated treatment effect (RR) derived from indirect evidence
 RoR - Ratio of Ratios (direct versus indirect)
 z - z-value of test for disagreement (direct versus indirect)
 p-value - p-value of test for disagreement (direct versus indirect)

**Netrank**

Higher p-score values indicate higher ranking of treatments

P-score
nefazodone 0.5672
desipramine 0.5615
doxepin 0.5292
dothiepin 0.5189
sertraline 0.5127
duloxetine 0.5101
fluoxetine 0.5087
agomelatine 0.5062
lofepramine 0.5057
imipramine 0.5050
mianserin 0.5036
citalopram 0.5025
placebo 0.5012
paroxetine 0.5000
trimipramine 0.4976
clomipramine 0.4976
nomifensine 0.4959
amitriptyline 0.4931
venlafaxine 0.4780
trazodone 0.4761
mirtazapine 0.4677
nortriptyline 0.4330
escitalopram 0.4283

**R. Secondary outcome: Serious adverse events**

**Characteristics of the network**

Number of treatments:

20

Number of studies:

80

Number of individuals included:

6119

Number of individuals randomized to each treatment:

Treatment name N. individuals randomized
1 agomelatine 124
2 amitriptyline 253
3 citalopram 306
4 clomipramine 12
5 desipramine 76
6 dothiepin 25
7 duloxetine 21
8 escitalopram 508
9 fluoxetine 404
10 imipramine 14
11 mirtazapine 109
12 nefazodone 9
13 nomifensine 13
14 nortriptyline 132
15 paroxetine 483
16 placebo 2351
17 sertraline 1091
18 trazodone 33
19 trimipramine 18
20 venlafaxine 137

**Pairwise meta-analysis**
Standardized mean differences below 0 favor the first treatment of the comparison.

**Network map**

The thickness of lines is proportional to the number of studies comparing the two treatments and the size of circles is proportional to the number of individuals for each treatment.

**Netleague table**

Standardized mean differences (SMDs) and 95% confidence intervals (CIs) are reported. Results of the network meta-analysis are reported in the lower left part of the table and results from the pairwise meta-analysis are reported in the upper right part of the table. SMDs lower than 1 favour the column-defining treatment.

**Forest plot** Placebo was used as a common comparator. SMDs below 0 favor the treatment over the common comparator.

**Assessment of heterogeneity and consistency**

*Global heterogeneity*

We interpreted tau^2 as follows: heterogeneity low with tau^2≤0.010, moderate with 0.010<tau^2≤0.242, high with tau^2>0.242, and I^2 statistics as follows: not important (0%-40%), moderate (30%-60%), substantial (50%-90%), considerable (75%-100%).

tau^2= 0; tau= 0

I^2= 0 % (0 % to 29.47 %)

Judgment: no heterogeneity

*Consistency: global approach*

Q statistic to assess consistency under the assumption of a full design-by-treatment interaction random effects model

Q df p-value tau.within tau2.within
Between designs 4.21 20 0.9999 0 0

*Consistency: local approach*

Separate indirect from direct evidence (SIDE) using back-calculation method

Random effects model:

 comparison k prop nma direct indir. RoR z p-value
 agomelatine:fluoxetine 1 0.44 0.71 0.45 1.01 0.45 -0.50 0.6198
 agomelatine:paroxetine 1 0.34 0.89 0.93 0.87 1.07 0.04 0.9690
 agomelatine:sertraline 1 0.33 0.58 1.00 0.45 2.25 0.48 0.6301
 amitriptyline:citalopram 1 0.20 1.13 1.00 1.16 0.86 -0.10 0.9239
 amitriptyline:nomifensine 1 0.80 1.15 1.00 1.98 0.50 -0.23 0.8209
 amitriptyline:paroxetine 2 0.49 2.12 1.95 2.30 0.85 -0.13 0.8937
 placebo:amitriptyline 1 0.19 0.76 1.00 0.71 1.41 0.23 0.8209
 amitriptyline:sertraline 1 0.27 1.38 2.13 1.18 1.81 0.43 0.6637
 citalopram:desipramine 1 0.24 1.22 2.27 1.00 2.28 0.61 0.5418
 citalopram:fluoxetine 1 0.09 1.50 1.00 1.56 0.64 -0.31 0.7572
 citalopram:mirtazapine 1 0.30 0.69 0.82 0.64 1.28 0.15 0.8803
 placebo:citalopram 4 0.91 0.85 0.86 0.76 1.13 0.15 0.8818
 desipramine:fluoxetine 2 0.38 1.23 1.29 1.20 1.07 0.06 0.9557
 placebo:desipramine 2 0.44 1.04 0.85 1.22 0.70 -0.32 0.7476
 desipramine:sertraline 1 0.16 1.01 1.05 1.00 1.06 0.04 0.9711
 desipramine:venlafaxine 1 0.33 1.10 1.05 1.12 0.94 -0.04 0.9692
 escitalopram:mirtazapine 1 0.29 0.56 1.00 0.44 2.25 0.49 0.6229
 placebo:escitalopram 10 1.00 1.05 1.05 0.47 2.25 0.49 0.6229
 fluoxetine:paroxetine 1 0.14 1.25 0.82 1.35 0.61 -0.34 0.7305
 placebo:fluoxetine 9 0.67 1.28 1.39 1.09 1.27 0.32 0.7489
 fluoxetine:sertraline 1 0.07 0.82 1.15 0.79 1.45 0.26 0.7912
 placebo:mirtazapine 1 0.43 0.59 0.36 0.86 0.41 -0.59 0.5584
 placebo:nomifensine 1 0.80 0.87 1.00 0.50 1.98 0.23 0.8209
 nortriptyline:paroxetine 3 0.77 2.67 3.45 1.14 3.04 0.94 0.3449
 placebo:nortriptyline 3 0.52 0.60 1.23 0.28 4.41 1.37 0.1706
 placebo:paroxetine 6 0.59 1.61 1.36 2.05 0.66 -0.50 0.6172
 paroxetine:venlafaxine 1 0.28 0.71 0.81 0.68 1.20 0.11 0.9126
 placebo:sertraline 17 0.98 1.05 1.04 1.62 0.64 -0.61 0.5395
 placebo:venlafaxine 3 0.70 1.14 1.20 1.02 1.18 0.12 0.9055
 sertraline:venlafaxine 1 0.22 1.09 1.00 1.12 0.89 -0.07 0.9416

Legend:
 comparison - Treatment comparison
 k - Number of studies providing direct evidence
 prop - Direct evidence proportion
 nma - Estimated treatment effect (RR) in network meta-analysis
 direct - Estimated treatment effect (RR) derived from direct evidence
 indir. - Estimated treatment effect (RR) derived from indirect evidence
 RoR - Ratio of Ratios (direct versus indirect)
 z - z-value of test for disagreement (direct versus indirect)
 p-value - p-value of test for disagreement (direct versus indirect)

**Netrank**

Higher p-score values indicate higher ranking of treatments

P-score
paroxetine 0.7227
agomelatine 0.7024
fluoxetine 0.6221
nefazodone 0.6208
venlafaxine 0.5455
escitalopram 0.5217
sertraline 0.5190
dothiepin 0.5139
trazodone 0.5061
duloxetine 0.5050
desipramine 0.5049
trimipramine 0.4925
imipramine 0.4924
clomipramine 0.4924
placebo 0.4716
nomifensine 0.4531
citalopram 0.3825
amitriptyline 0.3681
mirtazapine 0.2944
nortriptyline 0.2688

**Supplement S. Changes to the original protocol**

The following changes to the original protocol were made:

- For the primary outcome, whether mean change was not available, we looked for the endpoint score, according to Ostinelli et al. Res Synth Methods 2024;15(5):758-768, although no further studies were included in the final analysis;
- We conducted a sensitivity analysis which was not pre-planned by removing trials that included participants with minor or minor-to-moderate severity of the physical condition. The sensitivity analysis was conducted for both efficacy and acceptability;
- In the original protocol, we planned to calculate a treatment hierarchy by means of surface under the cumulative ranking curve (SUCRA) and mean ranks. However, since we performed the analyses with the R *netmeta* package, we used p-scores instead (Rücker G, Schwarzer G. BMC Med Res Methodol 2015:15:58).
